# Supplementary material for: Framework to construct and interpret latent class trajectory modelling
Source: BMJ Open. 2018 Jul 7;8(7):e020683. doi: 10.1136/bmjopen-2017-020683 (PMC6042544; doi:10.1136/bmjopen-2017-020683)
Supplement: Supplementary file 1 [file bmjopen-2017-020683supp001.pdf]

SUPPLEMENTAL MATERIAL

A framework to construct and interpret latent class trajectory modelling

Hannah Lennon[1,3] Scott Kelly,[2] Matthew Sperrin,[3] Iain Buchan,[3] Amanda J Cross,[4] Michael Leitzmann,[5] Michael B Cook,[2] Andrew G Renehan[1,3, 6]

Table S1 Different models with increasing complexity, associated software and referenced use in literature

Model	Description	Interpretation	Software *Command*	References
A	Fixed effects homoscedastic (common residual variance across classes)	No random effects – with the interpretation that any deviation of an individuals trajectory from its mean class trajectory is due to random error only	SAS traj *PROC TRAJ*	(1-5)
B	Fixed effects heteroscedastic (class-specific residual variances)	The same interpretation as Model A with random errors that can be larger and smaller in different classes.	R mmlcr *mmlcr*	(6)
C	Random intercept	The interpretation is allowing individuals to vary in initial weight but each class member is assumed to follow the same shape and magnitude of the mean trajectory	SAS traj *PROC TRAJ*	(7)
D	Random slope	Allowing individuals to vary in initial weight and slope of the mean trajectory but same curvature as trajectory	SAS traj *PROC TRAJ*	(7)
E	Random quadratic – Common variance structure across classes	Additional freedom of allowing individuals to vary within classes by initial weight, shape and magnitude, however each class is assumed to have the same amount of variability	R lcmm *hlme/lcmm*	
F	Random quadratic – Proportionality constraint to allow variance structures to vary across classes	Increasing flexibility of model E as variance structures are allowed to differ up to a multiplicative factor to allow some classes to have larger or smaller within-class variances. This model is can be thought of more parsimonious version of model G from (reducing the number of variance-covariance parameters to be estimated from 6xK parameters to 6+(K-1) parameters.	R lcmm *hlme/lcmm*	
G	Random quadratic – Class-specific variance structure (unstructured)	The most flexible model in which each class has its own separate random quadratic variance structure to describe its own within-class variability. Statistically this permits the variance and covariance of the intercept, slope and quadratic term to vary freely across all classes.	SAS traj *PROC TRAJ*	

[1]The SAS traj package has been converted for Stata users as the traj command in Stata (Collage Station, TX, USA).

More detailed description of model structure

For N individuals, the latent class trajectory model we consider is given by:

$$BMI_{itk} = \beta_0^k + \beta_1^k AGE_t + \beta_2^k AGE_t^2 + b_0^k + b_1^k AGE_t + b_2^k AGE_t^2 + \epsilon_t, \qquad \text{(Equation 1)}$$

where BMI_{itk} is the BMI of individual $i = 1, \dots, N$, at time $t = 1, \dots, T$ in class $k = 1, \dots, K$. The random effect b^k is class-specific and follows a multivariate Normal distribution with zero mean and a 3×3 variance-covariance matrix B. For the residual error term ϵ_t, the usual assumption hold, ϵ_t is normally distributed with zero mean and variance σ^2. The probability of an individual belonging to class k is described by a multinomial distribution, i.e.,

$$Pr(individual\ i\ belonging\ to\ class\ k) = \frac{e^{\pi_k}}{\sum_{k=1}^{K} e^{\pi_k}} = \frac{e^{\pi_k}}{e^{\pi_1} + \dots + e^{\pi_K}}, \qquad \text{(Equation 2)}$$

such that π_k are parameters to be estimated in the model. To select the number of latent classes, we assume a working model (Equation 1) for the random effect structure and the criterion used to select the number of classes was the lowest Bayesian Information Criteria (BIC).

More detailed description of model adequacy assessment

Common tools for model adequacy assessment checking are described below in Table S2. Here we give more details of the two extensions of these tools; degree of separation and Elsensohn's residuals (to random effects).

Degrees of separation

A model's ability to detect classes accurately is affected by the degree of separation between latent trajectory curves (8, 9). To describe the separation of latent growth curves, we used the multivariate Mahalanobis distance with the multivariate Mahalanobis distance (D) units defined as:

$$D_{ij} = (\mu_i - \mu_j)^T \Sigma^{-1} (\mu_i - \mu_j), \qquad i, j = 1, \dots K,$$

where μ_i is a $T\times 1$ vector of mean values for class i, and Σ^{-1} is the inverse of a $T \times T$ matrix of sample covariances of at times $t = 1, \dots, T$. The larger the difference, the larger the separation between curves. Peugh and Fan (9) argue that it is reasonable to expect that it is easier to identify heterogeneous latent growth trajectories when the statistical separation distance among the subpopulations is larger than when the separation distance among the latent subpopulations is much smaller.

To give an overall measure of separation for each model, we propose a weighted sum of multivariate Mahalanobis distance matrix with weights being the estimated class proportions, $\widehat{\pi}_\iota$.. Then the degree of separation DoS_K is defined as

$$DoS_K = \sum_{i=1}^{K} \sum_{j=1}^{i} \widehat{\pi}_\iota \widehat{\pi}_J D_{ij}\,, \quad i,j = 1,2,\dots,K.$$

Larger values of DoS_K indicate the mean trajectories are well separated while DoS_K is zero in the special case when all mean trajectories are identical. If the DoS_K value is small, then you may wish to consider a model with fewer classes.

Elsensohn's envelope of residuals

To check the model assumption in fixed effect latent class models, Elsenhohn et al. (6) suggest plotting the local standard deviations of the residuals to check the appropriateness of the model. With the assumption that the residuals are homogeneous over time. To check the appropriateness of each of our model assumptions, we extended their method to include random effects in the models. We compute the local standard deviations of the residuals using the following steps:

1) Compute the observed residuals r_{itk} for each subject i, at time t given the individual is in class k

Equation 1: $\quad r_{itk} = y_{it} - \hat{y}_{itk} = y_{it} - x_i^T \beta^k - z_i^T b^k,$

where y_{itk} is the observed value for individual i at time t in class k and $\hat{y}_{itk}$ is the fitted value of BMI from our fitted model, here the random effect model. 2) Compute the class- and time-specific weighted local variance of the residuals, $Var_w[r_{itk}]$, with weights being p_{ik}, the posterior probabilities of individual i belonging to group k. #) Plot the upper and lower boundaries for the local standard deviations of the residuals

Equation 2: $b_{itk} = \mu_{tk} \pm \sqrt{Var_w[r_{itk}]}$,

$where\ \mu_{tk}$ is the mean value of class k at time t. 4) Plot the boundaries b_{itk} onto the mean trajectory plots.

The shape of the local standard deviation of the residuals indicates the appropriateness of the model assumptions; where non-parallel boundaries indicate heteroscedasticity of residuals suggesting poor model fit, and differing interval widths suggest across group variability may not be fully accounted for. We believe this complements the others metrics well. If the boundaries suggest a poor fit, you may consider a more complex random effect structure to be more suitable, for example, a higher order polynomial.

Figure S1 Standardised residual plots of fixed-effect model (model A) for each class justifying the selection of a random-effect structure in the scoping model

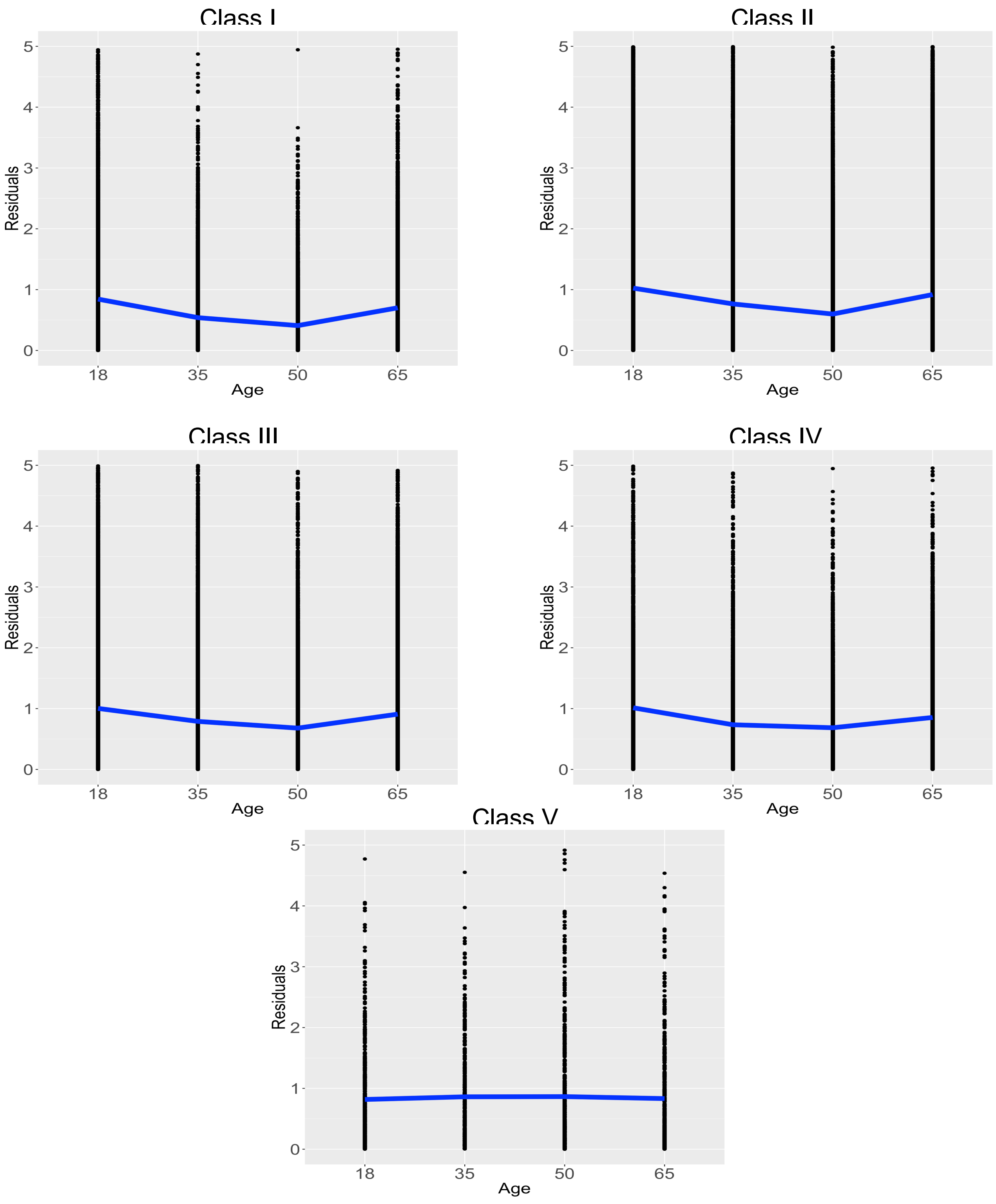

Table S2 Number of classes (K = 1† to 7) using random quadratic effect models: model G (unrestricted) by gender, NIH-AARP cohort

Model	K	Num of parame ters	BIC	Proportions per class (%)						
				Class I	Class II	Class III	Class IV	Class V	Class VI	Class VII
**MEN**										
**Model G**	2	14	3361853	94	6					
	3	21	3341066	87	7	7				
	4	28	3329426	52	37	7	5			
	5	35	**3320005**	51	33	9	6	1		
	6	42	3315240	45	31	12	9	2	1	
	7	48	3311326	51	19	13	7	5	3	0.4
**WOMEN**										
**Model G**	2	14	2219825	89	11					
	3	21	2203726	61	34	0.5				
	4	28	2194293	61	32	5	2			
	5	35	**2187707**	60	29	7	3	1		
	6	42	2184412	63	19	10	4	1	0.5	
	7	48	*							

* Models failed to converge, † Models with K=1 failed to converge

Table S3 Common model selection tools for latent class trajectory models

Tool	Criteria for a good model	Description
Likelihood based measures	Lowest BIC, AIC,	These likelihood based model selection tools include: Bayesian Information Criteria (BIC), Akaike Information Criteria (AIC), Likelihood Ratio Tests (LRT), Lo-Mendel-Rubin (LMT-LRT), Bootstrap-LRT. (See Peugh and Fan, 2015 for discussion) (10)
APPA	APPA > 70% for each class	The average Posterior Probability Assignment, $\bar{p}_k$, looks at whether individuals are assigned with high probability and the overall average probability of assignment to each class.
Odds of correct classification	OCC > 5.0 for each class	The odds of correct classification (OCC) is the ratio of the odds of a correct classification into each group on the basis of the maximum probability classification rule and the estimated class membership proportions, $\widehat{\pi_k}$, $$OCC_k = \frac{\bar{p}_k/(1-\bar{p}_k)}{\widehat{\pi_k}/(1-\widehat{\pi_k})}.$$
Mismatch	Close to 0 for each class	The difference between the estimated class proportions and the class membership proportions once individuals have been assigned to a class, i.e. $$Mismatch_k = \widehat{\pi_k} - \frac{N_k}{N},$$ where N_k is the number of individuals in a class and N is the total number.
Entropy	Close to 0	Entropy is a global measure of classification uncertainty, which takes into account all $N \times K$ posterior probabilities. The entropy of a model is defined as $$E = -\sum_{i=1}^{N}\sum_{k=1}^{K} \hat{p}_{ik} \log \hat{p}_{ik},$$ which takes values from $[0, \infty)$, with higher values indicating a larger amount of uncertainty. Entropy values closest to 0 correspond to models with least classification uncertainty.
Relative Entropy	Close to 1	Relative entropy is a standardised version of entropy in the interval $[0,1]$ defined by $$E_K = 1 - \frac{E}{N \log K},$$ where values close to 1 indicate lowest classification uncertainty. In the special case when there is most uncertainty and each individual has equal probability of belonging to each class, $E_K = 0$. Jedidi et al., describes relative entropy as a relative measure of 'fuzziness', and suggested cause concern when close to zero, as this implies that the latent class centroids are not sufficiently separated (11).

Table S4 Concurrence between fixed effect (Model A) and random quadratic effect LCTM models (Model F and G) in men and women in the NIH-AARP cohort

Models	Cohen's κ_w (95% CI)	Concurrence						
**MEN**						Class		
**Model A and F**	0.31		Model F	I	II	II	IV	V
	(0.27,0.34)	Model A	177453	91106	38437	36430	10118	1362
		Class I	120866	81148	4961	34757	0	0
		Class II	44383	7826	28617	1035	6785	120
		Class III	6723	1405	3611	78	1491	138
		Class IV	4763	619	1076	553	1607	908
		Class V	718	108	172	7	235	196
			Model F	I	II	II	IV	V
		Model G	177453	99897	53372	14978	8320	886
**Model F and G**	0.57	Class I	120866	77962	41688	1117	99	0
	(0.56,0.59)	Class II	44383	21684	11464	6433	4796	6
		Class III	6723	219	10	6389	1	104
		Class IV	4763	17	210	746	3402	388
		Class V	718	15	0	293	22	388
**WOMEN**								
**Model A and F**	0.80		Model F	I	II	II	IV	V
	(0.80,0.80)	Model A	111503	50712	40080	14784	4840	1087
		Class I	36311	36311	0	0	0	0
		Class II	45832	13060	32383	389	0	0
		Class III	23544	1066	6881	13008	2589	0
		Class IV	3898	245	588	969	1501	595
		Class V	1918	30	228	418	750	492
**Model F and G**	0.65		Model F	I	II	II	IV	V
	(0.65,0.65)	Model G	111503	74481	25824	6929	3133	1136
		Class I	36311	36299	9	3	0	0
		Class II	45832	37334	8298	200	0	0
		Class III	23544	840	16896	4320	1463	25
		Class IV	3898	5	617	1098	1658	520
		Class V	1918	3	4	1308	12	591

Table S5 Kappa Matrices† between Models A to G in men and women in the NIH-AARP cohort

Models	Cohen's Kappa Values (95% CI)					
**MEN**				Model		
**Unweighted, κ**						
		Model B	Model C	Model D	Model F	Model G
	Model A	0.64 (0.64, 0.64)	0.20 (0.20, 0.21)	0.20 (0.19, 0.20)	0.38 (0.38, 0.38)	0.13 (0.12, 0.13)
	Model B		0.20 (0.20, 0.20)	0.19 (0.19, 0.19)	0.32 (0.31, 0.32)	0.16 (0.16, 0.17)
	Model C			0.59 (0.58, 0.59)	0.48 (0.47, 0.48)	0.43 (0.42, 0.43)
	Model D				0.50 (0.50, 0.50)	0.49 (0.48, 0.49)
	Model F					0.18 (0.18, 0.19)
**Weighted, κ_w**						
		Model B	Model C	Model D	Model F	Model G
	Model A	0.36 (0.34, 0.38)	0.43 (0.41, 0.44)	0.37 (0.34, 0.41)	0.31 (0.27, 0.34)	0.38 (0.36, 0.39)
	Model B		0.43 (0.42, 0.44)	0.39 (0.37, 0.41)	0.43 (0.42, 0.44)	0.39 (0.38, 0.4)
	Model C			0.49 (0.45, 0.52)	0.65 (0.65, 0.65)	0.62 (0.62, 0.62)
	Model D				0.71 (0.71, 0.71)	0.67 (0.67, 0.67)
	Model F					0.57 (0.56, 0.59)
**WOMEN**						
**Unweighted, κ**						
		Model B	Model C	Model D	Model F	Model G
	Model A	0.64 (0.64, 0.64)	0.65 (0.65, 0.66)	0.25 (0.24, 0.25)	0.63 (0.63, 0.63)	0.37 (0.37, 0.38)
	Model B		0.45 (0.44, 0.45)	0.17 (0.17, 0.17)	0.48 (0.47, 0.48)	0.30 (0.29, 0.30)
	Model C			0.26 (0.25, 0.26)	0.49 (0.49, 0.5)	0.41 (0.41, 0.41)
	Model D				0.28 (0.27, 0.28)	0.52 (0.52, 0.53)
	Model F					0.32 (0.31, 0.32)
**Weighted, κ_w**						
		Model B	Model C	Model D	Model F	Model G
	Model A	0.78 (0.78, 0.78)	0.69 (0.69, 0.69)	0.57 (0.57, 0.57)	0.80 (0.80, 0.80)	0.65 (0.65, 0.65)
	Model B		0.48 (0.48, 0.49)	0.42 (0.41, 0.42)	0.68 (0.68, 0.68)	0.45 (0.45, 0.46)
	Model C			0.64 (0.64, 0.64)	0.71 (0.71, 0.71)	0.63 (0.63, 0.63)
	Model D				0.64 (0.63, 0.66)	0.81 (0.81, 0.81)
	Model F					0.65 (0.65, 0.65)

† Kappa values presented here are optimised over the (5!=120) possible combinations of matching the classes labels across the models

Figure S2 Mean trajectories and 95% prediction intervals for each class in model F – men (left) and women (right)

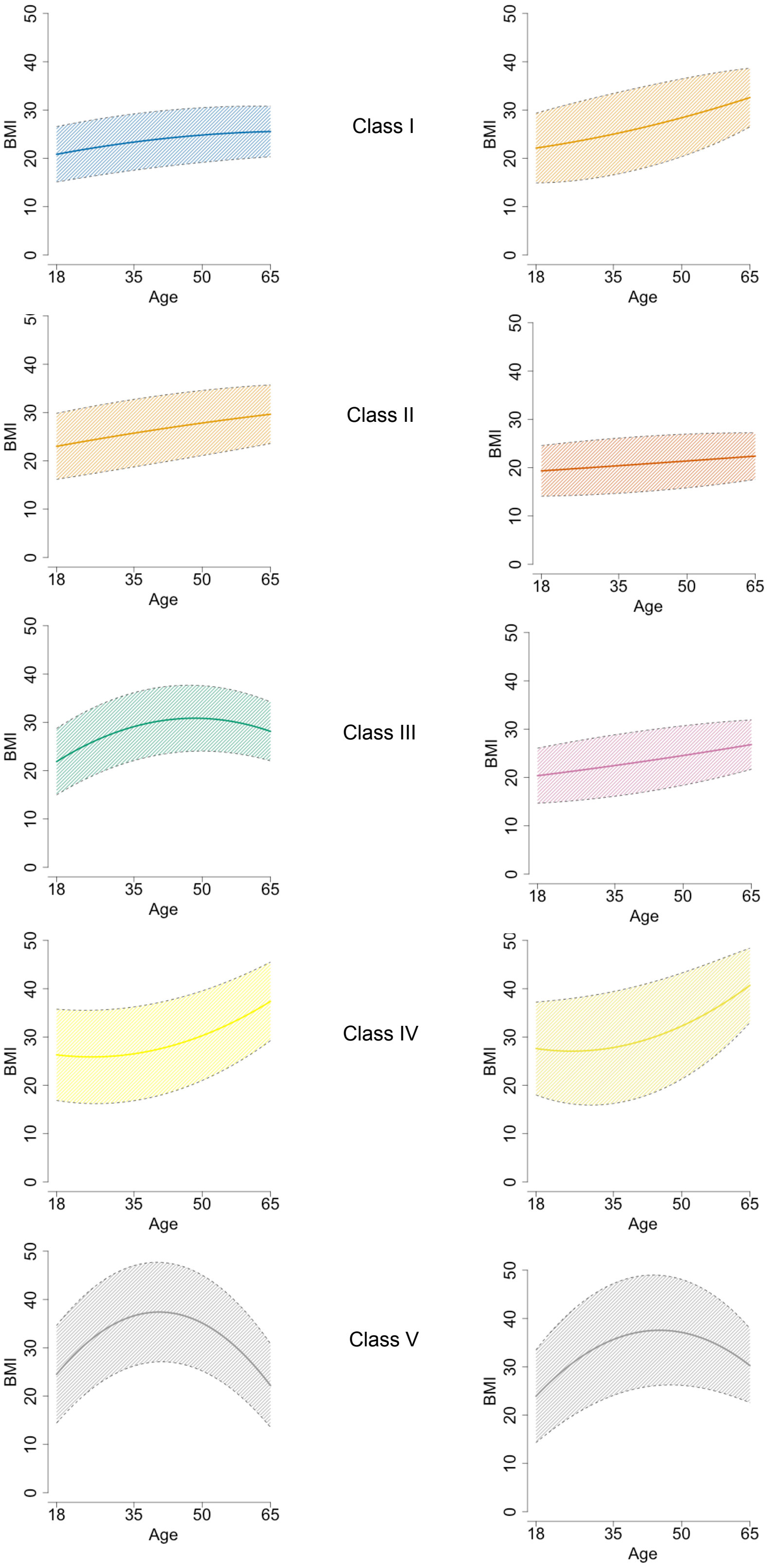

Figure S3 A randomly selected sample of 250 men from each class in model F

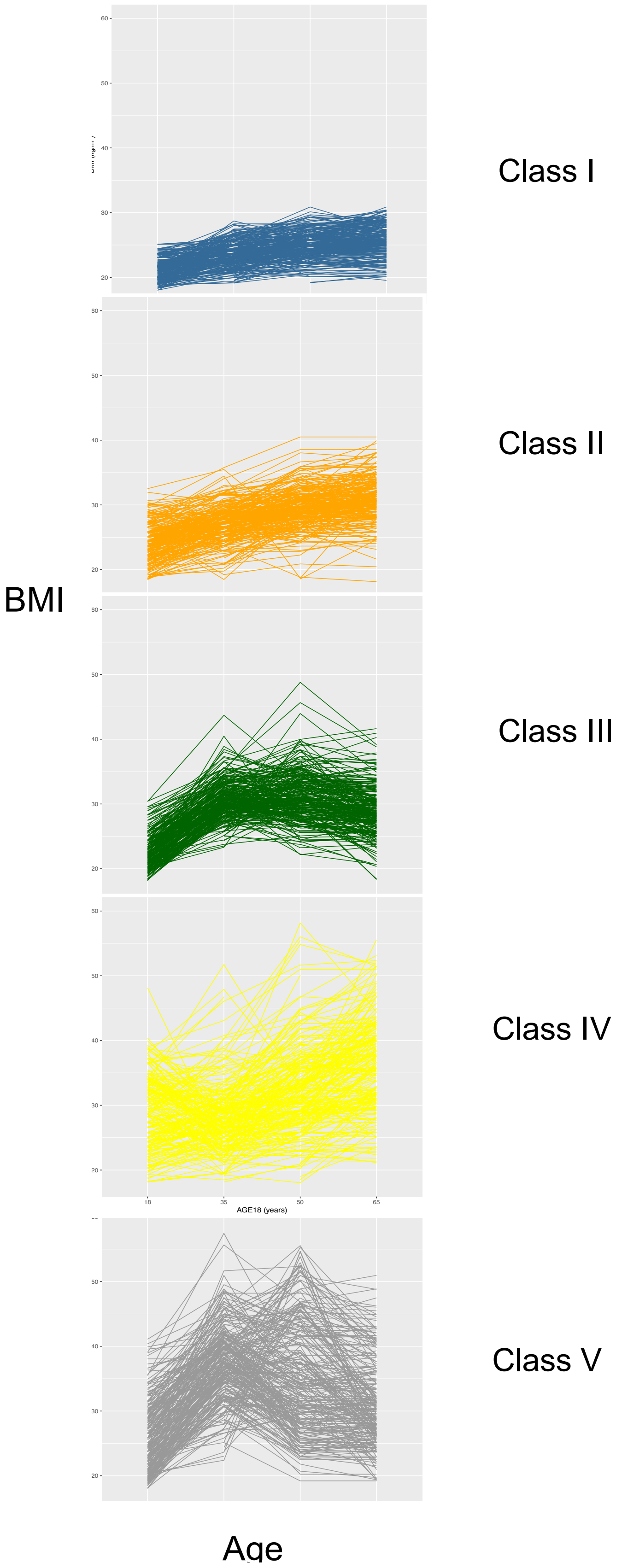

Table S5 Characteristics by five latent classes (Model F) of 177,453 men in the NIH-AARP cohort

	Model F (preferred model)				
	**Class I**	**Class II**	**Class III**	**Class IV**	**Class V**
	Stable normal weight	Normal weight to overweight	Normal to stable obese	Overweight to obese	Rapid increase to obese
Number of subjects, N	120 867 (68%)	44 383 (32%)	6 723 (5%)	4 764 (3%)	718 (0.5%)
Mean (sd) entry age, years	62.88 (0.01)	61.59 (0.03)	62.03 (0.06)	59.71 (0.07)	57.73 (0.18)
Mean (sd) current (at baseline) BMI, kg/m^2	25.39 (0.01)	30.84 (0.02)	29.07 (0.05)	35.14 (0.12)	30.51 (0.28)
Mean (sd) BMI at 50 years, kg/m^2	24.66 (0.01)	29.12 (0.02)	31.34 (0.05)	30.95 (0.12)	35.01 (0.35)
Mean (sd) BMI at 35 years, kg/m^2	23.35 (0.01)	26.72 (0.01)	30.24 (0.04)	26.61 (0.09)	37.69 (0.26)
Mean (sd) BMI at 18 years, kg/m^2	20.75 (0.01)	23.92 (0.02)	21.69 (0.04)	27.16 (0.09)	24.76 (0.2)
Mean (sd) maximum BMI, kg/m^2	26.94 (0.01)	32.76 (0.02)	34.09 (0.05)	37.04 (0.13)	40.17 (0.33)
Mean (sd) minimum BMI, kg/m^2	20.56 (0.01)	23.16 (0.02)	22.46 (0.05)	23.15 (0.07)	24.42 (0.20)
Mean (sd) waist circumference, cm	94.38 (0.02)	106.23 (0.05)	102.73 (0.13)	115.24 (0.28)	105.91 (0.64)
Mean (sd) hip circumference, cm	100.28 (0.02)	109.58 (0.05)	107.02 (0.13)	117.73 (0.25)	110.54 (0.62)
Mean (sd) hip-to-waist ratio,	0.94 (<0.01)	0.97 (<0.01)	0.96 (<0.01)	0.98 (<0.01)	0.96 (<0.01)
Mean (sd) height, m	1.78 (<0.01)	1.79 (<0.01)	1.79 (<0.01)	1.81 (<0.01)	1.78 (<0.01)
Comorbidity, %					
Diabetes	8036 (7%)	6155 (14%)	1999 (30%)	861 (18%)	281 (39%)
Heart disease	19927 (16%)	8709 (20%)	1890 (28%)	908 (19%)	165 (23%)
Smoking, %					
Current	11 823 (10%)	3 761 (8%)	638 (9%)	460 (10%)	106 (15%)
Former	67 948 (56%)	26 898 (61%)	3 809 (57%)	3 002 (63%)	370 (52%)
Never	37 149 (31%)	12 130 (27%)	2 012 (30%)	1 110 (23%)	218 (30%)
Missing	3 946 (3%)	1 594 (4%)	264 (4%)	191 (4%)	24 (3%)
Mean (sd) alcohol consumption, g/day	18.69 (0.13)	17.45 (0.21)	15.48 (0.56)	15.57 (0.69)	10.22 (1.14)
Race, %					
Non-Hispanic White	113 685 (94%)	41 819 (94%)	6 303 (94%)	4 447 (93%)	668 (93%)
Hispanic	2 194 (2%)	1 132 (3%)	157 (2%)	145 (3%)	19 (3%)
Black	1 922 (2%)	650 (1%)	129 (2%)	60 (1%)	14 (2%)
Asian, Pacific islander, American Indian, or Alaskan	2 023 (2%)	343 (1%)	48 (1%)	50 (1%)	4 (1%)

Missing	1 042 (1%)	439 (1%)	86 (1%)	61 (1%)	13 (2%)
Highest Education level achieved, %	5 508 (5%)	2 439 (5%)	415 (6%)	301 (6%)	37 (5%)
Less than 8 years old	16 768 (14%)	6 993 (16%)	1 281 (19%)	813 (17%)	139 (19%)
Completed primary	11 284 (9%)	4 324 (10%)	737 (11%)	474 (10%)	82 (11%)
Completed high school	25 929 (21%)	10 708 (24%)	1 674 (25%)	1 239 (26%)	197 (27%)
Post high school or come college	61 377 (51%)	19 919 (45%)	2 616 (39%)	1 936 (41%)	263 (37%)
College graduate or postgraduate	5 508 (5%)	2 439 (5%)	415 (6%)	301 (6%)	37 (5%)
Vigorous physical activity three or more times per week in the last year, %	91 250 (75%)	29 941 (67%)	4 670 (69%)	2 600 (55%)	438 (61%)
**Nutrition**					
At least 3 fruit servings per day, %	88 699 (73%)	31 603 (71%)	4 855 (72%)	3 335 (70%)	519 (72%)
At least 3 vegetable servings per day, %	56 255 (47%)	19 376 (44%)	3 245 (48%)	2 050 (43%)	364 (51%)
Mean (sd) red meat consumption, g/day	72.68 (0.18)	88.78 (0.37)	86.4 (0.99)	104.96 (1.44)	91.76 (3.6)
Mean (sd) calories per day, kcal	2010 (3)	2095 (5)	2089 (13)	2264 (20)	2146 (42)
Mean (sd) calories per day 10 years ago, kcal					
Mean (sd) calories per day age 12, kcal	1355 (2)	1404 (3)	1473 (8)	1491 (10)	1695 (30)
Drug use					
Ibuprofen use currently					
Aspirin use currently	38720 (32%)	15719 (35%)	2621 (39%)	1697 (36%)	266 (37%)
Fibre					
Mean (sd) fibre from diet age 12, mg/day	9.61 (0.01)	9.85 (0.02)	10.31 (0.07)	10.22 (0.08)	11.2 (0.23)
Mean (sd) fibre from diet 10 years ago, mg/day	6.95 (0.01)	6.95 (0.02)	7.43 (0.05)	7.26 (0.06)	7.71 (0.17)
Mean (sd) fibre CSFII, g/day	20.31 (0.03)	20.45 (0.05)	21.58 (0.14)	21.54 (0.19)	22.6 (0.48)
Mean (sd) fibre total NDSR, g/day	20.71 (0.03)	20.81 (0.05)	21.96 (0.14)	21.93 (0.19)	22.81 (0.5)
Calcium					
Mean (sd) calcium supplement, mg	152.09 (0.75)	136.56 (1.16)	143 (3.1)	138.13 (3.59)	159.2 (10.81)
Mean (sd) calcium from diet current, mg	808.96 (1.34)	845.53 (2.46)	874.55 (6.59)	906.76 (9.07)	910.57 (20.65)
Mean (sd) calcium from diet 10 years ago, mg					
Mean (sd) calcium from diet age 12, mg	653.16 (1.08)	653.03 (1.8)	681.39 (4.77)	672.61 (5.63)	734.68 (15.26)
Folate					
Mean (sd) folate supplement, µg/day	209.35 (0.67)	196.89 (1.13)	207.14 (2.92)	197.72 (3.49)	222.93 (9.74)
Mean (sd) folate pre 1998, µg/day	335.71 (0.48)	336.53 (0.83)	349.14 (2.29)	346.99 (2.85)	355.86 (7.87)

Mean (sd) folate NDSR, µg/day	447.13 (0.58)	451.13 (1.03)	463.45 (2.85)	470.15 (3.73)	482.46 (10.16)

sd: standard deviation

Table S6 Characteristics by five latent classes (Model F) of 111,503 women in the NIH-AARP cohort

	Model F (preferred model)				
	Class I	Class II	Class III	Class IV	Class V
	Stable normal weight	Normal weight to overweight	Normal weight to obese	Overweight to obese	Rapid increase to obese
Number of subjects, N	36 311 (32.7%)	45 832 (41.1%)	23 544 (21.1%)	3 898 (3.5%)	1 918 (1.7%)
Mean (sd) entry age, years	62.48 (0.03)	62.23 (0.02)	61.07 (0.03)	59.61 (0.09)	59.69 (0.12)
Mean (sd) current (at baseline) BMI, kg/m^2	21.97 (0.01)	26.66 (0.01)	32.4 (0.03)	37.47 (0.15)	34.4 (0.19)
Mean (sd) BMI at 50 years, kg/m^2	21.16 (0.01)	24.77 (0.01)	29.52 (0.03)	33.51 (0.13)	37.69 (0.2)
Mean (sd) BMI at 35 years, kg/m^2	20.26 (0.01)	22.53 (0.01)	25.51 (0.02)	28.38 (0.1)	36.92 (0.16)
Mean (sd) BMI at 18 years, kg/m^2	19.3 (0.01)	20.44 (0.01)	22.61 (0.02)	29.01 (0.09)	23.97 (0.1)
Mean (sd) maximum BMI, kg/m^2	23.29 (0.01)	28.13 (0.02)	34.72 (0.04)	40.73 (0.14)	43.18 (0.2)
Mean (sd) minimum BMI, kg/m^2	18.57 (0.01)	20.08 (0.01)	21.85 (0.02)	24.05 (0.07)	24.59 (0.11)
Mean (sd) waist circumference, cm	76.19 (0.04)	86.66 (0.05)	97.82 (0.09)	106.29 (0.32)	101.96 (0.41)
Mean (sd) hip circumference, cm	96.78 (0.03)	105.23 (0.04)	114.7 (0.08)	122.14 (0.31)	118.59 (0.42)
Mean (sd) hip-to-waist ratio,	0.78 (<0.01)	0.82 (<0.01)	0.84 (<0.01)	0.84 (<0.01)	0.85 (<0.01)
Mean (sd) height, m	1.65 (<0.01)	1.64 (<0.01)	1.63 (<0.01)	1.63 (<0.01)	1.62 (<0.01)
Comorbidity, %					
Diabetes	680 (2%)	2344 (5%)	3237 (14%)	725 (19%)	604 (31%)
Heart disease	2269 (6%)	3677 (8%)	2518 (11%)	447 (11%)	296 (15%)
Smoking, %					
Current	5803 (16%)	5660 (12%)	2733 (12%)	495 (13%)	214 (11%)
Former	14173 (39%)	18557 (40%)	9748 (41%)	1764 (45%)	731 (38%)
Never	15313 (42%)	20290 (44%)	10417 (44%)	1507 (39%)	909 (47%)
Missing	1022 (3%)	1325 (3%)	646 (3%)	132 (3%)	64 (3%)
Mean (sd) alcohol consumption, g/day	8.07 (0.1)	6.26 (0.08)	4.47 (0.11)	3.92 (0.29)	3.4 (0.48)
Race, %					
Non-Hispanic White	34468 (95%)	42261 (92%)	21178 (90%)	3446 (88%)	1745 (91%)

Hispanic	699 (2%)	1882 (4%)	1563 (7%)	293 (8%)	105 (5%)
Black	369 (1%)	739 (2%)	324 (1%)	58 (1%)	26 (1%)
Asian, Pacific islander, American Indian, or Alaskan	467 (1%)	443 (1%)	174 (1%)	35 (1%)	15 (1%)
Missing	308 (1%)	507 (1%)	305 (1%)	66 (2%)	27 (1%)
Highest Education level achieved, %					
Less than 8 years old	1155 (3%)	2151 (5%)	1457 (6%)	225 (6%)	124 (6%)
Completed primary	7578 (21%)	11545 (25%)	5984 (25%)	922 (24%)	524 (27%)
Completed high school	3700 (10%)	5021 (11%)	2771 (12%)	489 (13%)	258 (13%)
Post high school or come college	9801 (27%)	11877 (26%)	6088 (26%)	1038 (27%)	521 (27%)
College graduate or postgraduate	14077 (39%)	15238 (33%)	7244 (31%)	1224 (31%)	491 (26%)
Vigorous physical activity three or more times per week in the last year, %	25959 (71%)	30035 (66%)	12692 (54%)	1809 (46%)	1011 (53%)
**Nutrition**					
At least 3 fruit servings per day, %	28441 (78%)	35607 (78%)	17801 (76%)	2889 (74%)	1473 (77%)
At least 3 vegetable servings per day, %	22030 (61%)	27235 (59%)	13282 (56%)	2184 (56%)	1163 (61%)
Mean (sd) red meat consumption, g/day	40.46 (0.19)	46.46 (0.19)	54.41 (0.33)	60.98 (0.98)	56.86 (1.22)
Mean (sd) calories per day, kcal	1546 (4)	1557 (3)	1634 (5)	1727 (15)	1688 (20)
Mean (sd) calories per day 10years ago, kcal	817 (2)	853 (2)	889 (3)	901 (7)	961 (10)
Mean (sd) calories per day age 12, kcal	1250 (3)	1261 (3)	1290 (4)	1336 (10)	1356 (14)
Drug use					
Ibuprofen use currently	4250 (12%)	7065 (15%)	4953 (21%)	1025 (26%)	474 (25%)
Aspirin use currently	6627 (18%)	9559 (21%)	5221 (22%)	913 (23%)	492 (26%)
Hormone Therapy Use, %					
Ever	13989 (39%)	20206 (44%)	12215 (52%)	2241 (57%)	1092 (57%)
Never	22322 (61%)	25626 (56%)	11329 (48%)	1657 (43%)	826 (43%)
Fibre					
Mean (sd) fibre from diet 10years ago, mg/day	7.14 (0.02)	7.22 (0.02)	7.31 (0.03)	7.45 (0.06)	7.57 (0.09)
Mean (sd) fibre from diet age 12, mg/day	8.82 (0.02)	8.96 (0.02)	9.17 (0.03)	9.45 (0.08)	9.39 (0.11)
Mean (sd) fibre CSFII, g/day	18.01 (0.05)	17.75 (0.04)	18.04 (0.06)	18.78 (0.17)	19.14 (0.26)
Mean (sd) fibre total NDSR, g/day	18.05 (0.05)	17.78 (0.04)	18.08 (0.07)	18.85 (0.17)	19.17 (0.26)
Calcium					

Mean (sd) calcium supplement, mg/day	466.85 (2.53)	393.26 (2.1)	337.9 (2.79)	318.84 (6.84)	341.76 (10.22)
Mean (sd) calcium from diet current, mg/day	712.35 (2.23)	716.94 (1.96)	733.95 (2.97)	779.53 (8.16)	765.72 (10.81)
Mean (sd) calcium from diet 10 years ago, mg/day	384.29 (1.18)	387.85 (1.04)	392 (1.5)	390.64 (3.67)	411.4 (5.56)
Mean (sd) calcium from diet age 12, mg/day	677 (2.04)	659.92 (1.81)	650.42 (2.55)	652.48 (6.35)	651.64 (9.03)
Folate					
Mean (sd) folate supplement, µg/day	252.3 (1.23)	239.96 (1.1)	227.06 (1.54)	217.19 (3.78)	228.85 (5.44)
Mean (sd) folate pre 1998, µg/day	291.37 (0.79)	286.07 (0.67)	288.08 (1.02)	295.47 (2.65)	297.1 (3.77)
Mean (sd) folate NDSR, µg/day	375.26 (0.96)	371.15 (0.82)	377.73 (1.26)	389.45 (3.31)	391.35 (4.83)

sd: standard deviation

Table S7 Characteristics by BMI categories of 177,453 men in the AARP cohort

	Normal weight	Overweight	Obese I	Obese II	Obese III
Number of subjects, N (%)	53466 (30.1)	86967 (49.0)	28234 (15.9)	6033 (3.4)	1878 (1.1)
Mean (sd) entry age, years	62.83 (0.02)	62.47 (0.02)	61.87 (0.03)	61.16 (0.07)	60.48 (0.12)
Mean (sd) current (at baseline) BMI, kg/m^2					
Mean (sd) BMI at 50 years, kg/m^2	23.18 (0.01)	27.16 (0)	31.9 (0.01)	36.86 (0.02)	43.93 (0.1)
Mean (sd) BMI at 35 years, kg/m^2	23.28 (0.01)	26.25 (0.01)	29.74 (0.02)	33.33 (0.05)	38.15 (0.15)
Mean (sd) BMI at 18 years, kg/m^2	22.66 (0.01)	24.64 (0.01)	26.89 (0.02)	29.07 (0.05)	31.7 (0.12)
	20.65 (0.01)	21.75 (0.01)	23.14 (0.02)	24.46 (0.05)	25.92 (0.11)
Mean (sd) maximum BMI, kg/m^2					
Mean (sd) minimum BMI, kg/m^2	25.19 (0.01)	28.89 (0.01)	33.58 (0.02)	38.61 (0.05)	45.63 (0.14)
	20.05 (0.01)	21.47 (0.01)	22.85 (0.02)	24.17 (0.05)	25.57 (0.11)
Mean (sd) waist circumference, cm					
Mean (sd) hip circumference, cm	89.56 (0.03)	98.19 (0.03)	108.65 (0.05)	119.1 (0.12)	132.59 (0.29)
Mean (sd) hip-to-waist ratio,	96.92 (0.03)	103.26 (0.02)	111.41 (0.05)	120.44 (0.14)	132.79 (0.36)
Mean (sd) height, m	0.93 (0)	0.96 (0)	0.98 (0)	1 (0)	1 (0)
	70.23 (0.01)	70.07 (0.01)	70.18 (0.02)	69.93 (0.04)	69.7 (0.08)
Comorbidity, %					
Diabetes	3194 (6%)	7752 (9%)	4375 (15%)	1404 (23%)	555 (30%)
Heart disease	8624 (16%)	15314 (18%)	5788 (21%)	1312 (22%)	410 (22%)
Smoking, %					
Current	6513 (12%)	7345 (8%)	2219 (8%)	427 (7%)	129 (7%)
Former	27129 (51%)	51742 (59%)	17719 (63%)	3818 (63%)	1203 (64%)
Never	18089 (34%)	27129 (51%)	7278 (26%)	1557 (26%)	484 (26%)
Missing	1735 (3%)	2948 (3%)	1018 (4%)	231 (4%)	62 (3%)
Mean (sd) alcohol consumption per day, g	18.24 (0.19)	18.36 (0.15)	18.01 (0.28)	16.41 (0.62)	11.72 (0.88)
Race, %					
Non-Hispanic White	50172 (94%)	81972 (94%)	26548 (94%)	5675 (94%)	1738 (93%)
Hispanic	843 (2%)	1775 (2%)	777 (3%)	171 (3%)	68 (4%)
Black	750 (1%)	1448 (2%)	454 (2%)	86 (1%)	28 (1%)
Asian, Pacific islander, American Indian, or Alaskan	1222 (2%)	1001 (1%)	176 (1%)	34 (1%)	15 (1%)
Missing	479 (1%)	771 (1%)	279 (1%)	67 (1%)	29 (2%)

Highest Education level achieved, %					
Less than 8 years old	2136 (4%)	4259 (5%)	1695 (6%)	422 (7%)	148 (8%)
Completed primary	6591 (12%)	13066 (15%)	4805 (17%)	1075 (18%)	356 (19%)
Completed high school	4643 (9%)	8406 (10%)	2930 (10%)	639 (11%)	209 (11%)
Post high school or come college	10728 (20%)	19668 (23%)	7120 (25%)	1540 (26%)	498 (27%)
College graduate or postgraduate	29368 (55%)	41568 (48%)	11684 (41%)	2357 (39%)	667 (36%)
Vigorous physical activity three or more times per week in the last year, %	41480 (78%)	64458 (74%)	18280 (65%)	3284 (54%)	786 (42%)
**Nutrition**					
At least 3 fruit servings per day, %	40079 (75%)	62921 (72%)	19870 (70%)	4203 (70%)	1303 (69%)
At least 3 vegetable servings per day, %	32887 (62%)	49213 (57%)	15003 (53%)	3220 (53%)	1037 (55%)
Mean (sd) red meat consumption, g/day	66.09 (0.28)	77.79 (0.21)	93.13 (0.44)	107.21 (1.09)	124.23 (2.72)
Mean (sd) calories per day, kcal	1997.3 (3.96)	2019.26 (3.08)	2123.92 (6.21)	2244.87 (14.7)	2411.64 (34.3)
Mean (sd) calories per day 10 years ago, kcal	861.51 (1.57)	865.75 (1.28)	896.59 (2.4)	936.47 (5.47)	974.51 (10.19)
Mean (sd) calories per day age 12, kcal	667.55 (1.63)	650.14 (1.28)	645.21 (2.27)	657.3 (4.93)	667.25 (9.06)
Drug use					
Ibuprofen use currently,	3969 (15%)	8036 (17%)	3688 (23%)	973 (27%)	408 (36%)
Missing	27029 (51%)	39942 (46%)	11894 (42%)	2460 (41%)	743 (40%)
Aspirin use currently,	16456 (40%)	29091 (43%)	10360 (47%)	2174 (47%)	699 (50%)
Missing	12646 (24%)	18622 (21%)	6146 (22%)	1444 (24%)	469 (25%)
Fibre					
Mean (sd) fibre from diet age 12, mg	9.65 (0.02)	9.69 (0.02)	9.84 (0.03)	10.04 (0.07)	10.3 (0.13)
Mean (sd) fibre from diet 10 years ago, mg	7.12 (0.01)	6.89 (0.01)	6.95 (0.02)	7.1 (0.05)	7.35 (0.09)
Mean (sd) fibre CSFII, g	20.95 (0.05)	20.11 (0.03)	20.26 (0.06)	20.85 (0.14)	22.3 (0.28)
Mean (sd) fibre total NDSR, g	21.35 (0.05)	20.49 (0.03)	20.66 (0.07)	21.22 (0.15)	22.65 (0.29)
Calcium					
Mean (sd) calcium supplement, mg	811.3 (2)	812.92 (1.62)	852.6 (3.12)	906.94 (7.45)	973.28 (16.08)
Mean (sd) calcium from diet current, mg	163.44 (1.18)	144.71 (0.85)	131.79 (1.43)	127.03 (3.03)	122.25 (5.34)
Mean (sd) calcium from diet 10 years ago, mg	406 (1)	398.45 (0.79)	404.71 (1.45)	422.22 (3.28)	426.44 (5.87)
Mean (sd) calcium from diet age 12, mg	667.55 (1.63)	650.14 (1.28)	645.21 (2.27)	657.3 (4.93)	667.25 (9.06)
Folate					

Mean (sd) folate supplement, g	342.65 (0.74)	332.76 (0.56)	335.07 (1.04)	343.98 (2.3)	360.39 (4.58)
Mean (sd) folate pre 1998, g	217.7 (1.01)	205.22 (0.8)	191.35 (1.41)	186.39 (3.01)	180.32 (5.43)
Mean (sd) folate NDSR, g	455.75 (0.92)	443.69 (0.69)	449.63 (1.3)	464.98 (2.95)	490.92 (5.98)

sd: standard deviation

N=875 men classified as <18.0 kg/m^2 at study entry not included

Table S8 Characteristics by BMI categories of 111,503 women in the AARP cohort

	Normal weight	Overweight	Obese I	Obese II	Obese III
Number of subjects, N (%)	48273 (43.3)	36025 (32.2)	16245 (14.6)	6126 (5.5.)	3507 (3.1)
Mean (sd) entry age, years	61.96 (0.02)	62.15 (0.03)	61.89 (0.04)	61.21 (0.07)	60.45 (0.09)
Mean (sd) current (at baseline) BMI, kg/m^2	22.45 (0.01)	27.23 (0.01)	32.12 (0.01)	37.14 (0.02)	44.66 (0.08)
Mean (sd) BMI at 50 years, kg/m^2	22.04 (0.01)	25.31 (0.01)	28.75 (0.03)	32.46 (0.06)	38.33 (0.12)
Mean (sd) BMI at 35 years, kg/m^2	21.12 (0.01)	22.93 (0.01)	24.93 (0.03)	27.22 (0.06)	30.72 (0.11)
Mean (sd) BMI at 18 years, kg/m^2	20.11 (0.01)	20.83 (0.01)	21.88 (0.03)	23.03 (0.05)	24.46 (0.08)
Mean (sd) maximum BMI, kg/m^2	24.21 (0.01)	28.88 (0.02)	34.05 (0.03)	39.21 (0.06)	46.57 (0.11)
Mean (sd) minimum BMI, kg/m^2	19.16 (0.01)	20.3 (0.01)	21.44 (0.02)	22.57 (0.05)	24.24 (0.08)
Mean (sd) waist circumference, cm	76.99 (0.04)	88.19 (0.05)	97.92 (0.08)	106.84 (0.13)	120.12 (0.23)
Mean (sd) hip circumference, cm	97.47 (0.03)	106.73 (0.04)	115.24 (0.07)	124.36 (0.14)	137.66 (0.24)
Mean (sd) hip-to-waist ratio,	0.79 (0)	0.83 (0)	0.85 (0)	0.86 (0)	0.85 (0)
Mean (sd) height, m	1218 (3%)	2199 (6%)	2050 (13%)	1211 (20%)	873 (25%)
Comorbidity, %					
Diabetes	1218 (3%)	2199 (6%)	2050 (13%)	1211 (20%)	873 (25%)
Heart disease	3050 (6%)	3043 (8%)	1749 (11%)	779 (13%)	482 (14%)
Smoking, %					
Current	7699 (16%)	4467 (12%)	1584 (10%)	502 (8%)	256 (7%)
Former	18632 (39%)	14883 (41%)	6843 (42%)	2633 (43%)	1586 (45%)
Never	20558 (43%)	15636 (43%)	7380 (45%)	2823 (46%)	1552 (44%)
Missing	1384 (3%)	1039 (3%)	438 (3%)	168 (3%)	113 (3%)
Mean (sd) alcohol consumption per day, g	7.94 (0.09)	5.92 (0.09)	4.3 (0.13)	3.25 (0.21)	2.67 (0.26)
Race, %					
Non-Hispanic White	45651 (95%)	33005 (92%)	14627 (90%)	5471 (89%)	3090 (88%)
Hispanic	988 (2%)	1688 (5%)	1067 (7%)	472 (8%)	299 (9%)
Black	582 (1%)	581 (2%)	225 (1%)	74 (1%)	45 (1%)
Asian, Pacific islander, American Indian, or Alaskan	620 (1%)	333 (1%)	114 (1%)	32 (1%)	16 (0%)
Missing	432 (1%)	418 (1%)	212 (1%)	77 (1%)	57 (2%)

Highest Education level achieved, %					
Less than 8 years old	1585 (3%)	1797 (5%)	1017 (6%)	417 (7%)	261 (7%)
Completed primary	10367 (21%)	9077 (25%)	4288 (26%)	1604 (26%)	954 (27%)
Completed high school	4914 (10%)	4138 (11%)	1869 (12%)	740 (12%)	459 (13%)
Post high school or come college	12696 (26%)	9375 (26%)	4327 (27%)	1644 (27%)	938 (27%)
College graduate or postgraduate	18711 (39%)	11638 (32%)	4744 (29%)	1721 (28%)	895 (26%)
Vigorous physical activity three or more times per week in the last year, %	34629 (72%)	23170 (64%)	8917 (55%)	2694 (44%)	1241 (35%)
**Nutrition**	37782 (78%)	27905 (77%)	12355 (76%)	4596 (75%)	2559 (73%)
At least 3 fruit servings per day, %	34422 (71%)	24747 (69%)	10907 (67%)	3921 (64%)	2257 (64%)
At least 3 vegetable servings per day, %					
Mean (sd) red meat consumption, g/day	40.35 (0.17)	47.73 (0.22)	54.09 (0.39)	61.25 (0.67)	71.18 (1.07)
Mean (sd) calories per day, kcal	1531.86 (2.99)	1568.13 (3.81)	1626.68 (6.33)	1696.9 (10.28)	1864.32 (16.6)
Mean (sd) calories per day 10years ago, kcal	818.42 (1.6)	859.29 (2.01)	894.97 (3.17)	910.64 (5.15)	962.24 (7.27)
Mean (sd) calories per day age 12, kcal	673.14 (1.77)	658.24 (2.05)	650.22 (3.07)	646.33 (5.04)	657.19 (6.67)
Hormone Therapy Use, %					
Never	18932 (39%)	16154 (45%)	8334 (51%)	3459 (56%)	2261 (64%)
Ever	29341 (61%)	19871 (55%)	7911 (49%)	2667 (44%)	1246 (36%)
Ibuprofen use currently,	5842 (21%)	5795 (26%)	3352 (33%)	1566 (39%)	1098 (47%)
Ibuprofen use missing	20318 (42%)	14129 (39%)	6018 (37%)	2075 (34%)	1191 (34%)
Aspirin use currently,	8875 (28%)	7709 (32%)	3714 (36%)	1445 (38%)	833 (41%)
Asprin use missing	16220 (34%)	12185 (34%)	5777 (36%)	2364 (39%)	1483 (42%)
,					
Fibre					
Mean (sd) fibre from diet 10years ago, mg	8.82 (0.02)	9.01 (0.02)	9.14 (0.04)	9.32 (0.06)	9.5 (0.08)
Mean (sd) fibre from diet age 12, mg	7.14 (0.02)	7.22 (0.02)	7.36 (0.03)	7.34 (0.05)	7.65 (0.07)
Mean (sd) fibre CSFII, g	18 (0.04)	17.74 (0.05)	17.94 (0.08)	18 (0.12)	19.35 (0.18)
Mean (sd) fibre total NDSR, g	18.05 (0.04)	17.77 (0.05)	17.98 (0.08)	18.05 (0.12)	19.43 (0.18)
Calcium					
Mean (sd) calcium supplement, mg	710.52 (1.9)	719.2 (2.26)	733.66 (3.61)	751.64 (5.75)	808.42 (8.73)
Mean (sd) calcium from diet current, mg	458.8 (2.18)	382.25 (2.35)	333.6 (3.31)	307.85 (5.3)	270.5 (6.54)
Mean (sd) calcium from diet 10 years ago, mg	382.09 (1.01)	388.3 (1.19)	397.37 (1.82)	397.86 (2.95)	414.1 (4.09)

Mean (sd) calcium from diet age 12, mg	673.14 (1.77)	658.24 (2.05)	650.22 (3.07)	646.33 (5.04)	657.19 (6.67)
Folate					
Mean (sd) folate supplement, g	250.24 (1.07)	239.5 (1.25)	225.57 (1.84)	217.96 (3.02)	209.48 (4.03)
Mean (sd) folate pre 1998, g	290.16 (0.68)	285.89 (0.78)	287.43 (1.2)	288.09 (1.98)	304.36 (2.79)
Mean (sd) folate NDSR, g	373.99 (0.82)	371.32 (0.96)	376.02 (1.49)	380.5 (2.43)	407.36 (3.5)

sd: standard deviation
N=1327 Women classified as <18.0 kg/m^2 at study entry not included

Figure S4 Latent Class trajectories derived on individuals with at least three time points, with random quadratic effect proportional specification (model F) (left: Men; right: Women)

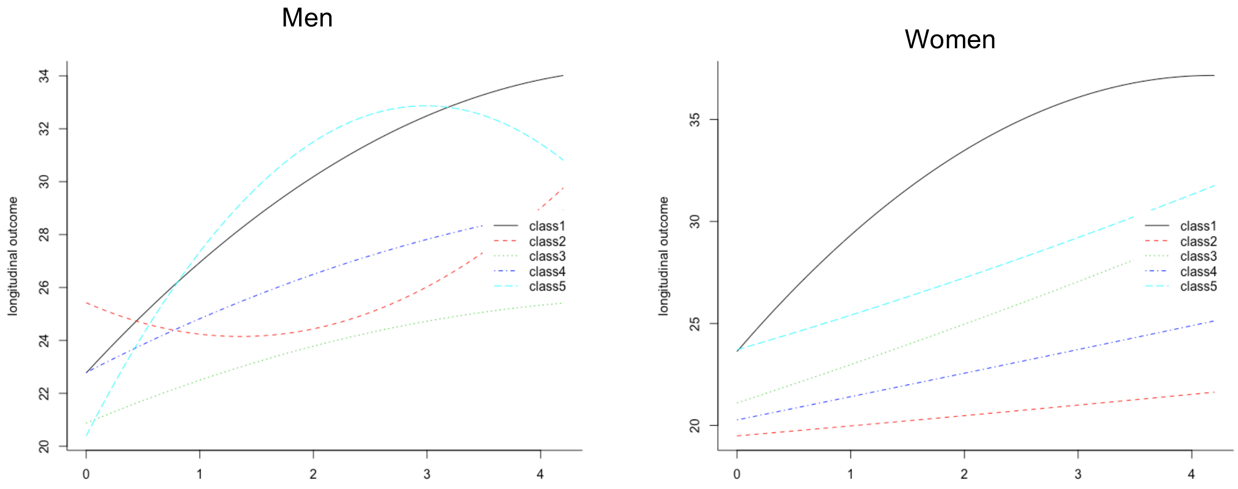

REFERENCES

1. Kubzansky LD, Gilthorpe MS, Goodman E. A prospective study of psychological distress and weight status in adolescents/young adults. Annals of Behavioral Medicine 2012;43(2):219-228.

2. Kelly SP, Graubard BI, Andreotti G, Younes N, Cleary SD, Cook MB. Prediagnostic Body Mass Index Trajectories in Relation to Prostate Cancer Incidence and Mortality in the PLCO Cancer Screening Trial. J Natl Cancer Inst 2017;109(3).

3. Song M, Willett WC, Hu FB, Spiegelman D, Must A, Wu K, et al. Trajectory of body shape across the lifespan and cancer risk. Int J Cancer 2016;138(10):2383-95.

4. Song M, Hu FB, Wu K, Must A, Chan AT, Willett WC, et al. Trajectory of body shape in early and middle life and all cause and cause specific mortality: results from two prospective US cohort studies. BMJ 2016;353:i2195.

5. Zheng H, Tumin D, Qian Z. Obesity and Mortality Risk: New Findings From Body Mass Index Trajectories. American Journal of Epidemiology 2013;178(11):1591-1599.

6. Elsensohn M-H, Klich A, Ecochard R, Bastard M, Genolini C, Etard J-F, et al. A graphical method to assess distribution assumption in group-based trajectory models. Statistical methods in medical research 2016;25(2):968-982.

7. Vistisen D, Witte DR, Tabak AG, Herder C, Brunner EJ, Kivimaki M, et al. Patterns of obesity development before the diagnosis of type 2 diabetes: the Whitehall II cohort study. PLoS Med 2014;11(2):e1001602.

8. Lubke G, Neale MC. Distinguishing between latent classes and continuous factors: Resolution by maximum likelihood? Multivariate Behavioral Research 2006;41(4):499-532.

9. Peugh J, Fan X. How Well Does Growth Mixture Modeling Identify Heterogeneous Growth Trajectories? A Simulation Study Examining GMM's Performance Characteristics. Structural Equation Modeling: A Multidisciplinary Journal 2012;19(2):204-226.

10. Peugh J, Fan X. Enumeration Index Performance in Generalized Growth Mixture Models: A Monte Carlo Test of Muthén’s (2003) Hypothesis. Structural Equation Modeling: A Multidisciplinary Journal 2015;22(1):115-131.

11. Jedidi K, Ramaswamy V, Desarbo WS. A maximum likelihood method for latent class regression involving a censored dependent variable. Psychometrika 1993;58(3):375-394.
