# Supplementary material for: Framework to construct and interpret latent class trajectory modelling
Source: BMJ Open. 2018 Jul 7;8(7):e020683. doi: 10.1136/bmjopen-2017-020683 (PMC6042544; doi:10.1136/bmjopen-2017-020683)
Supplement: Author's manuscript [file bmjopen-2017-020683.draft_revisions.pdf]

BMJ Open

BMJ Open is committed to open peer review. As part of this commitment we make the peer review history of every article we publish publicly available.

When an article is published we post the peer reviewers' comments and the authors' responses online. We also post the versions of the paper that were used during peer review. These are the versions that the peer review comments apply to.

The versions of the paper that follow are the versions that were submitted during the peer review process. They are not the versions of record or the final published versions. They should not be cited or distributed as the published version of this manuscript.

BMJ Open is an open access journal and the full, final, typeset and author-corrected version of record of the manuscript is available on our site with no access controls, subscription charges or pay-per-view fees (http://bmjopen.bmj.com).

If you have any questions on BMJ Open's open peer review process please email
info.bmjopen@bmj.com

BMJ Open

A framework to construct and interpret latent class trajectory modelling

Journal:	*BMJ Open*
Manuscript ID	bmjopen-2017-020683
Article Type:	Research
Date Submitted by the Author:	16-Nov-2017

Complete List of Authors:	Lennon, Hannah; University of Manchester, Division of Cancer Sciences; University of Manchester Institute of Population Health<br>Kelly, Scott; National Institutes of Health, Division of Cancer Epidemiology and Genetics, National Cancer Institute<br>Sperrin, Matthew; University of Manchester Institute of Population Health, Division of Informatics, Imaging and Data Sciences<br>Buchan, Iain; University of Manchester Institute of Population Health, Division of Informatics, Imaging and Data Sciences<br>Cross, Amanda; Imperial College London School of Public Health, Department of Epidemiology and Biostatistics<br>Leitzmann, Michael; Universitat Regensburg Fakultat fur Medizin, Department for Epidemiology and Preventive Medicine<br>Cook , Michael ; National Institutes of Health, Division of Cancer Epidemiology and Genetics, National Cancer Institute<br>Renehan, Andrew; University of Manchester, Division of Cancer Sciences; University of Manchester Institute of Population Health, MRC Health eResearch Centre (HeRC), Division of Informatics, Imaging and Data Sciences,

**Primary Subject Heading**:	Research methods
Secondary Subject Heading:	Health informatics, Epidemiology, Medical education and training, Communication
Keywords:	Latent class models, Growth curves, Growth mixture models, Lifetime obesity, Trajectories

SCHOLARONE™ Manuscripts

Original article (formatted for BMJ Open)

A framework to construct and interpret latent class trajectory modelling

Hannah Lennon, [1,3] Scott Kelly,[2] Matthew Sperrin,[3] Iain Buchan,[3] Amanda J Cross,[4] Michael Leitzmann,[5] Michael B Cook,[2] Andrew G Renehan[1,3]

[1] Division of Cancer Sciences, School of Medical Sciences, Faculty of Biology, Medicine and Health, University of Manchester, Manchester, UK

[2] Division of Cancer Epidemiology and Genetics, National Cancer Institute, National Institutes of Health, Maryland, US

[3] MRC Health eResearch Centre (HeRC), Division of Informatics, Imaging and Data Sciences, University of Manchester, Manchester, UK

[4] Department of Epidemiology and Biostatistics, Imperial College, London, UK

[5] Department of Epidemiology and Preventive Medicine, University of Regensburg, Regensburg, Germany

Running title: *latent class modelling*

Keywords*: Latent class models, growth curves, growth mixture models, body mass index, trajectories, lifetime obesity*

Correspondence to:

Hannah Lennon
Manchester Cancer Research Centre
NIHR Manchester Biomedical Research Centre
Division of Cancer Sciences, School of Medical Sciences,
Faculty of Biology, Medicine and Health,
University of Manchester,
Manchester,
M13 9PL UK
Tel: +44 161 306 7925
E-mail: hannah.lennon@manchester.ac.uk

Abstract: 262 words; main text: 3381 words (max: 3000); 5 tables; 2 figures; References 28 (max: 30); Supplemental material (18 pages); language: UK English.

For peer review only - http://bmjopen.bmj.com/site/about/guidelines.xhtml

ABSTRACT

Objectives: Latent class trajectory modelling (LCTM) is a relatively new methodology in epidemiology to describe life-course exposures, which simplifies heterogeneous populations into homogeneous patterns or classes. Here, we rationalise a systematic framework to construct and interpret these models.

Methods: We developed an eight step framework: step 1, a scoping model; step 2, refining the number of classes; step 3, refining model structure (from fixed-effects through to a flexible random-effect specification); step 4, model adequacy assessment; step 5, graphical presentations; step 6, use of additional discrimination tools ('degree of separation'; Elsensohn's envelope of residual plots); step 7, clinical characterisation and plausibility; and step 8, sensitivity analysis. We illustrated these steps using data from the NIH-AARP cohort of repeated determinations of body mass index (BMI) at baseline (mean age: 62.5 years), and BMI derived by weight recall at ages 18, 35 and 50 years.

Results: From 288,993 participants, we derived a 5-class model for each gender (men: 177,455; women: 111,538). From seven model structures, the preferred model was a proportional random quadratic structure (model F). Favourable properties were also noted for the unrestricted random quadratic structure (model G). However, class proportions varied considerably by model structure – concordance between Models F and G were moderate (Cohen κ: men, 0.57; women, 0.65) but poor with other models. Model adequacy assessments, evaluations using discrimination tools, clinical characterisation and plausibility assessment, and sensitivity analyses supported our model selection.

Conclusion: Latent classes offer an additional disease prevention opportunity as opposed to baseline only approaches. We propose a framework to construct LCTMs that will standardise and facilitate generalisability of results.

Funding: Cancer Research UK National Awareness and Early Detection Initiative (NAEDI), Award Number A17962.

For peer review only

For peer review only - http://bmjopen.bmj.com/site/about/guidelines.xhtml

STRENGTHS AND LIMITATIONS OF THIS STUDY

- We developed a systematic approach, with rationale for each of eight steps, to derive a latent class trajectory model of *preferred* number of classes and model structure specification
- We used a variety of assessment and discrimination tools, clinical characterisation and plausibility, and sensitivity analyses to support model selection
- The results presented here are based on modelling data from a large well-characterised US cohort; allowing the derivation of numerically meaningful subpopulations (i.e., classes) with distinct phenotypes
- Compared with 'one-off' BMI categorisation, BMI latent classes offer additional phenotypic information, and offer a disease prevention opportunity through 'targeting' identifiable and meaningful trajectories
- Our study did not directly measure BMI values in childhood or early adulthood as this would require a different strategy and neither did our study directly capture records of other common disease risk factors, such as smoking, over time (i.e., in a time-varying manner)

For peer review only

For peer review only - http://bmjopen.bmj.com/site/about/guidelines.xhtml

INTRODUCTION

In many epidemiological studies, a risk factor is measured at a single point in time and related to the subsequent development of disease, under the assumption that a single 'one-off' measure is an approximation for that exposure over a long time. Thus, baseline measurement of body mass index (BMI) is associated with subsequent development of common disease like cardiovascular disease (1), diabetes (2), several cancers (3), and all-cause mortality (4). This approach is crude and many investigators seek to use alternative methods that might better capture long-term risk factor exposure termed *life-course analysis*. There are widely-used examples that capture cumulative exposure, such as pack-years for smoking and lung cancer, but the assumption that incidence rate is proportional to total lifetime dose is questionable (5). Many other life-course models simply extract features for use in standard regression approaches; for example, a weight change over time. A more sophisticated approach, which takes account of within-individual correlations, is *mixed-effect modelling*, but this is difficult to interpret for public health implementation. An extension of this approach is the use of *latent classes*, also termed growth mixture models.

Latent class trajectory modelling (LCTM) simplifies heterogeneous populations into more homogeneous clusters or classes. From these, one can potentially include random effects to allow for individual variation within these classes. These models have a long history in the criminology (6) and psychology (7) literatures, and now, are increasingly reported in the human epidemiology literature. Of relevance to this paper, LCTM has been used in association studies of repeated BMI measures with all-cause mortality (8) and cancer incidence (multiple cancer types (9)); gastro-oesophageal (10); prostate (11)) and cancer mortality (11). The LCTM has two general advantages compared with using 'one-off' exposure determinations: first, it better informs aetiological associations by deeply phenotyping certain 'at risk' subpopulations; and second, LCTM offers a public health strategy to identify early divergent adverse trajectories as potential intervention targets. Some researchers additionally argue that LCTM is well-equipped for future forecasting and

For peer review only - http://bmjopen.bmj.com/site/about/guidelines.xhtml

new-patient generalisations in prediction models, as it handles data following a different predictable pattern from that learnt by the model (12).

However, LCTM is a complex form of modelling and requires several different structure assumptions (13). Implementation of different specifications within LCTMs might yield different findings. Although firmly acknowledged in the GRoLTS-Checklist: Guidelines for Reporting on Latent Trajectory Studies (14), structure-related assumptions have not been systematically evaluated. Thus, different observed patterns of latent classes between studies might reflect different modelling assumptions rather than true differences between populations. Here, we propose a framework to build and interpret LCTM, using an example of repeatedly determined body mass index (BMI) across adulthood in the National Institutes of Health (NIH)-AARP Diet and Health Study cohort.

METHODS

Cohort

The NIH-AARP Diet and Health Study is a US cohort of men and women recruited from 1995 (15). A baseline medical and lifestyle questionnaire, including self-reported BMI, was returned by 566,398 participants (aged 50 to 71 years; mean age: 62.5 years). An additional risk factor questionnaire, including recalled weights at different ages was mailed in 1996 and completed by a sub-cohort of 327,860; 288,993 (177,455 men and 111,538 women) provided weight for all four time-points - ages 18, 35 and 50 years, and baseline weight and height, and these are the data included in the present analysis. We excluded participants with extreme BMI values (<15 or >70 kg/m^2) recorded at any time point. Means and standard deviations for derived recalled BMI distributions are representative of BMI distributions for historical period-equivalent US populations (16).

Latent class trajectory modelling

We developed an eight step framework **(Table 1)** modelling BMI as a function of age. Latent classes were used to identify subgroups of participants with distinct trajectories (detailed

For peer review only - http://bmjopen.bmj.com/site/about/guidelines.xhtml

mathematical equations in supplemental material p1) (17). We used maximum likelihood approaches to fit the model with the '*hlme'* function from 'lcmm' library (18) in the R software environment (version 3.2.1) (19) and cross-checked results using the '*PROC TRAJ'* function in 'SAS traj' (SAS Institute, Inc., Cary, NC) (20) (Table S1).

Step 1: We initially constructed a scoping model provisionally selecting the plausible number of classes based on available literature – in the context of BMI trajectories, we used k = 5 classes as reported elsewhere (9, 11). To determine the initial working model structure, we followed the rationale of Verbeke and Molenburgh (21) and examined the shape of standardised residual plots for each of the five classes in a model with no random-effects. If the residual profile could be approximated by a flat, straight line or a curve, then a random intercept, slope or quadratic term, respectively, should be considered. Preliminary plots suggested preference for a quadratic random-effects model (Figure S1).

Step 2: We refined the preliminary working model from step 1 to determine the optimal number of classes, testing k = 1 to 7. We built models for both genders, as BMI patterns of lifetime changes differ for men and women (16). The number of classes chosen was based on the lowest Bayesian Information Criteria (BIC).

Step 3: We further refined the model, using the preferred k derived in step 2, testing for the optimal model structure. We tested seven models (detailed in Table S2): ranging from a simple fixed-effects model (model A) through a rudimentary method that allows the residual variances to vary between classes (model B) to a suite of five random-effects models with different variance structures (models C-G). We selected an optimal model structure using the lowest BIC value and referred to the outcome of steps 2 and 3 as the *preferred model*.

Step 4: We then performed a number of model adequacy assessments. First, we calculated the posterior probability for each participant of being assigned to each trajectory class, and

For peer review only - http://bmjopen.bmj.com/site/about/guidelines.xhtml

assigned the individual to the trajectory with the highest probability. An average of these maximum Posterior Probability of Assignments (APPA) above 70%, in all classes, is regarded as acceptable (6). We further assessed model adequacy using Odds of Correct Classification (OCC), mismatch scores and Entropy, E_k (detailed in Table S3). These diagnostic tools assist in model selection (6, 22) - if these are strongly violated, one might go back to steps 2 and 3 and consider a different model with a higher BIC value.

Step 5: We used three graphical presentation approaches. The conventional approach is to plot mean trajectories with time encompassing each class. Alternatives include the use of mean trajectory plots with 95% predictive intervals for each class, which displays the estimated random variation within each class; or to plot individual level 'spaghetti plots' with time (for example, a random sample of participants), which allows the reader to observe the patterns of changes within classes.

Step 6: We assessed model discrimination, including Degrees of Separation, DoS_K (23, 24) and Elsensohn's envelope of residuals (25). To describe the separation of latent trajectory curves, a multivariate Mahalanobis distance was used. Peugh and Fan (24) argue that it is reasonable to speculate that identification of heterogeneous latent trajectories is facilitated by large statistical separation distance among the subpopulations. Thus, larger values of DoS_K indicate the mean trajectories are well separated while DoS_K equal to zero is the special case when all mean trajectories are identical. If the DoS_K value is small, then one might consider a model with fewer classes.

To check structure assumptions in fixed-effects latent class models, Elsenhohn et al. (25) plotted the local standard deviations of the residuals against time. We extended this method to random-effects models; first, computing the observed residuals for each participant; and second, computing the class- and time-specific weighted local variance of the residuals, with weights being the posterior probabilities of individual belonging to a class. We plotted the upper and lower boundary values of the local standard deviations of the

For peer review only - http://bmjopen.bmj.com/site/about/guidelines.xhtml

residuals around the mean values for each class. The resulting shape indicates the appropriateness of the model assumptions; where non-parallel boundaries indicate heteroscedasticity of residuals suggesting poor model fit, and differing interval widths suggest that across class variability may not be fully accounted for.

Step 7: We assessed for clinical characterisation and plausibility using four approaches: i) assessing the clinical meaningfulness of the trajectory patterns, aiming to include classes with at least 1% capture of the population; ii) assessing the clinical plausibility of the trajectory classes; iii) tabulation of characteristics by latent classes and description of patterns compared with conventional categorisations; and iv) concordance of class characteristics with those for other well-established variables, arguing that low concordance indicates that the new latent classes offer information above and beyond that from the conventional categorisation.

Step 8: We conducted sensitivity analyses, in this example, with individuals with at least two and three BMI values, as LCTMs are flexible enough to deal with different observation times between participants.

Statistical algorithms

All R and SAS codes used to implement these tools are available via the authors and can be downloaded from *www.github.com/hlennon/LCTMtools.*

RESULTS

Number of classes

From the preliminary working model of a quadratic random-effects model, model F (proportional covariance structure), we derived BICs for up to seven classes: three of the class models failed to converge in men and women. **Table 2** reports that the lowest BIC was obtained with five classes in men and women – confirming our initial working model. The

For peer review only - http://bmjopen.bmj.com/site/about/guidelines.xhtml

proportions by class in men were 68.1%, 25.0%, 3.8%, 2.7%, and 0.4%; and in women, were 32.6%, 41.1%, 21.1%, 3.5% and 1.7%. For model G (our second choice model structure), the lowest BICs were noted for five classes in men and women, and without problems of failure to converge (Table S2).

Assessment for model structures

With the number of classes now selected as five, we tested the seven model structures – A to G. **Table 3** reports that the lowest BIC was for model F in men and women, justifying the selection of model F in the preliminary working phase. The class sizes varied between models, with Class I ranging from 41% to 68% in men and from 32% to 95% in women. The APPA for each class in model F was 0.81, 0.74, 0.87, 0.83, and 0.74 in men and 0.74, 0.79, 0.80, 0.83 and 0.84 in women, indicating a good discrimination of trajectory. The classes were well differentiated with the relative Entropy, E_K values ranging from 0.59 to 0.81 in men and 0.66 to 0.83 in women.

There was moderately good concordance (unweighted and weighted) between the unstructured variance models G with model F in men (κ: 0.57) and women (κ: 0.65) (Tables S4 and S5), but poorer concordance between the final preferred models and fixed-effects models in men, indicating that the models are informing different patterns.

Graphical presentation

We plotted the mean trajectories for model A, B, C, D, F and G in men and women **(Figure 1)** illustrating the increased complexity from model A to G. As alternatives, we plotted separately mean trajectories with 95% predictive intervals for each class, in model F (Figure S2), which displays the estimated random variation within each of the classes with time, noting that variation was greater with the more 'complex' classes (classes IV and V compared with classes I, II and III). Spaghetti plots of individual level data illustrated that the timing and size of BMI increases characterise the classes – for example, sharp increases in BMI in early adulthood in Class III but later in adulthood for Class IV (Figure S3).

For peer review only - http://bmjopen.bmj.com/site/about/guidelines.xhtml

Additional tools of suitability of fit

The DoS_k values ranged from 0.10 to 0.36 and 0 to 0.34, in men and women, respectively **(Table 3)**. The covariances were high and in the positive direction and therefore models with non-parallel mean trajectories lead to higher separation.

We plotted the local standard deviation of the residuals with time and found that these were broadly homogeneous, i.e., there were few parallel boundaries **(Figure 2)**. The local residuals for the rapidly obese groups in both genders are the exceptions to parallel lines, which might reflect comorbidities in this group and smaller numbers.

Clinical assessment

Having established the preferred model, model F with five classes, in both genders, we assigned descriptive labels to each respective class as follows **(Table 4)**: stable normal weight; normal weight to overweight; normal weight to obese; overweight to obese; and rapid early obesity. We noted that the proportion in the rapid early obesity (Class V) was less than 1% in men. However, overall, the proportion for Class V for men and women combined was nearly 1% - we retained this class in our preferred model as we judged it to be clinically meaningful as follows. In men and women, there was a rapid increase in obesity from early to middle adulthood, then apparent severe weight loss. We rationalised that this was clinically plausible, as it could be explained either by intentional (e.g. bariatric surgery) or non-intentional weight loss (e.g. reverse causality from development of disease).

We then tabulated the baseline characteristics according to the five classes for model F in men and women, and noted patterns across the classes **(Table 4** – and fully expanded in Tables S5 and S6). Thus, for example, for current smoking status, there were little differences in patterns by class in men: 10%, 8%, 9%, 10%, and 15%; and in women, 16%, 12%, 12%, 13% and 11%. This contrasts with BMI categories—men (12%, 8%, 8%, 7% and 7%) and women (16%, 12%, 10%, 8%, and 7%) (Tables S7 and S8)—suggesting that the

For peer review only - http://bmjopen.bmj.com/site/about/guidelines.xhtml

clinical characteristics derived from the LCTM differed from those derived from conventional categorisation approaches.

Finally, we noted very poor concordance between the preferred final model and conventional BMI categorisation in men (κ: 0.18) and women (κ: 0.52) **(Table 5)**, indicating that the classifications are informing very different patterns in men.

Sensitivity analyses

We tested the preferred model using a larger sample of individuals with at least three measures, and found no material differences between these models, in men and women, and the main model (Figure S4).

DISCUSSION

Main findings

We propose a standardised framework for model selection in the context of latent class trajectory modelling; thus, we argue for random- rather than fixed-effects models. We show that different model structures result in very different classes and hence phenotypes, and we propose pre-specified criteria for model selection and reporting of a ‘core’ model can facilitate generalisability. Compared with ‘one-off’ covariate categorisation, latent classes offer additional phenotypic information; and offer a disease prevention opportunity through identifying classes of exposure prior to disease diagnosis.

Context of other literature

Criticisms of the use of LCTM models previously have been that the uncertainty in class membership is not discussed. However, entropy-based measures, such as E and E_k, which have been developed to quantify uncertainty (19) can be used with LCTMs. This issue can be further overcome if these tools are made publicly available to non-specialists and can be used in an easy manner.

For peer review only - http://bmjopen.bmj.com/site/about/guidelines.xhtml

We tested a variety of assumptions and demonstrated that small changes in random-effects structures can have dramatic effects on the shapes of the trajectories derived and, therefore, it is vital that when conducting a LCTM study, analysts are aware of the differences between fixed-effects and random-effects models and are transparent when reporting their model selection procedures. For example, Viristen et al. (27) specified a random slope but didn't explain the reason for the choice of this assumption. Since the publication of the GRoLTS Guidelines (Guidelines on Reporting of Latent Trajectory Studies) earlier this year, we hope this will become common practice for future LCTM studies.

Strengths and weaknesses

The study has strengths. First, the considered and strategic workflow to optimise identification and application of latent classes provides for a more robust and transparent application of these models in epidemiology. Second, the results presented are based on modelling data from a large well-characterised US cohort, therefore allowing the derivation of numerically meaningful subpopulations (i.e. classes) with distinct phenotypes. We have demonstrated that these subpopulations are markedly different to considering only one measure at baseline and more likely linked to what the clinician sees in practise as the BMI history has been taken into account. Third, we constructed several gender-specific models with differing number of classes and a range of distributional assumptions; we extensively explored different model selection and adequacy tools and described extensions to other tools, to supplement the assessment of suitable model fit. Fourth, we have made the code freely and publicly available to aid ease of interpretation of these measures to allow LCTM analysts to consider the model specification more carefully.

There are several study weaknesses. First, directly capturing records of other common disease risk factors, such as smoking, over time (i.e., in a time-varying manner) is difficult with LCTMs as we are currently considering trajectories of one risk factor at a time. Second, the two-stage approach requires a methodological approach to ensure model selection is not unconsciously 'cherry-picked'. Although specialist knowledge of the data aids

For peer review only - http://bmjopen.bmj.com/site/about/guidelines.xhtml

model building, a clear understanding of the difference between fixed- and random-effects models is required by the analyst. Third, the Kappa value is not optimal criteria for LCTM comparisons. It is typically used to for inter-rater agreement where the labels are the same across the classes, e.g, BMI categories – as opposed to this unsupervised learning approach where the classes are defined by the algorithm. To draw a fair comparison between models and the matching of the class labels across the models, we computed κ for all 120 possible combinations and selected the optimal κ to report. Fourth, while the multiple diagnostics tests we have implemented can be useful, it is likely that some measures favour one model, while other measures favour another. Therefore it is important that the analyst is aware that model selection of any LCTM must be guided by model interpretation, as well as likelihood-based model-fit criteria (17). Nagin (6) summarises this well by saying "*In the end, the objective of the model selection is not the maximisation of some statistic of model fit. Rather it is to summarise the distinctive features of the data in as parsimonious a fashion as possible"*.

Clinical implications and future research

Variations of model A (fixed-effects) have been commonly used in the clinical literature (8-11), which assume no within-class variability when deriving latent classes. Interpretation in this setting is that variation from the mean trajectory is random, i.e., the correlation between measurements for the same individual is explained by latent class membership. In the context of any repeated measures in the general population, this assumption might not be valid (13). Saunders (28) argued in support of full random-effects models (i.e. models F and G), calling upon Moffitt's theory from criminology, which recognises that "there are distinct developmental clusters of trajectories of anti-social behaviour that are the result of divergent aetiologies" – in other words, it is unlikely that latent classes start from a similar baseline.

A larger number of classes can become difficult to interpret, and five BMI trajectory classes is supported by other studies in the literature (8-11). Determining the number of latent classes seems to be the focus of many trajectory studies and method papers. Whilst

For peer review only - http://bmjopen.bmj.com/site/about/guidelines.xhtml

this is an important question, we have demonstrated the importance of determining an appropriate model structure to the problem and provided a framework and tools to facilitate generalisability in future LCTM studies. With BMI measurements varying more between individuals than within individuals, BMI trajectory studies need to be implemented and interpreted carefully. Our standardised framework provides a means to robustly model risk factor trajectories.

For peer review only

Role of the funding source

The funders of the study had no role in study design, data collection, data analysis, data interpretation, or writing of the report.

Conflict of Interest

AGR has received lecture honoraria from Merck Serona and Janssen-Cilag, and independent research funding from Novo Nordisk. All other authors have no conflicts of interest to declare.

Contributions

HL, MS, MC and AGR conceptualised the paper. HL, MS and SK designed the statistical approaches; HL performed the modelling. AC and ML facilitated data access and interpretation of the AARP data. All authors contributed to data interpretation; IB and AGR put modelling into clinical context.

Acknowledgements

We acknowledge the generous funding from Cancer Research UK National Awareness and Early Detection Initiative (NAEDI).

Data sharing statement

All statistical codes used in this paper are available via the authors and can be downloaded from www.github.com/hlennon/LCTMtools. Supplementary materials are available from the Dryad repository, DOI: [include DOI here].

For peer review only

REFERENCES

1. Wormser D, Kaptoge S, Di Angelantonio E, Wood AM, Pennells L, Thompson A, et al. Separate and combined associations of body-mass index and abdominal adiposity with cardiovascular disease: collaborative analysis of 58 prospective studies. Lancet 2011;377(9771):1085-95.

2. Vazquez G, Duval S, Jacobs DR, Jr., Silventoinen K. Comparison of body mass index, waist circumference, and waist/hip ratio in predicting incident diabetes: a meta-analysis. Epidemiol Rev 2007;29:115-28.

3. Lauby-Secretan B, Scoccianti C, Loomis D, Grosse Y, Bianchini F, Straif K. Body Fatness and Cancer--Viewpoint of the IARC Working Group. Colditz G, Anderson AS, Herbert RA, Kaaks R, Thompson HJ, Baker JL, Breda J, Byers T, Cleary MP, Di Cesare M, Gapstur SM, Gunter M, Hursting SD, Leitzmann M, Ligibel J, Renehan A, Romieu I, Shimokawa I, Ulrich CM, Wade K, Weiderpass E. N Engl J Med 2016;375(8):794-8.

4. Global BMI Mortality Collaboration. Body-mass index and all-cause mortality: individual-participant-data meta-analysis of 239 prospective studies in four continents. Lancet 2016.

5. Peto J. That the effects of smoking should be measured in pack-years: misconceptions 4. Br J Cancer 2012;107(3):406-7.

6. Nagin D. Group-based modeling of development: Harvard University Press; 2005.

7. Stevenson J, Thompson MJ, Sonuga-Barke E. Mental health of preschool children and their mothers in a mixed urban/rural population. III. Latent variable models. Br J Psychiatry 1996;168(1):26-32.

8. Song M, Hu FB, Wu K, Must A, Chan AT, Willett WC, et al. Trajectory of body shape in early and middle life and all cause and cause specific mortality: results from two prospective US cohort studies. BMJ 2016;353:i2195.

9. Song M, Willett WC, Hu FB, Spiegelman D, Must A, Wu K, et al. Trajectory of body shape across the lifespan and cancer risk. Int J Cancer 2016;138(10):2383-95.

10. Petrick JL, Kelly SP, Liao LM, Freedman ND, Graubard BI, Cook MB. Body weight trajectories and risk of oesophageal and gastric cardia adenocarcinomas: a pooled analysis of NIH-AARP and PLCO Studies. Br J Cancer 2017;116(7):951-959.

11. Kelly SP, Graubard BI, Andreotti G, Younes N, Cleary SD, Cook MB. Prediagnostic Body Mass Index Trajectories in Relation to Prostate Cancer Incidence and Mortality in the PLCO Cancer Screening Trial. J Natl Cancer Inst 2017;109(3).

12. Qian T, Masino AJ. Latent Patient Cluster Discovery for Robust Future Forecasting and New-Patient Generalization. PLoS One 2016;11(9):e0162812.

13. Gilthorpe MS, Dahly DL, Tu YK, Kubzansky LD, Goodman E. Challenges in modelling the random structure correctly in growth mixture models and the impact this has on model mixtures. J Dev Orig Health Dis 2014;5(3):197-205.

14. van de Schoot R, Sijbrandij M, Winter SD, Depaoli S, Vermunt JK. The GRoLTS-Checklist: Guidelines for Reporting on Latent Trajectory Studies. Structural Equation Modeling: A Multidisciplinary Journal 2017;24:451-467.

For peer review only

For peer review only - http://bmjopen.bmj.com/site/about/guidelines.xhtml

15. Schatzkin A, Subar AF, Thompson FE, Harlan LC, Tangrea J, Hollenbeck AR, et al. Design and serendipity in establishing a large cohort with wide dietary intake distributions : the National Institutes of Health-American Association of Retired Persons Diet and Health Study. Am J Epidemiol 2001;154(12):1119-25.

16. Renehan AG, Flood A, Adams KF, Olden M, Hollenbeck AR, Cross AJ, et al. Body mass index at different adult ages, weight change, and colorectal cancer risk in the National Institutes of Health-AARP Cohort. American journal of epidemiology 2012;176(12):1130-1140.

17. Tu Y-K, Gilthorpe MS. Statistical thinking in epidemiology: CRC Press; 2011.

18. Proust-Lima C, Philipps V, Diakite A, Liquet B. Package 'lcmm' Title Extended Mixed Models Using Latent Classes and Latent Processes. In; 2016.

19. Shannon CE. Prediction and Entropy of Printed English. Bell System Technical Journal 1951;30(1):50-64.

20. Jones BL, Nagin DS. Advances in group-based trajectory modeling and an SAS procedure for estimating them. Sociological Methods & Research 2007;35(542-571).

21. Verbeke G, Molenberghs G. Linear Mixed Models for Longitudinal Data. 2000.

22. Klijn SL, Weijenberg MP, Lemmens P, van den Brandt PA, Lima Passos V. Introducing the fit-criteria assessment plot - A visualisation tool to assist class enumeration in group-based trajectory modelling. Stat Methods Med Res 2015.

23. Lubke G, Neale MC. Distinguishing between latent classes and continuous factors: Resolution by maximum likelihood? Multivariate Behavioral Research 2006;41(4):499-532.

24. Peugh J, Fan X. How Well Does Growth Mixture Modeling Identify Heterogeneous Growth Trajectories? A Simulation Study Examining GMM's Performance Characteristics. Structural Equation Modeling: A Multidisciplinary Journal 2012;19(2):204-226.

25. Elsensohn M-H, Klich A, Ecochard R, Bastard M, Genolini C, Etard J-F, et al. A graphical method to assess distribution assumption in group-based trajectory models. Statistical methods in medical research 2016;25(2):968-982.

26. Klijn SL, Weijenberg MP, Lemmens P, Brandt PAvd, Passos VL. Introducing the fit-criteria assessment plot – A visualisation tool to assist class enumeration in group-based trajectory modelling. Statistical Methods in Medical Research 2015;0(0):0962280215598665.

27. Vistisen D, Witte DR, Tabak AG, Herder C, Brunner EJ, Kivimaki M, et al. Patterns of obesity development before the diagnosis of type 2 diabetes: the Whitehall II cohort study. PLoS Med 2014;11(2):e1001602.

28. Saunders JM. Understanding Random Effects in Group-Based Trajectory Modeling: An Application of Moffitt's Developmental Taxonomy. J Drug Issues 2010;40(1):195-220.

For peer review only

For peer review only - http://bmjopen.bmj.com/site/about/guidelines.xhtml

Table 1 Framework of eight steps to construct a latent class trajectory model

Step	Step description	Criteria for selection
1	Scoping model provisionally selecting a plausible number of classes based on available literature and the structure based on plausible clinical patterns	Examine linearity of the shape of standardised residual plots for each of the classes in a model with no random-effects
2	Detailed refinement of the model from step 1 to confirm the optimal number of classes, typically testing k = 1 to 6 classes	Lowest Bayesian Information Criteria (BIC) value
3	Detailed refinement to confirm the optimal model structure from fixed- through to unrestricted random-effects of the model using the preferred k derived in step 2	Lowest BIC value
4	Model adequacy assessment including Posterior Probability of Assignments (APPA); Odds of Correct Classification (OCC); and entropy	i) APPA: average of maximum probabilities should be greater than 70% for all classes<br>ii) OCC values greater than 5.0<br>iii) Entropy values greater than 0.5
5	Graphical presentation	i) Plot mean trajectories across time for each class in a single graph<br>ii) Plot mean trajectories with 95% predictive intervals for each class (one class per graph)<br>iii) Plot individual class 'spaghetti plots' across time for a random sample
6	Additional tools to assess discrimination including Degrees of Separation and Elsensohn's envelope of residuals	i) DoS greater than zero<br>ii) Envelope of residuals is assessed in plots by observing clear separations between classes
7	Assessment for clinical characterisation and plausibility	i) Tabulation of characteristics by latent classes. Are the trajectory patterns clinically meaningful? Perhaps, consider classes with a minimum percentage of the population.<br>ii) Are the trajectory patterns clinically plausible?<br>iii) Concordance of class characteristics with those for other well-established variables
8	Sensitivity analyses – for example, testing models without complete data at all time points	General assessment of patterns of trajectories compared with main model

For peer review only - http://bmjopen.bmj.com/site/about/guidelines.xhtml

Table 2 Number of classes (*k* = 1 to 7) using random-effects quadratic structure model F (proportional covariance structure) by gender

Model	*k*	Number of parameters	BIC	Proportions per class %						
				Class I	Class II	Class III	Class IV	Class V	Class VI	Class VII
**MEN**										
**Model F**	1	10	∗	100						
	2	15	3324009	83	17					
	3	20	3310908	62	32	3				
	4	25	3324128	100	0	0	0			
	5	30	**3301301**	68	25	4	3	0.4		
	6	35	∗							
	7	40	∗							
**WOMEN**										
**Model F**	1	10	∗	100						
	2	15	2195386	86	14					
	3	20	2179080	58	34	8				
	4	25	2179137	100	0	0	0			
	5	30	**2169791**	41	32	21	4	2		
	6	35	∗							
	7	40	∗							

∗ Models failed to converge
BIC: Bayesian Information Criteria
Results from random-effects quadratic structure Model G (unrestricted) by gender are shown in supplemental material.

For peer review only

Table 3 Model adequacy assessments of latent trajectory class models based on different assumptions for *k* = 5 classes, by gender in the NIH-AARP cohort

Model	Description	BIC	Proportion per class %	Average Posterior Probability Assignment (APPA)	Relative Entropy E_k	Degree of Separation DoS_k
**Men**						
A	Homoscedastic	3476322	51: 22: 21: 6: 1	83: 84: 85: 91: 95	0.78	0.10
B	Heteroscedastic	3511564	41: 26: 20: 7: 6	85: 82: 84: 89: 86	-	0.15
C	Random intercept	3364856	68: 24: 4: 3: 2	90: 82: 89: 85: 82	0.81	0.34
D	Random slope	3325463	63: 19: 13: 3: 4	72: 70: 74: 83: 83	0.59	0.26
E	Random quadratic - Equal	*				
F	Random quadratic - Proportional	**3301301**	68: 25: 4: 3: 0.4	81: 74: 87: 83: 74	0.68	0.36
G	Random quadratic unrestricted	3320005	56: 30: 8: 5: 0.4	74: 70: 80: 79: 88	0.63	0.33
**Women**						
A	Homoscedastic	2289509	45: 36: 13: 4: 1	90: 84: 88: 93: 96	0.83	0.14
B	Heteroscedastic	2238364	32: 30: 15: 15: 8	87: 83: 85: 89: 91	-	0.05
C	Random intercept	2240681	52: 35: 9: 2: 2	89: 82: 86: 89: 91	0.79	0.21
D	Random slope	2193155	79: 12: 5: 2: 2	91: 80: 79: 86: 91	0.82	0.34
E	Random quadratic - Equal	2188224	95: 5: 0: 0: 0	49: 88: 0: 0: 0	0.26	0.05
F	Random quadratic - Proportional	**2169793**	41: 33: 21: 3: 2	74: 79: 80: 83:84	0.66	0.09
G	Random quadratic - unrestricted	2187707	67: 23: 6: 3: 1	84: 77: 84:82: 87	0.73	0.34

*Failed to converge

For peer review only

Table 4 Latent class characteristics of 177,453† men and 111,503† women in the NIH-AARP cohort

	Model F (preferred model)				
	**Class I** Stable normal weight	**Class II** Normal weight to overweight	**Class III** Normal weight to obese	**Class IV** Overweight to obese	**Class V** Rapid increase to obese
**MEN**					
Number of participants, N	120 867 (68.1%)	44 383 (25.0%)	6 723 (3.8%)	4 764 (2.7%)	718 (0.4%)
Mean (sd) entry age, years	62.88 (0.01)	61.59 (0.03)	62.03 (0.06)	59.71 (0.07)	57.73 (0.18)
Mean (sd) current (at baseline) BMI, kg/m^2	25.39 (0.01)	30.84 (0.02)	29.07 (0.05)	35.14 (0.12)	30.51 (0.28)
Mean (sd) waist circumference, cm	94.38 (0.02)	106.23 (0.05)	102.73 (0.13)	115.24 (0.28)	105.91 (0.64)
Smoking, %					
Current	11 823 (10%)	3 761 (8%)	638 (9%)	460 (10%)	106 (15%)
Former	67 948 (56%)	26 898 (61%)	3 809 (57%)	3 002 (63%)	370 (52%)
Never	37 149 (31%)	12 130 (27%)	2 012 (30%)	1 110 (23%)	218 (30%)
Missing	3 946 (3%)	1 594 (4%)	264 (4%)	191 (4%)	24 (3%)
Mean (sd) alcohol consumption per day, g	18.69 (0.13)	17.45 (0.21)	15.48 (0.56)	15.57 (0.69)	10.22 (1.14)
**WOMEN**					
Number of participants, N	36 311 (32.6%)	45 832 (41.1%)	23 544 (21.1%)	3 898 (3.5%)	1 918 (1.7%)
Mean (sd) entry age, years	62.48 (0.03)	62.23 (0.02)	61.07 (0.03)	59.61 (0.09)	59.69 (0.12)
Mean (sd) current (at baseline) BMI, kg/m^2	21.97 (0.01)	26.66 (0.01)	32.4 (0.03)	37.47 (0.15)	34.4 (0.19)
Mean (sd) waist circumference, cm	76.19 (0.04)	86.66 (0.05)	97.82 (0.09)	106.29 (0.32)	101.96 (0.41)
Smoking, %					
Current	5803 (16%)	5660 (12%)	2733 (12%)	495 (13%)	214 (11%)
Former	14173 (39%)	18557 (40%)	9748 (41%)	1764 (45%)	731 (38%)
Never	15313 (42%)	20290 (44%)	10417 (44%)	1507 (39%)	909 (47%)
Missing	1022 (3%)	1325 (3%)	646 (3%)	132 (3%)	64 (3%)
Mean (sd) alcohol consumption per day, g	8.07 (0.1)	6.26 (0.08)	4.47 (0.11)	3.92 (0.29)	3.4 (0.48)
Hormone Therapy Use, %					
Ever	13989 (39%)	20206 (44%)	12215 (52%)	2241 (57%)	1092 (57%)
Never	22322 (61%)	25626 (56%)	11329 (48%)	1657 (43%)	826 (43%)

sd: standard deviation; †Exclusions include 35 women and 2 men with biologically implausible cancers from classes in proportions (2,16,12,0,5) and (1,0,0,1,0) respectively.

For peer review only - http://bmjopen.bmj.com/site/about/guidelines.xhtml

For peer review only

For peer review only - http://bmjopen.bmj.com/site/about/guidelines.xhtml

Table 5 Concurrence between BMI categories and classes in model F from the NIH-AARP cohort

Latent class	N	BMI categories (kg/m^2) < 18.5	18.5 to 24.9	25.0 to 29.9	30.0 to 34.9	>35.0
				**Men**		
	177453	875	53466	86967	28234	7911
I	120866	312	50171	68646	1736	1
II	44383	319	1860	14097	23472	4635
III	6723	47	859	3335	1943	539
IV	4763	172	437	679	917	2558
V	718	25	139	210	166	178
				**Cohen κ_ω = 0.182 (0.181,0.183)**		
				**Women**		
	111503	1327	48273	36025	16245	9633
I	36311	1215	33371	1725	0	0
II	45832	3	12584	28636	4607	2
III	23544	83	1683	4777	10587	6414
IV	3898	14	382	519	614	2369
V	1918	12	253	368	437	848
				**Cohen κ_ω= 0.52 (0.51,0.53)**		

BMI: body mass index; κ_ω weighted

For peer review only

FIGURE LEGENDS

Figure 1 BMI mean trajectories by men (left) and women (right) for models A-G. Colours are used to discriminate classes within each plot, but should not be used for direct comparisons across plots.

Figure 2 Illustration of the local (Elsensohn) residual envelope plots (shown here for model B)

For peer review only

For peer review only - http://bmjopen.bmj.com/site/about/guidelines.xhtml

Model A (Fixed effect - Homoscedastic)

Model B (Fixed effect - Heteroscedastic)

Model C (Random intercept)

Model D (Random slope)

Model F (Random quadratic - proportional)

Model G (Random quadratic - unrestricted)

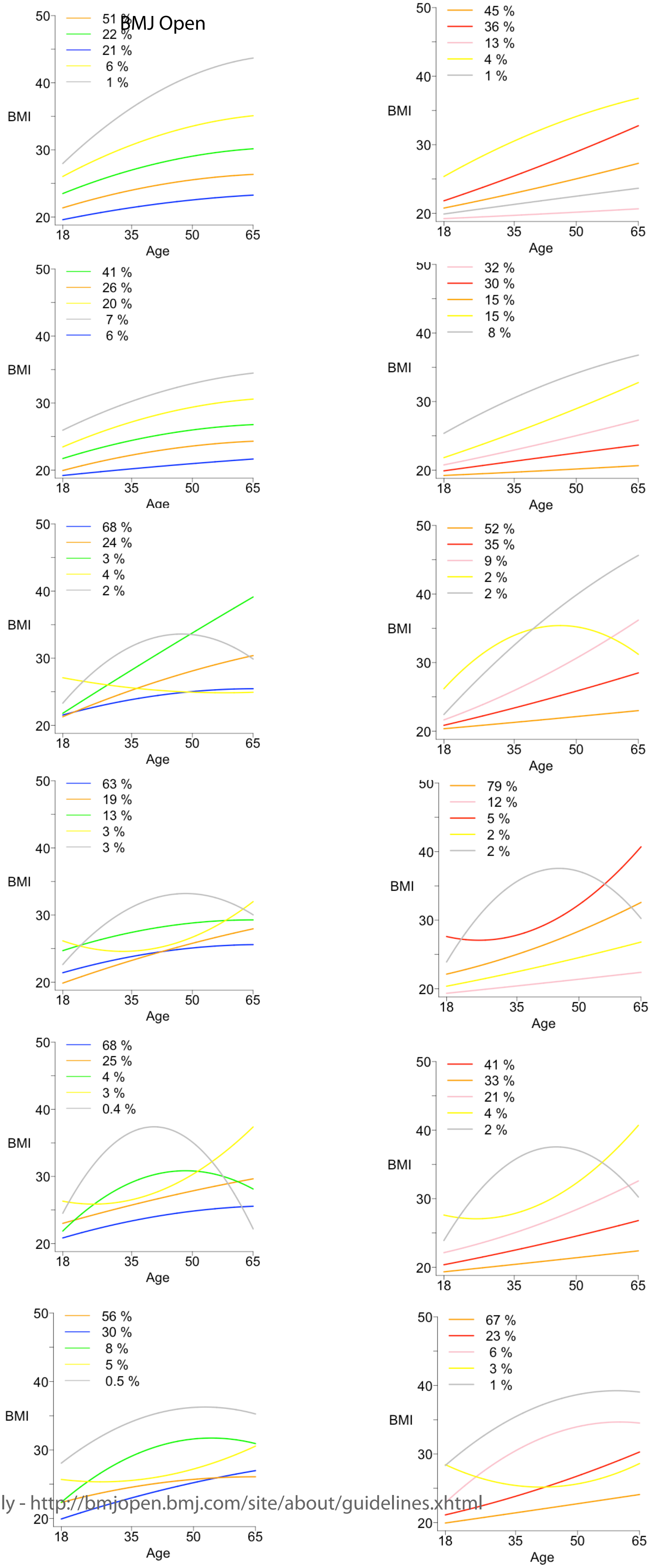

Figure 1

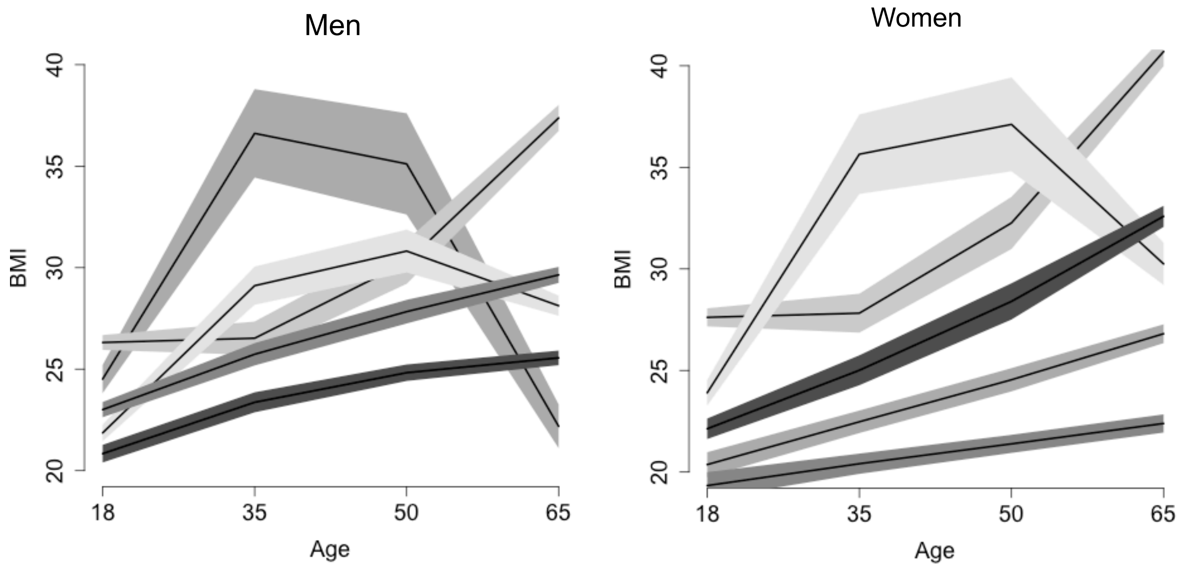

Figure 2

SUPPLEMENTAL MATERIAL

A framework to construct and interpret latent class trajectory modelling

Hannah Lennon[1,3] Scott Kelly,[2] Matthew Sperrin,[3] Iain Buchan,[3] Amanda J Cross,[4] Michael Leitzmann,[5] Michael B Cook,[2] Andrew G Renehan[1,3]

For peer review only

Table S1 Different models with increasing complexity, associated software and referenced use in literature

Model	Description	Interpretation	Software *Command*	References
A	Fixed effects homoscedastic (common residual variance across classes)	No random effects – with the interpretation that any deviation of an individuals trajectory from its mean class trajectory is due to random error only	SAS traj *PROC TRAJ*	(1-5)
B	Fixed effects heteroscedastic (class-specific residual variances)	The same interpretation as Model A with random errors that can be larger and smaller in different classes.	R mmlcr *mmlcr*	(6)
C	Random intercept	The interpretation is allowing individuals to vary in initial weight but each class member is assumed to follow the same shape and magnitude of the mean trajectory	SAS traj *PROC TRAJ*	(7)
D	Random slope	Allowing individuals to vary in initial weight and slope of the mean trajectory but same curvature as trajectory	SAS traj *PROC TRAJ*	(7)
E	Random quadratic – Common variance structure across classes	Additional freedom of allowing individuals to vary within classes by initial weight, shape and magnitude, however each class is assumed to have the same amount of variability	R lcmm *hlme/lcmm*	
F	Random quadratic – Proportionality constraint to allow variance structures to vary across classes	Increasing flexibility of model E as variance structures are allowed to differ up to a multiplicative factor to allow some classes to have larger or smaller within-class variances. This model is can be thought of more parsimonious version of model G from (reducing the number of variance-covariance parameters to be estimated from 6xK parameters to 6+(K-1) parameters.	R lcmm *hlme/lcmm*	
G	Random quadratic – Class-specific variance structure (unstructured)	The most flexible model in which each class has its own separate random quadratic variance structure to describe its own within-class variability. Statistically this permits the variance and covariance of the intercept, slope and quadratic term to vary freely across all classes.	SAS traj *PROC TRAJ*	

[1]The SAS traj package has been converted for Stata users as the traj command in Stata (Collage Station, TX, USA).

For peer review only - http://bmjopen.bmj.com/site/about/guidelines.xhtml

More detailed description of model structure

For N individuals, the latent class trajectory model we consider is given by:

$$BMI_{itk} = \beta_0^k + \beta_1^k AGE_t + \beta_2^k AGE_t^2 + b_0^k + b_1^k AGE_t + b_2^k AGE_t^2 + \epsilon_t, \qquad \text{(Equation 1)}$$

where BMI_{itk} is the BMI of individual $i = 1, \dots, N$, at time $t = 1, \dots, T$ in class $k = 1, \dots, K$. The random effect b^k is class-specific and follows a multivariate Normal distribution with zero mean and a 3×3 variance-covariance matrix B. For the residual error term ϵ_t, the usual assumption hold, ϵ_t is normally distributed with zero mean and variance σ^2. The probability of an individual belonging to class k is described by a multinomial distribution, i.e.,

$$Pr(individual\ i\ belonging\ to\ class\ k) = \frac{e^{\pi_k}}{\sum_{k=1}^{K} e^{\pi_k}} = \frac{e^{\pi_k}}{e^{\pi_1} + \cdots + e^{\pi_K}}, \qquad \text{(Equation 2)}$$

such that π_k are parameters to be estimated in the model. To select the number of latent classes, we assume a working model (Equation 1) for the random effect structure and the criterion used to select the number of classes was the lowest Bayesian Information Criteria (BIC).

More detailed description of model adequacy assessment

Common tools for model adequacy assessment checking are described below in Table S2. Here we give more details of the two extensions of these tools; degree of separation and Elsensohn's residuals (to random effects).

Degrees of separation

A model's ability to detect classes accurately is affected by the degree of separation between latent trajectory curves (8, 9). To describe the separation of latent growth curves, we used the multivariate Mahalanobis distance with the multivariate Mahalanobis distance (D) units defined as:

$$D_{ij} = (\mu_i - \mu_j)^T \Sigma^{-1} (\mu_i - \mu_j), \qquad i, j = 1, \dots K,$$

For peer review only - http://bmjopen.bmj.com/site/about/guidelines.xhtml

where μ_i is a $T\times 1$ vector of mean values for class i, and Σ^{-1} is the inverse of a $T\times T$ matrix of sample covariances of at times $t = 1, \dots, T$. The larger the difference, the larger the separation between curves. Peugh and Fan (9) argue that it is reasonable to expect that it is easier to identify heterogeneous latent growth trajectories when the statistical separation distance among the subpopulations is larger than when the separation distance among the latent subpopulations is much smaller.

To give an overall measure of separation for each model, we propose a weighted sum of multivariate Mahalanobis distance matrix with weights being the estimated class proportions, $\hat{\pi}_i$.. Then the degree of separation DoS_K is defined as

$$DoS_K = \sum_{i=1}^{K}\sum_{j=1}^{i} \hat{\pi}_i \hat{\pi}_j D_{ij}, \quad i,j = 1,2,\dots,K.$$

Larger values of DoS_K indicate the mean trajectories are well separated while DoS_K is zero in the special case when all mean trajectories are identical. If the DoS_K value is small, then you may wish to consider a model with fewer classes.

Elsensohn's envelope of residuals

To check the model assumption in fixed effect latent class models, Elsenhohn et al. (6) suggest plotting the local standard deviations of the residuals to check the appropriateness of the model. With the assumption that the residuals are homogeneous over time. To check the appropriateness of each of our model assumptions, we extended their method to include random effects in the models. We compute the local standard deviations of the residuals using the following steps:

1) Compute the observed residuals r_{itk} for each subject i, at time t given the individual is in class k

Equation 1: $r_{itk} = y_{it} - \hat{y}_{itk} = y_{it} - x_i^T \beta^k - z_i^T b^k,$

For peer review only - http://bmjopen.bmj.com/site/about/guidelines.xhtml

where y_{itk} is the observed value for individual i at time t in class k and $\hat{y}_{itk}$ is the fitted value of BMI from our fitted model, here the random effect model. 2) Compute the class- and time-specific weighted local variance of the residuals, $Var_w[r_{itk}]$, with weights being p_{ik}, the posterior probabilities of individual i belonging to group k. #) Plot the upper and lower boundaries for the local standard deviations of the residuals

Equation 2: $b_{itk} = \mu_{tk} \pm \sqrt{Var_w[r_{itk}]}$,

$where\ \mu_{tk}$ is the mean value of class k at time t. 4) Plot the boundaries b_{itk} onto the mean trajectory plots.

The shape of the local standard deviation of the residuals indicates the appropriateness of the model assumptions; where non-parallel boundaries indicate heteroscedasticity of residuals suggesting poor model fit, and differing interval widths suggest across group variability may not be fully accounted for. We believe this complements the others metrics well. If the boundaries suggest a poor fit, you may consider a more complex random effect structure to be more suitable, for example, a higher order polynomial.

For peer review only

Figure S1 Standardised residual plots of fixed-effect model (model A) for each class justifying the selection of a random-effect structure in the scoping model

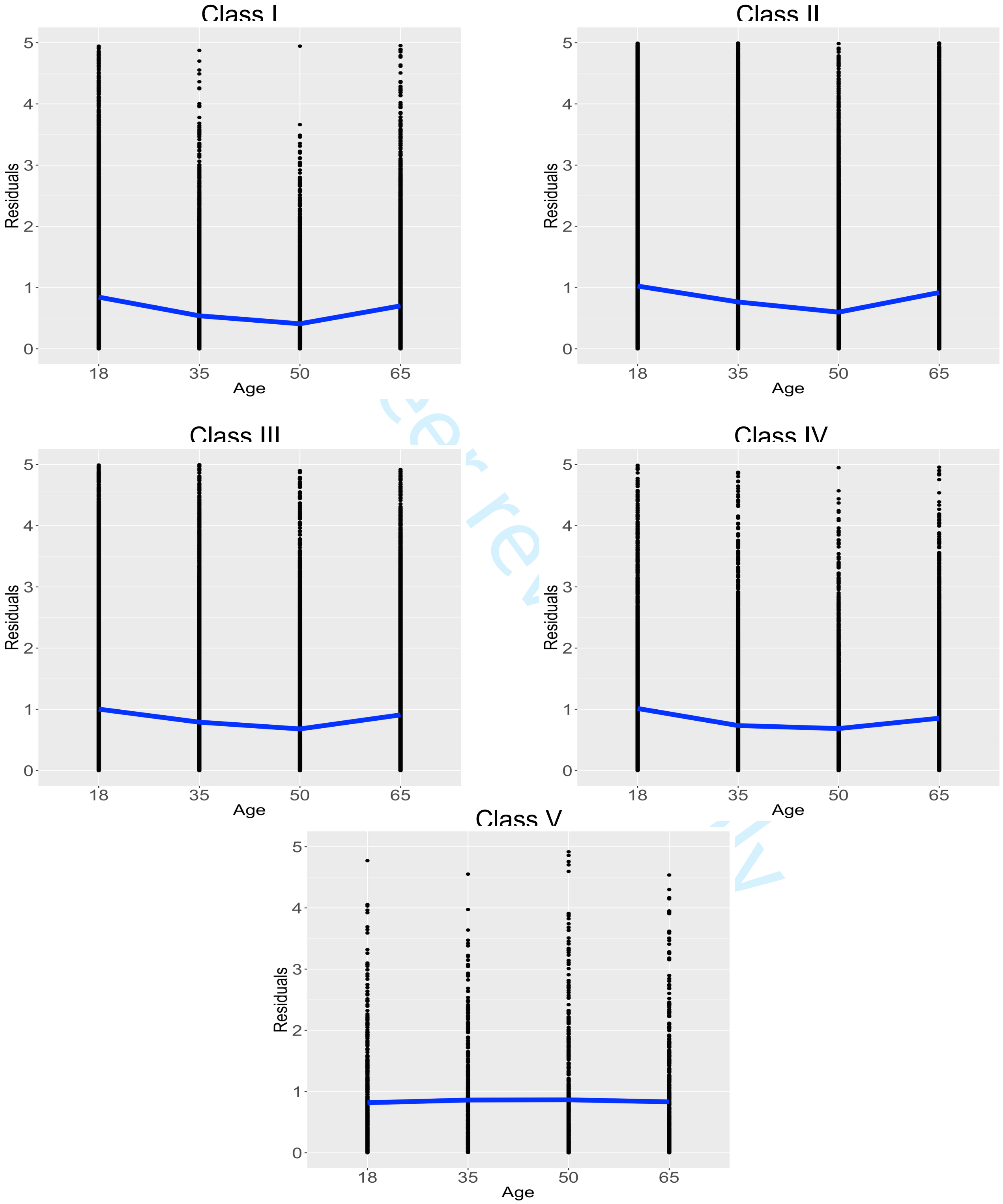

For peer review only - http://bmjopen.bmj.com/site/about/guidelines.xhtml

Table S2 Number of classes (k = 1 to 7) using random quadratic effect models: model G (unrestricted) by gender, NIH-AARP cohort

Model	k	Num of parameters	BIC	Proportions per class (%)						
				Class I	Class II	Class III	Class IV	Class V	Class VI	Class VII
**MEN**										
**Model G**	2	14	3361853	94	6					
	3	21	3341066	87	7	7				
	4	28	3329426	52	37	7	5			
	5	35	**3320005**	51	33	9	6	1		
	6	42	3315240	45	31	12	9	2	1	
	7	48	3311326	51	19	13	7	5	3	0.4
**WOMEN**										
**Model G**	2	14	2219825	89	11					
	3	21	2203726	61	34	0.5				
	4	28	2194293	61	32	5	2			
	5	35	**2187707**	60	29	7	3	1		
	6	42	2184412	63	19	10	4	1	0.5	
	7	48	*							

* Models failed to converge

For peer review only

Table S3 Common model selection tools for latent class trajectory models

Tool	Criteria for a good model	Description
Likelihood based measures	Lowest BIC, AIC,	These likelihood based model selection tools include: Bayesian Information Criteria (BIC), Akaike Information Criteria (AIC), Likelihood Ratio Tests (LRT), Lo-Mendel-Rubin (LMT-LRT), Bootstrap-LRT. (See Peugh and Fan, 2015 for discussion) (10)
APPA	APPA > 70% for each class	The average Posterior Probability Assignment, $\bar{p}_k$, looks at whether individuals are assigned with high probability and the overall average probability of assignment to each class.
Odds of correct classification	OCC > 5.0 for each class	The odds of correct classification (OCC) is the ratio of the odds of a correct classification into each group on the basis of the maximum probability classification rule and the estimated class membership proportions, $\widehat{\pi_k}$, $$OCC_k = \frac{\bar{p}_k/(1-\bar{p}_k)}{\widehat{\pi_k}/(1-\widehat{\pi_k})}.$$
Mismatch	Close to 0 for each class	The difference between the estimated class proportions and the class membership proportions once individuals have been assigned to a class, i.e. $$Mismatch_k = \widehat{\pi_k} - \frac{N_k}{N},$$ where N_k is the number of individuals in a class and N is the total number.
Entropy	Close to 0	Entropy is a global measure of classification uncertainty, which takes into account all $N \times K$ posterior probabilities. The entropy of a model is defined as $$E = -\sum_{i=1}^{N}\sum_{k=1}^{K} \hat{p}_{ik} \log \hat{p}_{ik},$$ which takes values from $[0, \infty)$, with higher values indicating a larger amount of uncertainty. Entropy values closest to 0 correspond to models with least classification uncertainty.
Relative Entropy	Close to 1	Relative entropy is a standardised version of entropy in the interval $[0,1]$ defined by $$E_K = 1 - \frac{E}{N \log K},$$ where values close to 1 indicate lowest classification uncertainty. In the special case when there is most uncertainty and each individual has equal probability of belonging to each class, $E_K = 0$. Jedidi et al., describes relative entropy as a relative measure of 'fuzziness', and suggested cause concern when close to zero, as this implies that the latent class centroids are not sufficiently separated (11).

For peer review only

Table S4 Concurrence between fixed effect (Model A) and random quadratic effect LCTM models (Model F and G) in men and women in the NIH-AARP cohort

Models	Cohen's κ_w (95% CI)	Concurrence						
**MEN**						Class		
**Model A and F**	0.31		Model F	I	II	II	IV	V
	(0.27,0.34)	Model A	177453	91106	38437	36430	10118	1362
		Class I	120866	81148	4961	34757	0	0
		Class II	44383	7826	28617	1035	6785	120
		Class III	6723	1405	3611	78	1491	138
		Class IV	4763	619	1076	553	1607	908
		Class V	718	108	172	7	235	196
			Model F	I	II	II	IV	V
		Model G	177453	99897	53372	14978	8320	886
**Model F and G**	0.57	Class I	120866	77962	41688	1117	99	0
	(0.56,0.59)	Class II	44383	21684	11464	6433	4796	6
		Class III	6723	219	10	6389	1	104
		Class IV	4763	17	210	746	3402	388
		Class V	718	15	0	293	22	388
**WOMEN**								
**Model A and F**	0.80		Model F	I	II	II	IV	V
	(0.80,0.80)	Model A	111503	50712	40080	14784	4840	1087
		Class I	36311	36311	0	0	0	0
		Class II	45832	13060	32383	389	0	0
		Class III	23544	1066	6881	13008	2589	0
		Class IV	3898	245	588	969	1501	595
		Class V	1918	30	228	418	750	492
**Model F and G**	0.65		Model F	I	II	II	IV	V
	(0.65,0.65)	Model G	111503	74481	25824	6929	3133	1136
		Class I	36311	36299	9	3	0	0
		Class II	45832	37334	8298	200	0	0
		Class III	23544	840	16896	4320	1463	25
		Class IV	3898	5	617	1098	1658	520
		Class V	1918	3	4	1308	12	591

For peer review only

Table S5 Kappa Matrices between Models A to G in men and women in the NIH-AARP cohort

**Models**		**Cohen's Kappa Values (95% CI)**				
**MEN**		Model				
**Unweighted, κ**						
		Model B	Model C	Model D	Model F	Model G
	Model A	0.64 (0.64, 0.64)	0.20 (0.20, 0.21)	0.20 (0.19, 0.20)	0.38 (0.38, 0.38)	0.13 (0.12, 0.13)
	Model B		0.20 (0.20, 0.20)	0.19 (0.19, 0.19)	0.32 (0.31, 0.32)	0.16 (0.16, 0.17)
	Model C			0.59 (0.58, 0.59)	0.48 (0.47, 0.48)	0.43 (0.42, 0.43)
	Model D				0.50 (0.50, 0.50)	0.49 (0.48, 0.49)
	Model F					0.18 (0.18, 0.19)
**Weighted, κ_w**						
		Model B	Model C	Model D	Model F	Model G
	Model A	0.36 (0.34, 0.38)	0.43 (0.41, 0.44)	0.37 (0.34, 0.41)	0.31 (0.27, 0.34)	0.38 (0.36, 0.39)
	Model B		0.43 (0.42, 0.44)	0.39 (0.37, 0.41)	0.43 (0.42, 0.44)	0.39 (0.38, 0.4)
	Model C			0.49 (0.45, 0.52)	0.65 (0.65, 0.65)	0.62 (0.62, 0.62)
	Model D				0.71 (0.71, 0.71)	0.67 (0.67, 0.67)
	Model F					0.57 (0.56, 0.59)
**WOMEN**						
**Unweighted, κ**						
		Model B	Model C	Model D	Model F	Model G
	Model A	0.64 (0.64, 0.64)	0.65 (0.65, 0.66)	0.25 (0.24, 0.25)	0.63 (0.63, 0.63)	0.37 (0.37, 0.38)
	Model B		0.45 (0.44, 0.45)	0.17 (0.17, 0.17)	0.48 (0.47, 0.48)	0.30 (0.29, 0.30)
	Model C			0.26 (0.25, 0.26)	0.49 (0.49, 0.5)	0.41 (0.41, 0.41)
	Model D				0.28 (0.27, 0.28)	0.52 (0.52, 0.53)
	Model F					0.32 (0.31, 0.32)
**Weighted, κ_w**						
		Model B	Model C	Model D	Model F	Model G
	Model A	0.78 (0.78, 0.78)	0.69 (0.69, 0.69)	0.57 (0.57, 0.57)	0.80 (0.80, 0.80)	0.65 (0.65, 0.65)
	Model B		0.48 (0.48, 0.49)	0.42 (0.41, 0.42)	0.68 (0.68, 0.68)	0.45 (0.45, 0.46)
	Model C			0.64 (0.64, 0.64)	0.71 (0.71, 0.71)	0.63 (0.63, 0.63)
	Model D				0.64 (0.63, 0.66)	0.81 (0.81, 0.81)
	Model F					0.65 (0.65, 0.65)

† Kappa values presented here are optimised over the (5!=120) possible combinations of matching the classes labels across the models

For peer review only

Figure S2 Mean trajectories and 95% prediction intervals for each class in model F – men (left) and women (right)

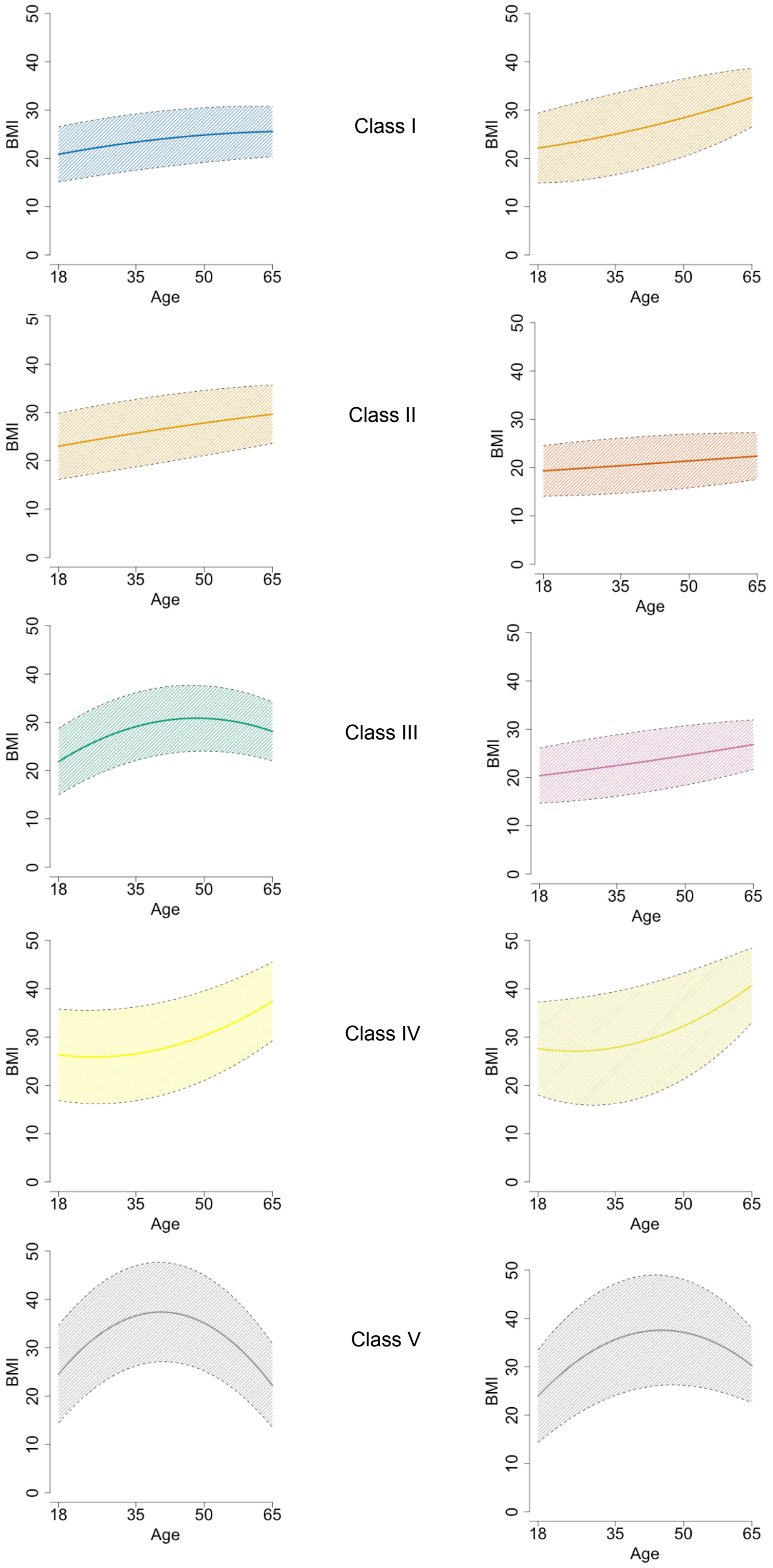

Figure S3 A randomly selected sample of 250 men from each class in model F

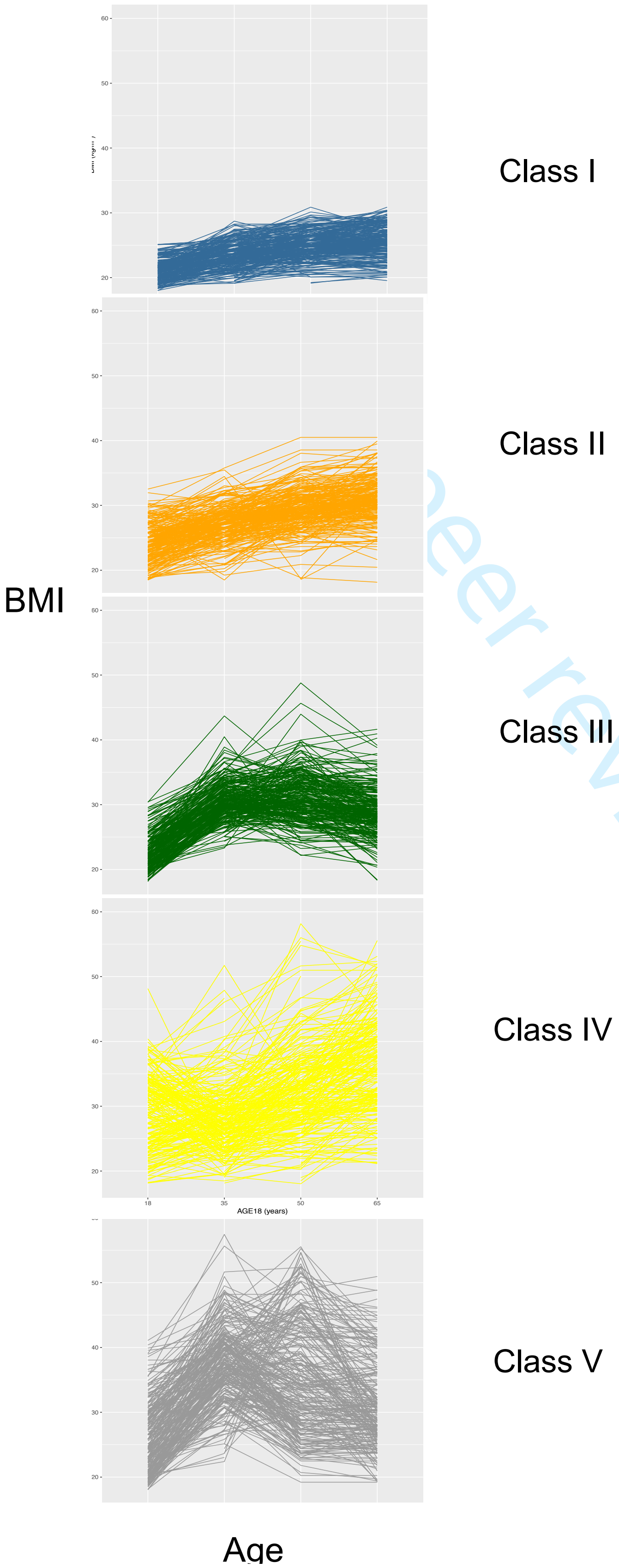

For peer review only

Table S5 Characteristics by five latent classes (Model F) of 177,453 men in the NIH-AARP cohort

	Model F (preferred model)				
	Class I	Class II	Class III	Class IV	Class V
	Stable normal weight	Normal weight to overweight	Normal to stable obese	Overweight to obese	Rapid increase to obese
Number of subjects, N	120 867 (68%)	44 383 (32%)	6 723 (5%)	4 764 (3%)	718 (0.5%)
Mean (sd) entry age, years	62.88 (0.01)	61.59 (0.03)	62.03 (0.06)	59.71 (0.07)	57.73 (0.18)
Mean (sd) current (at baseline) BMI, kg/m^2	25.39 (0.01)	30.84 (0.02)	29.07 (0.05)	35.14 (0.12)	30.51 (0.28)
Mean (sd) BMI at 50 years, kg/m^2	24.66 (0.01)	29.12 (0.02)	31.34 (0.05)	30.95 (0.12)	35.01 (0.35)
Mean (sd) BMI at 35 years, kg/m^2	23.35 (0.01)	26.72 (0.01)	30.24 (0.04)	26.61 (0.09)	37.69 (0.26)
Mean (sd) BMI at 18 years, kg/m^2	20.75 (0.01)	23.92 (0.02)	21.69 (0.04)	27.16 (0.09)	24.76 (0.2)
Mean (sd) maximum BMI, kg/m^2	26.94 (0.01)	32.76 (0.02)	34.09 (0.05)	37.04 (0.13)	40.17 (0.33)
Mean (sd) minimum BMI, kg/m^2	20.56 (0.01)	23.16 (0.02)	22.46 (0.05)	23.15 (0.07)	24.42 (0.20)
Mean (sd) waist circumference, cm	94.38 (0.02)	106.23 (0.05)	102.73 (0.13)	115.24 (0.28)	105.91 (0.64)
Mean (sd) hip circumference, cm	100.28 (0.02)	109.58 (0.05)	107.02 (0.13)	117.73 (0.25)	110.54 (0.62)
Mean (sd) hip-to-waist ratio,	0.94 (<0.01)	0.97 (<0.01)	0.96 (<0.01)	0.98 (<0.01)	0.96 (<0.01)
Mean (sd) height, m	1.78 (<0.01)	1.79 (<0.01)	1.79 (<0.01)	1.81 (<0.01)	1.78 (<0.01)
Comorbidity, %					
Diabetes	8036 (7%)	6155 (14%)	1999 (30%)	861 (18%)	281 (39%)
Heart disease	19927 (16%)	8709 (20%)	1890 (28%)	908 (19%)	165 (23%)
Smoking, %					
Current	11 823 (10%)	3 761 (8%)	638 (9%)	460 (10%)	106 (15%)
Former	67 948 (56%)	26 898 (61%)	3 809 (57%)	3 002 (63%)	370 (52%)
Never	37 149 (31%)	12 130 (27%)	2 012 (30%)	1 110 (23%)	218 (30%)
Missing	3 946 (3%)	1 594 (4%)	264 (4%)	191 (4%)	24 (3%)
Mean (sd) alcohol consumption, g/day	18.69 (0.13)	17.45 (0.21)	15.48 (0.56)	15.57 (0.69)	10.22 (1.14)
Race, %					
Non-Hispanic White	113 685 (94%)	41 819 (94%)	6 303 (94%)	4 447 (93%)	668 (93%)
Hispanic	2 194 (2%)	1 132 (3%)	157 (2%)	145 (3%)	19 (3%)
Black	1 922 (2%)	650 (1%)	129 (2%)	60 (1%)	14 (2%)
Asian, Pacific islander, American Indian, or Alaskan	2 023 (2%)	343 (1%)	48 (1%)	50 (1%)	4 (1%)

For peer review only - http://bmjopen.bmj.com/site/about/guidelines.xhtml

For peer review only

Missing	1 042 (1%)	439 (1%)	86 (1%)	61 (1%)	13 (2%)
Highest Education level achieved, %	5 508 (5%)	2 439 (5%)	415 (6%)	301 (6%)	37 (5%)
Less than 8 years old	16 768 (14%)	6 993 (16%)	1 281 (19%)	813 (17%)	139 (19%)
Completed primary	11 284 (9%)	4 324 (10%)	737 (11%)	474 (10%)	82 (11%)
Completed high school	25 929 (21%)	10 708 (24%)	1 674 (25%)	1 239 (26%)	197 (27%)
Post high school or come college	61 377 (51%)	19 919 (45%)	2 616 (39%)	1 936 (41%)	263 (37%)
College graduate or postgraduate	5 508 (5%)	2 439 (5%)	415 (6%)	301 (6%)	37 (5%)
Vigorous physical activity three or more times per week in the last year, %	91 250 (75%)	29 941 (67%)	4 670 (69%)	2 600 (55%)	438 (61%)
**Nutrition**					
At least 3 fruit servings per day, %	88 699 (73%)	31 603 (71%)	4 855 (72%)	3 335 (70%)	519 (72%)
At least 3 vegetable servings per day, %	56 255 (47%)	19 376 (44%)	3 245 (48%)	2 050 (43%)	364 (51%)
Mean (sd) red meat consumption, g/day	72.68 (0.18)	88.78 (0.37)	86.4 (0.99)	104.96 (1.44)	91.76 (3.6)
Mean (sd) calories per day, kcal	2010 (3)	2095 (5)	2089 (13)	2264 (20)	2146 (42)
Mean (sd) calories per day 10 years ago, kcal					
Mean (sd) calories per day age 12, kcal	1355 (2)	1404 (3)	1473 (8)	1491 (10)	1695 (30)
Drug use					
Ibuprofen use currently					
Aspirin use currently	38720 (32%)	15719 (35%)	2621 (39%)	1697 (36%)	266 (37%)
Fibre					
Mean (sd) fibre from diet age 12, mg/day	9.61 (0.01)	9.85 (0.02)	10.31 (0.07)	10.22 (0.08)	11.2 (0.23)
Mean (sd) fibre from diet 10 years ago, mg/day	6.95 (0.01)	6.95 (0.02)	7.43 (0.05)	7.26 (0.06)	7.71 (0.17)
Mean (sd) fibre CSFII, g/day	20.31 (0.03)	20.45 (0.05)	21.58 (0.14)	21.54 (0.19)	22.6 (0.48)
Mean (sd) fibre total NDSR, g/day	20.71 (0.03)	20.81 (0.05)	21.96 (0.14)	21.93 (0.19)	22.81 (0.5)
Calcium					
Mean (sd) calcium supplement, mg	152.09 (0.75)	136.56 (1.16)	143 (3.1)	138.13 (3.59)	159.2 (10.81)
Mean (sd) calcium from diet current, mg	808.96 (1.34)	845.53 (2.46)	874.55 (6.59)	906.76 (9.07)	910.57 (20.65)
Mean (sd) calcium from diet 10 years ago, mg					
Mean (sd) calcium from diet age 12, mg	653.16 (1.08)	653.03 (1.8)	681.39 (4.77)	672.61 (5.63)	734.68 (15.26)
Folate					
Mean (sd) folate supplement, µg/day	209.35 (0.67)	196.89 (1.13)	207.14 (2.92)	197.72 (3.49)	222.93 (9.74)
Mean (sd) folate pre 1998, µg/day	335.71 (0.48)	336.53 (0.83)	349.14 (2.29)	346.99 (2.85)	355.86 (7.87)

For peer review only - http://bmjopen.bmj.com/site/about/guidelines.xhtml

Mean (sd) folate NDSR, µg/day	447.13 (0.58)	451.13 (1.03)	463.45 (2.85)	470.15 (3.73)	482.46 (10.16)

sd: standard deviation

For peer review only

Table S6 Characteristics by five latent classes (Model F) of 111,503 women in the NIH-AARP cohort

	Model F (preferred model)				
	**Class I** Stable normal weight	**Class II** Normal weight to overweight	**Class III** Normal weight to obese	**Class IV** Overweight to obese	**Class V** Rapid increase to obese
Number of subjects, N	36 311 (32.7%)	45 832 (41.1%)	23 544 (21.1%)	3 898 (3.5%)	1 918 (1.7%)
Mean (sd) entry age, years	62.48 (0.03)	62.23 (0.02)	61.07 (0.03)	59.61 (0.09)	59.69 (0.12)
Mean (sd) current (at baseline) BMI, kg/m^2	21.97 (0.01)	26.66 (0.01)	32.4 (0.03)	37.47 (0.15)	34.4 (0.19)
Mean (sd) BMI at 50 years, kg/m^2	21.16 (0.01)	24.77 (0.01)	29.52 (0.03)	33.51 (0.13)	37.69 (0.2)
Mean (sd) BMI at 35 years, kg/m^2	20.26 (0.01)	22.53 (0.01)	25.51 (0.02)	28.38 (0.1)	36.92 (0.16)
Mean (sd) BMI at 18 years, kg/m^2	19.3 (0.01)	20.44 (0.01)	22.61 (0.02)	29.01 (0.09)	23.97 (0.1)
Mean (sd) maximum BMI, kg/m^2	23.29 (0.01)	28.13 (0.02)	34.72 (0.04)	40.73 (0.14)	43.18 (0.2)
Mean (sd) minimum BMI, kg/m^2	18.57 (0.01)	20.08 (0.01)	21.85 (0.02)	24.05 (0.07)	24.59 (0.11)
Mean (sd) waist circumference, cm	76.19 (0.04)	86.66 (0.05)	97.82 (0.09)	106.29 (0.32)	101.96 (0.41)
Mean (sd) hip circumference, cm	96.78 (0.03)	105.23 (0.04)	114.7 (0.08)	122.14 (0.31)	118.59 (0.42)
Mean (sd) hip-to-waist ratio,	0.78 (<0.01)	0.82 (<0.01)	0.84 (<0.01)	0.84 (<0.01)	0.85 (<0.01)
Mean (sd) height, m	1.65 (<0.01)	1.64 (<0.01)	1.63 (<0.01)	1.63 (<0.01)	1.62 (<0.01)
Comorbidity, %					
Diabetes	680 (2%)	2344 (5%)	3237 (14%)	725 (19%)	604 (31%)
Heart disease	2269 (6%)	3677 (8%)	2518 (11%)	447 (11%)	296 (15%)
Smoking, %					
Current	5803 (16%)	5660 (12%)	2733 (12%)	495 (13%)	214 (11%)
Former	14173 (39%)	18557 (40%)	9748 (41%)	1764 (45%)	731 (38%)
Never	15313 (42%)	20290 (44%)	10417 (44%)	1507 (39%)	909 (47%)
Missing	1022 (3%)	1325 (3%)	646 (3%)	132 (3%)	64 (3%)
Mean (sd) alcohol consumption, g/day	8.07 (0.1)	6.26 (0.08)	4.47 (0.11)	3.92 (0.29)	3.4 (0.48)
Race, %					
Non-Hispanic White	34468 (95%)	42261 (92%)	21178 (90%)	3446 (88%)	1745 (91%)

For peer review only - http://bmjopen.bmj.com/site/about/guidelines.xhtml

Hispanic	699 (2%)	1882 (4%)	1563 (7%)	293 (8%)	105 (5%)
Black	369 (1%)	739 (2%)	324 (1%)	58 (1%)	26 (1%)
Asian, Pacific islander, American Indian, or Alaskan	467 (1%)	443 (1%)	174 (1%)	35 (1%)	15 (1%)
Missing	308 (1%)	507 (1%)	305 (1%)	66 (2%)	27 (1%)
Highest Education level achieved, %					
Less than 8 years old	1155 (3%)	2151 (5%)	1457 (6%)	225 (6%)	124 (6%)
Completed primary	7578 (21%)	11545 (25%)	5984 (25%)	922 (24%)	524 (27%)
Completed high school	3700 (10%)	5021 (11%)	2771 (12%)	489 (13%)	258 (13%)
Post high school or come college	9801 (27%)	11877 (26%)	6088 (26%)	1038 (27%)	521 (27%)
College graduate or postgraduate	14077 (39%)	15238 (33%)	7244 (31%)	1224 (31%)	491 (26%)
Vigorous physical activity three or more times per week in the last year, %	25959 (71%)	30035 (66%)	12692 (54%)	1809 (46%)	1011 (53%)
**Nutrition**					
At least 3 fruit servings per day, %	28441 (78%)	35607 (78%)	17801 (76%)	2889 (74%)	1473 (77%)
At least 3 vegetable servings per day, %	22030 (61%)	27235 (59%)	13282 (56%)	2184 (56%)	1163 (61%)
Mean (sd) red meat consumption, g/day	40.46 (0.19)	46.46 (0.19)	54.41 (0.33)	60.98 (0.98)	56.86 (1.22)
Mean (sd) calories per day, kcal	1546 (4)	1557 (3)	1634 (5)	1727 (15)	1688 (20)
Mean (sd) calories per day 10years ago, kcal	817 (2)	853 (2)	889 (3)	901 (7)	961 (10)
Mean (sd) calories per day age 12, kcal	1250 (3)	1261 (3)	1290 (4)	1336 (10)	1356 (14)
Drug use					
Ibuprofen use currently	4250 (12%)	7065 (15%)	4953 (21%)	1025 (26%)	474 (25%)
Aspirin use currently	6627 (18%)	9559 (21%)	5221 (22%)	913 (23%)	492 (26%)
Hormone Therapy Use, %					
Ever	13989 (39%)	20206 (44%)	12215 (52%)	2241 (57%)	1092 (57%)
Never	22322 (61%)	25626 (56%)	11329 (48%)	1657 (43%)	826 (43%)
Fibre					
Mean (sd) fibre from diet 10years ago, mg/day	7.14 (0.02)	7.22 (0.02)	7.31 (0.03)	7.45 (0.06)	7.57 (0.09)
Mean (sd) fibre from diet age 12, mg/day	8.82 (0.02)	8.96 (0.02)	9.17 (0.03)	9.45 (0.08)	9.39 (0.11)
Mean (sd) fibre CSFII, g/day	18.01 (0.05)	17.75 (0.04)	18.04 (0.06)	18.78 (0.17)	19.14 (0.26)
Mean (sd) fibre total NDSR, g/day	18.05 (0.05)	17.78 (0.04)	18.08 (0.07)	18.85 (0.17)	19.17 (0.26)
Calcium					

For peer review only

Mean (sd) calcium supplement, mg/day	466.85 (2.53)	393.26 (2.1)	337.9 (2.79)	318.84 (6.84)	341.76 (10.22)
Mean (sd) calcium from diet current, mg/day	712.35 (2.23)	716.94 (1.96)	733.95 (2.97)	779.53 (8.16)	765.72 (10.81)
Mean (sd) calcium from diet 10 years ago, mg/day	384.29 (1.18)	387.85 (1.04)	392 (1.5)	390.64 (3.67)	411.4 (5.56)
Mean (sd) calcium from diet age 12, mg/day	677 (2.04)	659.92 (1.81)	650.42 (2.55)	652.48 (6.35)	651.64 (9.03)
Folate					
Mean (sd) folate supplement, µg/day	252.3 (1.23)	239.96 (1.1)	227.06 (1.54)	217.19 (3.78)	228.85 (5.44)
Mean (sd) folate pre 1998, µg/day	291.37 (0.79)	286.07 (0.67)	288.08 (1.02)	295.47 (2.65)	297.1 (3.77)
Mean (sd) folate NDSR, µg/day	375.26 (0.96)	371.15 (0.82)	377.73 (1.26)	389.45 (3.31)	391.35 (4.83)

sd: standard deviation

For peer review only

For peer review only - http://bmjopen.bmj.com/site/about/guidelines.xhtml

Table S7 Characteristics by BMI categories of 177,453 men in the AARP cohort

	Normal weight	Overweight	Obese I	Obese II	Obese III
Number of subjects, N (%)	53466 (30.1)	86967 (49.0)	28234 (15.9)	6033 (3.4)	1878 (1.1)
Mean (sd) entry age, years	62.83 (0.02)	62.47 (0.02)	61.87 (0.03)	61.16 (0.07)	60.48 (0.12)
Mean (sd) current (at baseline) BMI, kg/m^2					
Mean (sd) BMI at 50 years, kg/m^2	23.18 (0.01)	27.16 (0)	31.9 (0.01)	36.86 (0.02)	43.93 (0.1)
Mean (sd) BMI at 35 years, kg/m^2	23.28 (0.01)	26.25 (0.01)	29.74 (0.02)	33.33 (0.05)	38.15 (0.15)
Mean (sd) BMI at 18 years, kg/m^2	22.66 (0.01)	24.64 (0.01)	26.89 (0.02)	29.07 (0.05)	31.7 (0.12)
	20.65 (0.01)	21.75 (0.01)	23.14 (0.02)	24.46 (0.05)	25.92 (0.11)
Mean (sd) maximum BMI, kg/m^2					
Mean (sd) minimum BMI, kg/m^2	25.19 (0.01)	28.89 (0.01)	33.58 (0.02)	38.61 (0.05)	45.63 (0.14)
	20.05 (0.01)	21.47 (0.01)	22.85 (0.02)	24.17 (0.05)	25.57 (0.11)
Mean (sd) waist circumference, cm					
Mean (sd) hip circumference, cm	89.56 (0.03)	98.19 (0.03)	108.65 (0.05)	119.1 (0.12)	132.59 (0.29)
Mean (sd) hip-to-waist ratio,	96.92 (0.03)	103.26 (0.02)	111.41 (0.05)	120.44 (0.14)	132.79 (0.36)
Mean (sd) height, m	0.93 (0)	0.96 (0)	0.98 (0)	1 (0)	1 (0)
	70.23 (0.01)	70.07 (0.01)	70.18 (0.02)	69.93 (0.04)	69.7 (0.08)
Comorbidity, %					
Diabetes	3194 (6%)	7752 (9%)	4375 (15%)	1404 (23%)	555 (30%)
Heart disease	8624 (16%)	15314 (18%)	5788 (21%)	1312 (22%)	410 (22%)
Smoking, %					
Current	6513 (12%)	7345 (8%)	2219 (8%)	427 (7%)	129 (7%)
Former	27129 (51%)	51742 (59%)	17719 (63%)	3818 (63%)	1203 (64%)
Never	18089 (34%)	27129 (51%)	7278 (26%)	1557 (26%)	484 (26%)
Missing	1735 (3%)	2948 (3%)	1018 (4%)	231 (4%)	62 (3%)
Mean (sd) alcohol consumption per day, g	18.24 (0.19)	18.36 (0.15)	18.01 (0.28)	16.41 (0.62)	11.72 (0.88)
Race, %					
Non-Hispanic White	50172 (94%)	81972 (94%)	26548 (94%)	5675 (94%)	1738 (93%)
Hispanic	843 (2%)	1775 (2%)	777 (3%)	171 (3%)	68 (4%)
Black	750 (1%)	1448 (2%)	454 (2%)	86 (1%)	28 (1%)
Asian, Pacific islander, American Indian, or Alaskan	1222 (2%)	1001 (1%)	176 (1%)	34 (1%)	15 (1%)
Missing	479 (1%)	771 (1%)	279 (1%)	67 (1%)	29 (2%)

For peer review only - http://bmjopen.bmj.com/site/about/guidelines.xhtml

Highest Education level achieved, %					
Less than 8 years old	2136 (4%)	4259 (5%)	1695 (6%)	422 (7%)	148 (8%)
Completed primary	6591 (12%)	13066 (15%)	4805 (17%)	1075 (18%)	356 (19%)
Completed high school	4643 (9%)	8406 (10%)	2930 (10%)	639 (11%)	209 (11%)
Post high school or come college	10728 (20%)	19668 (23%)	7120 (25%)	1540 (26%)	498 (27%)
College graduate or postgraduate	29368 (55%)	41568 (48%)	11684 (41%)	2357 (39%)	667 (36%)
Vigorous physical activity three or more times per week in the last year, %	41480 (78%)	64458 (74%)	18280 (65%)	3284 (54%)	786 (42%)
**Nutrition**					
At least 3 fruit servings per day, %	40079 (75%)	62921 (72%)	19870 (70%)	4203 (70%)	1303 (69%)
At least 3 vegetable servings per day, %	32887 (62%)	49213 (57%)	15003 (53%)	3220 (53%)	1037 (55%)
Mean (sd) red meat consumption, g/day	66.09 (0.28)	77.79 (0.21)	93.13 (0.44)	107.21 (1.09)	124.23 (2.72)
Mean (sd) calories per day, kcal	1997.3 (3.96)	2019.26 (3.08)	2123.92 (6.21)	2244.87 (14.7)	2411.64 (34.3)
Mean (sd) calories per day 10 years ago, kcal	861.51 (1.57)	865.75 (1.28)	896.59 (2.4)	936.47 (5.47)	974.51 (10.19)
Mean (sd) calories per day age 12, kcal	667.55 (1.63)	650.14 (1.28)	645.21 (2.27)	657.3 (4.93)	667.25 (9.06)
Drug use					
Ibuprofen use currently,	3969 (15%)	8036 (17%)	3688 (23%)	973 (27%)	408 (36%)
Missing	27029 (51%)	39942 (46%)	11894 (42%)	2460 (41%)	743 (40%)
Aspirin use currently,	16456 (40%)	29091 (43%)	10360 (47%)	2174 (47%)	699 (50%)
Missing	12646 (24%)	18622 (21%)	6146 (22%)	1444 (24%)	469 (25%)
Fibre					
Mean (sd) fibre from diet age 12, mg	9.65 (0.02)	9.69 (0.02)	9.84 (0.03)	10.04 (0.07)	10.3 (0.13)
Mean (sd) fibre from diet 10 years ago, mg	7.12 (0.01)	6.89 (0.01)	6.95 (0.02)	7.1 (0.05)	7.35 (0.09)
Mean (sd) fibre CSFII, g	20.95 (0.05)	20.11 (0.03)	20.26 (0.06)	20.85 (0.14)	22.3 (0.28)
Mean (sd) fibre total NDSR, g	21.35 (0.05)	20.49 (0.03)	20.66 (0.07)	21.22 (0.15)	22.65 (0.29)
Calcium					
Mean (sd) calcium supplement, mg	811.3 (2)	812.92 (1.62)	852.6 (3.12)	906.94 (7.45)	973.28 (16.08)
Mean (sd) calcium from diet current, mg	163.44 (1.18)	144.71 (0.85)	131.79 (1.43)	127.03 (3.03)	122.25 (5.34)
Mean (sd) calcium from diet 10 years ago, mg	406 (1)	398.45 (0.79)	404.71 (1.45)	422.22 (3.28)	426.44 (5.87)
Mean (sd) calcium from diet age 12, mg	667.55 (1.63)	650.14 (1.28)	645.21 (2.27)	657.3 (4.93)	667.25 (9.06)
Folate					

For peer review only - http://bmjopen.bmj.com/site/about/guidelines.xhtml

Mean (sd) folate supplement, g	342.65 (0.74)	332.76 (0.56)	335.07 (1.04)	343.98 (2.3)	360.39 (4.58)
Mean (sd) folate pre 1998, g	217.7 (1.01)	205.22 (0.8)	191.35 (1.41)	186.39 (3.01)	180.32 (5.43)
Mean (sd) folate NDSR, g	455.75 (0.92)	443.69 (0.69)	449.63 (1.3)	464.98 (2.95)	490.92 (5.98)

sd: standard deviation
N=875 men classified as <18.0 kg/m^2 at study entry not included

For peer review only

For peer review only - http://bmjopen.bmj.com/site/about/guidelines.xhtml

Table S8 Characteristics by BMI categories of 111,503 women in the AARP cohort

	Normal weight	Overweight	Obese I	Obese II	Obese III
Number of subjects, N (%)	48273 (43.3)	36025 (32.2)	16245 (14.6)	6126 (5.5.)	3507 (3.1)
Mean (sd) entry age, years	61.96 (0.02)	62.15 (0.03)	61.89 (0.04)	61.21 (0.07)	60.45 (0.09)
Mean (sd) current (at baseline) BMI, kg/m^2	22.45 (0.01)	27.23 (0.01)	32.12 (0.01)	37.14 (0.02)	44.66 (0.08)
Mean (sd) BMI at 50 years, kg/m^2	22.04 (0.01)	25.31 (0.01)	28.75 (0.03)	32.46 (0.06)	38.33 (0.12)
Mean (sd) BMI at 35 years, kg/m^2	21.12 (0.01)	22.93 (0.01)	24.93 (0.03)	27.22 (0.06)	30.72 (0.11)
Mean (sd) BMI at 18 years, kg/m^2	20.11 (0.01)	20.83 (0.01)	21.88 (0.03)	23.03 (0.05)	24.46 (0.08)
Mean (sd) maximum BMI, kg/m^2	24.21 (0.01)	28.88 (0.02)	34.05 (0.03)	39.21 (0.06)	46.57 (0.11)
Mean (sd) minimum BMI, kg/m^2	19.16 (0.01)	20.3 (0.01)	21.44 (0.02)	22.57 (0.05)	24.24 (0.08)
Mean (sd) waist circumference, cm	76.99 (0.04)	88.19 (0.05)	97.92 (0.08)	106.84 (0.13)	120.12 (0.23)
Mean (sd) hip circumference, cm	97.47 (0.03)	106.73 (0.04)	115.24 (0.07)	124.36 (0.14)	137.66 (0.24)
Mean (sd) hip-to-waist ratio,	0.79 (0)	0.83 (0)	0.85 (0)	0.86 (0)	0.85 (0)
Mean (sd) height, m	1218 (3%)	2199 (6%)	2050 (13%)	1211 (20%)	873 (25%)
Comorbidity, %					
Diabetes	1218 (3%)	2199 (6%)	2050 (13%)	1211 (20%)	873 (25%)
Heart disease	3050 (6%)	3043 (8%)	1749 (11%)	779 (13%)	482 (14%)
Smoking, %					
Current	7699 (16%)	4467 (12%)	1584 (10%)	502 (8%)	256 (7%)
Former	18632 (39%)	14883 (41%)	6843 (42%)	2633 (43%)	1586 (45%)
Never	20558 (43%)	15636 (43%)	7380 (45%)	2823 (46%)	1552 (44%)
Missing	1384 (3%)	1039 (3%)	438 (3%)	168 (3%)	113 (3%)
Mean (sd) alcohol consumption per day, g	7.94 (0.09)	5.92 (0.09)	4.3 (0.13)	3.25 (0.21)	2.67 (0.26)
Race, %					
Non-Hispanic White	45651 (95%)	33005 (92%)	14627 (90%)	5471 (89%)	3090 (88%)
Hispanic	988 (2%)	1688 (5%)	1067 (7%)	472 (8%)	299 (9%)
Black	582 (1%)	581 (2%)	225 (1%)	74 (1%)	45 (1%)
Asian, Pacific islander, American Indian, or Alaskan	620 (1%)	333 (1%)	114 (1%)	32 (1%)	16 (0%)
Missing	432 (1%)	418 (1%)	212 (1%)	77 (1%)	57 (2%)

For peer review only - http://bmjopen.bmj.com/site/about/guidelines.xhtml

Highest Education level achieved, %					
Less than 8 years old	1585 (3%)	1797 (5%)	1017 (6%)	417 (7%)	261 (7%)
Completed primary	10367 (21%)	9077 (25%)	4288 (26%)	1604 (26%)	954 (27%)
Completed high school	4914 (10%)	4138 (11%)	1869 (12%)	740 (12%)	459 (13%)
Post high school or come college	12696 (26%)	9375 (26%)	4327 (27%)	1644 (27%)	938 (27%)
College graduate or postgraduate	18711 (39%)	11638 (32%)	4744 (29%)	1721 (28%)	895 (26%)
Vigorous physical activity three or more times per week in the last year, %	34629 (72%)	23170 (64%)	8917 (55%)	2694 (44%)	1241 (35%)
**Nutrition**	37782 (78%)	27905 (77%)	12355 (76%)	4596 (75%)	2559 (73%)
At least 3 fruit servings per day, %	34422 (71%)	24747 (69%)	10907 (67%)	3921 (64%)	2257 (64%)
At least 3 vegetable servings per day, %					
Mean (sd) red meat consumption, g/day	40.35 (0.17)	47.73 (0.22)	54.09 (0.39)	61.25 (0.67)	71.18 (1.07)
Mean (sd) calories per day, kcal	1531.86 (2.99)	1568.13 (3.81)	1626.68 (6.33)	1696.9 (10.28)	1864.32 (16.6)
Mean (sd) calories per day 10years ago, kcal	818.42 (1.6)	859.29 (2.01)	894.97 (3.17)	910.64 (5.15)	962.24 (7.27)
Mean (sd) calories per day age 12, kcal	673.14 (1.77)	658.24 (2.05)	650.22 (3.07)	646.33 (5.04)	657.19 (6.67)
Hormone Therapy Use, %					
Never	18932 (39%)	16154 (45%)	8334 (51%)	3459 (56%)	2261 (64%)
Ever	29341 (61%)	19871 (55%)	7911 (49%)	2667 (44%)	1246 (36%)
Ibuprofen use currently,	5842 (21%)	5795 (26%)	3352 (33%)	1566 (39%)	1098 (47%)
Ibuprofen use missing	20318 (42%)	14129 (39%)	6018 (37%)	2075 (34%)	1191 (34%)
Aspirin use currently,	8875 (28%)	7709 (32%)	3714 (36%)	1445 (38%)	833 (41%)
Asprin use missing	16220 (34%)	12185 (34%)	5777 (36%)	2364 (39%)	1483 (42%)
,					
Fibre					
Mean (sd) fibre from diet 10years ago, mg	8.82 (0.02)	9.01 (0.02)	9.14 (0.04)	9.32 (0.06)	9.5 (0.08)
Mean (sd) fibre from diet age 12, mg	7.14 (0.02)	7.22 (0.02)	7.36 (0.03)	7.34 (0.05)	7.65 (0.07)
Mean (sd) fibre CSFII, g	18 (0.04)	17.74 (0.05)	17.94 (0.08)	18 (0.12)	19.35 (0.18)
Mean (sd) fibre total NDSR, g	18.05 (0.04)	17.77 (0.05)	17.98 (0.08)	18.05 (0.12)	19.43 (0.18)
Calcium					
Mean (sd) calcium supplement, mg	710.52 (1.9)	719.2 (2.26)	733.66 (3.61)	751.64 (5.75)	808.42 (8.73)
Mean (sd) calcium from diet current, mg	458.8 (2.18)	382.25 (2.35)	333.6 (3.31)	307.85 (5.3)	270.5 (6.54)
Mean (sd) calcium from diet 10 years ago, mg	382.09 (1.01)	388.3 (1.19)	397.37 (1.82)	397.86 (2.95)	414.1 (4.09)

For peer review only - http://bmjopen.bmj.com/site/about/guidelines.xhtml

Mean (sd) calcium from diet age 12, mg	673.14 (1.77)	658.24 (2.05)	650.22 (3.07)	646.33 (5.04)	657.19 (6.67)
Folate					
Mean (sd) folate supplement, g	250.24 (1.07)	239.5 (1.25)	225.57 (1.84)	217.96 (3.02)	209.48 (4.03)
Mean (sd) folate pre 1998, g	290.16 (0.68)	285.89 (0.78)	287.43 (1.2)	288.09 (1.98)	304.36 (2.79)
Mean (sd) folate NDSR, g	373.99 (0.82)	371.32 (0.96)	376.02 (1.49)	380.5 (2.43)	407.36 (3.5)

sd: standard deviation
N=1327 Women classified as <18.0 kg/m^2 at study entry not included

For peer review only

Figure S4 Latent Class trajectories derived on individuals with at least three time points, with random quadratic effect proportional specification (model F) (left: Men; right: Women)

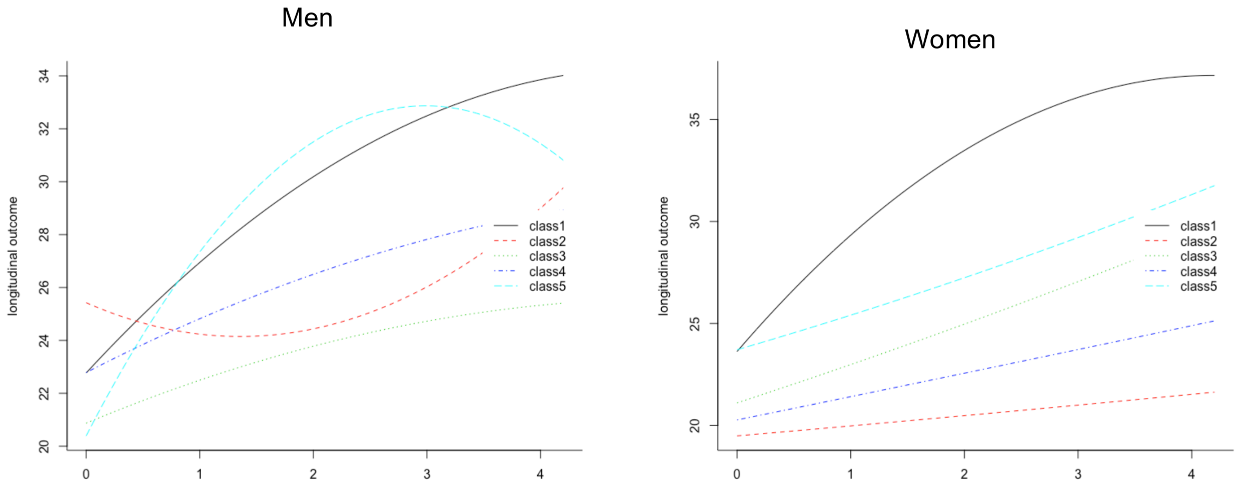

er review only

References

1. Kubzansky LD, Gilthorpe MS, Goodman E. A prospective study of psychological distress and weight status in adolescents/young adults. Annals of Behavioral Medicine 2012;43(2):219-228.

2. Kelly SP, Graubard BI, Andreotti G, Younes N, Cleary SD, Cook MB. Prediagnostic Body Mass Index Trajectories in Relation to Prostate Cancer Incidence and Mortality in the PLCO Cancer Screening Trial. J Natl Cancer Inst 2017;109(3).

3. Song M, Willett WC, Hu FB, Spiegelman D, Must A, Wu K, et al. Trajectory of body shape across the lifespan and cancer risk. Int J Cancer 2016;138(10):2383-95.

4. Song M, Hu FB, Wu K, Must A, Chan AT, Willett WC, et al. Trajectory of body shape in early and middle life and all cause and cause specific mortality: results from two prospective US cohort studies. BMJ 2016;353:i2195.

5. Zheng H, Tumin D, Qian Z. Obesity and Mortality Risk: New Findings From Body Mass Index Trajectories. American Journal of Epidemiology 2013;178(11):1591-1599.

6. Elsensohn M-H, Klich A, Ecochard R, Bastard M, Genolini C, Etard J-F, et al. A graphical method to assess distribution assumption in group-based trajectory models. Statistical methods in medical research 2016;25(2):968-982.

7. Vistisen D, Witte DR, Tabak AG, Herder C, Brunner EJ, Kivimaki M, et al. Patterns of obesity development before the diagnosis of type 2 diabetes: the Whitehall II cohort study. PLoS Med 2014;11(2):e1001602.

8. Lubke G, Neale MC. Distinguishing between latent classes and continuous factors: Resolution by maximum likelihood? Multivariate Behavioral Research 2006;41(4):499-532.

9. Peugh J, Fan X. How Well Does Growth Mixture Modeling Identify Heterogeneous Growth Trajectories? A Simulation Study Examining GMM's Performance Characteristics. Structural Equation Modeling: A Multidisciplinary Journal 2012;19(2):204-226.

10. Peugh J, Fan X. Enumeration Index Performance in Generalized Growth Mixture Models: A Monte Carlo Test of Muthén's (2003) Hypothesis. Structural Equation Modeling: A Multidisciplinary Journal 2015;22(1):115-131.

11. Jedidi K, Ramaswamy V, Desarbo WS. A maximum likelihood method for latent class regression involving a censored dependent variable. Psychometrika 1993;58(3):375-394.

For peer review only

BMJ Open

A framework to construct and interpret latent class trajectory modelling

Journal:	*BMJ Open*
Manuscript ID	bmjopen-2017-020683.R1
Article Type:	Research
Date Submitted by the Author:	07-Feb-2018

Complete List of Authors:	Lennon, Hannah; University of Manchester, Division of Cancer Sciences; University of Manchester Institute of Population Health<br>Kelly, Scott; National Institutes of Health, Division of Cancer Epidemiology and Genetics, National Cancer Institute<br>Sperrin, Matthew; University of Manchester Institute of Population Health, Division of Informatics, Imaging and Data Sciences<br>Buchan, Iain; University of Manchester Institute of Population Health, Division of Informatics, Imaging and Data Sciences<br>Cross, Amanda; Imperial College London School of Public Health, Department of Epidemiology and Biostatistics<br>Leitzmann, Michael; Universitat Regensburg Fakultat fur Medizin, Department for Epidemiology and Preventive Medicine<br>Cook , Michael ; National Institutes of Health, Division of Cancer Epidemiology and Genetics, National Cancer Institute<br>Renehan, Andrew; University of Manchester, Division of Cancer Sciences; University of Manchester Institute of Population Health, Manchester Cancer Research Centre, NIHR Manchester Biochemical Research Centre

**Primary Subject Heading**:	Research methods
Secondary Subject Heading:	Health informatics, Epidemiology, Medical education and training, Communication
Keywords:	Latent class models, Growth curves, Growth mixture models, Lifetime obesity, Trajectories

SCHOLARONE™ Manuscripts

Original article (formatted for BMJ Open)

A framework to construct and interpret latent class trajectory modelling

Hannah Lennon, [1,3] Scott Kelly,[2] Matthew Sperrin,[3] Iain Buchan,[3] Amanda J Cross,[4] Michael Leitzmann,[5] Michael B Cook,[2] Andrew G Renehan[1,3,6]

[1] Division of Cancer Sciences, School of Medical Sciences, Faculty of Biology, Medicine and Health, University of Manchester, Manchester, UK

[2] Division of Cancer Epidemiology and Genetics, National Cancer Institute, National Institutes of Health, Maryland, US

[3] MRC Health eResearch Centre (HeRC), Division of Informatics, Imaging and Data Sciences, University of Manchester, Manchester, UK

[4] Department of Epidemiology and Biostatistics, Imperial College, London, UK

[5] Department of Epidemiology and Preventive Medicine, University of Regensburg, Regensburg, Germany

[6] Manchester Cancer Research Centre, NIHR Manchester Biochemical Research Centre, University of Manchester, Manchester, UK

Running title: *latent class modelling*

Keywords*: Latent class models, growth curves, growth mixture models, body mass index, trajectories, lifetime obesity*

Correspondence to:

Hannah Lennon
Manchester Cancer Research Centre
NIHR Manchester Biomedical Research Centre
Division of Cancer Sciences, School of Medical Sciences,
Faculty of Biology, Medicine and Health,
University of Manchester,
Manchester,
M13 9PL UK
Tel: +44 161 306 7925
E-mail: hannah.lennon@manchester.ac.uk

Abstract: 274 words; main text: 3323 words (max: 3000); 5 tables; 2 figures; References 30 (max: 30); Supplemental material (18 pages); language: UK English.

For peer review only - http://bmjopen.bmj.com/site/about/guidelines.xhtml

For peer review only

ABSTRACT

Objectives: Latent class trajectory modelling (LCTM) is a relatively new methodology in epidemiology to describe life-course exposures, which simplifies heterogeneous populations into homogeneous patterns or classes. However, for a given dataset, it is possible to derive scores of different models based on number of classes, model structure, and trajectory property. Here, we rationalise a systematic framework to derive a 'core' favoured model.

Methods: We developed an eight step framework: step 1, a scoping model; step 2, refining the number of classes; step 3, refining model structure (from fixed-effects through to a flexible random-effect specification); step 4, model adequacy assessment; step 5, graphical presentations; step 6, use of additional discrimination tools ('degree of separation'; Elsensohn's envelope of residual plots); step 7, clinical characterisation and plausibility; and step 8, sensitivity analysis. We illustrated these steps using data from the NIH-AARP cohort of repeated determinations of body mass index (BMI) at baseline (mean age: 62.5 years), and BMI derived by weight recall at ages 18, 35 and 50 years.

Results: From 288,993 participants, we derived a 5-class model for each gender (men: 177,455; women: 111,538). From seven model structures, the favoured model was a proportional random quadratic structure (model F). Favourable properties were also noted for the unrestricted random quadratic structure (model G). However, class proportions varied considerably by model structure – concordance between Models F and G were moderate (Cohen κ: men, 0.57; women, 0.65) but poor with other models. Model adequacy assessments, evaluations using discrimination tools, clinical plausibility, and sensitivity analyses supported our model selection.

Conclusion: We propose a framework to construct and select a 'core' LCTM, which will facilitate generalisability of results in future studies.

Funding: Cancer Research UK National Awareness and Early Detection Initiative (NAEDI), Award Number A17962.

For peer review only - http://bmjopen.bmj.com/site/about/guidelines.xhtml

STRENGTHS AND LIMITATIONS OF THIS STUDY

- We developed a systematic approach, with rationale for each of eight steps, to derive a latent class trajectory model of favoured number of classes and 'core' model structure specification
- The results presented here are based on modelling data from a large well-characterised US cohort; allowing the derivation of numerically meaningful subpopulations (i.e., classes) with distinct phenotypes
- Compared with 'one-off' BMI categorisation, latent class trajectory modelling offers additional phenotypic information and opportunities to identify and intervene early in sub-populations with adverse trajectories
- Whilst we described multiple diagnostic tests, ultimately model selection was based on case-study appropriate model interpretation (for example, model adequacy; discrimination; clinical plausibility; sensitivity analyses) by a multi-disciplinary research team

For peer review only

For peer review only - http://bmjopen.bmj.com/site/about/guidelines.xhtml

INTRODUCTION

In many epidemiological studies, a risk factor is measured at a single point in time and related to the subsequent development of disease, under the assumption that a single 'one-off' measure is an approximation for that exposure over a long time. Thus, baseline measurement of body mass index (BMI) is associated with subsequent development of common disease like cardiovascular disease (1), diabetes (2), several cancers (3), and all-cause mortality (4). This approach is crude and many investigators seek to use alternative methods that might better capture long-term risk factor exposure termed *life-course analysis*. There are widely-used examples that capture cumulative exposure, such as pack-years for smoking and lung cancer, but the assumption that incidence rate is proportional to total lifetime dose is questionable (5). Many other life-course models simply extract features for use in standard regression approaches; for example, a weight change over time. A more sophisticated approach, which takes account of within-individual correlations, is *mixed-effect modelling*, but this is difficult to interpret for public health implementation. An extension of this approach is the use of *latent classes*, also termed growth mixture models.

Latent class trajectory modelling (LCTM) simplifies heterogeneous populations into more homogeneous clusters or classes. From these, one can potentially include random effects to allow for individual variation within these classes. These models have a long history in the criminology (6) and psychology (7) literatures, and now, are increasingly reported in the human epidemiology literature (for example, disentangling the heterogeneity of childhood asthma (8)). Of relevance to this paper, LCTM has been used in association studies of repeated BMI measures with the following endpoints: all-cause mortality (9); cancer incidence (multiple cancer types (10)); gastro-oesophageal (11); prostate (12)); and cancer mortality (12). The LCTM has three general advantages compared with using 'one-off' exposure determinations: first, it better informs aetiological associations by deeply phenotyping certain 'at risk' subpopulations; and second, LCTM offers a public health strategy to identify early divergent adverse trajectories as potential intervention targets. Some researchers additionally argue that LCTM is well-equipped for future forecasting and

For peer review only - http://bmjopen.bmj.com/site/about/guidelines.xhtml

new-patient generalisations in prediction models, as it handles data following a different predictable pattern from that learnt by the model (13). Thirdly, the trajectory approach allows a better understanding of the causes of between-individual variation in certain features (e.g., weight variation over age), by analysing the trajectory as an outcome rather than exposure.

However, LCTM is a complex form of modelling and requires several different structure assumptions (14). Although firmly acknowledged in the GRoLTS-Checklist: Guidelines for Reporting on Latent Trajectory Studies (15), structure-related assumptions have not been systematically evaluated. For many exposures of interest, typically two to seven classes might be described and, as detailed latter, at least seven model structures might be fitted, with and without linear curve properties, such that it is possible to derive greater than eighty different models. Thus, reported differences between studies using latent class modelling might reflect different modelling assumptions rather than true differences between populations. To facilitate the generalizability of results in future studies, here, we propose a framework to construct and select a 'core' LCTM, using an example of repeatedly determined body mass index (BMI) across adulthood in the National Institutes of Health (NIH)-AARP Diet and Health Study cohort. For exposure-disease outcome association analyses, current approaches generally use two stages: first, latent class trajectory modelling, followed by standard association modelling. The framework described here is limited to the first stage.

METHODS

Cohort

The NIH-AARP Diet and Health Study is a US cohort recruited from 1995 (16). A baseline medical and lifestyle questionnaire, including self-reported weight and height, was returned by 566,398 participants (aged 50 to 71 years; mean age: 62.5 years). An additional risk factor questionnaire was mailed in 1996 and completed by a sub-cohort of 327,860; of whom 288,993 (177,455 men and 111,538 women) provided recall weight for all four time-points -

For peer review only - http://bmjopen.bmj.com/site/about/guidelines.xhtml

ages 18, 35 and 50 years. Derived BMIs at baseline and these ages (assuming constant height) form the data in the present analysis. We excluded participants with extreme BMI values (<15 or >70 kg/m^2) recorded at any time point. Means and standard deviations for derived recalled BMI distributions are representative of BMI distributions for historical period-equivalent US populations (17).

Latent class trajectory modelling

We developed an eight step framework **(Table 1)** modelling BMI as a function of age. Latent classes were used to identify subgroups of participants with distinct trajectories (detailed mathematical equations in supplemental material p2) (18). We used maximum likelihood approaches to fit the model with the '*hlme'* function from 'lcmm' library (19) in the R software environment (version 3.2.1) and cross-checked results using the '*PROC TRAJ'* function in 'SAS traj' (SAS Institute, Inc., Cary, NC) (20) (Table S1).

Step 1: We initially constructed a scoping model provisionally selecting the plausible number of classes based on available literature – in the context of BMI trajectories, we used K = 5 classes as reported elsewhere (10, 12). We built models for both genders, as BMI patterns of lifetime changes differ for men and women (21). To determine the initial working model structure, we followed the rationale of Verbeke and Molenburgh (22) and examined the shape of standardised residual plots for each of the five classes in a model with no random-effects. If the residual profile could be approximated by a flat, straight line or a curve, then a random intercept, slope or quadratic term, respectively, were considered. Preliminary plots suggested preference for a quadratic random-effects model (Figure S1).

Step 2: We refined the preliminary working model from step 1 to determine the optimal number of classes, testing K = 1 to 7. The number of classes chosen was based on the lowest Bayesian Information Criteria (BIC).

For peer review only - http://bmjopen.bmj.com/site/about/guidelines.xhtml

Step 3: We further refined the model, using the favoured K derived in step 2, testing for the optimal model structure. We tested seven models (detailed in Table S2): ranging from a simple fixed-effects model (model A) through a rudimentary method that allows the residual variances to vary between classes (model B) to a suite of five random-effects models with different variance structures (models C-G).

Step 4: We then performed a number of model adequacy assessments. First, for each participant, we calculated the posterior probability of being assigned to each trajectory class, and assigned the individual to the class with the highest probability. An average of these maximum Posterior Probability of Assignments (APPA) above 70%, in all classes, is regarded as acceptable (6). We further assessed model adequacy using Odds of Correct Classification (OCC), mismatch scores and Entropy, E_k (detailed in Table S3). These diagnostic tools assist in model selection (6, 23). In some examples, BIC values may decrease as more groups and parameters are added reflecting model overfit. Therefore, the BIC value might not always provide the optimum selection criteria, and model selection must balance between meaningful trajectories, model parsimony and model adequacy. For example, if the model adequacy measures are strongly violated, one might go back to steps 2 and 3 and consider a different model with a higher BIC value. We selected an optimal model structure using the lowest BIC value and satisfactory values from the model adequacy assessments and referred to the outcome of steps 1-4 as the *favoured model*. To assess the interpretability of the resulting classes, we investigated characteristics of lifestyle behaviours of the favoured model such as smoking, alcohol consumption and physical activity.

Step 5: We used three graphical presentation approaches. The conventional approach is to plot mean trajectories with time encompassing each class. Alternatives include the use of mean trajectory plots with 95% predictive intervals for each class, which displays the predicted random variation within each class; or to plot individual level 'spaghetti plots' with

For peer review only - http://bmjopen.bmj.com/site/about/guidelines.xhtml

time (for example, a random sample of participants), which allows the reader to observe the patterns of changes within classes.

Step 6: We assessed model discrimination, including Degrees of Separation, DoS_K (24, 25) and Elsensohn's envelope of residuals (26). To describe the separation of latent trajectory curves, a multivariate Mahalanobis distance was used. Peugh and Fan (25) argue that it is reasonable to speculate that identification of heterogeneous latent trajectories is facilitated by large statistical separation distance among the subpopulations. Thus, larger values of DoS_K indicate the mean trajectories are well separated while DoS_K equal to zero is the special case when all mean trajectories are identical. If the DoS_K value is small, then one might consider a model with fewer classes.

To check structure assumptions in fixed-effects latent class models, Elsenhohn et al. (26) plotted the local standard deviations of the residuals against time. We extended this method to random-effects models; first, computing the observed residuals for each participant; and second, computing the class- and time-specific weighted local variance of the residuals, with weights being the posterior probabilities of individual belonging to a class. We plotted the upper and lower boundary values of the local standard deviations of the residuals around the mean values for each class. The resulting shape indicates the appropriateness of the model assumptions; where non-parallel boundaries indicate heteroscedasticity of residuals suggesting poor model fit, and differing interval widths suggest that across class variability may not be fully accounted for.

Step 7: We assessed for clinical characterisation and plausibility using four approaches: i) assessing the clinical meaningfulness of the trajectory patterns, aiming to include classes with at least 1% capture of the population; ii) assessing the clinical plausibility of the trajectory classes; iii) tabulation of characteristics by latent classes versus conventional categorisations; and iv) concordance of class membership with conventional BMI category

For peer review only - http://bmjopen.bmj.com/site/about/guidelines.xhtml

membership using the kappa statistic (as LCTM is an unsupervised learning approach, we computed κ for all possible combinations and selected the optimal κ).

Step 8: We conducted sensitivity analyses, in this example, with individuals with at least two and three BMI values, as LCTMs are flexible enough to deal with different observation times between participants.

Statistical algorithms

All R and SAS codes used to implement these tools are available via the authors and can be downloaded from *www.github.com/hlennon/LCTMtools.*

RESULTS

Number of classes

From the preliminary working model of a quadratic random-effects model, model F (proportional covariance structure), we derived BICs for up to seven classes: three of the class models failed to converge in men and women. **Table 2** reports that the lowest BIC was obtained with five classes in men and women – confirming our initial working model. The proportions by class in men were 68.1%, 25.0%, 3.8%, 2.7%, and 0.4%; and in women, were 32.6%, 41.1%, 21.1%, 3.5% and 1.7%. For model G (our second favoured model), the lowest BICs were noted for five classes in men and women (Table S2).

Assessment for model structures

With the number of classes now selected as five, we tested the seven model structures – A to G. **Table 3** reports that the lowest BIC was for model F in men and women, justifying the selection of model F in the preliminary working phase. The class sizes varied between models, with Class I ranging from 41% to 68% in men and from 32% to 95% in women. The APPA for each class in model F was 0.81, 0.74, 0.87, 0.83, and 0.74 in men and 0.74, 0.79, 0.80, 0.83 and 0.84 in women, indicating a good discrimination of trajectory. The classes

For peer review only - http://bmjopen.bmj.com/site/about/guidelines.xhtml

were well differentiated with the relative Entropy, E_K values ranging from 0.59 to 0.81 in men and 0.66 to 0.83 in women.

There was moderately good concordance (unweighted and weighted) between the unstructured variance models G with model F in men (κ: 0.57) and women (κ: 0.65) (Tables S4 and S5), but poorer concordance between the favoured models and fixed-effects models in men.

Graphical presentation

We plotted the mean trajectories for model A, B, C, D, F and G in men and women **(Figure 1)** illustrating the increased complexity from model A to G. As alternatives, we plotted separately mean trajectories with 95% predictive intervals for each class, in model F (Figure S2), which displays the predicted random variation within each of the classes with time, noting that variation was greater with the more 'complex' classes (classes IV and V compared with classes I, II and III). Spaghetti plots of individual level data illustrated that the timing and size of BMI changes characterise the classes – for example, sharp increases in BMI in early adulthood in Class III but later in adulthood for Class IV (Figure S3).

Additional tools of suitability of fit

The DoS_k values ranged from 0.10 to 0.36 and 0 to 0.34, in men and women, respectively **(Table 3)**. The covariances were high and in the positive direction and therefore models with non-parallel mean trajectories lead to higher separation.

We plotted the local standard deviation of the residuals with time and found that these were broadly homogeneous, i.e., there were few parallel boundaries **(Figure 2)**. The local residuals for the rapidly obese groups in both genders are the exceptions to parallel lines, which might reflect comorbidities in this group and smaller numbers.

Clinical assessment

For peer review only - http://bmjopen.bmj.com/site/about/guidelines.xhtml

Having established the favoured model, model F with five classes in both genders, we assigned descriptive labels to each respective class as follows **(Table 4)**: stable normal weight; normal weight to overweight; normal weight to obese; overweight to obese; and rapid early obesity. We noted that the proportion in the rapid early obesity (Class V) was less than 1% in men. However, overall, the proportion for Class V for men and women combined was nearly 1%. Thus, we retained this class as we judged it to be clinically meaningful as follows. In both genders, there were rapid increases in obesity from early to middle adulthood, then apparent severe weight reductions. We rationalised that this was clinically plausible, as it could be explained either by intentional (e.g. bariatric surgery) or non-intentional weight loss (e.g. reverse causality from development of disease).

We then tabulated the baseline characteristics according to the five classes for model F in men and women, and noted patterns across the classes **(Table 4** – and fully expanded in Tables S5 and S6). Thus, for example, for current smoking status, there were little differences in patterns by class in men: 10%, 8%, 9%, 10%, and 15%; and in women, 16%, 12%, 12%, 13% and 11%. This contrasts with BMI categories—men (12%, 8%, 8%, 7% and 7%) and women (16%, 12%, 10%, 8%, and 7%) (Tables S7 and S8).

Finally, we noted very poor concordance between the favoured model and conventional BMI categorisation in men (κ: 0.18) and women (κ: 0.52) **(Table 5)**.

Sensitivity analyses

We tested the favoured model using a larger sample of individuals with at least three measures, and found no material differences between these models, in men and women, and the main model (Figure S4).

DISCUSSION

Main findings

We propose an eight-step framework for the construction and selection of models derived from latent class trajectory modelling. We evaluated a range of model structures from fixed-

For peer review only - http://bmjopen.bmj.com/site/about/guidelines.xhtml

effect models to a set of random-effects models, favouring the latter models in this case study, as they include different variance structures, and more likely to reflect the natural history of changes with time in BMI distributions in different sub-populations. We showed that different model structures resulted in different classes with contrasting clinical phenotypes. We propose pre-specified criteria for model selection and that the reporting of a 'core' model will facilitate generalizability of results in future studies.

Context of other literature

To the best of our knowledge, this is the first study to systematically address structure-related assumptions in LCTMs, and their potential impact on clinically-relevant endpoints – in this example, BMI trajectories. Anecdotally, there is a justifiable criticism regarding the use of LCTM models and an uncertainty of how class memberships are derived – a 'black box' effect. The proposed framework, here, encourages the opposite – a transparent stepwise approach to class and model structure selection. To enhance this process, for example, we have 'borrowed' tools developed to address to quantify uncertainty, such as entropy measures, E and E_k, and applied them to assist assessment of model adequacy. A further modification of discrimination measurement with variance estimation has been described by Shah and colleagues (27), and might have importance for class assignment where 'yes/no' treatment decisions are required.

Variations of model A (fixed-effects) have been reported in the clinical literature (9-12), which assume no within-class variability when deriving latent classes. Interpretation in this setting is that variation from the mean trajectory is random, i.e., the correlation between measurements for the same individual is explained by latent class membership. In the context of any repeated measures in the general population, this assumption might not be valid (14). Saunders (28) argued in support of full random-effects models (i.e. models F and G), calling upon Moffitt's theory from criminology, which recognises that "*there are distinct developmental clusters of trajectories of anti-social behaviour that are the result of divergent aetiologies*" – in other words, it is unlikely that latent classes start from a similar baseline.

For peer review only - http://bmjopen.bmj.com/site/about/guidelines.xhtml

The publication of the 16-item GRoLTS Guidelines in 2017 (15) heralded an important advance for the application of LCTM. Here, we add a framework for construction and interpretation.

Strengths and weaknesses

The study has strengths. First, the considered and strategic workflow to optimise identification and application of latent classes provides for a more robust and transparent application of these models in epidemiology. Second, the results presented are based on modelling data from a large well-characterised US cohort, therefore allowing the derivation of numerically meaningful subpopulations (i.e. classes) with distinct phenotypes. We uniquely used averaged kappa values to demonstrate that the LCTM-derived subpopulations are markedly different to those derived from a 'one-off' BMI determinations. In turn, BMI trajectories are more likely to reflect normal clinical practice of considering a 'weight history'. Third, we extensively explored different model selections and adequacy tools, and described extensions to other tools, to supplement model interpretation. Fifth, we further supplement model interpretation, we embedded this project within a multi-disciplinary research team including data scientists, statisticians, clinicians and epidemiologists – an approach echoed elsewhere (29). Finally, we have made the statistical algorithms freely available.

There are several study weaknesses. First, LCTMs currently only considers trajectories of one risk factor at a time. Second, there were only four time points in the AARP such that it was not possible to assess weight cycling. Third, whilst we described multiple diagnostic tests, ultimately model selection was based on case-study appropriate model interpretation (for example, model adequacy; discrimination; clinical plausibility; sensitivity analyses) as well as likelihood-based model-fit criteria (18). The trade-off between BIC efficiency and model adequacy is summarised by Nagin (6) as "…..*the objective of the model selection is not the maximisation of some statistic of model fit …. (but rather) ….to summarise the distinctive features of the data in as parsimonious a fashion as possible".*

For peer review only - http://bmjopen.bmj.com/site/about/guidelines.xhtml

Clinical implications and future research

We showed that different model structures resulted in different classes with contrasting clinical phenotypes. Thus, for example, it is well-recognised that the proportions of current smokers decreases with increasing BMI categories. However, this 'trend' is not observed across the latent classes derived in our favoured model F, suggesting that the clinical characteristics derived from the LCTM differed from those derived from conventional categorisation approaches. Thus, compared with 'one-off' BMI categorisation, latent class trajectory modelling offers additional phenotypic information.

For future research, improving the construction, interpretation and reporting of LCTM (advocated here) is hugely important as the LCTM approach has opportunities to identify and intervene early in sub-populations with adverse trajectories. This approach is analogous to the well-held public strategy of using childhood growth charts to identify and intervening in young children failing to thrive. Thus, in the example of BMI, remembering that 80% of obese adults were not obese in childhood (30), future LCTM studies might identify (new) individuals in their 20s or early 30s on adverse trajectories towards later adulthood obesity. This strategy is a new methodological paradigm, as the repeated measurement of a risk factor (here, BMI) becomes a clinically-relevant endpoint rather than just an exposure.

For peer review only

For peer review only - http://bmjopen.bmj.com/site/about/guidelines.xhtml

Role of the funding source

The funders of the study had no role in study design, data collection, data analysis, data interpretation, or writing of the report.

Conflict of Interest

AGR has received lecture honoraria from Merck Serona and Janssen-Cilag, and independent research funding from Novo Nordisk. All other authors have no conflicts of interest to declare.

Contributions

HL, MS, MC and AGR conceptualised the paper. HL, MS and SK designed the statistical approaches; HL performed the modelling. AC and ML facilitated data access and interpretation of the AARP data. All authors contributed to data interpretation; IB and AGR put modelling into clinical context.

Acknowledgements

We acknowledge the generous funding from Cancer Research UK National Awareness and Early Detection Initiative (NAEDI).

Data sharing statement

All statistical codes used in this paper are available via the authors and can be downloaded from *www.github.com/hlennon/LCTMtools*. Supplementary materials are available from the Dryad repository, DOI: [include DOI here].

For peer review only

For peer review only - http://bmjopen.bmj.com/site/about/guidelines.xhtml

REFERENCES

1. Wormser D, Kaptoge S, Di Angelantonio E, Wood AM, Pennells L, Thompson A, et al. Separate and combined associations of body-mass index and abdominal adiposity with cardiovascular disease: collaborative analysis of 58 prospective studies. Lancet 2011;377(9771):1085-95.

2. Vazquez G, Duval S, Jacobs DR, Jr., Silventoinen K. Comparison of body mass index, waist circumference, and waist/hip ratio in predicting incident diabetes: a meta-analysis. Epidemiol Rev 2007;29:115-28.

3. Lauby-Secretan B, Scoccianti C, Loomis D, Grosse Y, Bianchini F, Straif K. Body Fatness and Cancer--Viewpoint of the IARC Working Group. Colditz G, Anderson AS, Herbert RA, Kaaks R, Thompson HJ, Baker JL, Breda J, Byers T, Cleary MP, Di Cesare M, Gapstur SM, Gunter M, Hursting SD, Leitzmann M, Ligibel J, Renehan A, Romieu I, Shimokawa I, Ulrich CM, Wade K, Weiderpass E. N Engl J Med 2016;375(8):794-8.

4. Global BMI Mortality Collaboration. Body-mass index and all-cause mortality: individual-participant-data meta-analysis of 239 prospective studies in four continents. Lancet 2016.

5. Peto J. That the effects of smoking should be measured in pack-years: misconceptions 4. Br J Cancer 2012;107(3):406-7.

6. Nagin D. Group-based modeling of development: Harvard University Press; 2005.

7. Stevenson J, Thompson MJ, Sonuga-Barke E. Mental health of preschool children and their mothers in a mixed urban/rural population. III. Latent variable models. Br J Psychiatry 1996;168(1):26-32.

8. Belgrave DC, Buchan I, Bishop C, Lowe L, Simpson A, Custovic A. Trajectories of lung function during childhood. Am J Respir Crit Care Med 2014;189(9):1101-9.

9. Song M, Hu FB, Wu K, Must A, Chan AT, Willett WC, et al. Trajectory of body shape in early and middle life and all cause and cause specific mortality: results from two prospective US cohort studies. BMJ 2016;353:i2195.

10. Song M, Willett WC, Hu FB, Spiegelman D, Must A, Wu K, et al. Trajectory of body shape across the lifespan and cancer risk. Int J Cancer 2016;138(10):2383-95.

11. Petrick JL, Kelly SP, Liao LM, Freedman ND, Graubard BI, Cook MB. Body weight trajectories and risk of oesophageal and gastric cardia adenocarcinomas: a pooled analysis of NIH-AARP and PLCO Studies. Br J Cancer 2017;116(7):951-959.

12. Kelly SP, Graubard BI, Andreotti G, Younes N, Cleary SD, Cook MB. Prediagnostic Body Mass Index Trajectories in Relation to Prostate Cancer Incidence and Mortality in the PLCO Cancer Screening Trial. J Natl Cancer Inst 2017;109(3).

13. Qian T, Masino AJ. Latent Patient Cluster Discovery for Robust Future Forecasting and New-Patient Generalization. PLoS One 2016;11(9):e0162812.

14. Gilthorpe MS, Dahly DL, Tu YK, Kubzansky LD, Goodman E. Challenges in modelling the random structure correctly in growth mixture models and the impact this has on model mixtures. J Dev Orig Health Dis 2014;5(3):197-205.

For peer review only - http://bmjopen.bmj.com/site/about/guidelines.xhtml

15. van de Schoot R, Sijbrandij M, Winter SD, Depaoli S, Vermunt JK. The GRoLTS-Checklist: Guidelines for Reporting on Latent Trajectory Studies. . Structural Equation Modeling: A Multidisciplinary Journal;24:451-467.

16. Schatzkin A, Subar AF, Thompson FE, Harlan LC, Tangrea J, Hollenbeck AR, et al. Design and serendipity in establishing a large cohort with wide dietary intake distributions : the National Institutes of Health-American Association of Retired Persons Diet and Health Study. Am J Epidemiol 2001;154(12):1119-25.

17. Renehan AG, Flood A, Adams KF, Olden M, Hollenbeck AR, Cross AJ, et al. Body mass index at different adult ages, weight change, and colorectal cancer risk in the National Institutes of Health-AARP Cohort. American journal of epidemiology 2012;176(12):1130-1140.

18. Tu Y-K, Gilthorpe MS. Statistical thinking in epidemiology: CRC Press; 2011.

19. Proust-Lima C, Philipps V, Diakite A, Liquet B. Package 'lcmm' Title Extended Mixed Models Using Latent Classes and Latent Processes. In; 2016.

20. Jones BL, Nagin DS. Advances in group-based trajectory modeling and an SAS procedure for estimating them. Sociological Methods & Research 2007;35(542-571).

21. Sperrin M, Marshall AD, Higgins V, Buchan IE, Renehan AG. Slowing down of adult body mass index trend increases in England: a latent class analysis of cross-sectional surveys (1992-2010). Int J Obes (Lond) 2014;38(6):818-24.

22. Verbeke G, Molenberghs G. Linear Mixed Models for Longitudinal Data. 2000.

23. Klijn SL, Weijenberg MP, Lemmens P, van den Brandt PA, Lima Passos V. Introducing the fit-criteria assessment plot - A visualisation tool to assist class enumeration in group-based trajectory modelling. Stat Methods Med Res 2015.

24. Lubke G, Neale MC. Distinguishing between latent classes and continuous factors: Resolution by maximum likelihood? Multivariate Behavioral Research 2006;41(4):499-532.

25. Peugh J, Fan X. How Well Does Growth Mixture Modeling Identify Heterogeneous Growth Trajectories? A Simulation Study Examining GMM's Performance Characteristics. Structural Equation Modeling: A Multidisciplinary Journal 2012;19(2):204-226.

26. Elsensohn M-H, Klich A, Ecochard R, Bastard M, Genolini C, Etard J-F, et al. A graphical method to assess distribution assumption in group-based trajectory models. Statistical methods in medical research 2016;25(2):968-982.

27. Shah NH, Hipwell AE, Stepp SD, Chang CC. Measures of discrimination for latent group-based trajectory models. J Appl Stat 2015;42(1):1-11.

28. Saunders JM. Understanding Random Effects in Group-Based Trajectory Modeling: An Application of Moffitt's Developmental Taxonomy. J Drug Issues 2010;40(1):195-220.

29. Belgrave D, Henderson J, Simpson A, Buchan I, Bishop C, Custovic A. Disaggregating asthma: Big investigation versus big data. J Allergy Clin Immunol 2017;139(2):400-407.

30. Simmonds M, Burch J, Llewellyn A, Griffiths C, Yang H, Owen C, et al. The use of measures of obesity in childhood for predicting obesity and the development of obesity-

For peer review only - http://bmjopen.bmj.com/site/about/guidelines.xhtml

related diseases in adulthood: a systematic review and meta-analysis. Health Technol Assess 2015;19(43):1-336.

For peer review only

For peer review only - http://bmjopen.bmj.com/site/about/guidelines.xhtml

Table 1 Framework of eight steps to construct a latent class trajectory model

Step	Step description	Criteria for selection
1	Scope model by provisionally selecting a plausible number of classes based on available literature and the structure based on plausible clinical patterns	Examine linearity of the shape of standardised residual plots for each of the classes in a model with no random-effects
2	Refine the model from step 1 to confirm the optimal number of classes, typically testing K = 1 to 7 classes	Lowest Bayesian Information Criteria (BIC) value
3	Refine optimal model structure from fixed- through to unrestricted random-effects of the model using the favoured K derived in step 2	
4	Run model adequacy assessments as described in Table S3 including Posterior Probability of Assignments (APPA); Odds of Correct Classification (OCC); and relative entropy	i) APPA: average of maximum probabilities should be greater than 70% for all classes<br>ii) OCC values greater than 5.0<br>iii) Relative entropy values greater than 0.5
5	Investigate graphical presentation	i) Plot mean trajectories across time for each class in a single graph<br>ii) Plot mean trajectories with 95% predictive intervals for each class (one class per graph)<br>iii) Plot individual class 'spaghetti plots' across time for a random sample
6	Run additional tools to assess discrimination including Degrees of Separation and Elsensohn's envelope of residuals	i) DoS greater than zero<br>ii) Envelope of residuals is assessed in plots by observing clear separations between classes
7	Assess for clinical characterisation and plausibility	i) Tabulation of characteristics by latent classes. Are the trajectory patterns clinically meaningful? Perhaps, consider classes with a minimum percentage of the population.<br>ii) Are the trajectory patterns clinically plausible?<br>iii) Concordance of class characteristics with those for other well-established variables
8	Conduct sensitivity analyses – for example, testing models without complete data at all time points	General assessment of patterns of trajectories compared with main model

For peer review only

For peer review only - http://bmjopen.bmj.com/site/about/guidelines.xhtml

Table 2 Number of classes (*K* = 1 to 7) using random-effects quadratic structure model F (proportional covariance structure) by gender

Model	*K*	Number of parameters	BIC	Proportions per class %						
				Class I	Class II	Class III	Class IV	Class V	Class VI	Class VII
**MEN**										
**Model F**	1	10	⁎	100						
	2	15	3324009	83	17					
	3	20	3310908	62	32	3				
	4	25	3324128	100	0	0	0			
	5	30	**3301301**	68	25	4	3	0.4		
	6	35	⁎							
	7	40	⁎							
**WOMEN**										
**Model F**	1	10	⁎	100						
	2	15	2195386	86	14					
	3	20	2179080	58	34	8				
	4	25	2179137	100	0	0	0			
	5	30	**2169791**	41	32	21	4	2		
	6	35	⁎							
	7	40	⁎							

⁎ Models failed to converge
BIC: Bayesian Information Criteria
Results from random-effects quadratic structure Model G (unrestricted) by gender are shown in supplemental material TS2.

For peer review only

Table 3 Model adequacy assessments of latent trajectory class models based on different assumptions for *K* = 5 classes, by gender in the NIH-AARP cohort

Model	Description	BIC	Proportion per class %	Average Posterior Probability Assignment (APPA)	Relative Entropy E_k	Degree of Separation DoS_k
**Men**						
A	Homoscedastic	3476322	51: 22: 21: 6: 1	83: 84: 85: 91: 95	0.78	0.10
B	Heteroscedastic	3511646	41: 26: 20: 7: 6	85: 82: 84: 89: 86	-	0.15
C	Random intercept	3364856	68: 24: 4: 3: 2	90: 82: 89: 85: 82	0.81	0.34
D	Random slope	3325463	63: 19: 13: 3: 4	72: 70: 74: 83: 83	0.59	0.26
E	Random quadratic, Equal	*				
F	Random quadratic, Proportional	**3301301**	68: 25: 4: 3: 0.4	81: 74: 87: 83: 74	0.68	0.36
G	Random quadratic, Unrestricted	3320005	56: 30: 8: 5: 0.4	74: 70: 80: 79: 88	0.63	0.33
**Women**						
A	Homoscedastic	2289509	45: 36: 13: 4: 1	90: 84: 88: 93: 96	0.83	0.14
B	Heteroscedastic	2238444	32: 30: 15: 15: 8	87: 83: 85: 89: 91	-	0.05
C	Random intercept	2240681	52: 35: 9: 2: 2	89: 82: 86: 89: 91	0.79	0.21
D	Random slope	2193155	79: 12: 5: 2: 2	91: 80: 79: 86: 91	0.82	0.34
E	Random quadratic, Equal	2188224	95: 5: 0: 0: 0	49: 88: 0: 0: 0	0.26	0.05
F	Random quadratic, Proportional	**2169793**	41: 33: 21: 3: 2	74: 79: 80: 83:84	0.66	0.09
G	Random quadratic, Unrestricted	2187707	67: 23: 6: 3: 1	84: 77: 84:82: 87	0.73	0.34

*Failed to converge

For peer review only

Table 4 Latent class characteristics of 177,453† men and 111,503† women in the NIH-AARP cohort

	Model F (favoured model)				
	**Class I** Stable normal weight	**Class II** Normal weight to overweight	**Class III** Normal weight to obese	**Class IV** Overweight to obese	**Class V** Rapid increase to obese
**MEN**					
Number of participants, N	120 867 (68.1%)	44 383 (25.0%)	6 723 (3.8%)	4 764 (2.7%)	718 (0.4%)
Mean (sd) BMI at 18 years	20.75 (0.01)	23.92 (0.02)	21.69 (0.04)	27.16 (0.09)	24.76 (0.2)
Mean (sd) BMI at 35 years	23.35 (0.01)	26.72 (0.01)	30.24 (0.04)	26.61 (0.09)	37.69 (0.26)
Mean (sd) BMI at 50 years	24.66 (0.01)	29.12 (0.02)	31.34 (0.05)	30.95 (0.12)	35.01 (0.35)
Mean (sd) entry age, years	62.88 (0.01)	61.59 (0.03)	62.03 (0.06)	59.71 (0.07)	57.73 (0.18)
Mean (sd) current (at baseline) BMI, kg/m^2	25.39 (0.01)	30.84 (0.02)	29.07 (0.05)	35.14 (0.12)	30.51 (0.28)
Mean (sd) waist circumference, cm	94.38 (0.02)	106.23 (0.05)	102.73 (0.13)	115.24 (0.28)	105.91 (0.64)
Smoking, %					
Current	11 823 (10%)	3 761 (8%)	638 (9%)	460 (10%)	106 (15%)
Former	67 948 (56%)	26 898 (61%)	3 809 (57%)	3 002 (63%)	370 (52%)
Never	37 149 (31%)	12 130 (27%)	2 012 (30%)	1 110 (23%)	218 (30%)
Missing	3 946 (3%)	1 594 (4%)	264 (4%)	191 (4%)	24 (3%)
Mean (sd) alcohol consumption per day, g	18.69 (0.13)	17.45 (0.21)	15.48 (0.56)	15.57 (0.69)	10.22 (1.14)
**WOMEN**					
Number of participants, N	36 311 (32.6%)	45 832 (41.1%)	23 544 (21.1%)	3 898 (3.5%)	1 918 (1.7%)
Mean (sd) entry age, years	62.48 (0.03)	62.23 (0.02)	61.07 (0.03)	59.61 (0.09)	59.69 (0.12)
Mean (sd) BMI at 18 years	19.3 (0.01)	20.44 (0.01)	22.61 (0.02)	29.01 (0.09)	23.97 (0.1)
Mean (sd) BMI at 35 years	20.26 (0.01)	22.53 (0.01)	25.51 (0.02)	28.38 (0.1)	36.92 (0.16)
Mean (sd) BMI at 50 years	21.16 (0.01)	24.77 (0.01)	29.52 (0.03)	33.51 (0.13)	37.69 (0.2)
Mean (sd) current (at baseline) BMI, kg/m^2	21.97 (0.01)	26.66 (0.01)	32.4 (0.03)	37.47 (0.15)	34.4 (0.19)
Mean (sd) waist circumference, cm	76.19 (0.04)	86.66 (0.05)	97.82 (0.09)	106.29 (0.32)	101.96 (0.41)
Smoking, %					
Current	5803 (16%)	5660 (12%)	2733 (12%)	495 (13%)	214 (11%)
Former	14173 (39%)	18557 (40%)	9748 (41%)	1764 (45%)	731 (38%)
Never	15313 (42%)	20290 (44%)	10417 (44%)	1507 (39%)	909 (47%)
Missing	1022 (3%)	1325 (3%)	646 (3%)	132 (3%)	64 (3%)
Mean (sd) alcohol consumption per day, g	8.07 (0.1)	6.26 (0.08)	4.47 (0.11)	3.92 (0.29)	3.4 (0.48)

For peer review only - http://bmjopen.bmj.com/site/about/guidelines.xhtml

Hormone Therapy Use, %					
Ever	13989 (39%)	20206 (44%)	12215 (52%)	2241 (57%)	1092 (57%)
Never	22322 (61%)	25626 (56%)	11329 (48%)	1657 (43%)	826 (43%)

sd: standard deviation; †Exclusions include 35 women and 2 men with biologically implausible cancers from classes in proportions (2,16,12,0,5) and (1,0,0,1,0) respectively.

For peer review only

Table 5 Concurrence between BMI categories and classes in model F from the NIH-AARP cohort

Latent class	N	BMI categories (kg/m^2) < 18.5	18.5 to 24.9	25.0 to 29.9	30.0 to 34.9	>35.0
		**MEN**				
	177453	875	53466	86967	28234	7911
I	120866	312	50171	68646	1736	1
II	44383	319	1860	14097	23472	4635
III	6723	47	859	3335	1943	539
IV	4763	172	437	679	917	2558
V	718	25	139	210	166	178
		**Cohen κ_ω = 0.182 (0.181,0.183)**				
		**WOMEN**				
	111503	1327	48273	36025	16245	9633
I	36311	1215	33371	1725	0	0
II	45832	3	12584	28636	4607	2
III	23544	83	1683	4777	10587	6414
IV	3898	14	382	519	614	2369
V	1918	12	253	368	437	848
		**Cohen κ_ω= 0.52 (0.51,0.53)**				

BMI: body mass index; κ_ω weighted

For peer review only

FIGURE LEGENDS

Figure 1 BMI mean trajectories by men (left) and women (right) for models A-G. Colours are used to discriminate classes within each plot, but should not be used for direct comparisons across plots.

Figure 2 Illustration of the local (Elsensohn) residual envelope plots (shown here for model B)

For peer review only

For peer review only - http://bmjopen.bmj.com/site/about/guidelines.xhtml

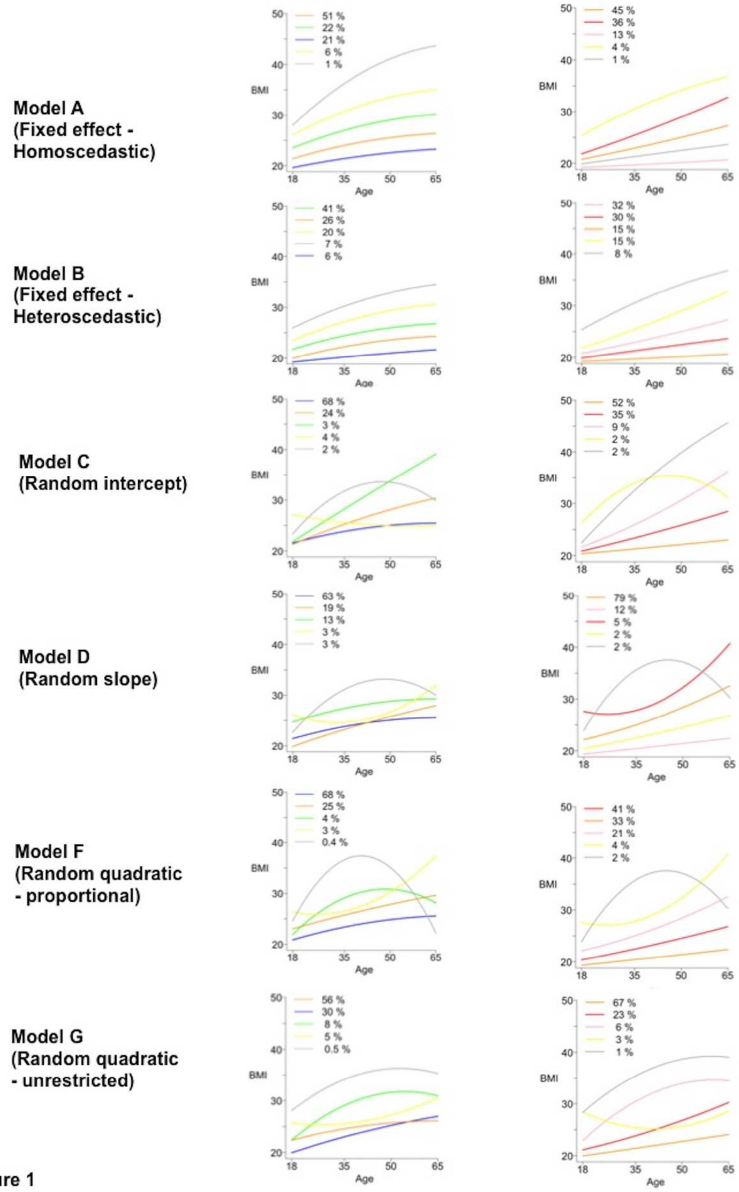

Figure 1

BMI mean trajectories by men (left) and women (right) for models A-G. Colours are used to discriminate classes within each plot, but should not be used for direct comparisons across plots.

254x366mm (300 x 300 DPI)

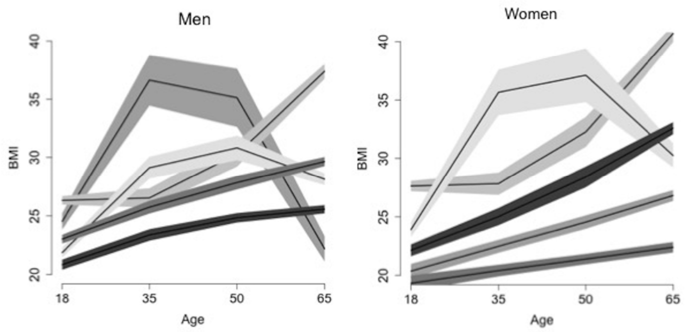

Figure 2

Illustration of the local (Elsensohn) residual envelope plots

254x366mm (300 x 300 DPI)

Supplemental Material

A framework to construct and interpret latent class trajectory modelling

Hannah Lennon[1,3] Scott Kelly,[2] Matthew Sperrin,[3] Iain Buchan,[3] Amanda J Cross,[4] Michael Leitzmann,[5] Michael B Cook,[2] Andrew G Renehan[1,3, 6]

For peer review only

Table S1 Different models with increasing complexity, associated software and referenced use in literature

Model	Description	Interpretation	Software *Command*	References
A	Fixed effects homoscedastic (common residual variance across classes)	No random effects – with the interpretation that any deviation of an individuals trajectory from its mean class trajectory is due to random error only	SAS traj *PROC TRAJ*	(1-5)
B	Fixed effects heteroscedastic (class-specific residual variances)	The same interpretation as Model A with random errors that can be larger and smaller in different classes.	R mmlcr *mmlcr*	(6)
C	Random intercept	The interpretation is allowing individuals to vary in initial weight but each class member is assumed to follow the same shape and magnitude of the mean trajectory	SAS traj *PROC TRAJ*	(7)
D	Random slope	Allowing individuals to vary in initial weight and slope of the mean trajectory but same curvature as trajectory	SAS traj *PROC TRAJ*	(7)
E	Random quadratic – Common variance structure across classes	Additional freedom of allowing individuals to vary within classes by initial weight, shape and magnitude, however each class is assumed to have the same amount of variability	R lcmm *hlme/lcmm*	
F	Random quadratic – Proportionality constraint to allow variance structures to vary across classes	Increasing flexibility of model E as variance structures are allowed to differ up to a multiplicative factor to allow some classes to have larger or smaller within-class variances. This model is can be thought of more parsimonious version of model G from (reducing the number of variance-covariance parameters to be estimated from 6xK parameters to 6+(K-1) parameters.	R lcmm *hlme/lcmm*	
G	Random quadratic – Class-specific variance structure (unstructured)	The most flexible model in which each class has its own separate random quadratic variance structure to describe its own within-class variability. Statistically this permits the variance and covariance of the intercept, slope and quadratic term to vary freely across all classes.	SAS traj *PROC TRAJ*	

[1]The SAS traj package has been converted for Stata users as the traj command in Stata (Collage Station, TX, USA).

For peer review only

MORE DETAILED DESCRIPTION OF MODEL STRUCTURE

For N individuals, the latent class trajectory model we consider is given by:

$$BMI_{itk} = \beta_0^k + \beta_1^k AGE_t + \beta_2^k AGE_t^2 + b_0^k + b_1^k AGE_t + b_2^k AGE_t^2 + \epsilon_t, \qquad \text{(Equation 1)}$$

where BMI_{itk} is the BMI of individual $i = 1, \dots, N$, at time $t = 1, \dots, T$ in class $k = 1, \dots, K$. The random effect b^k is class-specific and follows a multivariate Normal distribution with zero mean and a 3×3 variance-covariance matrix B. For the residual error term ϵ_t, the usual assumption hold, ϵ_t is normally distributed with zero mean and variance σ^2. The probability of an individual belonging to class k is described by a multinomial distribution, i.e.,

$$Pr(individual\ i\ belonging\ to\ class\ k) = \frac{e^{\pi_k}}{\sum_{k=1}^{K} e^{\pi_k}} = \frac{e^{\pi_k}}{e^{\pi_1} + \cdots + e^{\pi_K}}, \qquad \text{(Equation 2)}$$

such that π_k are parameters to be estimated in the model. To select the number of latent classes, we assume a working model (Equation 1) for the random effect structure and the criterion used to select the number of classes was the lowest Bayesian Information Criteria (BIC).

MORE DETAILED DESCRIPTION OF MODEL ADEQUACY ASSESSMENT

Common tools for model adequacy assessment checking are described below in Table S2. Here we give more details of the two extensions of these tools; degree of separation and Elsensohn's residuals (to random effects).

Degrees of separation

A model's ability to detect classes accurately is affected by the degree of separation between latent trajectory curves (8, 9). To describe the separation of latent growth curves, we used the multivariate Mahalanobis distance with the multivariate Mahalanobis distance (D) units defined as:

$$D_{ij} = (\mu_i - \mu_j)^T \Sigma^{-1} (\mu_i - \mu_j), \qquad i, j = 1, \dots K,$$

For peer review only - http://bmjopen.bmj.com/site/about/guidelines.xhtml

where μ_i is a $T\times1$ vector of mean values for class i, and Σ^{-1} is the inverse of a $T\times T$ matrix of sample covariances of at times $t = 1, \dots, T$. The larger the difference, the larger the separation between curves. Peugh and Fan (9) argue that it is reasonable to expect that it is easier to identify heterogeneous latent growth trajectories when the statistical separation distance among the subpopulations is larger than when the separation distance among the latent subpopulations is much smaller.

To give an overall measure of separation for each model, we propose a weighted sum of multivariate Mahalanobis distance matrix with weights being the estimated class proportions, $\hat{\pi}_\iota$.. Then the degree of separation DoS_K is defined as

$$DoS_K = \sum_{i=1}^{K}\sum_{j=1}^{i} \hat{\pi}_\iota \hat{\pi}_J D_{ij}\,, \quad i,j = 1,2,\dots,K.$$

Larger values of DoS_K indicate the mean trajectories are well separated while DoS_K is zero in the special case when all mean trajectories are identical. If the DoS_K value is small, then you may wish to consider a model with fewer classes.

Elsensohn's envelope of residuals

To check the model assumption in fixed effect latent class models, Elsenhohn et al. (6) suggest plotting the local standard deviations of the residuals to check the appropriateness of the model. With the assumption that the residuals are homogeneous over time. To check the appropriateness of each of our model assumptions, we extended their method to include random effects in the models. We compute the local standard deviations of the residuals using the following steps:

1) Compute the observed residuals r_{itk} for each subject i, at time t given the individual is in class k

Equation 1: $r_{itk} = y_{it} - \hat{y}_{itk} = y_{it} - x_i^T\beta^k - z_i^T b^k,$

For peer review only - http://bmjopen.bmj.com/site/about/guidelines.xhtml

where y_{itk} is the observed value for individual i at time t in class k and $\hat{y}_{itk}$ is the fitted value of BMI from our fitted model, here the random effect model. 2) Compute the class- and time-specific weighted local variance of the residuals, $Var_w[r_{itk}]$, with weights being p_{ik}, the posterior probabilities of individual i belonging to group k. #) Plot the upper and lower boundaries for the local standard deviations of the residuals

Equation 2: $b_{itk} = \mu_{tk} \pm \sqrt{Var_w[r_{itk}]}$,

$where\ \mu_{tk}$ is the mean value of class k at time t. 4) Plot the boundaries b_{itk} onto the mean trajectory plots.

The shape of the local standard deviation of the residuals indicates the appropriateness of the model assumptions; where non-parallel boundaries indicate heteroscedasticity of residuals suggesting poor model fit, and differing interval widths suggest across group variability may not be fully accounted for. We believe this complements the others metrics well. If the boundaries suggest a poor fit, you may consider a more complex random effect structure to be more suitable, for example, a higher order polynomial.

For peer review only

For peer review only - http://bmjopen.bmj.com/site/about/guidelines.xhtml

Figure S1 Standardised residual plots of fixed-effect model (model A) for each class justifying the selection of a random-effect structure in the scoping model

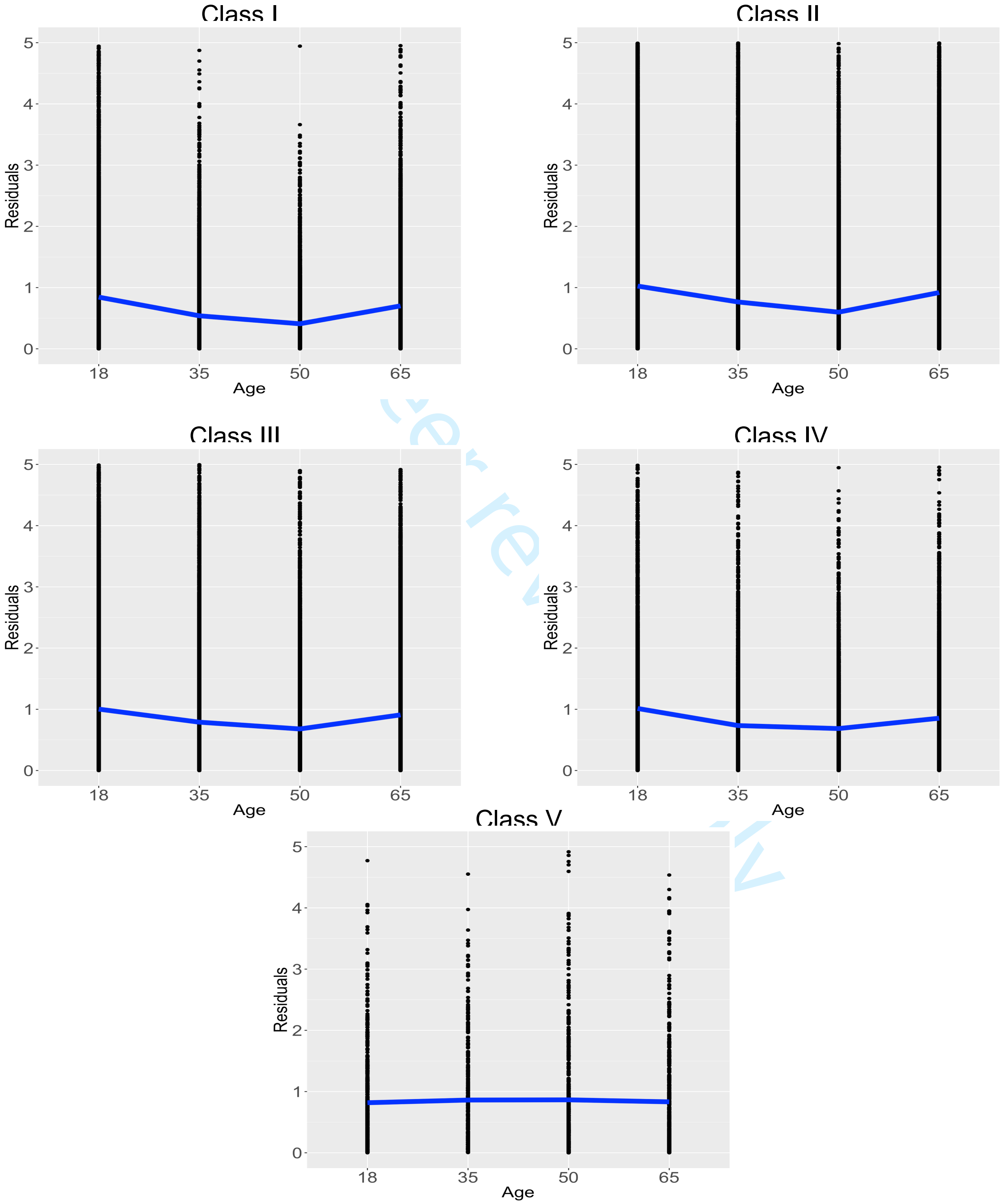

For peer review only - http://bmjopen.bmj.com/site/about/guidelines.xhtml

Table S2 Number of classes (K = 1† to 7) using random quadratic effect models: model G (unrestricted) by gender, NIH-AARP cohort

Model	K	Num of parameters	BIC	Proportions per class (%)						
				Class I	Class II	Class III	Class IV	Class V	Class VI	Class VII
**MEN**										
**Model G**	2	14	3361853	94	6					
	3	21	3341066	87	7	7				
	4	28	3329426	52	37	7	5			
	5	35	**3320005**	51	33	9	6	1		
	6	42	3315240	45	31	12	9	2	1	
	7	48	3311326	51	19	13	7	5	3	0.4
**WOMEN**										
**Model G**	2	14	2219825	89	11					
	3	21	2203726	61	34	0.5				
	4	28	2194293	61	32	5	2			
	5	35	**2187707**	60	29	7	3	1		
	6	42	2184412	63	19	10	4	1	0.5	
	7	48	*							

⁎ Models failed to converge, † Models with K=1 failed to converge

For peer review only - http://bmjopen.bmj.com/site/about/guidelines.xhtml

Table S3 Common model selection tools for latent class trajectory models

Tool	Criteria for a good model	Description
Likelihood based measures	Lowest BIC, AIC,	These likelihood based model selection tools include: Bayesian Information Criteria (BIC), Akaike Information Criteria (AIC), Likelihood Ratio Tests (LRT), Lo-Mendel-Rubin (LMT-LRT), Bootstrap-LRT. (See Peugh and Fan, 2015 for discussion) (10)
APPA	APPA > 70% for each class	The average Posterior Probability Assignment, $\bar{p}_k$, looks at whether individuals are assigned with high probability and the overall average probability of assignment to each class.
Odds of correct classification	OCC > 5.0 for each class	The odds of correct classification (OCC) is the ratio of the odds of a correct classification into each group on the basis of the maximum probability classification rule and the estimated class membership proportions, $\widehat{\pi_k}$, $$OCC_k = \frac{\bar{p}_k/(1-\bar{p}_k)}{\widehat{\pi_k}/(1-\widehat{\pi_k})}.$$
Mismatch	Close to 0 for each class	The difference between the estimated class proportions and the class membership proportions once individuals have been assigned to a class, i.e. $$Mismatch_k = \widehat{\pi_k} - \frac{N_k}{N},$$ where N_k is the number of individuals in a class and N is the total number.
Entropy	Close to 0	Entropy is a global measure of classification uncertainty, which takes into account all $N \times K$ posterior probabilities. The entropy of a model is defined as $$E = -\sum_{i=1}^{N}\sum_{k=1}^{K} \hat{p}_{ik} \log \hat{p}_{ik},$$ which takes values from $[0, \infty)$, with higher values indicating a larger amount of uncertainty. Entropy values closest to 0 correspond to models with least classification uncertainty.
Relative Entropy	Close to 1	Relative entropy is a standardised version of entropy in the interval $[0,1]$ defined by $$E_K = 1 - \frac{E}{N \log K},$$ where values close to 1 indicate lowest classification uncertainty. In the special case when there is most uncertainty and each individual has equal probability of belonging to each class, $E_K = 0$. Jedidi et al., describes relative entropy as a relative measure of 'fuzziness', and suggested cause concern when close to zero, as this implies that the latent class centroids are not sufficiently separated (11).

For peer review only

Table S4 Concurrence between fixed effect (Model A) and random quadratic effect LCTM models (Model F and G) in men and women in the NIH-AARP cohort

Models	Cohen's κ_w (95% CI)	Concurrence						
**MEN**						Class		
**Model A and F**	0.31		Model F	I	II	II	IV	V
	(0.27,0.34)	Model A	177453	91106	38437	36430	10118	1362
		Class I	120866	81148	4961	34757	0	0
		Class II	44383	7826	28617	1035	6785	120
		Class III	6723	1405	3611	78	1491	138
		Class IV	4763	619	1076	553	1607	908
		Class V	718	108	172	7	235	196
			Model F	I	II	II	IV	V
		Model G	177453	99897	53372	14978	8320	886
**Model F and G**	0.57	Class I	120866	77962	41688	1117	99	0
	(0.56,0.59)	Class II	44383	21684	11464	6433	4796	6
		Class III	6723	219	10	6389	1	104
		Class IV	4763	17	210	746	3402	388
		Class V	718	15	0	293	22	388
**WOMEN**								
**Model A and F**	0.80		Model F	I	II	II	IV	V
	(0.80,0.80)	Model A	111503	50712	40080	14784	4840	1087
		Class I	36311	36311	0	0	0	0
		Class II	45832	13060	32383	389	0	0
		Class III	23544	1066	6881	13008	2589	0
		Class IV	3898	245	588	969	1501	595
		Class V	1918	30	228	418	750	492
**Model F and G**	0.65		Model F	I	II	II	IV	V
	(0.65,0.65)	Model G	111503	74481	25824	6929	3133	1136
		Class I	36311	36299	9	3	0	0
		Class II	45832	37334	8298	200	0	0
		Class III	23544	840	16896	4320	1463	25
		Class IV	3898	5	617	1098	1658	520
		Class V	1918	3	4	1308	12	591

For peer review only

Table S5 Kappa Matrices† between Models A to G in men and women in the NIH-AARP cohort

Models	Cohen's Kappa Values (95% CI)					
**MEN**				Model		
**Unweighted, κ**						
		Model B	Model C	Model D	Model F	Model G
	Model A	0.64 (0.64, 0.64)	0.20 (0.20, 0.21)	0.20 (0.19, 0.20)	0.38 (0.38, 0.38)	0.13 (0.12, 0.13)
	Model B		0.20 (0.20, 0.20)	0.19 (0.19, 0.19)	0.32 (0.31, 0.32)	0.16 (0.16, 0.17)
	Model C			0.59 (0.58, 0.59)	0.48 (0.47, 0.48)	0.43 (0.42, 0.43)
	Model D				0.50 (0.50, 0.50)	0.49 (0.48, 0.49)
	Model F					0.18 (0.18, 0.19)
**Weighted, κ_w**						
		Model B	Model C	Model D	Model F	Model G
	Model A	0.36 (0.34, 0.38)	0.43 (0.41, 0.44)	0.37 (0.34, 0.41)	0.31 (0.27, 0.34)	0.38 (0.36, 0.39)
	Model B		0.43 (0.42, 0.44)	0.39 (0.37, 0.41)	0.43 (0.42, 0.44)	0.39 (0.38, 0.4)
	Model C			0.49 (0.45, 0.52)	0.65 (0.65, 0.65)	0.62 (0.62, 0.62)
	Model D				0.71 (0.71, 0.71)	0.67 (0.67, 0.67)
	Model F					0.57 (0.56, 0.59)
**WOMEN**						
**Unweighted, κ**						
		Model B	Model C	Model D	Model F	Model G
	Model A	0.64 (0.64, 0.64)	0.65 (0.65, 0.66)	0.25 (0.24, 0.25)	0.63 (0.63, 0.63)	0.37 (0.37, 0.38)
	Model B		0.45 (0.44, 0.45)	0.17 (0.17, 0.17)	0.48 (0.47, 0.48)	0.30 (0.29, 0.30)
	Model C			0.26 (0.25, 0.26)	0.49 (0.49, 0.5)	0.41 (0.41, 0.41)
	Model D				0.28 (0.27, 0.28)	0.52 (0.52, 0.53)
	Model F					0.32 (0.31, 0.32)
**Weighted, κ_w**						
		Model B	Model C	Model D	Model F	Model G
	Model A	0.78 (0.78, 0.78)	0.69 (0.69, 0.69)	0.57 (0.57, 0.57)	0.80 (0.80, 0.80)	0.65 (0.65, 0.65)
	Model B		0.48 (0.48, 0.49)	0.42 (0.41, 0.42)	0.68 (0.68, 0.68)	0.45 (0.45, 0.46)
	Model C			0.64 (0.64, 0.64)	0.71 (0.71, 0.71)	0.63 (0.63, 0.63)
	Model D				0.64 (0.63, 0.66)	0.81 (0.81, 0.81)
	Model F					0.65 (0.65, 0.65)

† Kappa values presented here are optimised over the (5!=120) possible combinations of matching the classes labels across the models

For peer review only

Figure S2 Mean trajectories and 95% prediction intervals for each class in model F – men (left) and women (right)

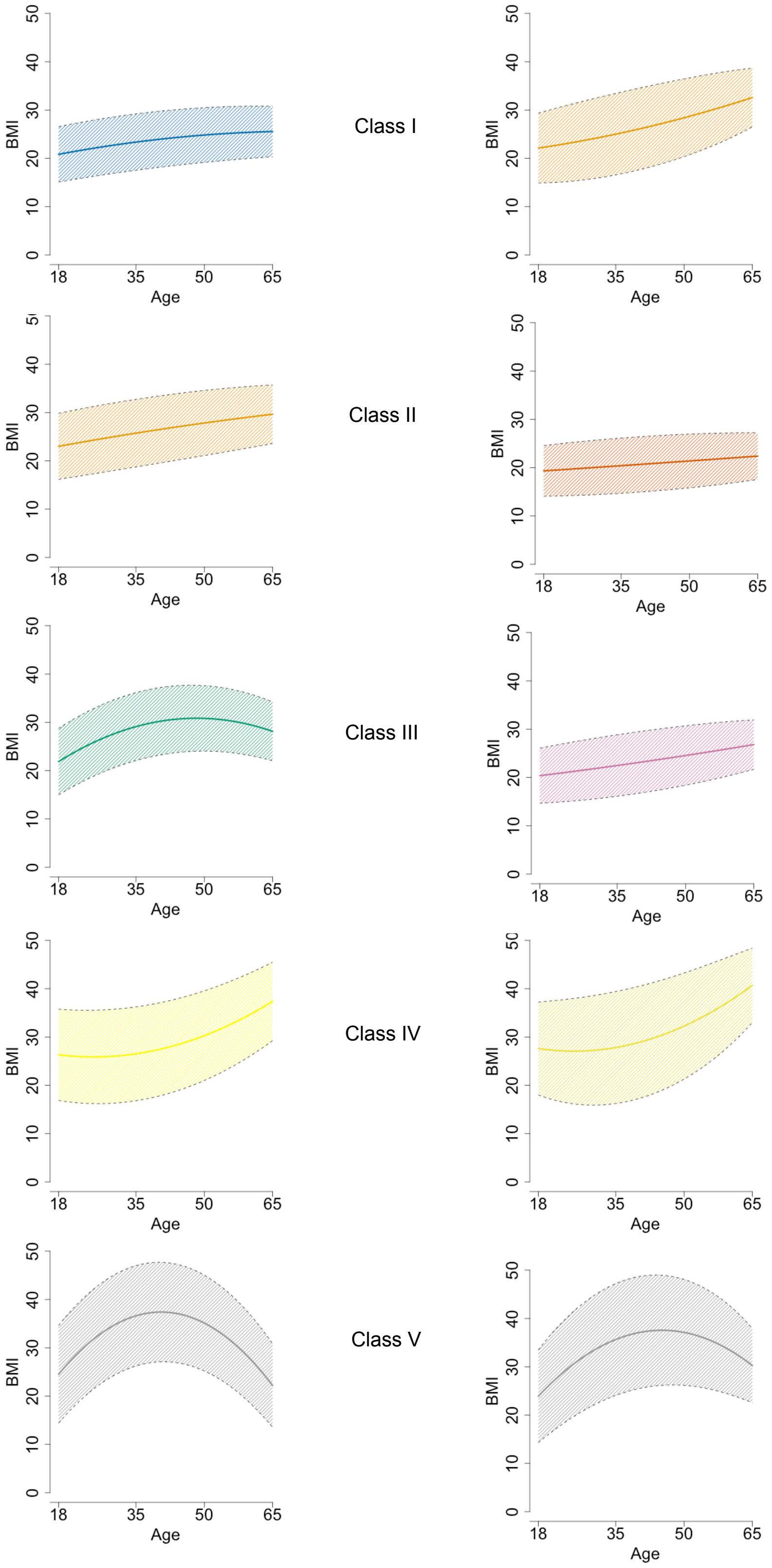

Figure S3 A randomly selected sample of 250 men from each class in model F

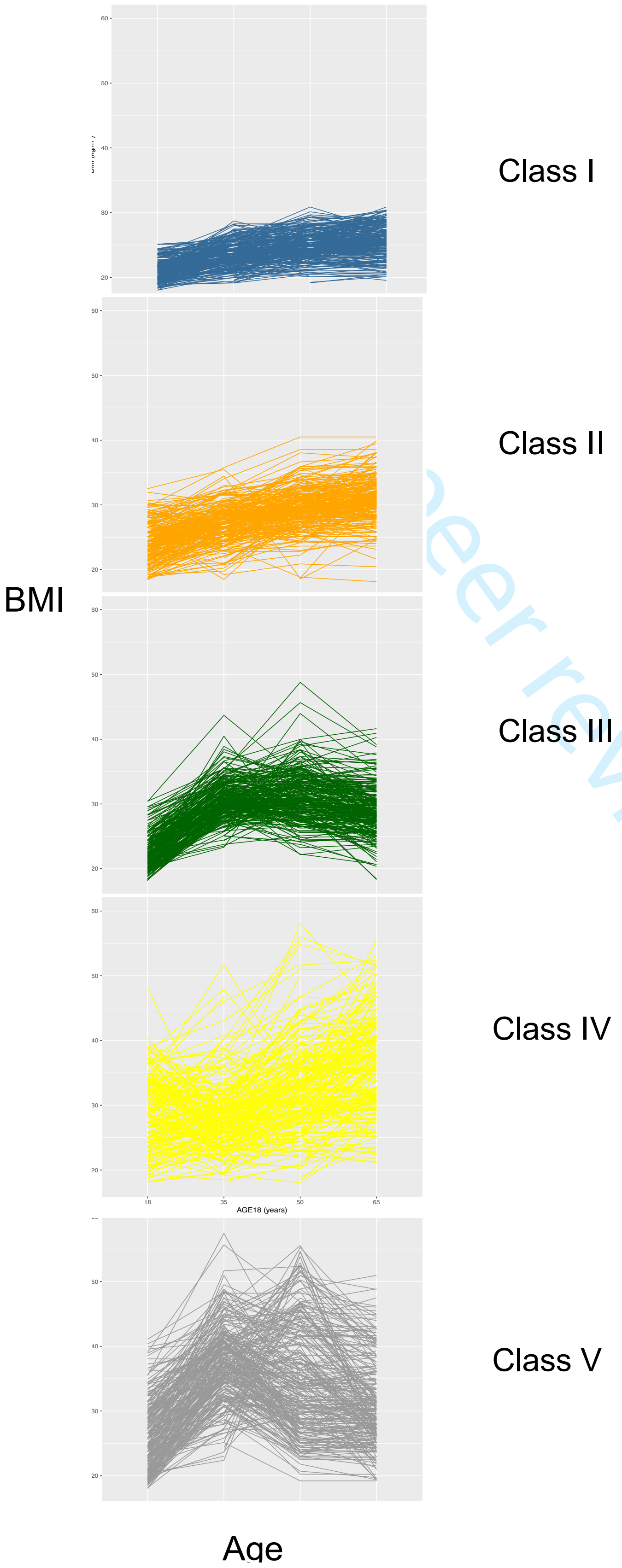

For peer review only - http://bmjopen.bmj.com/site/about/guidelines.xhtml

Table S5 Characteristics by five latent classes (Model F) of 177,453 men in the NIH-AARP cohort

	Model F (preferred model)				
	**Class I**	**Class II**	**Class III**	**Class IV**	**Class V**
	Stable normal weight	Normal weight to overweight	Normal to stable obese	Overweight to obese	Rapid increase to obese
Number of subjects, N	120 867 (68%)	44 383 (32%)	6 723 (5%)	4 764 (3%)	718 (0.5%)
Mean (sd) entry age, years	62.88 (0.01)	61.59 (0.03)	62.03 (0.06)	59.71 (0.07)	57.73 (0.18)
Mean (sd) current (at baseline) BMI, kg/m^2	25.39 (0.01)	30.84 (0.02)	29.07 (0.05)	35.14 (0.12)	30.51 (0.28)
Mean (sd) BMI at 50 years, kg/m^2	24.66 (0.01)	29.12 (0.02)	31.34 (0.05)	30.95 (0.12)	35.01 (0.35)
Mean (sd) BMI at 35 years, kg/m^2	23.35 (0.01)	26.72 (0.01)	30.24 (0.04)	26.61 (0.09)	37.69 (0.26)
Mean (sd) BMI at 18 years, kg/m^2	20.75 (0.01)	23.92 (0.02)	21.69 (0.04)	27.16 (0.09)	24.76 (0.2)
Mean (sd) maximum BMI, kg/m^2	26.94 (0.01)	32.76 (0.02)	34.09 (0.05)	37.04 (0.13)	40.17 (0.33)
Mean (sd) minimum BMI, kg/m^2	20.56 (0.01)	23.16 (0.02)	22.46 (0.05)	23.15 (0.07)	24.42 (0.20)
Mean (sd) waist circumference, cm	94.38 (0.02)	106.23 (0.05)	102.73 (0.13)	115.24 (0.28)	105.91 (0.64)
Mean (sd) hip circumference, cm	100.28 (0.02)	109.58 (0.05)	107.02 (0.13)	117.73 (0.25)	110.54 (0.62)
Mean (sd) hip-to-waist ratio,	0.94 (<0.01)	0.97 (<0.01)	0.96 (<0.01)	0.98 (<0.01)	0.96 (<0.01)
Mean (sd) height, m	1.78 (<0.01)	1.79 (<0.01)	1.79 (<0.01)	1.81 (<0.01)	1.78 (<0.01)
Comorbidity, %					
Diabetes	8036 (7%)	6155 (14%)	1999 (30%)	861 (18%)	281 (39%)
Heart disease	19927 (16%)	8709 (20%)	1890 (28%)	908 (19%)	165 (23%)
Smoking, %					
Current	11 823 (10%)	3 761 (8%)	638 (9%)	460 (10%)	106 (15%)
Former	67 948 (56%)	26 898 (61%)	3 809 (57%)	3 002 (63%)	370 (52%)
Never	37 149 (31%)	12 130 (27%)	2 012 (30%)	1 110 (23%)	218 (30%)
Missing	3 946 (3%)	1 594 (4%)	264 (4%)	191 (4%)	24 (3%)
Mean (sd) alcohol consumption, g/day	18.69 (0.13)	17.45 (0.21)	15.48 (0.56)	15.57 (0.69)	10.22 (1.14)
Race, %					
Non-Hispanic White	113 685 (94%)	41 819 (94%)	6 303 (94%)	4 447 (93%)	668 (93%)
Hispanic	2 194 (2%)	1 132 (3%)	157 (2%)	145 (3%)	19 (3%)
Black	1 922 (2%)	650 (1%)	129 (2%)	60 (1%)	14 (2%)
Asian, Pacific islander, American Indian, or Alaskan	2 023 (2%)	343 (1%)	48 (1%)	50 (1%)	4 (1%)

For peer review only

Missing	1 042 (1%)	439 (1%)	86 (1%)	61 (1%)	13 (2%)
Highest Education level achieved, %	5 508 (5%)	2 439 (5%)	415 (6%)	301 (6%)	37 (5%)
Less than 8 years old	16 768 (14%)	6 993 (16%)	1 281 (19%)	813 (17%)	139 (19%)
Completed primary	11 284 (9%)	4 324 (10%)	737 (11%)	474 (10%)	82 (11%)
Completed high school	25 929 (21%)	10 708 (24%)	1 674 (25%)	1 239 (26%)	197 (27%)
Post high school or come college	61 377 (51%)	19 919 (45%)	2 616 (39%)	1 936 (41%)	263 (37%)
College graduate or postgraduate	5 508 (5%)	2 439 (5%)	415 (6%)	301 (6%)	37 (5%)
Vigorous physical activity three or more times per week in the last year, %	91 250 (75%)	29 941 (67%)	4 670 (69%)	2 600 (55%)	438 (61%)
**Nutrition**					
At least 3 fruit servings per day, %	88 699 (73%)	31 603 (71%)	4 855 (72%)	3 335 (70%)	519 (72%)
At least 3 vegetable servings per day, %	56 255 (47%)	19 376 (44%)	3 245 (48%)	2 050 (43%)	364 (51%)
Mean (sd) red meat consumption, g/day	72.68 (0.18)	88.78 (0.37)	86.4 (0.99)	104.96 (1.44)	91.76 (3.6)
Mean (sd) calories per day, kcal	2010 (3)	2095 (5)	2089 (13)	2264 (20)	2146 (42)
Mean (sd) calories per day 10 years ago, kcal					
Mean (sd) calories per day age 12, kcal	1355 (2)	1404 (3)	1473 (8)	1491 (10)	1695 (30)
Drug use					
Ibuprofen use currently					
Aspirin use currently	38720 (32%)	15719 (35%)	2621 (39%)	1697 (36%)	266 (37%)
Fibre					
Mean (sd) fibre from diet age 12, mg/day	9.61 (0.01)	9.85 (0.02)	10.31 (0.07)	10.22 (0.08)	11.2 (0.23)
Mean (sd) fibre from diet 10 years ago, mg/day	6.95 (0.01)	6.95 (0.02)	7.43 (0.05)	7.26 (0.06)	7.71 (0.17)
Mean (sd) fibre CSFII, g/day	20.31 (0.03)	20.45 (0.05)	21.58 (0.14)	21.54 (0.19)	22.6 (0.48)
Mean (sd) fibre total NDSR, g/day	20.71 (0.03)	20.81 (0.05)	21.96 (0.14)	21.93 (0.19)	22.81 (0.5)
Calcium					
Mean (sd) calcium supplement, mg	152.09 (0.75)	136.56 (1.16)	143 (3.1)	138.13 (3.59)	159.2 (10.81)
Mean (sd) calcium from diet current, mg	808.96 (1.34)	845.53 (2.46)	874.55 (6.59)	906.76 (9.07)	910.57 (20.65)
Mean (sd) calcium from diet 10 years ago, mg					
Mean (sd) calcium from diet age 12, mg	653.16 (1.08)	653.03 (1.8)	681.39 (4.77)	672.61 (5.63)	734.68 (15.26)
Folate					
Mean (sd) folate supplement, µg/day	209.35 (0.67)	196.89 (1.13)	207.14 (2.92)	197.72 (3.49)	222.93 (9.74)
Mean (sd) folate pre 1998, µg/day	335.71 (0.48)	336.53 (0.83)	349.14 (2.29)	346.99 (2.85)	355.86 (7.87)

For peer review only - http://bmjopen.bmj.com/site/about/guidelines.xhtml

Mean (sd) folate NDSR, µg/day	447.13 (0.58)	451.13 (1.03)	463.45 (2.85)	470.15 (3.73)	482.46 (10.16)

sd: standard deviation

For peer review only

For peer review only - http://bmjopen.bmj.com/site/about/guidelines.xhtml

Table S6 Characteristics by five latent classes (Model F) of 111,503 women in the NIH-AARP cohort

	Model F (preferred model)				
	**Class I**	**Class II**	**Class III**	**Class IV**	**Class V**
	Stable normal weight	Normal weight to overweight	Normal weight to obese	Overweight to obese	Rapid increase to obese
Number of subjects, N	36 311 (32.7%)	45 832 (41.1%)	23 544 (21.1%)	3 898 (3.5%)	1 918 (1.7%)
Mean (sd) entry age, years	62.48 (0.03)	62.23 (0.02)	61.07 (0.03)	59.61 (0.09)	59.69 (0.12)
Mean (sd) current (at baseline) BMI, kg/m^2	21.97 (0.01)	26.66 (0.01)	32.4 (0.03)	37.47 (0.15)	34.4 (0.19)
Mean (sd) BMI at 50 years, kg/m^2	21.16 (0.01)	24.77 (0.01)	29.52 (0.03)	33.51 (0.13)	37.69 (0.2)
Mean (sd) BMI at 35 years, kg/m^2	20.26 (0.01)	22.53 (0.01)	25.51 (0.02)	28.38 (0.1)	36.92 (0.16)
Mean (sd) BMI at 18 years, kg/m^2	19.3 (0.01)	20.44 (0.01)	22.61 (0.02)	29.01 (0.09)	23.97 (0.1)
Mean (sd) maximum BMI, kg/m^2	23.29 (0.01)	28.13 (0.02)	34.72 (0.04)	40.73 (0.14)	43.18 (0.2)
Mean (sd) minimum BMI, kg/m^2	18.57 (0.01)	20.08 (0.01)	21.85 (0.02)	24.05 (0.07)	24.59 (0.11)
Mean (sd) waist circumference, cm	76.19 (0.04)	86.66 (0.05)	97.82 (0.09)	106.29 (0.32)	101.96 (0.41)
Mean (sd) hip circumference, cm	96.78 (0.03)	105.23 (0.04)	114.7 (0.08)	122.14 (0.31)	118.59 (0.42)
Mean (sd) hip-to-waist ratio,	0.78 (<0.01)	0.82 (<0.01)	0.84 (<0.01)	0.84 (<0.01)	0.85 (<0.01)
Mean (sd) height, m	1.65 (<0.01)	1.64 (<0.01)	1.63 (<0.01)	1.63 (<0.01)	1.62 (<0.01)
Comorbidity, %					
Diabetes	680 (2%)	2344 (5%)	3237 (14%)	725 (19%)	604 (31%)
Heart disease	2269 (6%)	3677 (8%)	2518 (11%)	447 (11%)	296 (15%)
Smoking, %					
Current	5803 (16%)	5660 (12%)	2733 (12%)	495 (13%)	214 (11%)
Former	14173 (39%)	18557 (40%)	9748 (41%)	1764 (45%)	731 (38%)
Never	15313 (42%)	20290 (44%)	10417 (44%)	1507 (39%)	909 (47%)
Missing	1022 (3%)	1325 (3%)	646 (3%)	132 (3%)	64 (3%)
Mean (sd) alcohol consumption, g/day	8.07 (0.1)	6.26 (0.08)	4.47 (0.11)	3.92 (0.29)	3.4 (0.48)
Race, %					
Non-Hispanic White	34468 (95%)	42261 (92%)	21178 (90%)	3446 (88%)	1745 (91%)

For peer review only

For peer review only - http://bmjopen.bmj.com/site/about/guidelines.xhtml

Hispanic	699 (2%)	1882 (4%)	1563 (7%)	293 (8%)	105 (5%)
Black	369 (1%)	739 (2%)	324 (1%)	58 (1%)	26 (1%)
Asian, Pacific islander, American Indian, or Alaskan	467 (1%)	443 (1%)	174 (1%)	35 (1%)	15 (1%)
Missing	308 (1%)	507 (1%)	305 (1%)	66 (2%)	27 (1%)
Highest Education level achieved, %					
Less than 8 years old	1155 (3%)	2151 (5%)	1457 (6%)	225 (6%)	124 (6%)
Completed primary	7578 (21%)	11545 (25%)	5984 (25%)	922 (24%)	524 (27%)
Completed high school	3700 (10%)	5021 (11%)	2771 (12%)	489 (13%)	258 (13%)
Post high school or come college	9801 (27%)	11877 (26%)	6088 (26%)	1038 (27%)	521 (27%)
College graduate or postgraduate	14077 (39%)	15238 (33%)	7244 (31%)	1224 (31%)	491 (26%)
Vigorous physical activity three or more times per week in the last year, %	25959 (71%)	30035 (66%)	12692 (54%)	1809 (46%)	1011 (53%)
**Nutrition**					
At least 3 fruit servings per day, %	28441 (78%)	35607 (78%)	17801 (76%)	2889 (74%)	1473 (77%)
At least 3 vegetable servings per day, %	22030 (61%)	27235 (59%)	13282 (56%)	2184 (56%)	1163 (61%)
Mean (sd) red meat consumption, g/day	40.46 (0.19)	46.46 (0.19)	54.41 (0.33)	60.98 (0.98)	56.86 (1.22)
Mean (sd) calories per day, kcal	1546 (4)	1557 (3)	1634 (5)	1727 (15)	1688 (20)
Mean (sd) calories per day 10years ago, kcal	817 (2)	853 (2)	889 (3)	901 (7)	961 (10)
Mean (sd) calories per day age 12, kcal	1250 (3)	1261 (3)	1290 (4)	1336 (10)	1356 (14)
Drug use					
Ibuprofen use currently	4250 (12%)	7065 (15%)	4953 (21%)	1025 (26%)	474 (25%)
Aspirin use currently	6627 (18%)	9559 (21%)	5221 (22%)	913 (23%)	492 (26%)
Hormone Therapy Use, %					
Ever	13989 (39%)	20206 (44%)	12215 (52%)	2241 (57%)	1092 (57%)
Never	22322 (61%)	25626 (56%)	11329 (48%)	1657 (43%)	826 (43%)
Fibre					
Mean (sd) fibre from diet 10years ago, mg/day	7.14 (0.02)	7.22 (0.02)	7.31 (0.03)	7.45 (0.06)	7.57 (0.09)
Mean (sd) fibre from diet age 12, mg/day	8.82 (0.02)	8.96 (0.02)	9.17 (0.03)	9.45 (0.08)	9.39 (0.11)
Mean (sd) fibre CSFII, g/day	18.01 (0.05)	17.75 (0.04)	18.04 (0.06)	18.78 (0.17)	19.14 (0.26)
Mean (sd) fibre total NDSR, g/day	18.05 (0.05)	17.78 (0.04)	18.08 (0.07)	18.85 (0.17)	19.17 (0.26)
Calcium					

For peer review only - http://bmjopen.bmj.com/site/about/guidelines.xhtml

Mean (sd) calcium supplement, mg/day	466.85 (2.53)	393.26 (2.1)	337.9 (2.79)	318.84 (6.84)	341.76 (10.22)
Mean (sd) calcium from diet current, mg/day	712.35 (2.23)	716.94 (1.96)	733.95 (2.97)	779.53 (8.16)	765.72 (10.81)
Mean (sd) calcium from diet 10 years ago, mg/day	384.29 (1.18)	387.85 (1.04)	392 (1.5)	390.64 (3.67)	411.4 (5.56)
Mean (sd) calcium from diet age 12, mg/day	677 (2.04)	659.92 (1.81)	650.42 (2.55)	652.48 (6.35)	651.64 (9.03)
Folate					
Mean (sd) folate supplement, µg/day	252.3 (1.23)	239.96 (1.1)	227.06 (1.54)	217.19 (3.78)	228.85 (5.44)
Mean (sd) folate pre 1998, µg/day	291.37 (0.79)	286.07 (0.67)	288.08 (1.02)	295.47 (2.65)	297.1 (3.77)
Mean (sd) folate NDSR, µg/day	375.26 (0.96)	371.15 (0.82)	377.73 (1.26)	389.45 (3.31)	391.35 (4.83)

sd: standard deviation

For peer review only

Table S7 Characteristics by BMI categories of 177,453 men in the AARP cohort

	Normal weight	Overweight	Obese I	Obese II	Obese III
Number of subjects, N (%)	53466 (30.1)	86967 (49.0)	28234 (15.9)	6033 (3.4)	1878 (1.1)
Mean (sd) entry age, years	62.83 (0.02)	62.47 (0.02)	61.87 (0.03)	61.16 (0.07)	60.48 (0.12)
Mean (sd) current (at baseline) BMI, kg/m^2					
Mean (sd) BMI at 50 years, kg/m^2	23.18 (0.01)	27.16 (0)	31.9 (0.01)	36.86 (0.02)	43.93 (0.1)
Mean (sd) BMI at 35 years, kg/m^2	23.28 (0.01)	26.25 (0.01)	29.74 (0.02)	33.33 (0.05)	38.15 (0.15)
Mean (sd) BMI at 18 years, kg/m^2	22.66 (0.01)	24.64 (0.01)	26.89 (0.02)	29.07 (0.05)	31.7 (0.12)
	20.65 (0.01)	21.75 (0.01)	23.14 (0.02)	24.46 (0.05)	25.92 (0.11)
Mean (sd) maximum BMI, kg/m^2					
Mean (sd) minimum BMI, kg/m^2	25.19 (0.01)	28.89 (0.01)	33.58 (0.02)	38.61 (0.05)	45.63 (0.14)
	20.05 (0.01)	21.47 (0.01)	22.85 (0.02)	24.17 (0.05)	25.57 (0.11)
Mean (sd) waist circumference, cm					
Mean (sd) hip circumference, cm	89.56 (0.03)	98.19 (0.03)	108.65 (0.05)	119.1 (0.12)	132.59 (0.29)
Mean (sd) hip-to-waist ratio,	96.92 (0.03)	103.26 (0.02)	111.41 (0.05)	120.44 (0.14)	132.79 (0.36)
Mean (sd) height, m	0.93 (0)	0.96 (0)	0.98 (0)	1 (0)	1 (0)
	70.23 (0.01)	70.07 (0.01)	70.18 (0.02)	69.93 (0.04)	69.7 (0.08)
Comorbidity, %					
Diabetes	3194 (6%)	7752 (9%)	4375 (15%)	1404 (23%)	555 (30%)
Heart disease	8624 (16%)	15314 (18%)	5788 (21%)	1312 (22%)	410 (22%)
Smoking, %					
Current	6513 (12%)	7345 (8%)	2219 (8%)	427 (7%)	129 (7%)
Former	27129 (51%)	51742 (59%)	17719 (63%)	3818 (63%)	1203 (64%)
Never	18089 (34%)	27129 (51%)	7278 (26%)	1557 (26%)	484 (26%)
Missing	1735 (3%)	2948 (3%)	1018 (4%)	231 (4%)	62 (3%)
Mean (sd) alcohol consumption per day, g	18.24 (0.19)	18.36 (0.15)	18.01 (0.28)	16.41 (0.62)	11.72 (0.88)
Race, %					
Non-Hispanic White	50172 (94%)	81972 (94%)	26548 (94%)	5675 (94%)	1738 (93%)
Hispanic	843 (2%)	1775 (2%)	777 (3%)	171 (3%)	68 (4%)
Black	750 (1%)	1448 (2%)	454 (2%)	86 (1%)	28 (1%)
Asian, Pacific islander, American Indian, or Alaskan	1222 (2%)	1001 (1%)	176 (1%)	34 (1%)	15 (1%)
Missing	479 (1%)	771 (1%)	279 (1%)	67 (1%)	29 (2%)

For peer review only

Highest Education level achieved, %					
Less than 8 years old	2136 (4%)	4259 (5%)	1695 (6%)	422 (7%)	148 (8%)
Completed primary	6591 (12%)	13066 (15%)	4805 (17%)	1075 (18%)	356 (19%)
Completed high school	4643 (9%)	8406 (10%)	2930 (10%)	639 (11%)	209 (11%)
Post high school or come college	10728 (20%)	19668 (23%)	7120 (25%)	1540 (26%)	498 (27%)
College graduate or postgraduate	29368 (55%)	41568 (48%)	11684 (41%)	2357 (39%)	667 (36%)
Vigorous physical activity three or more times per week in the last year, %	41480 (78%)	64458 (74%)	18280 (65%)	3284 (54%)	786 (42%)
**Nutrition**					
At least 3 fruit servings per day, %	40079 (75%)	62921 (72%)	19870 (70%)	4203 (70%)	1303 (69%)
At least 3 vegetable servings per day, %	32887 (62%)	49213 (57%)	15003 (53%)	3220 (53%)	1037 (55%)
Mean (sd) red meat consumption, g/day	66.09 (0.28)	77.79 (0.21)	93.13 (0.44)	107.21 (1.09)	124.23 (2.72)
Mean (sd) calories per day, kcal	1997.3 (3.96)	2019.26 (3.08)	2123.92 (6.21)	2244.87 (14.7)	2411.64 (34.3)
Mean (sd) calories per day 10 years ago, kcal	861.51 (1.57)	865.75 (1.28)	896.59 (2.4)	936.47 (5.47)	974.51 (10.19)
Mean (sd) calories per day age 12, kcal	667.55 (1.63)	650.14 (1.28)	645.21 (2.27)	657.3 (4.93)	667.25 (9.06)
Drug use					
Ibuprofen use currently,	3969 (15%)	8036 (17%)	3688 (23%)	973 (27%)	408 (36%)
Missing	27029 (51%)	39942 (46%)	11894 (42%)	2460 (41%)	743 (40%)
Aspirin use currently,	16456 (40%)	29091 (43%)	10360 (47%)	2174 (47%)	699 (50%)
Missing	12646 (24%)	18622 (21%)	6146 (22%)	1444 (24%)	469 (25%)
Fibre					
Mean (sd) fibre from diet age 12, mg	9.65 (0.02)	9.69 (0.02)	9.84 (0.03)	10.04 (0.07)	10.3 (0.13)
Mean (sd) fibre from diet 10 years ago, mg	7.12 (0.01)	6.89 (0.01)	6.95 (0.02)	7.1 (0.05)	7.35 (0.09)
Mean (sd) fibre CSFII, g	20.95 (0.05)	20.11 (0.03)	20.26 (0.06)	20.85 (0.14)	22.3 (0.28)
Mean (sd) fibre total NDSR, g	21.35 (0.05)	20.49 (0.03)	20.66 (0.07)	21.22 (0.15)	22.65 (0.29)
Calcium					
Mean (sd) calcium supplement, mg	811.3 (2)	812.92 (1.62)	852.6 (3.12)	906.94 (7.45)	973.28 (16.08)
Mean (sd) calcium from diet current, mg	163.44 (1.18)	144.71 (0.85)	131.79 (1.43)	127.03 (3.03)	122.25 (5.34)
Mean (sd) calcium from diet 10 years ago, mg	406 (1)	398.45 (0.79)	404.71 (1.45)	422.22 (3.28)	426.44 (5.87)
Mean (sd) calcium from diet age 12, mg	667.55 (1.63)	650.14 (1.28)	645.21 (2.27)	657.3 (4.93)	667.25 (9.06)
Folate					

For peer review only

Mean (sd) folate supplement, g	342.65 (0.74)	332.76 (0.56)	335.07 (1.04)	343.98 (2.3)	360.39 (4.58)
Mean (sd) folate pre 1998, g	217.7 (1.01)	205.22 (0.8)	191.35 (1.41)	186.39 (3.01)	180.32 (5.43)
Mean (sd) folate NDSR, g	455.75 (0.92)	443.69 (0.69)	449.63 (1.3)	464.98 (2.95)	490.92 (5.98)

sd: standard deviation
N=875 men classified as <18.0 kg/m^2 at study entry not included

For peer review only - http://bmjopen.bmj.com/site/about/guidelines.xhtml

Table S8 Characteristics by BMI categories of 111,503 women in the AARP cohort

	Normal weight	Overweight	Obese I	Obese II	Obese III
Number of subjects, N (%)	48273 (43.3)	36025 (32.2)	16245 (14.6)	6126 (5.5.)	3507 (3.1)
Mean (sd) entry age, years	61.96 (0.02)	62.15 (0.03)	61.89 (0.04)	61.21 (0.07)	60.45 (0.09)
Mean (sd) current (at baseline) BMI, kg/m^2	22.45 (0.01)	27.23 (0.01)	32.12 (0.01)	37.14 (0.02)	44.66 (0.08)
Mean (sd) BMI at 50 years, kg/m^2	22.04 (0.01)	25.31 (0.01)	28.75 (0.03)	32.46 (0.06)	38.33 (0.12)
Mean (sd) BMI at 35 years, kg/m^2	21.12 (0.01)	22.93 (0.01)	24.93 (0.03)	27.22 (0.06)	30.72 (0.11)
Mean (sd) BMI at 18 years, kg/m^2	20.11 (0.01)	20.83 (0.01)	21.88 (0.03)	23.03 (0.05)	24.46 (0.08)
Mean (sd) maximum BMI, kg/m^2	24.21 (0.01)	28.88 (0.02)	34.05 (0.03)	39.21 (0.06)	46.57 (0.11)
Mean (sd) minimum BMI, kg/m^2	19.16 (0.01)	20.3 (0.01)	21.44 (0.02)	22.57 (0.05)	24.24 (0.08)
Mean (sd) waist circumference, cm	76.99 (0.04)	88.19 (0.05)	97.92 (0.08)	106.84 (0.13)	120.12 (0.23)
Mean (sd) hip circumference, cm	97.47 (0.03)	106.73 (0.04)	115.24 (0.07)	124.36 (0.14)	137.66 (0.24)
Mean (sd) hip-to-waist ratio,	0.79 (0)	0.83 (0)	0.85 (0)	0.86 (0)	0.85 (0)
Mean (sd) height, m	1218 (3%)	2199 (6%)	2050 (13%)	1211 (20%)	873 (25%)
Comorbidity, %					
Diabetes	1218 (3%)	2199 (6%)	2050 (13%)	1211 (20%)	873 (25%)
Heart disease	3050 (6%)	3043 (8%)	1749 (11%)	779 (13%)	482 (14%)
Smoking, %					
Current	7699 (16%)	4467 (12%)	1584 (10%)	502 (8%)	256 (7%)
Former	18632 (39%)	14883 (41%)	6843 (42%)	2633 (43%)	1586 (45%)
Never	20558 (43%)	15636 (43%)	7380 (45%)	2823 (46%)	1552 (44%)
Missing	1384 (3%)	1039 (3%)	438 (3%)	168 (3%)	113 (3%)
Mean (sd) alcohol consumption per day, g	7.94 (0.09)	5.92 (0.09)	4.3 (0.13)	3.25 (0.21)	2.67 (0.26)
Race, %					
Non-Hispanic White	45651 (95%)	33005 (92%)	14627 (90%)	5471 (89%)	3090 (88%)
Hispanic	988 (2%)	1688 (5%)	1067 (7%)	472 (8%)	299 (9%)
Black	582 (1%)	581 (2%)	225 (1%)	74 (1%)	45 (1%)
Asian, Pacific islander, American Indian, or Alaskan	620 (1%)	333 (1%)	114 (1%)	32 (1%)	16 (0%)
Missing	432 (1%)	418 (1%)	212 (1%)	77 (1%)	57 (2%)

For peer review only - http://bmjopen.bmj.com/site/about/guidelines.xhtml

Highest Education level achieved, %					
Less than 8 years old	1585 (3%)	1797 (5%)	1017 (6%)	417 (7%)	261 (7%)
Completed primary	10367 (21%)	9077 (25%)	4288 (26%)	1604 (26%)	954 (27%)
Completed high school	4914 (10%)	4138 (11%)	1869 (12%)	740 (12%)	459 (13%)
Post high school or come college	12696 (26%)	9375 (26%)	4327 (27%)	1644 (27%)	938 (27%)
College graduate or postgraduate	18711 (39%)	11638 (32%)	4744 (29%)	1721 (28%)	895 (26%)
Vigorous physical activity three or more times per week in the last year, %	34629 (72%)	23170 (64%)	8917 (55%)	2694 (44%)	1241 (35%)
**Nutrition**	37782 (78%)	27905 (77%)	12355 (76%)	4596 (75%)	2559 (73%)
At least 3 fruit servings per day, %	34422 (71%)	24747 (69%)	10907 (67%)	3921 (64%)	2257 (64%)
At least 3 vegetable servings per day, %					
Mean (sd) red meat consumption, g/day	40.35 (0.17)	47.73 (0.22)	54.09 (0.39)	61.25 (0.67)	71.18 (1.07)
Mean (sd) calories per day, kcal	1531.86 (2.99)	1568.13 (3.81)	1626.68 (6.33)	1696.9 (10.28)	1864.32 (16.6)
Mean (sd) calories per day 10years ago, kcal	818.42 (1.6)	859.29 (2.01)	894.97 (3.17)	910.64 (5.15)	962.24 (7.27)
Mean (sd) calories per day age 12, kcal	673.14 (1.77)	658.24 (2.05)	650.22 (3.07)	646.33 (5.04)	657.19 (6.67)
Hormone Therapy Use, %					
Never	18932 (39%)	16154 (45%)	8334 (51%)	3459 (56%)	2261 (64%)
Ever	29341 (61%)	19871 (55%)	7911 (49%)	2667 (44%)	1246 (36%)
Ibuprofen use currently,	5842 (21%)	5795 (26%)	3352 (33%)	1566 (39%)	1098 (47%)
Ibuprofen use missing	20318 (42%)	14129 (39%)	6018 (37%)	2075 (34%)	1191 (34%)
Aspirin use currently,	8875 (28%)	7709 (32%)	3714 (36%)	1445 (38%)	833 (41%)
Asprin use missing	16220 (34%)	12185 (34%)	5777 (36%)	2364 (39%)	1483 (42%)
,					
Fibre					
Mean (sd) fibre from diet 10years ago, mg	8.82 (0.02)	9.01 (0.02)	9.14 (0.04)	9.32 (0.06)	9.5 (0.08)
Mean (sd) fibre from diet age 12, mg	7.14 (0.02)	7.22 (0.02)	7.36 (0.03)	7.34 (0.05)	7.65 (0.07)
Mean (sd) fibre CSFII, g	18 (0.04)	17.74 (0.05)	17.94 (0.08)	18 (0.12)	19.35 (0.18)
Mean (sd) fibre total NDSR, g	18.05 (0.04)	17.77 (0.05)	17.98 (0.08)	18.05 (0.12)	19.43 (0.18)
Calcium					
Mean (sd) calcium supplement, mg	710.52 (1.9)	719.2 (2.26)	733.66 (3.61)	751.64 (5.75)	808.42 (8.73)
Mean (sd) calcium from diet current, mg	458.8 (2.18)	382.25 (2.35)	333.6 (3.31)	307.85 (5.3)	270.5 (6.54)
Mean (sd) calcium from diet 10 years ago, mg	382.09 (1.01)	388.3 (1.19)	397.37 (1.82)	397.86 (2.95)	414.1 (4.09)

For peer review only

For peer review only - http://bmjopen.bmj.com/site/about/guidelines.xhtml

Mean (sd) calcium from diet age 12, mg	673.14 (1.77)	658.24 (2.05)	650.22 (3.07)	646.33 (5.04)	657.19 (6.67)
Folate					
Mean (sd) folate supplement, g	250.24 (1.07)	239.5 (1.25)	225.57 (1.84)	217.96 (3.02)	209.48 (4.03)
Mean (sd) folate pre 1998, g	290.16 (0.68)	285.89 (0.78)	287.43 (1.2)	288.09 (1.98)	304.36 (2.79)
Mean (sd) folate NDSR, g	373.99 (0.82)	371.32 (0.96)	376.02 (1.49)	380.5 (2.43)	407.36 (3.5)

sd: standard deviation
N=1327 Women classified as <18.0 kg/m^2 at study entry not included

For peer review only

Figure S4 Latent Class trajectories derived on individuals with at least three time points, with random quadratic effect proportional specification (model F) (left: Men; right: Women)

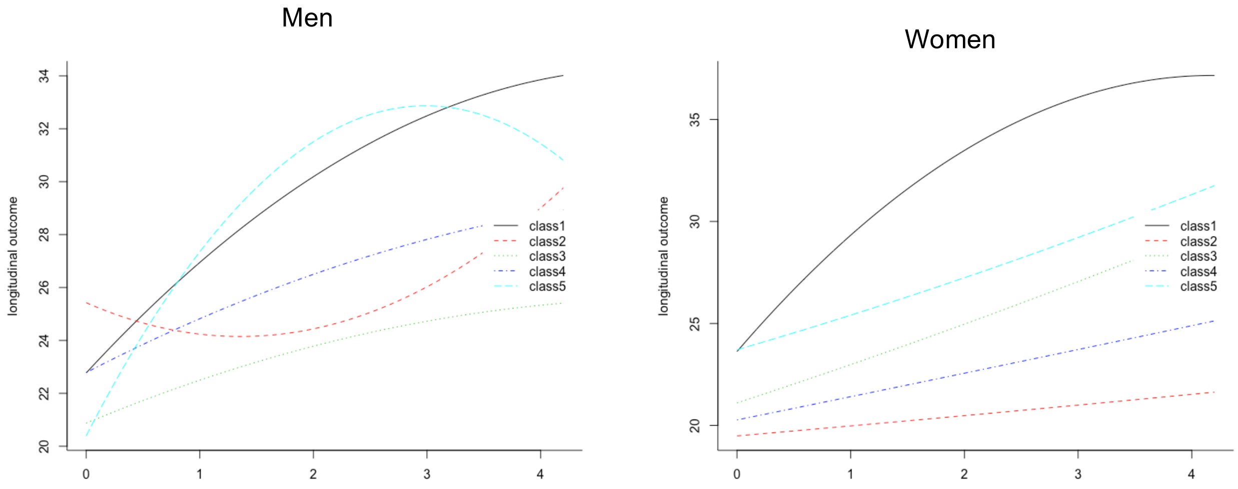

For peer review only

References

1. Kubzansky LD, Gilthorpe MS, Goodman E. A prospective study of psychological distress and weight status in adolescents/young adults. Annals of Behavioral Medicine 2012;43(2):219-228.

2. Kelly SP, Graubard BI, Andreotti G, Younes N, Cleary SD, Cook MB. Prediagnostic Body Mass Index Trajectories in Relation to Prostate Cancer Incidence and Mortality in the PLCO Cancer Screening Trial. J Natl Cancer Inst 2017;109(3).

3. Song M, Willett WC, Hu FB, Spiegelman D, Must A, Wu K, et al. Trajectory of body shape across the lifespan and cancer risk. Int J Cancer 2016;138(10):2383-95.

4. Song M, Hu FB, Wu K, Must A, Chan AT, Willett WC, et al. Trajectory of body shape in early and middle life and all cause and cause specific mortality: results from two prospective US cohort studies. BMJ 2016;353:i2195.

5. Zheng H, Tumin D, Qian Z. Obesity and Mortality Risk: New Findings From Body Mass Index Trajectories. American Journal of Epidemiology 2013;178(11):1591-1599.

6. Elsensohn M-H, Klich A, Ecochard R, Bastard M, Genolini C, Etard J-F, et al. A graphical method to assess distribution assumption in group-based trajectory models. Statistical methods in medical research 2016;25(2):968-982.

7. Vistisen D, Witte DR, Tabak AG, Herder C, Brunner EJ, Kivimaki M, et al. Patterns of obesity development before the diagnosis of type 2 diabetes: the Whitehall II cohort study. PLoS Med 2014;11(2):e1001602.

8. Lubke G, Neale MC. Distinguishing between latent classes and continuous factors: Resolution by maximum likelihood? Multivariate Behavioral Research 2006;41(4):499-532.

9. Peugh J, Fan X. How Well Does Growth Mixture Modeling Identify Heterogeneous Growth Trajectories? A Simulation Study Examining GMM's Performance Characteristics. Structural Equation Modeling: A Multidisciplinary Journal 2012;19(2):204-226.

10. Peugh J, Fan X. Enumeration Index Performance in Generalized Growth Mixture Models: A Monte Carlo Test of Muthén’s (2003) Hypothesis. Structural Equation Modeling: A Multidisciplinary Journal 2015;22(1):115-131.

11. Jedidi K, Ramaswamy V, Desarbo WS. A maximum likelihood method for latent class regression involving a censored dependent variable. Psychometrika 1993;58(3):375-394.

For peer review only - http://bmjopen.bmj.com/site/about/guidelines.xhtml

BMJ Open

A framework to construct and interpret latent class trajectory modelling

Journal:	*BMJ Open*
Manuscript ID	bmjopen-2017-020683.R2
Article Type:	Research
Date Submitted by the Author:	15-Mar-2018
Complete List of Authors:	Lennon, Hannah; University of Manchester, Division of Cancer Sciences; University of Manchester Institute of Population Health<br>Kelly, Scott; National Institutes of Health, Division of Cancer Epidemiology and Genetics, National Cancer Institute<br>Sperrin, Matthew; University of Manchester Institute of Population Health, Division of Informatics, Imaging and Data Sciences<br>Buchan, Iain; University of Manchester Institute of Population Health, Division of Informatics, Imaging and Data Sciences<br>Cross, Amanda; Imperial College London School of Public Health, Department of Epidemiology and Biostatistics<br>Leitzmann, Michael; Universitat Regensburg Fakultat fur Medizin, Department for Epidemiology and Preventive Medicine<br>Cook , Michael ; National Institutes of Health, Division of Cancer Epidemiology and Genetics, National Cancer Institute<br>Renehan, Andrew; University of Manchester, Division of Cancer Sciences; University of Manchester Institute of Population Health, Manchester Cancer Research Centre, NIHR Manchester Biochemical Research Centre
**Primary Subject Heading**:	Research methods
Secondary Subject Heading:	Health informatics, Epidemiology, Medical education and training, Communication
Keywords:	Latent class models, Growth curves, Growth mixture models, Lifetime obesity, Trajectories

SCHOLARONE™
Manuscripts

Original article (formatted for BMJ Open)

A framework to construct and interpret latent class trajectory modelling

Hannah Lennon,[1,3] Scott Kelly,[2] Matthew Sperrin,[3] Iain Buchan,[3] Amanda J Cross,[4] Michael Leitzmann,[5] Michael B Cook,[2] Andrew G Renehan[1,3,6]

[1] Division of Cancer Sciences, School of Medical Sciences, Faculty of Biology, Medicine and Health, University of Manchester, Manchester, UK

[2] Division of Cancer Epidemiology and Genetics, National Cancer Institute, National Institutes of Health, Maryland, US

[3] MRC Health eResearch Centre (HeRC), Division of Informatics, Imaging and Data Sciences, University of Manchester, Manchester, UK

[4] Department of Epidemiology and Biostatistics, Imperial College, London, UK

[5] Department of Epidemiology and Preventive Medicine, University of Regensburg, Regensburg, Germany

[6] Manchester Cancer Research Centre, NIHR Manchester Biochemical Research Centre, University of Manchester, Manchester, UK

For peer review only

Running title: *latent class modelling*

Keywords*: Latent class models, growth curves, growth mixture models, body mass index, trajectories, lifetime obesity*

Correspondence to:

Hannah Lennon
Manchester Cancer Research Centre
NIHR Manchester Biomedical Research Centre
Division of Cancer Sciences, School of Medical Sciences,
Faculty of Biology, Medicine and Health,
University of Manchester,
Manchester,
M13 9PL UK
Tel: +44 161 306 7925
E-mail: hannah.lennon@manchester.ac.uk

Abstract: 274 words; main text: 3365 words; 5 tables; 2 figures; References 30 (max: 30); Supplemental material (18 pages); language: UK English.

For peer review only - http://bmjopen.bmj.com/site/about/guidelines.xhtml

Abstract

Objectives: Latent class trajectory modelling (LCTM) is a relatively new methodology in epidemiology to describe life-course exposures, which simplifies heterogeneous populations into homogeneous patterns or classes. However, for a given dataset, it is possible to derive scores of different models based on number of classes, model structure, and trajectory property. Here, we rationalise a systematic framework to derive a 'core' favoured model.

Methods: We developed an eight step framework: step 1, a scoping model; step 2, refining the number of classes; step 3, refining model structure (from fixed-effects through to a flexible random-effect specification); step 4, model adequacy assessment; step 5, graphical presentations; step 6, use of additional discrimination tools ('degree of separation'; Elsensohn's envelope of residual plots); step 7, clinical characterisation and plausibility; and step 8, sensitivity analysis. We illustrated these steps using data from the NIH-AARP cohort of repeated determinations of body mass index (BMI) at baseline (mean age: 62.5 years), and BMI derived by weight recall at ages 18, 35 and 50 years.

Results: From 288,993 participants, we derived a 5-class model for each gender (men: 177,455; women: 111,538). From seven model structures, the favoured model was a proportional random quadratic structure (model F). Favourable properties were also noted for the unrestricted random quadratic structure (model G). However, class proportions varied considerably by model structure – concordance between Models F and G were moderate (Cohen κ: men, 0.57; women, 0.65) but poor with other models. Model adequacy assessments, evaluations using discrimination tools, clinical plausibility, and sensitivity analyses supported our model selection.

Conclusion: We propose a framework to construct and select a 'core' LCTM, which will facilitate generalisability of results in future studies.

Funding: Cancer Research UK National Awareness and Early Detection Initiative (NAEDI), Award Number A17962.

For peer review only - http://bmjopen.bmj.com/site/about/guidelines.xhtml

STRENGTHS AND LIMITATIONS OF THIS STUDY

- We developed a systematic approach, with rationale for each of eight steps, to derive a latent class trajectory model of favoured number of classes and ‘core’ model structure specification
- The results presented here are based on modelling data from a large well-characterised US cohort; allowing the derivation of numerically meaningful subpopulations (i.e., classes) with distinct phenotypes
- Compared with ‘one-off’ BMI categorisation, latent class trajectory modelling offers additional phenotypic information and opportunities to identify and intervene early in sub-populations with adverse trajectories
- Whilst we described multiple diagnostic tests, ultimately model selection was based on case-study appropriate model interpretation (for example, model adequacy; discrimination; clinical plausibility; sensitivity analyses) by a multi-disciplinary research team

For peer review only

INTRODUCTION

In many epidemiological studies, a risk factor is measured at a single point in time and related to the subsequent development of disease, under the assumption that a single 'one-off' measure is an approximation for that exposure over a long time. Thus, baseline measurement of body mass index (BMI) is associated with subsequent development of common disease like cardiovascular disease (1), diabetes (2), several cancers (3), and all-cause mortality (4). This approach is crude and many investigators seek to use alternative methods that might better capture long-term risk factor exposure termed *life-course analysis*. There are widely-used examples that capture cumulative exposure, such as pack-years for smoking and lung cancer, but the assumption that incidence rate is proportional to total lifetime dose is questionable (5). Many other life-course models simply extract features for use in standard regression approaches; for example, a weight change over time. A more sophisticated approach, which takes account of within-individual correlations, is *mixed-effect modelling*, but this is difficult to interpret for public health implementation. An extension of this approach is the use of *latent classes*, also termed growth mixture models.

Latent class trajectory modelling (LCTM) simplifies heterogeneous populations into more homogeneous clusters or classes. From these, one can potentially include random effects to allow for individual variation within these classes. These models have a long history in the criminology (6) and psychology (7) literatures, and now, are increasingly reported in the human epidemiology literature (for example, disentangling the heterogeneity of childhood asthma (8)). Of relevance to this paper, LCTM has been used in association studies of repeated BMI measures with the following endpoints: all-cause mortality (9); cancer incidence (multiple cancer types (10)); gastro-oesophageal (11); prostate (12)); and cancer mortality (12). The LCTM has three general advantages compared with using 'one-off' exposure determinations: first, it better informs aetiological associations by deeply phenotyping certain 'at risk' subpopulations; and second, LCTM offers a public health strategy to identify early divergent adverse trajectories as potential intervention targets. Some researchers additionally argue that LCTM is well-equipped for future forecasting and

For peer review only - http://bmjopen.bmj.com/site/about/guidelines.xhtml

new-patient generalisations in prediction models, as it handles data following a different predictable pattern from that learnt by the model (13). Thirdly, the trajectory approach allows a better understanding of the causes of between-individual variation in certain features (e.g., weight variation over age), by analysing the trajectory as an outcome rather than exposure.

However, LCTM is a complex form of modelling and requires several different structure assumptions (14). Although firmly acknowledged in the GRoLTS-Checklist: Guidelines for Reporting on Latent Trajectory Studies (15), structure-related assumptions have not been systematically evaluated. For many exposures of interest, typically two to seven classes might be described and, as detailed latter, at least seven model structures might be fitted, with and without linear curve properties, such that it is possible to derive greater than eighty different models. Thus, reported differences between studies using latent class modelling might reflect different modelling assumptions rather than true differences between populations. To facilitate the generalizability of results in future studies, here, we propose a framework to construct and select a 'core' LCTM, using an example of repeatedly determined body mass index (BMI) across adulthood in the National Institutes of Health (NIH)-AARP Diet and Health Study cohort. For exposure-disease outcome association analyses, current approaches generally use two stages: first, latent class trajectory modelling, followed by standard association modelling. The framework described here is limited to the first stage.

METHODS

Cohort

The NIH-AARP Diet and Health Study is a US cohort recruited from 1995 (16). A baseline medical and lifestyle questionnaire, including self-reported weight and height, was returned by 566,398 participants (aged 50 to 71 years; mean age: 62.5 years). An additional risk factor questionnaire was mailed in 1996 and completed by a sub-cohort of 327,860; of whom 288,993 (177,455 men and 111,538 women) provided recall weight for all four time-points -

For peer review only - http://bmjopen.bmj.com/site/about/guidelines.xhtml

ages 18, 35 and 50 years. Derived BMIs at baseline and these ages (assuming constant height) form the data in the present analysis. We excluded participants with extreme BMI values (<15 or >70 kg/m^2) recorded at any time point. Means and standard deviations for derived recalled BMI distributions are representative of BMI distributions for historical period-equivalent US populations (17).

Latent class trajectory modelling

We developed an eight step framework **(Table 1)** modelling BMI as a function of age. Latent classes were used to identify subgroups of participants with distinct trajectories (detailed mathematical equations in supplemental material p2) (18). We used maximum likelihood approaches to fit the model with the '*hlme'* function from 'lcmm' library (19) in the R software environment (version 3.2.1) and cross-checked results using the '*PROC TRAJ'* function in 'SAS traj' (SAS Institute, Inc., Cary, NC) (20) (Table S1).

Step 1: We initially constructed a scoping model provisionally selecting the plausible number of classes based on available literature – in the context of BMI trajectories, we used K = 5 classes as reported elsewhere (10, 12). We built models for both genders, as BMI patterns of lifetime changes differ for men and women (21). To determine the initial working model structure, we followed the rationale of Verbeke and Molenburgh (22) and examined the shape of standardised residual plots for each of the five classes in a model with no random-effects. If the residual profile could be approximated by a flat, straight line or a curve, then a random intercept, slope or quadratic term, respectively, were considered. Preliminary plots suggested preference for a quadratic random-effects model (Figure S1).

Step 2: We refined the preliminary working model from step 1 to determine the optimal number of classes, testing K = 1 to 7. The number of classes chosen was based on the lowest Bayesian Information Criteria (BIC).

For peer review only - http://bmjopen.bmj.com/site/about/guidelines.xhtml

Step 3: We further refined the model, using the favoured *K* derived in step 2, testing for the optimal model structure. We tested seven models (detailed in Table S2): ranging from a simple fixed-effects model (model A) through a rudimentary method that allows the residual variances to vary between classes (model B) to a suite of five random-effects models with different variance structures (models C-G).

Step 4: We then performed a number of model adequacy assessments. First, for each participant, we calculated the posterior probability of being assigned to each trajectory class, and assigned the individual to the class with the highest probability. An average of these maximum Posterior Probability of Assignments (APPA) above 70%, in all classes, is regarded as acceptable (6). We further assessed model adequacy using Odds of Correct Classification (OCC), mismatch scores and Entropy, E_k (detailed in Table S3). These diagnostic tools assist in model selection (6, 23). In some examples, BIC values may decrease as more groups and parameters are added reflecting model overfit. Therefore, the BIC value might not always provide the optimum selection criteria, and model selection must balance between meaningful trajectories, model parsimony and model adequacy. For example, if the model adequacy measures are strongly violated, one might go back to steps 2 and 3 and consider a different model with a higher BIC value. We selected an optimal model structure using the lowest BIC value and satisfactory values from the model adequacy assessments and referred to the outcome of steps 1-4 as the *favoured model*. To assess the interpretability of the resulting classes, we investigated characteristics of lifestyle behaviours of the favoured model such as smoking, alcohol consumption and physical activity.

Step 5: We used three graphical presentation approaches. The conventional approach is to plot mean trajectories with time encompassing each class. Alternatives include the use of mean trajectory plots with 95% predictive intervals for each class, which displays the predicted random variation within each class; or to plot individual level 'spaghetti plots' with

For peer review only - http://bmjopen.bmj.com/site/about/guidelines.xhtml

time (for example, a random sample of participants), which allows the reader to observe the patterns of changes within classes.

Step 6: We assessed model discrimination, including Degrees of Separation, DoS_K (24, 25) and Elsensohn's envelope of residuals (26). To describe the separation of latent trajectory curves, a multivariate Mahalanobis distance was used. Peugh and Fan (25) argue that it is reasonable to speculate that identification of heterogeneous latent trajectories is facilitated by large statistical separation distance among the subpopulations. Thus, larger values of DoS_K indicate the mean trajectories are well separated while DoS_K equal to zero is the special case when all mean trajectories are identical. If the DoS_K value is small, then one might consider a model with fewer classes.

To check structure assumptions in fixed-effects latent class models, Elsenhohn et al. (26) plotted the local standard deviations of the residuals against time. We extended this method to random-effects models; first, computing the observed residuals for each participant; and second, computing the class- and time-specific weighted local variance of the residuals, with weights being the posterior probabilities of individual belonging to a class. We plotted the upper and lower boundary values of the local standard deviations of the residuals around the mean values for each class. The resulting shape indicates the appropriateness of the model assumptions; where non-parallel boundaries indicate heteroscedasticity of residuals suggesting poor model fit, and differing interval widths suggest that across class variability may not be fully accounted for.

Step 7: We assessed for clinical characterisation and plausibility using four approaches: i) assessing the clinical meaningfulness of the trajectory patterns, aiming to include classes with at least 1% capture of the population; ii) assessing the clinical plausibility of the trajectory classes; iii) tabulation of characteristics by latent classes versus conventional categorisations; and iv) concordance of class membership with conventional BMI category

For peer review only - http://bmjopen.bmj.com/site/about/guidelines.xhtml

membership using the kappa statistic (as LCTM is an unsupervised learning approach, we computed κ for all possible combinations and selected the optimal κ).

Step 8: We conducted sensitivity analyses, in this example, with individuals with at least two and three BMI values, as LCTMs are flexible enough to deal with different observation times between participants.

Statistical algorithms

All R and SAS codes used to implement these tools are available via the authors and can be downloaded from *www.github.com/hlennon/LCTMtools*.

RESULTS

Number of classes

From the preliminary working model of a quadratic random-effects model, model F (proportional covariance structure), we derived BICs for up to seven classes: three of the class models failed to converge in men and women. **Table 2** reports that the lowest BIC was obtained with five classes in men and women – confirming our initial working model. The proportions by class in men were 68.1%, 25.0%, 3.8%, 2.7%, and 0.4%; and in women, were 32.6%, 41.1%, 21.1%, 3.5% and 1.7%. For model G (our second favoured model), the lowest BICs were noted for five classes in men and women (Table S2).

Assessment for model structures

With the number of classes now selected as five, we tested the seven model structures – A to G. **Table 3** reports that the lowest BIC was for model F in men and women, justifying the selection of model F in the preliminary working phase. The class sizes varied between models, with Class I ranging from 41% to 68% in men and from 32% to 95% in women. The APPA for each class in model F was 0.81, 0.74, 0.87, 0.83, and 0.74 in men and 0.74, 0.79, 0.80, 0.83 and 0.84 in women, indicating a good discrimination of trajectory. The classes

For peer review only

were well differentiated with the relative Entropy, E_K values ranging from 0.59 to 0.81 in men and 0.66 to 0.83 in women.

There was moderately good concordance (unweighted and weighted) between the unstructured variance models G with model F in men (κ: 0.57) and women (κ: 0.65) (Tables S4 and S5), but poorer concordance between the favoured models and fixed-effects models in men.

Graphical presentation

We plotted the mean trajectories for model A, B, C, D, F and G in men and women **(Figure 1)** illustrating the increased complexity from model A to G. As alternatives, we plotted separately mean trajectories with 95% predictive intervals for each class, in model F (Figure S2), which displays the predicted random variation within each of the classes with time, noting that variation was greater with the more 'complex' classes (classes IV and V compared with classes I, II and III). Spaghetti plots of individual level data illustrated that the timing and size of BMI changes characterise the classes – for example, sharp increases in BMI in early adulthood in Class III but later in adulthood for Class IV (Figure S3).

Additional tools of suitability of fit

The DoS_k values ranged from 0.10 to 0.36 and 0 to 0.34, in men and women, respectively **(Table 3)**. The covariances were high and in the positive direction and therefore models with non-parallel mean trajectories lead to higher separation.

We plotted the local standard deviation of the residuals with time and found that these were broadly homogeneous, i.e., there were few parallel boundaries **(Figure 2)**. The local residuals for the rapidly obese groups in both genders are the exceptions to parallel lines, which might reflect comorbidities in this group and smaller numbers.

Clinical assessment

For peer review only - http://bmjopen.bmj.com/site/about/guidelines.xhtml

Having established the favoured model, model F with five classes in both genders, we assigned descriptive labels to each respective class as follows **(Table 4)**: stable normal weight; normal weight to overweight; normal weight to obese; overweight to obese; and rapid early obesity. We noted that the proportion in the rapid early obesity (Class V) was less than 1% in men. However, overall, the proportion for Class V for men and women combined was nearly 1%. Thus, we retained this class as we judged it to be clinically meaningful as follows. In both genders, there were rapid increases in obesity from early to middle adulthood, then apparent severe weight reductions. We rationalised that this was clinically plausible, as it could be explained either by intentional (e.g. bariatric surgery) or non-intentional weight loss (e.g. reverse causality from development of disease).

We then tabulated the baseline characteristics according to the five classes for model F in men and women, and noted patterns across the classes **(Table 4** – and fully expanded in Tables S5 and S6). Thus, for example, for current smoking status, there were little differences in patterns by class in men: 10%, 8%, 9%, 10%, and 15%; and in women, 16%, 12%, 12%, 13% and 11%. This contrasts with BMI categories—men (12%, 8%, 8%, 7% and 7%) and women (16%, 12%, 10%, 8%, and 7%) (Tables S7 and S8).

Finally, we noted very poor concordance between the favoured model and conventional BMI categorisation in men (κ: 0.18) and women (κ: 0.52) **(Table 5)**.

Sensitivity analyses

We tested the favoured model using a larger sample of individuals with at least three measures, and found no material differences between these models, in men and women, and the main model (Figure S4).

DISCUSSION

Main findings

We propose an eight-step framework for the construction and selection of models derived from latent class trajectory modelling. We evaluated a range of model structures from fixed-

For peer review only - http://bmjopen.bmj.com/site/about/guidelines.xhtml

effect models to a set of random-effects models, favouring the latter models in this case study, as they include different variance structures, and more likely to reflect the natural history of changes with time in BMI distributions in different sub-populations. We showed that different model structures resulted in different classes with contrasting clinical phenotypes. We propose pre-specified criteria for model selection and that the reporting of a 'core' model will facilitate generalizability of results in future studies.

Context of other literature

To the best of our knowledge, this is the first study to systematically address structure-related assumptions in LCTMs, and their potential impact on clinically-relevant endpoints – in this example, BMI trajectories. Anecdotally, there is a justifiable criticism regarding the use of LCTM models and an uncertainty of how class memberships are derived – a 'black box' effect. The proposed framework, here, encourages the opposite – a transparent stepwise approach to class and model structure selection. To enhance this process, for example, we have 'borrowed' tools developed to address to quantify uncertainty, such as entropy measures, E and E_k, and applied them to assist assessment of model adequacy. A further modification of discrimination measurement with variance estimation has been described by Shah and colleagues (27), and might have importance for class assignment where 'yes/no' treatment decisions are required.

Variations of model A (fixed-effects) have been reported in the clinical literature (9-12), which assume no within-class variability when deriving latent classes. Interpretation in this setting is that variation from the mean trajectory is random, i.e., the correlation between measurements for the same individual is explained by latent class membership. In the context of any repeated measures in the general population, this assumption might not be valid (14). Saunders (28) argued in support of full random-effects models (i.e. models F and G), calling upon Moffitt's theory from criminology, which recognises that "*there are distinct developmental clusters of trajectories of anti-social behaviour that are the result of divergent aetiologies*" – in other words, it is unlikely that latent classes start from a similar baseline.

For peer review only - http://bmjopen.bmj.com/site/about/guidelines.xhtml

The publication of the 16-item GRoLTS Guidelines in 2017 (15) heralded an important advance for the application of LCTM. Here, we add a framework for construction and interpretation.

Strengths and weaknesses

The study has strengths. First, the considered and strategic workflow to optimise identification and application of latent classes provides for a more robust and transparent application of these models in epidemiology. Second, the results presented are based on modelling data from a large well-characterised US cohort, therefore allowing the derivation of numerically meaningful subpopulations (i.e. classes) with distinct phenotypes. We uniquely used averaged kappa values to demonstrate that the LCTM-derived subpopulations are markedly different to those derived from a 'one-off' BMI determinations. In turn, BMI trajectories are more likely to reflect normal clinical practice of considering a 'weight history'. Third, we extensively explored different model selections and adequacy tools, and described extensions to other tools, to supplement model interpretation. Fifth, we further supplement model interpretation, we embedded this project within a multi-disciplinary research team including data scientists, statisticians, clinicians and epidemiologists – an approach echoed elsewhere (29). Finally, we have made the statistical algorithms freely available.

There are several study weaknesses. First, LCTMs currently only considers trajectories of one risk factor at a time. Second, there were only four time points in the AARP such that it was not possible to assess weight cycling. Third, whilst we described multiple diagnostic tests, ultimately model selection was based on case-study appropriate model interpretation (for example, model adequacy; discrimination; clinical plausibility; sensitivity analyses) as well as likelihood-based model-fit criteria (18). Some discussion on statistical power and efficiency is warranted. The objective of model selection is a trade-off between efficiency and validation with the aim of summarising distinct features of the data as parsimonious as possible, and not just the maximisation of model fits (6). For example, in an hypothetical scenario, putting emphasis on the efficiency of a model in which ten classes

For peer review only - http://bmjopen.bmj.com/site/about/guidelines.xhtml

provides the most statistically efficient model is questioned if three of the ten classes each includes less than 0.5% of the population, and without markedly different characteristics.

Clinical implications and future research

We showed that different model structures resulted in different classes with contrasting clinical phenotypes. Thus, for example, it is well-recognised that the proportions of current smokers decreases with increasing BMI categories. However, this ‘trend’ is not observed across the latent classes derived in our favoured model F, suggesting that the clinical characteristics derived from the LCTM differed from those derived from conventional categorisation approaches. Thus, compared with ‘one-off’ BMI categorisation, latent class trajectory modelling offers additional phenotypic information.

For future research, improving the construction, interpretation and reporting of LCTM (advocated here) is hugely important as the LCTM approach has opportunities to identify and intervene early in sub-populations with adverse trajectories. This approach is analogous to the well-held public strategy of using childhood growth charts to identify and intervening in young children failing to thrive. Thus, in the example of BMI, remembering that 80% of obese adults were not obese in childhood (30), future LCTM studies might identify (new) individuals in their 20s or early 30s on adverse trajectories towards later adulthood obesity. This strategy is a new methodological paradigm, as the repeated measurement of a risk factor (here, BMI) becomes a clinically-relevant endpoint rather than just an exposure.

For peer review only - http://bmjopen.bmj.com/site/about/guidelines.xhtml

Role of the funding source

The funders of the study had no role in study design, data collection, data analysis, data interpretation, or writing of the report.

Conflict of Interest

AGR has received lecture honoraria from Merck Serona and Janssen-Cilag, and independent research funding from Novo Nordisk. All other authors have no conflicts of interest to declare.

Contributions

HL, MS, MC and AGR conceptualised the paper. HL, MS and SK designed the statistical approaches; HL performed the modelling. AC and ML facilitated data access and interpretation of the AARP data. All authors contributed to data interpretation; IB and AGR put modelling into clinical context.

Patient and Public Involvement

No patients and or public were involved with this manuscript.

Acknowledgements

We acknowledge the generous funding from Cancer Research UK National Awareness and Early Detection Initiative (NAEDI).

Data sharing statement

All statistical codes used in this paper are available via the authors and can be downloaded from *www.github.com/hlennon/LCTMtools*. Supplementary materials are available from the Dryad repository, DOI: [include DOI here].

For peer review only - http://bmjopen.bmj.com/site/about/guidelines.xhtml

For peer review only

REFERENCES

1. Wormser D, Kaptoge S, Di Angelantonio E, Wood AM, Pennells L, Thompson A, et al. Separate and combined associations of body-mass index and abdominal adiposity with cardiovascular disease: collaborative analysis of 58 prospective studies. Lancet 2011;377(9771):1085-95.

2. Vazquez G, Duval S, Jacobs DR, Jr., Silventoinen K. Comparison of body mass index, waist circumference, and waist/hip ratio in predicting incident diabetes: a meta-analysis. Epidemiol Rev 2007;29:115-28.

3. Lauby-Secretan B, Scoccianti C, Loomis D, Grosse Y, Bianchini F, Straif K. Body Fatness and Cancer--Viewpoint of the IARC Working Group. Colditz G, Anderson AS, Herbert RA, Kaaks R, Thompson HJ, Baker JL, Breda J, Byers T, Cleary MP, Di Cesare M, Gapstur SM, Gunter M, Hursting SD, Leitzmann M, Ligibel J, Renehan A, Romieu I, Shimokawa I, Ulrich CM, Wade K, Weiderpass E. N Engl J Med 2016;375(8):794-8.

4. Global BMI Mortality Collaboration. Body-mass index and all-cause mortality: individual-participant-data meta-analysis of 239 prospective studies in four continents. Lancet 2016.

5. Peto J. That the effects of smoking should be measured in pack-years: misconceptions 4. Br J Cancer 2012;107(3):406-7.

6. Nagin D. Group-based modeling of development: Harvard University Press; 2005.

7. Stevenson J, Thompson MJ, Sonuga-Barke E. Mental health of preschool children and their mothers in a mixed urban/rural population. III. Latent variable models. Br J Psychiatry 1996;168(1):26-32.

8. Belgrave DC, Buchan I, Bishop C, Lowe L, Simpson A, Custovic A. Trajectories of lung function during childhood. Am J Respir Crit Care Med 2014;189(9):1101-9.

9. Song M, Hu FB, Wu K, Must A, Chan AT, Willett WC, et al. Trajectory of body shape in early and middle life and all cause and cause specific mortality: results from two prospective US cohort studies. BMJ 2016;353:i2195.

10. Song M, Willett WC, Hu FB, Spiegelman D, Must A, Wu K, et al. Trajectory of body shape across the lifespan and cancer risk. Int J Cancer 2016;138(10):2383-95.

11. Petrick JL, Kelly SP, Liao LM, Freedman ND, Graubard BI, Cook MB. Body weight trajectories and risk of oesophageal and gastric cardia adenocarcinomas: a pooled analysis of NIH-AARP and PLCO Studies. Br J Cancer 2017;116(7):951-959.

12. Kelly SP, Graubard BI, Andreotti G, Younes N, Cleary SD, Cook MB. Prediagnostic Body Mass Index Trajectories in Relation to Prostate Cancer Incidence and Mortality in the PLCO Cancer Screening Trial. J Natl Cancer Inst 2017;109(3).

13. Qian T, Masino AJ. Latent Patient Cluster Discovery for Robust Future Forecasting and New-Patient Generalization. PLoS One 2016;11(9):e0162812.

14. Gilthorpe MS, Dahly DL, Tu YK, Kubzansky LD, Goodman E. Challenges in modelling the random structure correctly in growth mixture models and the impact this has on model mixtures. J Dev Orig Health Dis 2014;5(3):197-205.

For peer review only - http://bmjopen.bmj.com/site/about/guidelines.xhtml

15. van de Schoot R, Sijbrandij M, Winter SD, Depaoli S, Vermunt JK. The GRoLTS-Checklist: Guidelines for Reporting on Latent Trajectory Studies. . Structural Equation Modeling: A Multidisciplinary Journal;24:451-467.

16. Schatzkin A, Subar AF, Thompson FE, Harlan LC, Tangrea J, Hollenbeck AR, et al. Design and serendipity in establishing a large cohort with wide dietary intake distributions : the National Institutes of Health-American Association of Retired Persons Diet and Health Study. Am J Epidemiol 2001;154(12):1119-25.

17. Renehan AG, Flood A, Adams KF, Olden M, Hollenbeck AR, Cross AJ, et al. Body mass index at different adult ages, weight change, and colorectal cancer risk in the National Institutes of Health-AARP Cohort. American journal of epidemiology 2012;176(12):1130-1140.

18. Tu Y-K, Gilthorpe MS. Statistical thinking in epidemiology: CRC Press; 2011.

19. Proust-Lima C, Philipps V, Diakite A, Liquet B. Package 'lcmm' Title Extended Mixed Models Using Latent Classes and Latent Processes. In; 2016.

20. Jones BL, Nagin DS. Advances in group-based trajectory modeling and an SAS procedure for estimating them. Sociological Methods & Research 2007;35(542-571).

21. Sperrin M, Marshall AD, Higgins V, Buchan IE, Renehan AG. Slowing down of adult body mass index trend increases in England: a latent class analysis of cross-sectional surveys (1992-2010). Int J Obes (Lond) 2014;38(6):818-24.

22. Verbeke G, Molenberghs G. Linear Mixed Models for Longitudinal Data. 2000.

23. Klijn SL, Weijenberg MP, Lemmens P, van den Brandt PA, Lima Passos V. Introducing the fit-criteria assessment plot - A visualisation tool to assist class enumeration in group-based trajectory modelling. Stat Methods Med Res 2015.

24. Lubke G, Neale MC. Distinguishing between latent classes and continuous factors: Resolution by maximum likelihood? Multivariate Behavioral Research 2006;41(4):499-532.

25. Peugh J, Fan X. How Well Does Growth Mixture Modeling Identify Heterogeneous Growth Trajectories? A Simulation Study Examining GMM's Performance Characteristics. Structural Equation Modeling: A Multidisciplinary Journal 2012;19(2):204-226.

26. Elsensohn M-H, Klich A, Ecochard R, Bastard M, Genolini C, Etard J-F, et al. A graphical method to assess distribution assumption in group-based trajectory models. Statistical methods in medical research 2016;25(2):968-982.

27. Shah NH, Hipwell AE, Stepp SD, Chang CC. Measures of discrimination for latent group-based trajectory models. J Appl Stat 2015;42(1):1-11.

28. Saunders JM. Understanding Random Effects in Group-Based Trajectory Modeling: An Application of Moffitt's Developmental Taxonomy. J Drug Issues 2010;40(1):195-220.

29. Belgrave D, Henderson J, Simpson A, Buchan I, Bishop C, Custovic A. Disaggregating asthma: Big investigation versus big data. J Allergy Clin Immunol 2017;139(2):400-407.

30. Simmonds M, Burch J, Llewellyn A, Griffiths C, Yang H, Owen C, et al. The use of measures of obesity in childhood for predicting obesity and the development of obesity-

For peer review only

For peer review only - http://bmjopen.bmj.com/site/about/guidelines.xhtml

related diseases in adulthood: a systematic review and meta-analysis. Health Technol Assess 2015;19(43):1-336.

For peer review only

For peer review only - http://bmjopen.bmj.com/site/about/guidelines.xhtml

Table 1 Framework of eight steps to construct a latent class trajectory model

Step	Step description	Criteria for selection
1	Scope model by provisionally selecting a plausible number of classes based on available literature and the structure based on plausible clinical patterns	Examine linearity of the shape of standardised residual plots for each of the classes in a model with no random-effects
2	Refine the model from step 1 to confirm the optimal number of classes, typically testing K = 1 to 7 classes	Lowest Bayesian Information Criteria (BIC) value
3	Refine optimal model structure from fixed- through to unrestricted random-effects of the model using the favoured K derived in step 2	
4	Run model adequacy assessments as described in Table S3 including Posterior Probability of Assignments (APPA); Odds of Correct Classification (OCC); and relative entropy	i) APPA: average of maximum probabilities should be greater than 70% for all classes<br>ii) OCC values greater than 5.0<br>iii) Relative entropy values greater than 0.5
5	Investigate graphical presentation	i) Plot mean trajectories across time for each class in a single graph<br>ii) Plot mean trajectories with 95% predictive intervals for each class (one class per graph)<br>iii) Plot individual class 'spaghetti plots' across time for a random sample
6	Run additional tools to assess discrimination including Degrees of Separation and Elsensohn's envelope of residuals	i) DoS greater than zero<br>ii) Envelope of residuals is assessed in plots by observing clear separations between classes
7	Assess for clinical characterisation and plausibility	i) Tabulation of characteristics by latent classes. Are the trajectory patterns clinically meaningful? Perhaps, consider classes with a minimum percentage of the population.<br>ii) Are the trajectory patterns clinically plausible?<br>iii) Concordance of class characteristics with those for other well-established variables
8	Conduct sensitivity analyses – for example, testing models without complete data at all time points	General assessment of patterns of trajectories compared with main model

For peer review only - http://bmjopen.bmj.com/site/about/guidelines.xhtml

Table 2 Number of classes (*K* = 1 to 7) using random-effects quadratic structure model F (proportional covariance structure) by gender

Model	*K*	Number of parameters	BIC	Proportions per class %						
				Class I	Class II	Class III	Class IV	Class V	Class VI	Class VII
**MEN**										
**Model F**	1	10	∗	100						
	2	15	3324009	83	17					
	3	20	3310908	62	32	3				
	4	25	3324128	100	0	0	0			
	5	30	**3301301**	68	25	4	3	0.4		
	6	35	∗							
	7	40	∗							
**WOMEN**										
**Model F**	1	10	∗	100						
	2	15	2195386	86	14					
	3	20	2179080	58	34	8				
	4	25	2179137	100	0	0	0			
	5	30	**2169791**	41	32	21	4	2		
	6	35	∗							
	7	40	∗							

∗ Models failed to converge
BIC: Bayesian Information Criteria
Results from random-effects quadratic structure Model G (unrestricted) by gender are shown in supplemental material TS2.

For peer review only

Table 3 Model adequacy assessments of latent trajectory class models based on different assumptions for *K* = 5 classes, by gender in the NIH-AARP cohort

Model	Description	BIC	Proportion per class %	Average Posterior Probability Assignment (APPA)	Relative Entropy E_k	Degree of Separation DoS_k
**Men**						
A	Homoscedastic	3476322	51: 22: 21: 6: 1	83: 84: 85: 91: 95	0.78	0.10
B	Heteroscedastic	3511646	41: 26: 20: 7: 6	85: 82: 84: 89: 86	-	0.15
C	Random intercept	3364856	68: 24: 4: 3: 2	90: 82: 89: 85: 82	0.81	0.34
D	Random slope	3325463	63: 19: 13: 3: 4	72: 70: 74: 83: 83	0.59	0.26
E	Random quadratic, Equal	*				
F	Random quadratic, Proportional	**3301301**	68: 25: 4: 3: 0.4	81: 74: 87: 83: 74	0.68	0.36
G	Random quadratic, Unrestricted	3320005	56: 30: 8: 5: 0.4	74: 70: 80: 79: 88	0.63	0.33
**Women**						
A	Homoscedastic	2289509	45: 36: 13: 4: 1	90: 84: 88: 93: 96	0.83	0.14
B	Heteroscedastic	2238444	32: 30: 15: 15: 8	87: 83: 85: 89: 91	-	0.05
C	Random intercept	2240681	52: 35: 9: 2: 2	89: 82: 86: 89: 91	0.79	0.21
D	Random slope	2193155	79: 12: 5: 2: 2	91: 80: 79: 86: 91	0.82	0.34
E	Random quadratic, Equal	2188224	95: 5: 0: 0: 0	49: 88: 0: 0: 0	0.26	0.05
F	Random quadratic, Proportional	**2169793**	41: 33: 21: 3: 2	74: 79: 80: 83:84	0.66	0.09
G	Random quadratic, Unrestricted	2187707	67: 23: 6: 3: 1	84: 77: 84:82: 87	0.73	0.34

*Failed to converge

For peer review only

For peer review only - http://bmjopen.bmj.com/site/about/guidelines.xhtml

Table 4 Latent class characteristics of 177,453† men and 111,503† women in the NIH-AARP cohort

	Model F (favoured model)				
	**Class I** Stable normal weight	**Class II** Normal weight to overweight	**Class III** Normal weight to obese	**Class IV** Overweight to obese	**Class V** Rapid increase to obese
**MEN**					
Number of participants, N	120 867 (68.1%)	44 383 (25.0%)	6 723 (3.8%)	4 764 (2.7%)	718 (0.4%)
Mean (sd) BMI at 18 years	20.75 (0.01)	23.92 (0.02)	21.69 (0.04)	27.16 (0.09)	24.76 (0.2)
Mean (sd) BMI at 35 years	23.35 (0.01)	26.72 (0.01)	30.24 (0.04)	26.61 (0.09)	37.69 (0.26)
Mean (sd) BMI at 50 years	24.66 (0.01)	29.12 (0.02)	31.34 (0.05)	30.95 (0.12)	35.01 (0.35)
Mean (sd) entry age, years	62.88 (0.01)	61.59 (0.03)	62.03 (0.06)	59.71 (0.07)	57.73 (0.18)
Mean (sd) current (at baseline) BMI, kg/m^2	25.39 (0.01)	30.84 (0.02)	29.07 (0.05)	35.14 (0.12)	30.51 (0.28)
Mean (sd) waist circumference, cm	94.38 (0.02)	106.23 (0.05)	102.73 (0.13)	115.24 (0.28)	105.91 (0.64)
Smoking, %					
Current	11 823 (10%)	3 761 (8%)	638 (9%)	460 (10%)	106 (15%)
Former	67 948 (56%)	26 898 (61%)	3 809 (57%)	3 002 (63%)	370 (52%)
Never	37 149 (31%)	12 130 (27%)	2 012 (30%)	1 110 (23%)	218 (30%)
Missing	3 946 (3%)	1 594 (4%)	264 (4%)	191 (4%)	24 (3%)
Mean (sd) alcohol consumption per day, g	18.69 (0.13)	17.45 (0.21)	15.48 (0.56)	15.57 (0.69)	10.22 (1.14)
**WOMEN**					
Number of participants, N	36 311 (32.6%)	45 832 (41.1%)	23 544 (21.1%)	3 898 (3.5%)	1 918 (1.7%)
Mean (sd) entry age, years	62.48 (0.03)	62.23 (0.02)	61.07 (0.03)	59.61 (0.09)	59.69 (0.12)
Mean (sd) BMI at 18 years	19.3 (0.01)	20.44 (0.01)	22.61 (0.02)	29.01 (0.09)	23.97 (0.1)
Mean (sd) BMI at 35 years	20.26 (0.01)	22.53 (0.01)	25.51 (0.02)	28.38 (0.1)	36.92 (0.16)
Mean (sd) BMI at 50 years	21.16 (0.01)	24.77 (0.01)	29.52 (0.03)	33.51 (0.13)	37.69 (0.2)
Mean (sd) current (at baseline) BMI, kg/m^2	21.97 (0.01)	26.66 (0.01)	32.4 (0.03)	37.47 (0.15)	34.4 (0.19)
Mean (sd) waist circumference, cm	76.19 (0.04)	86.66 (0.05)	97.82 (0.09)	106.29 (0.32)	101.96 (0.41)
Smoking, %					
Current	5803 (16%)	5660 (12%)	2733 (12%)	495 (13%)	214 (11%)
Former	14173 (39%)	18557 (40%)	9748 (41%)	1764 (45%)	731 (38%)
Never	15313 (42%)	20290 (44%)	10417 (44%)	1507 (39%)	909 (47%)
Missing	1022 (3%)	1325 (3%)	646 (3%)	132 (3%)	64 (3%)
Mean (sd) alcohol consumption per day, g	8.07 (0.1)	6.26 (0.08)	4.47 (0.11)	3.92 (0.29)	3.4 (0.48)

For peer review only - http://bmjopen.bmj.com/site/about/guidelines.xhtml

Hormone Therapy Use, %					
Ever	13989 (39%)	20206 (44%)	12215 (52%)	2241 (57%)	1092 (57%)
Never	22322 (61%)	25626 (56%)	11329 (48%)	1657 (43%)	826 (43%)

sd: standard deviation; †Exclusions include 35 women and 2 men with biologically implausible cancers from classes in proportions (2,16,12,0,5) and (1,0,0,1,0) respectively.

For peer review only

For peer review only - http://bmjopen.bmj.com/site/about/guidelines.xhtml

Table 5 Concurrence between BMI categories and classes in model F from the NIH-AARP cohort

Latent class	N	BMI categories (kg/m^2) < 18.5	18.5 to 24.9	25.0 to 29.9	30.0 to 34.9	>35.0
			**Men**			
	177453	875	53466	86967	28234	7911
I	120866	312	50171	68646	1736	1
II	44383	319	1860	14097	23472	4635
III	6723	47	859	3335	1943	539
IV	4763	172	437	679	917	2558
V	718	25	139	210	166	178
			**Cohen κ_ω = 0.182 (0.181,0.183)**			
			**Women**			
	111503	1327	48273	36025	16245	9633
I	36311	1215	33371	1725	0	0
II	45832	3	12584	28636	4607	2
III	23544	83	1683	4777	10587	6414
IV	3898	14	382	519	614	2369
V	1918	12	253	368	437	848
			**Cohen κ_ω= 0.52 (0.51,0.53)**			

BMI: body mass index; κ_ω weighted

For peer review only

FIGURE LEGENDS

Figure 1 BMI mean trajectories by men (left) and women (right) for models A-G. Colours are used to discriminate classes within each plot, but should not be used for direct comparisons across plots.

Figure 2 Illustration of the local (Elsensohn) residual envelope plots (shown here for model B)

For peer review only

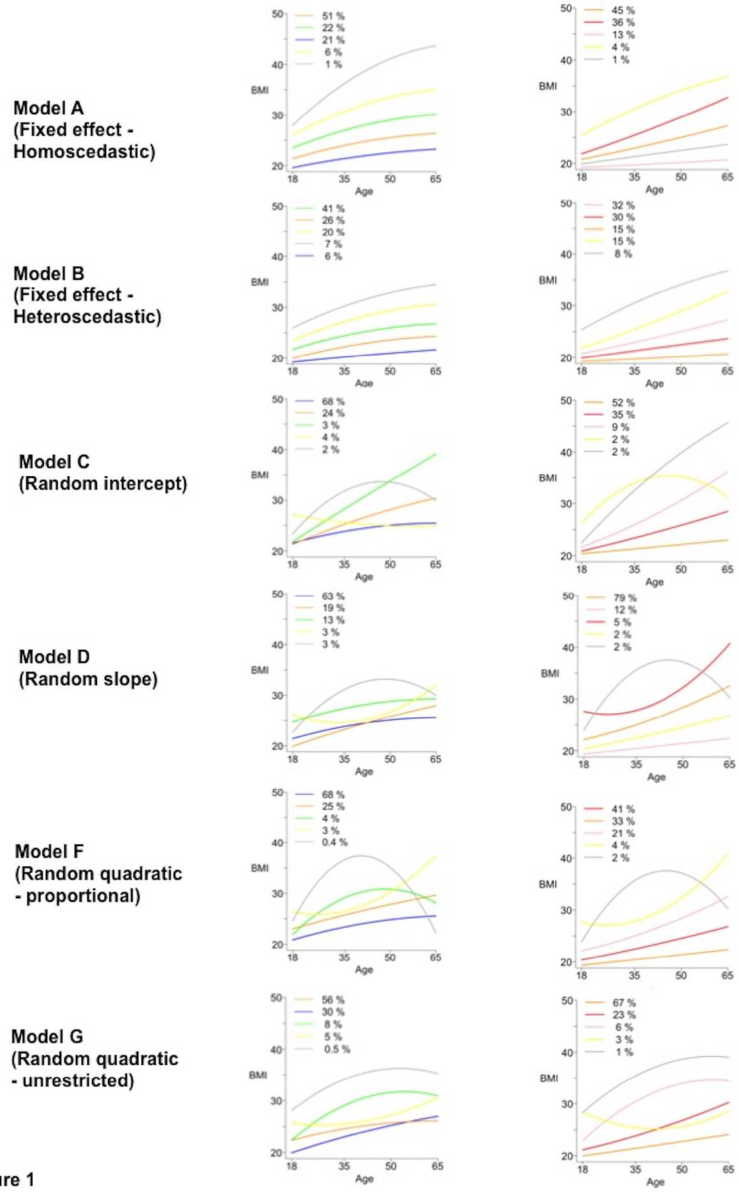

Figure 1

BMI mean trajectories by men (left) and women (right) for models A-G. Colours are used to discriminate classes within each plot, but should not be used for direct comparisons across plots.

254x366mm (300 x 300 DPI)

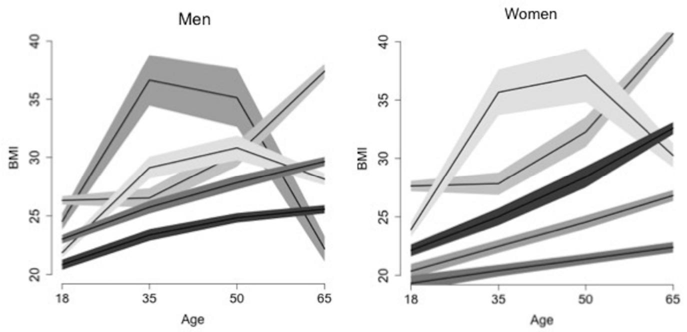

Figure 2

Illustration of the local (Elsensohn) residual envelope plots

254x366mm (300 x 300 DPI)

SUPPLEMENTAL MATERIAL

A framework to construct and interpret latent class trajectory modelling

Hannah Lennon[1,3] Scott Kelly,[2] Matthew Sperrin,[3] Iain Buchan,[3] Amanda J Cross,[4] Michael Leitzmann,[5] Michael B Cook,[2] Andrew G Renehan[1,3, 6]

For peer review only

Table S1 Different models with increasing complexity, associated software and referenced use in literature

Model	Description	Interpretation	Software *Command*	References
A	Fixed effects homoscedastic (common residual variance across classes)	No random effects – with the interpretation that any deviation of an individuals trajectory from its mean class trajectory is due to random error only	SAS traj *PROC TRAJ*	(1-5)
B	Fixed effects heteroscedastic (class-specific residual variances)	The same interpretation as Model A with random errors that can be larger and smaller in different classes.	R mmlcr *mmlcr*	(6)
C	Random intercept	The interpretation is allowing individuals to vary in initial weight but each class member is assumed to follow the same shape and magnitude of the mean trajectory	SAS traj *PROC TRAJ*	(7)
D	Random slope	Allowing individuals to vary in initial weight and slope of the mean trajectory but same curvature as trajectory	SAS traj *PROC TRAJ*	(7)
E	Random quadratic – Common variance structure across classes	Additional freedom of allowing individuals to vary within classes by initial weight, shape and magnitude, however each class is assumed to have the same amount of variability	R lcmm *hlme/lcmm*	
F	Random quadratic – Proportionality constraint to allow variance structures to vary across classes	Increasing flexibility of model E as variance structures are allowed to differ up to a multiplicative factor to allow some classes to have larger or smaller within-class variances. This model is can be thought of more parsimonious version of model G from (reducing the number of variance-covariance parameters to be estimated from 6xK parameters to 6+(K-1) parameters.	R lcmm *hlme/lcmm*	
G	Random quadratic – Class-specific variance structure (unstructured)	The most flexible model in which each class has its own separate random quadratic variance structure to describe its own within-class variability. Statistically this permits the variance and covariance of the intercept, slope and quadratic term to vary freely across all classes.	SAS traj *PROC TRAJ*	

[1]The SAS traj package has been converted for Stata users as the traj command in Stata (Collage Station, TX, USA).

For peer review only - http://bmjopen.bmj.com/site/about/guidelines.xhtml

More detailed description of model structure

For N individuals, the latent class trajectory model we consider is given by:

$$BMI_{itk} = \beta_0^k + \beta_1^k AGE_t + \beta_2^k AGE_t^2 + b_0^k + b_1^k AGE_t + b_2^k AGE_t^2 + \epsilon_t, \qquad \text{(Equation 1)}$$

where BMI_{itk} is the BMI of individual $i = 1, \dots, N$, at time $t = 1, \dots, T$ in class $k = 1, \dots, K$. The random effect b^k is class-specific and follows a multivariate Normal distribution with zero mean and a 3×3 variance-covariance matrix B. For the residual error term ϵ_t, the usual assumption hold, ϵ_t is normally distributed with zero mean and variance σ^2. The probability of an individual belonging to class k is described by a multinomial distribution, i.e.,

$$Pr(individual\ i\ belonging\ to\ class\ k) = \frac{e^{\pi_k}}{\sum_{k=1}^{K} e^{\pi_k}} = \frac{e^{\pi_k}}{e^{\pi_1} + \cdots + e^{\pi_K}}, \qquad \text{(Equation 2)}$$

such that π_k are parameters to be estimated in the model. To select the number of latent classes, we assume a working model (Equation 1) for the random effect structure and the criterion used to select the number of classes was the lowest Bayesian Information Criteria (BIC).

More detailed description of model adequacy assessment

Common tools for model adequacy assessment checking are described below in Table S2. Here we give more details of the two extensions of these tools; degree of separation and Elsensohn's residuals (to random effects).

Degrees of separation

A model's ability to detect classes accurately is affected by the degree of separation between latent trajectory curves (8, 9). To describe the separation of latent growth curves, we used the multivariate Mahalanobis distance with the multivariate Mahalanobis distance (D) units defined as:

$$D_{ij} = \left(\mu_i - \mu_j\right)^T \Sigma^{-1} \left(\mu_i - \mu_j\right), \qquad i,j = 1, \dots K,$$

For peer review only - http://bmjopen.bmj.com/site/about/guidelines.xhtml

where μ_i is a $T{\times}1$ vector of mean values for class i, and Σ^{-1} is the inverse of a $T \times T$ matrix of sample covariances of at times $t = 1, \dots, T$. The larger the difference, the larger the separation between curves. Peugh and Fan (9) argue that it is reasonable to expect that it is easier to identify heterogeneous latent growth trajectories when the statistical separation distance among the subpopulations is larger than when the separation distance among the latent subpopulations is much smaller.

To give an overall measure of separation for each model, we propose a weighted sum of multivariate Mahalanobis distance matrix with weights being the estimated class proportions, $\hat{\pi}_\iota$.. Then the degree of separation DoS_K is defined as

$$DoS_K = \sum_{i=1}^{K} \sum_{j=1}^{i} \hat{\pi}_\iota \hat{\pi}_J D_{ij}\,, \quad i, j = 1,2, \dots, K.$$

Larger values of DoS_K indicate the mean trajectories are well separated while DoS_K is zero in the special case when all mean trajectories are identical. If the DoS_K value is small, then you may wish to consider a model with fewer classes.

Elsensohn's envelope of residuals

To check the model assumption in fixed effect latent class models, Elsenhohn et al. (6) suggest plotting the local standard deviations of the residuals to check the appropriateness of the model. With the assumption that the residuals are homogeneous over time. To check the appropriateness of each of our model assumptions, we extended their method to include random effects in the models. We compute the local standard deviations of the residuals using the following steps:

1) Compute the observed residuals r_{itk} for each subject i, at time t given the individual is in class k

Equation 1: $r_{itk} = y_{it} - \hat{y}_{itk} = y_{it} - x_i^T \beta^k - z_i^T b^k,$

For peer review only - http://bmjopen.bmj.com/site/about/guidelines.xhtml

where y_{itk} is the observed value for individual i at time t in class k and $\hat{y}_{itk}$ is the fitted value of BMI from our fitted model, here the random effect model. 2) Compute the class- and time-specific weighted local variance of the residuals, $Var_w[r_{itk}]$, with weights being p_{ik}, the posterior probabilities of individual i belonging to group k. #) Plot the upper and lower boundaries for the local standard deviations of the residuals

Equation 2: $b_{itk} = \mu_{tk} \pm \sqrt{Var_w[r_{itk}]}$,

$where\ \mu_{tk}$ is the mean value of class k at time t. 4) Plot the boundaries b_{itk} onto the mean trajectory plots.

The shape of the local standard deviation of the residuals indicates the appropriateness of the model assumptions; where non-parallel boundaries indicate heteroscedasticity of residuals suggesting poor model fit, and differing interval widths suggest across group variability may not be fully accounted for. We believe this complements the others metrics well. If the boundaries suggest a poor fit, you may consider a more complex random effect structure to be more suitable, for example, a higher order polynomial.

For peer review only

Figure S1 Standardised residual plots of fixed-effect model (model A) for each class justifying the selection of a random-effect structure in the scoping model

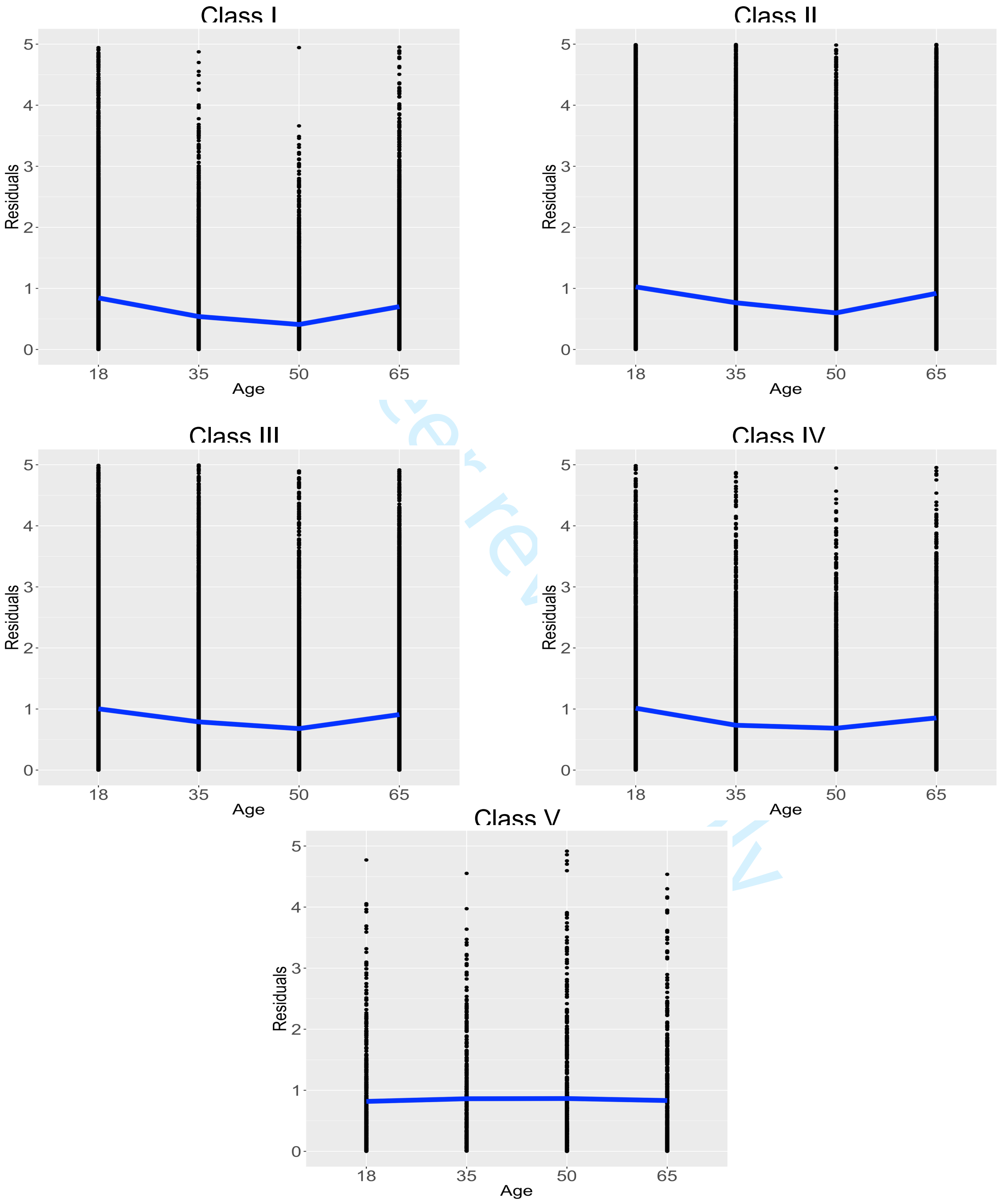

For peer review only - http://bmjopen.bmj.com/site/about/guidelines.xhtml

Table S2 Number of classes (K = 1† to 7) using random quadratic effect models: model G (unrestricted) by gender, NIH-AARP cohort

Model	K	Num of parameters	BIC	Proportions per class (%)						
				Class I	Class II	Class III	Class IV	Class V	Class VI	Class VII
**MEN**										
**Model G**	2	14	3361853	94	6					
	3	21	3341066	87	7	7				
	4	28	3329426	52	37	7	5			
	5	35	**3320005**	51	33	9	6	1		
	6	42	3315240	45	31	12	9	2	1	
	7	48	3311326	51	19	13	7	5	3	0.4
**WOMEN**										
**Model G**	2	14	2219825	89	11					
	3	21	2203726	61	34	0.5				
	4	28	2194293	61	32	5	2			
	5	35	**2187707**	60	29	7	3	1		
	6	42	2184412	63	19	10	4	1	0.5	
	7	48	*							

⁎ Models failed to converge, † Models with K=1 failed to converge

For peer review only

Table S3 Common model selection tools for latent class trajectory models

Tool	Criteria for a good model	Description
Likelihood based measures	Lowest BIC, AIC,	These likelihood based model selection tools include: Bayesian Information Criteria (BIC), Akaike Information Criteria (AIC), Likelihood Ratio Tests (LRT), Lo-Mendel-Rubin (LMT-LRT), Bootstrap-LRT. (See Peugh and Fan, 2015 for discussion) (10)
APPA	APPA > 70% for each class	The average Posterior Probability Assignment, $\bar{p}_k$, looks at whether individuals are assigned with high probability and the overall average probability of assignment to each class.
Odds of correct classification	OCC > 5.0 for each class	The odds of correct classification (OCC) is the ratio of the odds of a correct classification into each group on the basis of the maximum probability classification rule and the estimated class membership proportions, $\widehat{\pi_k}$, $$OCC_k = \frac{\bar{p}_k/(1-\bar{p}_k)}{\widehat{\pi_k}/(1-\widehat{\pi_k})}.$$
Mismatch	Close to 0 for each class	The difference between the estimated class proportions and the class membership proportions once individuals have been assigned to a class, i.e. $$Mismatch_k = \widehat{\pi_k} - \frac{N_k}{N},$$ where N_k is the number of individuals in a class and N is the total number.
Entropy	Close to 0	Entropy is a global measure of classification uncertainty, which takes into account all $N \times K$ posterior probabilities. The entropy of a model is defined as $$E = -\sum_{i=1}^{N}\sum_{k=1}^{K} \hat{p}_{ik} \log \hat{p}_{ik},$$ which takes values from $[0, \infty)$, with higher values indicating a larger amount of uncertainty. Entropy values closest to 0 correspond to models with least classification uncertainty.
Relative Entropy	Close to 1	Relative entropy is a standardised version of entropy in the interval $[0,1]$ defined by $$E_K = 1 - \frac{E}{N \log K},$$ where values close to 1 indicate lowest classification uncertainty. In the special case when there is most uncertainty and each individual has equal probability of belonging to each class, $E_K = 0$. Jedidi et al., describes relative entropy as a relative measure of 'fuzziness', and suggested cause concern when close to zero, as this implies that the latent class centroids are not sufficiently separated (11).

For peer review only

Table S4 Concurrence between fixed effect (Model A) and random quadratic effect LCTM models (Model F and G) in men and women in the NIH-AARP cohort

Models	Cohen's κ_w (95% CI)	Concurrence						
**MEN**					Class			
**Model A and F**	0.31		Model F	I	II	II	IV	V
	(0.27,0.34)	Model A	177453	91106	38437	36430	10118	1362
		Class I	120866	81148	4961	34757	0	0
		Class II	44383	7826	28617	1035	6785	120
		Class III	6723	1405	3611	78	1491	138
		Class IV	4763	619	1076	553	1607	908
		Class V	718	108	172	7	235	196
			Model F	I	II	II	IV	V
		Model G	177453	99897	53372	14978	8320	886
**Model F and G**	0.57	Class I	120866	77962	41688	1117	99	0
	(0.56,0.59)	Class II	44383	21684	11464	6433	4796	6
		Class III	6723	219	10	6389	1	104
		Class IV	4763	17	210	746	3402	388
		Class V	718	15	0	293	22	388
**WOMEN**								
**Model A and F**	0.80		Model F	I	II	II	IV	V
	(0.80,0.80)	Model A	111503	50712	40080	14784	4840	1087
		Class I	36311	36311	0	0	0	0
		Class II	45832	13060	32383	389	0	0
		Class III	23544	1066	6881	13008	2589	0
		Class IV	3898	245	588	969	1501	595
		Class V	1918	30	228	418	750	492
**Model F and G**	0.65		Model F	I	II	II	IV	V
	(0.65,0.65)	Model G	111503	74481	25824	6929	3133	1136
		Class I	36311	36299	9	3	0	0
		Class II	45832	37334	8298	200	0	0
		Class III	23544	840	16896	4320	1463	25
		Class IV	3898	5	617	1098	1658	520
		Class V	1918	3	4	1308	12	591

For peer review only

Table S5 Kappa Matrices† between Models A to G in men and women in the NIH-AARP cohort

**Models**		**Cohen's Kappa Values (95% CI)**				
**MEN**		Model				
**Unweighted, κ**						
		Model B	Model C	Model D	Model F	Model G
	Model A	0.64 (0.64, 0.64)	0.20 (0.20, 0.21)	0.20 (0.19, 0.20)	0.38 (0.38, 0.38)	0.13 (0.12, 0.13)
	Model B		0.20 (0.20, 0.20)	0.19 (0.19, 0.19)	0.32 (0.31, 0.32)	0.16 (0.16, 0.17)
	Model C			0.59 (0.58, 0.59)	0.48 (0.47, 0.48)	0.43 (0.42, 0.43)
	Model D				0.50 (0.50, 0.50)	0.49 (0.48, 0.49)
	Model F					0.18 (0.18, 0.19)
**Weighted, κ_w**						
		Model B	Model C	Model D	Model F	Model G
	Model A	0.36 (0.34, 0.38)	0.43 (0.41, 0.44)	0.37 (0.34, 0.41)	0.31 (0.27, 0.34)	0.38 (0.36, 0.39)
	Model B		0.43 (0.42, 0.44)	0.39 (0.37, 0.41)	0.43 (0.42, 0.44)	0.39 (0.38, 0.4)
	Model C			0.49 (0.45, 0.52)	0.65 (0.65, 0.65)	0.62 (0.62, 0.62)
	Model D				0.71 (0.71, 0.71)	0.67 (0.67, 0.67)
	Model F					0.57 (0.56, 0.59)
**WOMEN**						
**Unweighted, κ**						
		Model B	Model C	Model D	Model F	Model G
	Model A	0.64 (0.64, 0.64)	0.65 (0.65, 0.66)	0.25 (0.24, 0.25)	0.63 (0.63, 0.63)	0.37 (0.37, 0.38)
	Model B		0.45 (0.44, 0.45)	0.17 (0.17, 0.17)	0.48 (0.47, 0.48)	0.30 (0.29, 0.30)
	Model C			0.26 (0.25, 0.26)	0.49 (0.49, 0.5)	0.41 (0.41, 0.41)
	Model D				0.28 (0.27, 0.28)	0.52 (0.52, 0.53)
	Model F					0.32 (0.31, 0.32)
**Weighted, κ_w**						
		Model B	Model C	Model D	Model F	Model G
	Model A	0.78 (0.78, 0.78)	0.69 (0.69, 0.69)	0.57 (0.57, 0.57)	0.80 (0.80, 0.80)	0.65 (0.65, 0.65)
	Model B		0.48 (0.48, 0.49)	0.42 (0.41, 0.42)	0.68 (0.68, 0.68)	0.45 (0.45, 0.46)
	Model C			0.64 (0.64, 0.64)	0.71 (0.71, 0.71)	0.63 (0.63, 0.63)
	Model D				0.64 (0.63, 0.66)	0.81 (0.81, 0.81)
	Model F					0.65 (0.65, 0.65)

† Kappa values presented here are optimised over the (5!=120) possible combinations of matching the classes labels across the models

For peer review only

Figure S2 Mean trajectories and 95% prediction intervals for each class in model F – men (left) and women (right)

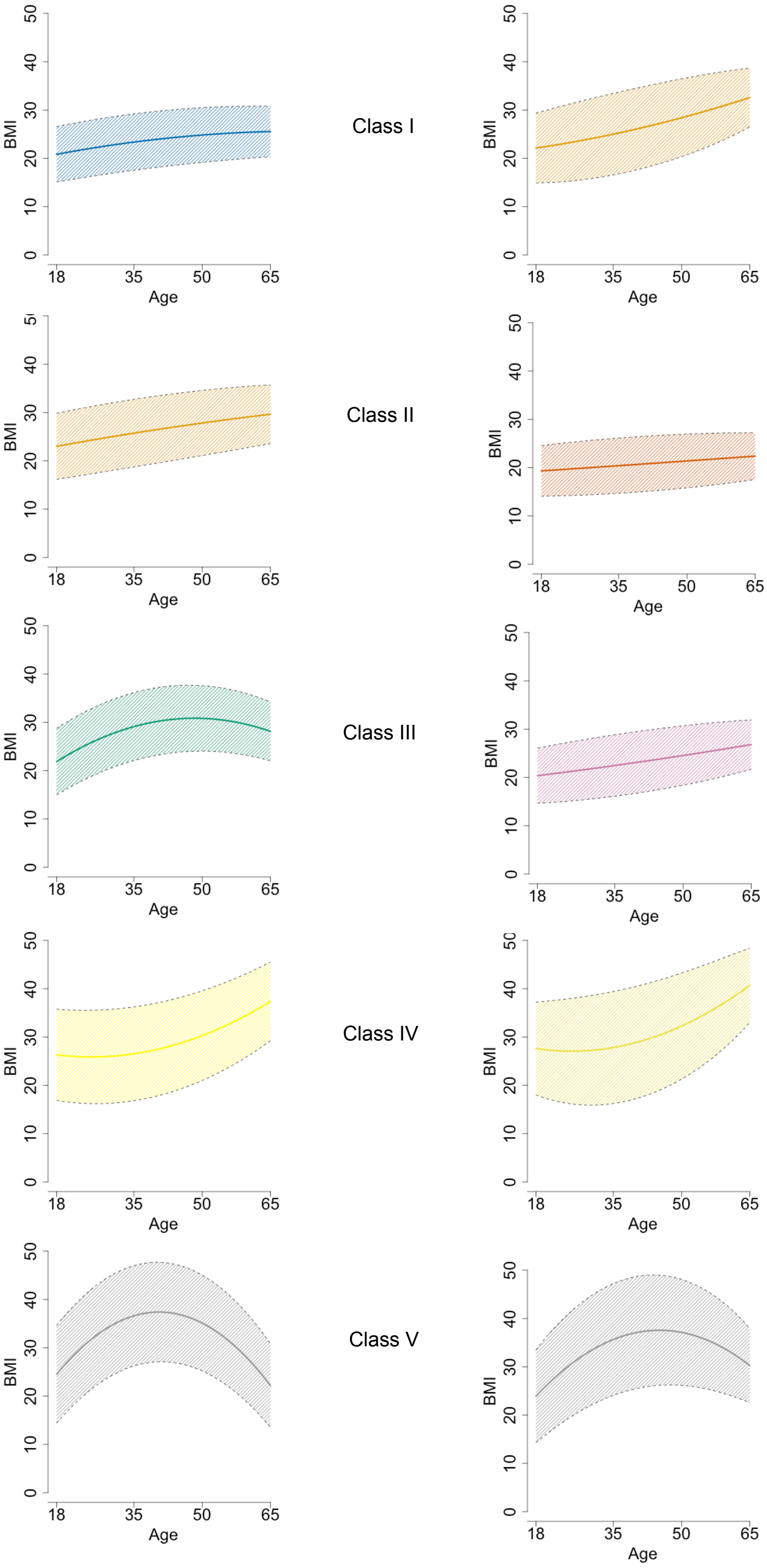

Figure S3 A randomly selected sample of 250 men from each class in model F

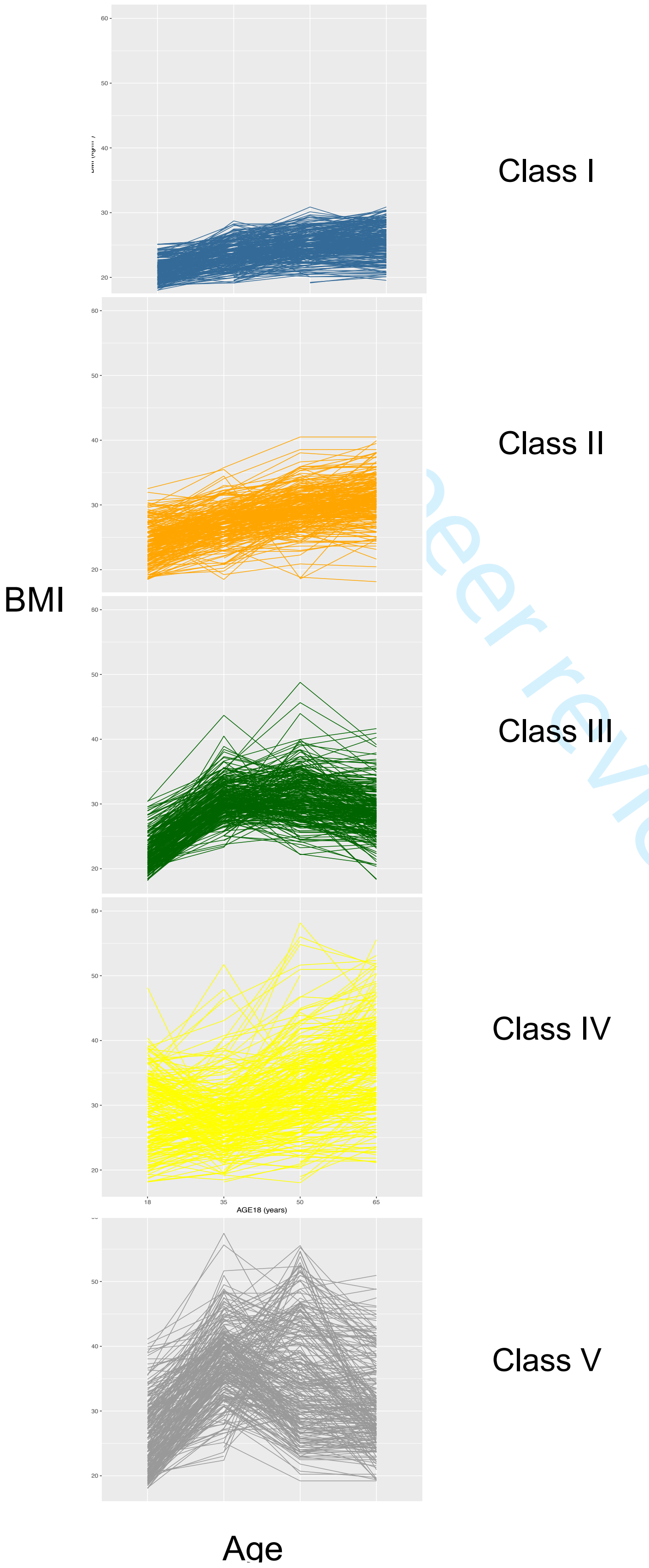

For peer review only

Table S5 Characteristics by five latent classes (Model F) of 177,453 men in the NIH-AARP cohort

	Model F (preferred model)				
	**Class I**	**Class II**	**Class III**	**Class IV**	**Class V**
	Stable normal weight	Normal weight to overweight	Normal to stable obese	Overweight to obese	Rapid increase to obese
Number of subjects, N	120 867 (68%)	44 383 (32%)	6 723 (5%)	4 764 (3%)	718 (0.5%)
Mean (sd) entry age, years	62.88 (0.01)	61.59 (0.03)	62.03 (0.06)	59.71 (0.07)	57.73 (0.18)
Mean (sd) current (at baseline) BMI, kg/m^2	25.39 (0.01)	30.84 (0.02)	29.07 (0.05)	35.14 (0.12)	30.51 (0.28)
Mean (sd) BMI at 50 years, kg/m^2	24.66 (0.01)	29.12 (0.02)	31.34 (0.05)	30.95 (0.12)	35.01 (0.35)
Mean (sd) BMI at 35 years, kg/m^2	23.35 (0.01)	26.72 (0.01)	30.24 (0.04)	26.61 (0.09)	37.69 (0.26)
Mean (sd) BMI at 18 years, kg/m^2	20.75 (0.01)	23.92 (0.02)	21.69 (0.04)	27.16 (0.09)	24.76 (0.2)
Mean (sd) maximum BMI, kg/m^2	26.94 (0.01)	32.76 (0.02)	34.09 (0.05)	37.04 (0.13)	40.17 (0.33)
Mean (sd) minimum BMI, kg/m^2	20.56 (0.01)	23.16 (0.02)	22.46 (0.05)	23.15 (0.07)	24.42 (0.20)
Mean (sd) waist circumference, cm	94.38 (0.02)	106.23 (0.05)	102.73 (0.13)	115.24 (0.28)	105.91 (0.64)
Mean (sd) hip circumference, cm	100.28 (0.02)	109.58 (0.05)	107.02 (0.13)	117.73 (0.25)	110.54 (0.62)
Mean (sd) hip-to-waist ratio,	0.94 (<0.01)	0.97 (<0.01)	0.96 (<0.01)	0.98 (<0.01)	0.96 (<0.01)
Mean (sd) height, m	1.78 (<0.01)	1.79 (<0.01)	1.79 (<0.01)	1.81 (<0.01)	1.78 (<0.01)
Comorbidity, %					
Diabetes	8036 (7%)	6155 (14%)	1999 (30%)	861 (18%)	281 (39%)
Heart disease	19927 (16%)	8709 (20%)	1890 (28%)	908 (19%)	165 (23%)
Smoking, %					
Current	11 823 (10%)	3 761 (8%)	638 (9%)	460 (10%)	106 (15%)
Former	67 948 (56%)	26 898 (61%)	3 809 (57%)	3 002 (63%)	370 (52%)
Never	37 149 (31%)	12 130 (27%)	2 012 (30%)	1 110 (23%)	218 (30%)
Missing	3 946 (3%)	1 594 (4%)	264 (4%)	191 (4%)	24 (3%)
Mean (sd) alcohol consumption, g/day	18.69 (0.13)	17.45 (0.21)	15.48 (0.56)	15.57 (0.69)	10.22 (1.14)
Race, %					
Non-Hispanic White	113 685 (94%)	41 819 (94%)	6 303 (94%)	4 447 (93%)	668 (93%)
Hispanic	2 194 (2%)	1 132 (3%)	157 (2%)	145 (3%)	19 (3%)
Black	1 922 (2%)	650 (1%)	129 (2%)	60 (1%)	14 (2%)
Asian, Pacific islander, American Indian, or Alaskan	2 023 (2%)	343 (1%)	48 (1%)	50 (1%)	4 (1%)

For peer review only - http://bmjopen.bmj.com/site/about/guidelines.xhtml

Missing	1 042 (1%)	439 (1%)	86 (1%)	61 (1%)	13 (2%)
Highest Education level achieved, %	5 508 (5%)	2 439 (5%)	415 (6%)	301 (6%)	37 (5%)
Less than 8 years old	16 768 (14%)	6 993 (16%)	1 281 (19%)	813 (17%)	139 (19%)
Completed primary	11 284 (9%)	4 324 (10%)	737 (11%)	474 (10%)	82 (11%)
Completed high school	25 929 (21%)	10 708 (24%)	1 674 (25%)	1 239 (26%)	197 (27%)
Post high school or come college	61 377 (51%)	19 919 (45%)	2 616 (39%)	1 936 (41%)	263 (37%)
College graduate or postgraduate	5 508 (5%)	2 439 (5%)	415 (6%)	301 (6%)	37 (5%)
Vigorous physical activity three or more times per week in the last year, %	91 250 (75%)	29 941 (67%)	4 670 (69%)	2 600 (55%)	438 (61%)
**Nutrition**					
At least 3 fruit servings per day, %	88 699 (73%)	31 603 (71%)	4 855 (72%)	3 335 (70%)	519 (72%)
At least 3 vegetable servings per day, %	56 255 (47%)	19 376 (44%)	3 245 (48%)	2 050 (43%)	364 (51%)
Mean (sd) red meat consumption, g/day	72.68 (0.18)	88.78 (0.37)	86.4 (0.99)	104.96 (1.44)	91.76 (3.6)
Mean (sd) calories per day, kcal	2010 (3)	2095 (5)	2089 (13)	2264 (20)	2146 (42)
Mean (sd) calories per day 10 years ago, kcal					
Mean (sd) calories per day age 12, kcal	1355 (2)	1404 (3)	1473 (8)	1491 (10)	1695 (30)
Drug use					
Ibuprofen use currently					
Aspirin use currently	38720 (32%)	15719 (35%)	2621 (39%)	1697 (36%)	266 (37%)
Fibre					
Mean (sd) fibre from diet age 12, mg/day	9.61 (0.01)	9.85 (0.02)	10.31 (0.07)	10.22 (0.08)	11.2 (0.23)
Mean (sd) fibre from diet 10 years ago, mg/day	6.95 (0.01)	6.95 (0.02)	7.43 (0.05)	7.26 (0.06)	7.71 (0.17)
Mean (sd) fibre CSFII, g/day	20.31 (0.03)	20.45 (0.05)	21.58 (0.14)	21.54 (0.19)	22.6 (0.48)
Mean (sd) fibre total NDSR, g/day	20.71 (0.03)	20.81 (0.05)	21.96 (0.14)	21.93 (0.19)	22.81 (0.5)
Calcium					
Mean (sd) calcium supplement, mg	152.09 (0.75)	136.56 (1.16)	143 (3.1)	138.13 (3.59)	159.2 (10.81)
Mean (sd) calcium from diet current, mg	808.96 (1.34)	845.53 (2.46)	874.55 (6.59)	906.76 (9.07)	910.57 (20.65)
Mean (sd) calcium from diet 10 years ago, mg					
Mean (sd) calcium from diet age 12, mg	653.16 (1.08)	653.03 (1.8)	681.39 (4.77)	672.61 (5.63)	734.68 (15.26)
Folate					
Mean (sd) folate supplement, µg/day	209.35 (0.67)	196.89 (1.13)	207.14 (2.92)	197.72 (3.49)	222.93 (9.74)
Mean (sd) folate pre 1998, µg/day	335.71 (0.48)	336.53 (0.83)	349.14 (2.29)	346.99 (2.85)	355.86 (7.87)

For peer review only - http://bmjopen.bmj.com/site/about/guidelines.xhtml

Mean (sd) folate NDSR, µg/day	447.13 (0.58)	451.13 (1.03)	463.45 (2.85)	470.15 (3.73)	482.46 (10.16)

sd: standard deviation

For peer review only

For peer review only - http://bmjopen.bmj.com/site/about/guidelines.xhtml

Table S6 Characteristics by five latent classes (Model F) of 111,503 women in the NIH-AARP cohort

	Model F (preferred model)				
	**Class I**	**Class II**	**Class III**	**Class IV**	**Class V**
	Stable normal weight	Normal weight to overweight	Normal weight to obese	Overweight to obese	Rapid increase to obese
Number of subjects, N	36 311 (32.7%)	45 832 (41.1%)	23 544 (21.1%)	3 898 (3.5%)	1 918 (1.7%)
Mean (sd) entry age, years	62.48 (0.03)	62.23 (0.02)	61.07 (0.03)	59.61 (0.09)	59.69 (0.12)
Mean (sd) current (at baseline) BMI, kg/m^2	21.97 (0.01)	26.66 (0.01)	32.4 (0.03)	37.47 (0.15)	34.4 (0.19)
Mean (sd) BMI at 50 years, kg/m^2	21.16 (0.01)	24.77 (0.01)	29.52 (0.03)	33.51 (0.13)	37.69 (0.2)
Mean (sd) BMI at 35 years, kg/m^2	20.26 (0.01)	22.53 (0.01)	25.51 (0.02)	28.38 (0.1)	36.92 (0.16)
Mean (sd) BMI at 18 years, kg/m^2	19.3 (0.01)	20.44 (0.01)	22.61 (0.02)	29.01 (0.09)	23.97 (0.1)
Mean (sd) maximum BMI, kg/m^2	23.29 (0.01)	28.13 (0.02)	34.72 (0.04)	40.73 (0.14)	43.18 (0.2)
Mean (sd) minimum BMI, kg/m^2	18.57 (0.01)	20.08 (0.01)	21.85 (0.02)	24.05 (0.07)	24.59 (0.11)
Mean (sd) waist circumference, cm	76.19 (0.04)	86.66 (0.05)	97.82 (0.09)	106.29 (0.32)	101.96 (0.41)
Mean (sd) hip circumference, cm	96.78 (0.03)	105.23 (0.04)	114.7 (0.08)	122.14 (0.31)	118.59 (0.42)
Mean (sd) hip-to-waist ratio,	0.78 (<0.01)	0.82 (<0.01)	0.84 (<0.01)	0.84 (<0.01)	0.85 (<0.01)
Mean (sd) height, m	1.65 (<0.01)	1.64 (<0.01)	1.63 (<0.01)	1.63 (<0.01)	1.62 (<0.01)
Comorbidity, %					
Diabetes	680 (2%)	2344 (5%)	3237 (14%)	725 (19%)	604 (31%)
Heart disease	2269 (6%)	3677 (8%)	2518 (11%)	447 (11%)	296 (15%)
Smoking, %					
Current	5803 (16%)	5660 (12%)	2733 (12%)	495 (13%)	214 (11%)
Former	14173 (39%)	18557 (40%)	9748 (41%)	1764 (45%)	731 (38%)
Never	15313 (42%)	20290 (44%)	10417 (44%)	1507 (39%)	909 (47%)
Missing	1022 (3%)	1325 (3%)	646 (3%)	132 (3%)	64 (3%)
Mean (sd) alcohol consumption, g/day	8.07 (0.1)	6.26 (0.08)	4.47 (0.11)	3.92 (0.29)	3.4 (0.48)
Race, %					
Non-Hispanic White	34468 (95%)	42261 (92%)	21178 (90%)	3446 (88%)	1745 (91%)

For peer review only

Hispanic	699 (2%)	1882 (4%)	1563 (7%)	293 (8%)	105 (5%)
Black	369 (1%)	739 (2%)	324 (1%)	58 (1%)	26 (1%)
Asian, Pacific islander, American Indian, or Alaskan	467 (1%)	443 (1%)	174 (1%)	35 (1%)	15 (1%)
Missing	308 (1%)	507 (1%)	305 (1%)	66 (2%)	27 (1%)
Highest Education level achieved, %					
Less than 8 years old	1155 (3%)	2151 (5%)	1457 (6%)	225 (6%)	124 (6%)
Completed primary	7578 (21%)	11545 (25%)	5984 (25%)	922 (24%)	524 (27%)
Completed high school	3700 (10%)	5021 (11%)	2771 (12%)	489 (13%)	258 (13%)
Post high school or come college	9801 (27%)	11877 (26%)	6088 (26%)	1038 (27%)	521 (27%)
College graduate or postgraduate	14077 (39%)	15238 (33%)	7244 (31%)	1224 (31%)	491 (26%)
Vigorous physical activity three or more times per week in the last year, %	25959 (71%)	30035 (66%)	12692 (54%)	1809 (46%)	1011 (53%)
**Nutrition**					
At least 3 fruit servings per day, %	28441 (78%)	35607 (78%)	17801 (76%)	2889 (74%)	1473 (77%)
At least 3 vegetable servings per day, %	22030 (61%)	27235 (59%)	13282 (56%)	2184 (56%)	1163 (61%)
Mean (sd) red meat consumption, g/day	40.46 (0.19)	46.46 (0.19)	54.41 (0.33)	60.98 (0.98)	56.86 (1.22)
Mean (sd) calories per day, kcal	1546 (4)	1557 (3)	1634 (5)	1727 (15)	1688 (20)
Mean (sd) calories per day 10years ago, kcal	817 (2)	853 (2)	889 (3)	901 (7)	961 (10)
Mean (sd) calories per day age 12, kcal	1250 (3)	1261 (3)	1290 (4)	1336 (10)	1356 (14)
Drug use					
Ibuprofen use currently	4250 (12%)	7065 (15%)	4953 (21%)	1025 (26%)	474 (25%)
Aspirin use currently	6627 (18%)	9559 (21%)	5221 (22%)	913 (23%)	492 (26%)
Hormone Therapy Use, %					
Ever	13989 (39%)	20206 (44%)	12215 (52%)	2241 (57%)	1092 (57%)
Never	22322 (61%)	25626 (56%)	11329 (48%)	1657 (43%)	826 (43%)
Fibre					
Mean (sd) fibre from diet 10years ago, mg/day	7.14 (0.02)	7.22 (0.02)	7.31 (0.03)	7.45 (0.06)	7.57 (0.09)
Mean (sd) fibre from diet age 12, mg/day	8.82 (0.02)	8.96 (0.02)	9.17 (0.03)	9.45 (0.08)	9.39 (0.11)
Mean (sd) fibre CSFII, g/day	18.01 (0.05)	17.75 (0.04)	18.04 (0.06)	18.78 (0.17)	19.14 (0.26)
Mean (sd) fibre total NDSR, g/day	18.05 (0.05)	17.78 (0.04)	18.08 (0.07)	18.85 (0.17)	19.17 (0.26)
Calcium					

For peer review only

Mean (sd) calcium supplement, mg/day	466.85 (2.53)	393.26 (2.1)	337.9 (2.79)	318.84 (6.84)	341.76 (10.22)
Mean (sd) calcium from diet current, mg/day	712.35 (2.23)	716.94 (1.96)	733.95 (2.97)	779.53 (8.16)	765.72 (10.81)
Mean (sd) calcium from diet 10 years ago, mg/day	384.29 (1.18)	387.85 (1.04)	392 (1.5)	390.64 (3.67)	411.4 (5.56)
Mean (sd) calcium from diet age 12, mg/day	677 (2.04)	659.92 (1.81)	650.42 (2.55)	652.48 (6.35)	651.64 (9.03)
Folate					
Mean (sd) folate supplement, µg/day	252.3 (1.23)	239.96 (1.1)	227.06 (1.54)	217.19 (3.78)	228.85 (5.44)
Mean (sd) folate pre 1998, µg/day	291.37 (0.79)	286.07 (0.67)	288.08 (1.02)	295.47 (2.65)	297.1 (3.77)
Mean (sd) folate NDSR, µg/day	375.26 (0.96)	371.15 (0.82)	377.73 (1.26)	389.45 (3.31)	391.35 (4.83)

sd: standard deviation

For peer review only

Table S7 Characteristics by BMI categories of 177,453 men in the AARP cohort

	Normal weight	Overweight	Obese I	Obese II	Obese III
Number of subjects, N (%)	53466 (30.1)	86967 (49.0)	28234 (15.9)	6033 (3.4)	1878 (1.1)
Mean (sd) entry age, years	62.83 (0.02)	62.47 (0.02)	61.87 (0.03)	61.16 (0.07)	60.48 (0.12)
Mean (sd) current (at baseline) BMI, kg/m^2					
Mean (sd) BMI at 50 years, kg/m^2	23.18 (0.01)	27.16 (0)	31.9 (0.01)	36.86 (0.02)	43.93 (0.1)
Mean (sd) BMI at 35 years, kg/m^2	23.28 (0.01)	26.25 (0.01)	29.74 (0.02)	33.33 (0.05)	38.15 (0.15)
Mean (sd) BMI at 18 years, kg/m^2	22.66 (0.01)	24.64 (0.01)	26.89 (0.02)	29.07 (0.05)	31.7 (0.12)
	20.65 (0.01)	21.75 (0.01)	23.14 (0.02)	24.46 (0.05)	25.92 (0.11)
Mean (sd) maximum BMI, kg/m^2					
Mean (sd) minimum BMI, kg/m^2	25.19 (0.01)	28.89 (0.01)	33.58 (0.02)	38.61 (0.05)	45.63 (0.14)
	20.05 (0.01)	21.47 (0.01)	22.85 (0.02)	24.17 (0.05)	25.57 (0.11)
Mean (sd) waist circumference, cm					
Mean (sd) hip circumference, cm	89.56 (0.03)	98.19 (0.03)	108.65 (0.05)	119.1 (0.12)	132.59 (0.29)
Mean (sd) hip-to-waist ratio,	96.92 (0.03)	103.26 (0.02)	111.41 (0.05)	120.44 (0.14)	132.79 (0.36)
Mean (sd) height, m	0.93 (0)	0.96 (0)	0.98 (0)	1 (0)	1 (0)
	70.23 (0.01)	70.07 (0.01)	70.18 (0.02)	69.93 (0.04)	69.7 (0.08)
Comorbidity, %					
Diabetes	3194 (6%)	7752 (9%)	4375 (15%)	1404 (23%)	555 (30%)
Heart disease	8624 (16%)	15314 (18%)	5788 (21%)	1312 (22%)	410 (22%)
Smoking, %					
Current	6513 (12%)	7345 (8%)	2219 (8%)	427 (7%)	129 (7%)
Former	27129 (51%)	51742 (59%)	17719 (63%)	3818 (63%)	1203 (64%)
Never	18089 (34%)	27129 (51%)	7278 (26%)	1557 (26%)	484 (26%)
Missing	1735 (3%)	2948 (3%)	1018 (4%)	231 (4%)	62 (3%)
Mean (sd) alcohol consumption per day, g	18.24 (0.19)	18.36 (0.15)	18.01 (0.28)	16.41 (0.62)	11.72 (0.88)
Race, %					
Non-Hispanic White	50172 (94%)	81972 (94%)	26548 (94%)	5675 (94%)	1738 (93%)
Hispanic	843 (2%)	1775 (2%)	777 (3%)	171 (3%)	68 (4%)
Black	750 (1%)	1448 (2%)	454 (2%)	86 (1%)	28 (1%)
Asian, Pacific islander, American Indian, or Alaskan	1222 (2%)	1001 (1%)	176 (1%)	34 (1%)	15 (1%)
Missing	479 (1%)	771 (1%)	279 (1%)	67 (1%)	29 (2%)

For peer review only - http://bmjopen.bmj.com/site/about/guidelines.xhtml

Highest Education level achieved, %					
Less than 8 years old	2136 (4%)	4259 (5%)	1695 (6%)	422 (7%)	148 (8%)
Completed primary	6591 (12%)	13066 (15%)	4805 (17%)	1075 (18%)	356 (19%)
Completed high school	4643 (9%)	8406 (10%)	2930 (10%)	639 (11%)	209 (11%)
Post high school or come college	10728 (20%)	19668 (23%)	7120 (25%)	1540 (26%)	498 (27%)
College graduate or postgraduate	29368 (55%)	41568 (48%)	11684 (41%)	2357 (39%)	667 (36%)
Vigorous physical activity three or more times per week in the last year, %	41480 (78%)	64458 (74%)	18280 (65%)	3284 (54%)	786 (42%)
**Nutrition**					
At least 3 fruit servings per day, %	40079 (75%)	62921 (72%)	19870 (70%)	4203 (70%)	1303 (69%)
At least 3 vegetable servings per day, %	32887 (62%)	49213 (57%)	15003 (53%)	3220 (53%)	1037 (55%)
Mean (sd) red meat consumption, g/day	66.09 (0.28)	77.79 (0.21)	93.13 (0.44)	107.21 (1.09)	124.23 (2.72)
Mean (sd) calories per day, kcal	1997.3 (3.96)	2019.26 (3.08)	2123.92 (6.21)	2244.87 (14.7)	2411.64 (34.3)
Mean (sd) calories per day 10 years ago, kcal	861.51 (1.57)	865.75 (1.28)	896.59 (2.4)	936.47 (5.47)	974.51 (10.19)
Mean (sd) calories per day age 12, kcal	667.55 (1.63)	650.14 (1.28)	645.21 (2.27)	657.3 (4.93)	667.25 (9.06)
Drug use					
Ibuprofen use currently,	3969 (15%)	8036 (17%)	3688 (23%)	973 (27%)	408 (36%)
Missing	27029 (51%)	39942 (46%)	11894 (42%)	2460 (41%)	743 (40%)
Aspirin use currently,	16456 (40%)	29091 (43%)	10360 (47%)	2174 (47%)	699 (50%)
Missing	12646 (24%)	18622 (21%)	6146 (22%)	1444 (24%)	469 (25%)
Fibre					
Mean (sd) fibre from diet age 12, mg	9.65 (0.02)	9.69 (0.02)	9.84 (0.03)	10.04 (0.07)	10.3 (0.13)
Mean (sd) fibre from diet 10 years ago, mg	7.12 (0.01)	6.89 (0.01)	6.95 (0.02)	7.1 (0.05)	7.35 (0.09)
Mean (sd) fibre CSFII, g	20.95 (0.05)	20.11 (0.03)	20.26 (0.06)	20.85 (0.14)	22.3 (0.28)
Mean (sd) fibre total NDSR, g	21.35 (0.05)	20.49 (0.03)	20.66 (0.07)	21.22 (0.15)	22.65 (0.29)
Calcium					
Mean (sd) calcium supplement, mg	811.3 (2)	812.92 (1.62)	852.6 (3.12)	906.94 (7.45)	973.28 (16.08)
Mean (sd) calcium from diet current, mg	163.44 (1.18)	144.71 (0.85)	131.79 (1.43)	127.03 (3.03)	122.25 (5.34)
Mean (sd) calcium from diet 10 years ago, mg	406 (1)	398.45 (0.79)	404.71 (1.45)	422.22 (3.28)	426.44 (5.87)
Mean (sd) calcium from diet age 12, mg	667.55 (1.63)	650.14 (1.28)	645.21 (2.27)	657.3 (4.93)	667.25 (9.06)
Folate					

For peer review only - http://bmjopen.bmj.com/site/about/guidelines.xhtml

Mean (sd) folate supplement, g	342.65 (0.74)	332.76 (0.56)	335.07 (1.04)	343.98 (2.3)	360.39 (4.58)
Mean (sd) folate pre 1998, g	217.7 (1.01)	205.22 (0.8)	191.35 (1.41)	186.39 (3.01)	180.32 (5.43)
Mean (sd) folate NDSR, g	455.75 (0.92)	443.69 (0.69)	449.63 (1.3)	464.98 (2.95)	490.92 (5.98)

sd: standard deviation

N=875 men classified as <18.0 kg/m^2 at study entry not included

For peer review only

Table S8 Characteristics by BMI categories of 111,503 women in the AARP cohort

	Normal weight	Overweight	Obese I	Obese II	Obese III
Number of subjects, N (%)	48273 (43.3)	36025 (32.2)	16245 (14.6)	6126 (5.5.)	3507 (3.1)
Mean (sd) entry age, years	61.96 (0.02)	62.15 (0.03)	61.89 (0.04)	61.21 (0.07)	60.45 (0.09)
Mean (sd) current (at baseline) BMI, kg/m^2	22.45 (0.01)	27.23 (0.01)	32.12 (0.01)	37.14 (0.02)	44.66 (0.08)
Mean (sd) BMI at 50 years, kg/m^2	22.04 (0.01)	25.31 (0.01)	28.75 (0.03)	32.46 (0.06)	38.33 (0.12)
Mean (sd) BMI at 35 years, kg/m^2	21.12 (0.01)	22.93 (0.01)	24.93 (0.03)	27.22 (0.06)	30.72 (0.11)
Mean (sd) BMI at 18 years, kg/m^2	20.11 (0.01)	20.83 (0.01)	21.88 (0.03)	23.03 (0.05)	24.46 (0.08)
Mean (sd) maximum BMI, kg/m^2	24.21 (0.01)	28.88 (0.02)	34.05 (0.03)	39.21 (0.06)	46.57 (0.11)
Mean (sd) minimum BMI, kg/m^2	19.16 (0.01)	20.3 (0.01)	21.44 (0.02)	22.57 (0.05)	24.24 (0.08)
Mean (sd) waist circumference, cm	76.99 (0.04)	88.19 (0.05)	97.92 (0.08)	106.84 (0.13)	120.12 (0.23)
Mean (sd) hip circumference, cm	97.47 (0.03)	106.73 (0.04)	115.24 (0.07)	124.36 (0.14)	137.66 (0.24)
Mean (sd) hip-to-waist ratio,	0.79 (0)	0.83 (0)	0.85 (0)	0.86 (0)	0.85 (0)
Mean (sd) height, m	1218 (3%)	2199 (6%)	2050 (13%)	1211 (20%)	873 (25%)
Comorbidity, %					
Diabetes	1218 (3%)	2199 (6%)	2050 (13%)	1211 (20%)	873 (25%)
Heart disease	3050 (6%)	3043 (8%)	1749 (11%)	779 (13%)	482 (14%)
Smoking, %					
Current	7699 (16%)	4467 (12%)	1584 (10%)	502 (8%)	256 (7%)
Former	18632 (39%)	14883 (41%)	6843 (42%)	2633 (43%)	1586 (45%)
Never	20558 (43%)	15636 (43%)	7380 (45%)	2823 (46%)	1552 (44%)
Missing	1384 (3%)	1039 (3%)	438 (3%)	168 (3%)	113 (3%)
Mean (sd) alcohol consumption per day, g	7.94 (0.09)	5.92 (0.09)	4.3 (0.13)	3.25 (0.21)	2.67 (0.26)
Race, %					
Non-Hispanic White	45651 (95%)	33005 (92%)	14627 (90%)	5471 (89%)	3090 (88%)
Hispanic	988 (2%)	1688 (5%)	1067 (7%)	472 (8%)	299 (9%)
Black	582 (1%)	581 (2%)	225 (1%)	74 (1%)	45 (1%)
Asian, Pacific islander, American Indian, or Alaskan	620 (1%)	333 (1%)	114 (1%)	32 (1%)	16 (0%)
Missing	432 (1%)	418 (1%)	212 (1%)	77 (1%)	57 (2%)

For peer review only

For peer review only - http://bmjopen.bmj.com/site/about/guidelines.xhtml

Highest Education level achieved, %					
Less than 8 years old	1585 (3%)	1797 (5%)	1017 (6%)	417 (7%)	261 (7%)
Completed primary	10367 (21%)	9077 (25%)	4288 (26%)	1604 (26%)	954 (27%)
Completed high school	4914 (10%)	4138 (11%)	1869 (12%)	740 (12%)	459 (13%)
Post high school or come college	12696 (26%)	9375 (26%)	4327 (27%)	1644 (27%)	938 (27%)
College graduate or postgraduate	18711 (39%)	11638 (32%)	4744 (29%)	1721 (28%)	895 (26%)
Vigorous physical activity three or more times per week in the last year, %	34629 (72%)	23170 (64%)	8917 (55%)	2694 (44%)	1241 (35%)
**Nutrition**	37782 (78%)	27905 (77%)	12355 (76%)	4596 (75%)	2559 (73%)
At least 3 fruit servings per day, %	34422 (71%)	24747 (69%)	10907 (67%)	3921 (64%)	2257 (64%)
At least 3 vegetable servings per day, %					
Mean (sd) red meat consumption, g/day	40.35 (0.17)	47.73 (0.22)	54.09 (0.39)	61.25 (0.67)	71.18 (1.07)
Mean (sd) calories per day, kcal	1531.86 (2.99)	1568.13 (3.81)	1626.68 (6.33)	1696.9 (10.28)	1864.32 (16.6)
Mean (sd) calories per day 10years ago, kcal	818.42 (1.6)	859.29 (2.01)	894.97 (3.17)	910.64 (5.15)	962.24 (7.27)
Mean (sd) calories per day age 12, kcal	673.14 (1.77)	658.24 (2.05)	650.22 (3.07)	646.33 (5.04)	657.19 (6.67)
Hormone Therapy Use, %					
Never	18932 (39%)	16154 (45%)	8334 (51%)	3459 (56%)	2261 (64%)
Ever	29341 (61%)	19871 (55%)	7911 (49%)	2667 (44%)	1246 (36%)
Ibuprofen use currently,	5842 (21%)	5795 (26%)	3352 (33%)	1566 (39%)	1098 (47%)
Ibuprofen use missing	20318 (42%)	14129 (39%)	6018 (37%)	2075 (34%)	1191 (34%)
Aspirin use currently,	8875 (28%)	7709 (32%)	3714 (36%)	1445 (38%)	833 (41%)
Asprin use missing	16220 (34%)	12185 (34%)	5777 (36%)	2364 (39%)	1483 (42%)
,					
Fibre					
Mean (sd) fibre from diet 10years ago, mg	8.82 (0.02)	9.01 (0.02)	9.14 (0.04)	9.32 (0.06)	9.5 (0.08)
Mean (sd) fibre from diet age 12, mg	7.14 (0.02)	7.22 (0.02)	7.36 (0.03)	7.34 (0.05)	7.65 (0.07)
Mean (sd) fibre CSFII, g	18 (0.04)	17.74 (0.05)	17.94 (0.08)	18 (0.12)	19.35 (0.18)
Mean (sd) fibre total NDSR, g	18.05 (0.04)	17.77 (0.05)	17.98 (0.08)	18.05 (0.12)	19.43 (0.18)
Calcium					
Mean (sd) calcium supplement, mg	710.52 (1.9)	719.2 (2.26)	733.66 (3.61)	751.64 (5.75)	808.42 (8.73)
Mean (sd) calcium from diet current, mg	458.8 (2.18)	382.25 (2.35)	333.6 (3.31)	307.85 (5.3)	270.5 (6.54)
Mean (sd) calcium from diet 10 years ago, mg	382.09 (1.01)	388.3 (1.19)	397.37 (1.82)	397.86 (2.95)	414.1 (4.09)

For peer review only - http://bmjopen.bmj.com/site/about/guidelines.xhtml

Mean (sd) calcium from diet age 12, mg	673.14 (1.77)	658.24 (2.05)	650.22 (3.07)	646.33 (5.04)	657.19 (6.67)
Folate					
Mean (sd) folate supplement, g	250.24 (1.07)	239.5 (1.25)	225.57 (1.84)	217.96 (3.02)	209.48 (4.03)
Mean (sd) folate pre 1998, g	290.16 (0.68)	285.89 (0.78)	287.43 (1.2)	288.09 (1.98)	304.36 (2.79)
Mean (sd) folate NDSR, g	373.99 (0.82)	371.32 (0.96)	376.02 (1.49)	380.5 (2.43)	407.36 (3.5)

sd: standard deviation
N=1327 Women classified as <18.0 kg/m^2 at study entry not included

For peer review only

Figure S4 Latent Class trajectories derived on individuals with at least three time points, with random quadratic effect proportional specification (model F) (left: Men; right: Women)

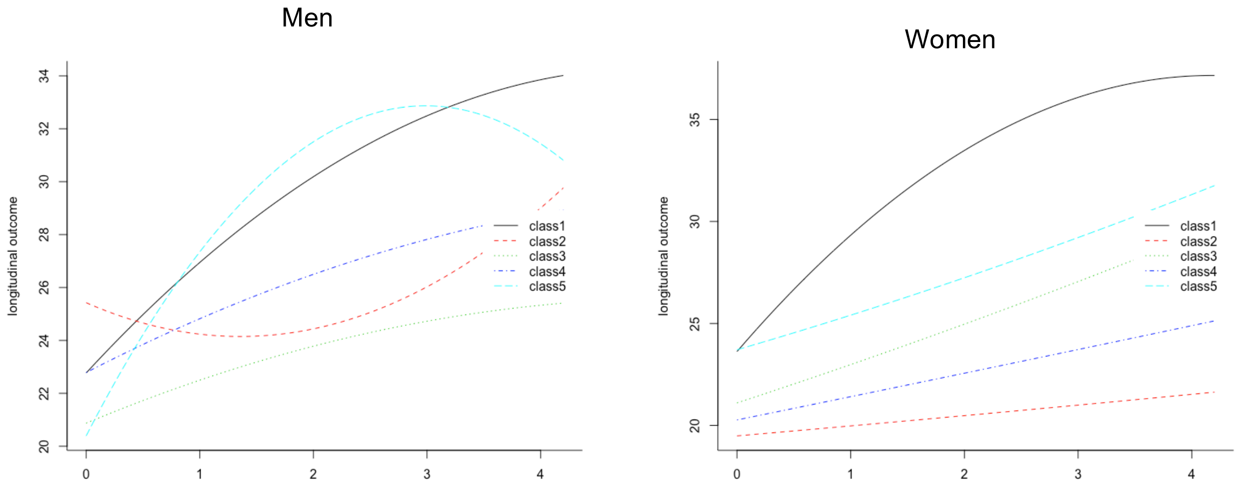

er review only

References

1. Kubzansky LD, Gilthorpe MS, Goodman E. A prospective study of psychological distress and weight status in adolescents/young adults. Annals of Behavioral Medicine 2012;43(2):219-228.

2. Kelly SP, Graubard BI, Andreotti G, Younes N, Cleary SD, Cook MB. Prediagnostic Body Mass Index Trajectories in Relation to Prostate Cancer Incidence and Mortality in the PLCO Cancer Screening Trial. J Natl Cancer Inst 2017;109(3).

3. Song M, Willett WC, Hu FB, Spiegelman D, Must A, Wu K, et al. Trajectory of body shape across the lifespan and cancer risk. Int J Cancer 2016;138(10):2383-95.

4. Song M, Hu FB, Wu K, Must A, Chan AT, Willett WC, et al. Trajectory of body shape in early and middle life and all cause and cause specific mortality: results from two prospective US cohort studies. BMJ 2016;353:i2195.

5. Zheng H, Tumin D, Qian Z. Obesity and Mortality Risk: New Findings From Body Mass Index Trajectories. American Journal of Epidemiology 2013;178(11):1591-1599.

6. Elsensohn M-H, Klich A, Ecochard R, Bastard M, Genolini C, Etard J-F, et al. A graphical method to assess distribution assumption in group-based trajectory models. Statistical methods in medical research 2016;25(2):968-982.

7. Vistisen D, Witte DR, Tabak AG, Herder C, Brunner EJ, Kivimaki M, et al. Patterns of obesity development before the diagnosis of type 2 diabetes: the Whitehall II cohort study. PLoS Med 2014;11(2):e1001602.

8. Lubke G, Neale MC. Distinguishing between latent classes and continuous factors: Resolution by maximum likelihood? Multivariate Behavioral Research 2006;41(4):499-532.

9. Peugh J, Fan X. How Well Does Growth Mixture Modeling Identify Heterogeneous Growth Trajectories? A Simulation Study Examining GMM's Performance Characteristics. Structural Equation Modeling: A Multidisciplinary Journal 2012;19(2):204-226.

10. Peugh J, Fan X. Enumeration Index Performance in Generalized Growth Mixture Models: A Monte Carlo Test of Muthén's (2003) Hypothesis. Structural Equation Modeling: A Multidisciplinary Journal 2015;22(1):115-131.

11. Jedidi K, Ramaswamy V, Desarbo WS. A maximum likelihood method for latent class regression involving a censored dependent variable. Psychometrika 1993;58(3):375-394.

For peer review only

For peer review only - http://bmjopen.bmj.com/site/about/guidelines.xhtml

BMJ Open

A framework to construct and interpret latent class trajectory modelling

Journal:	*BMJ Open*
Manuscript ID	bmjopen-2017-020683.R3
Article Type:	Research
Date Submitted by the Author:	19-Mar-2018
Complete List of Authors:	Lennon, Hannah; University of Manchester, Division of Cancer Sciences; University of Manchester Institute of Population Health<br>Kelly, Scott; National Institutes of Health, Division of Cancer Epidemiology and Genetics, National Cancer Institute<br>Sperrin, Matthew; University of Manchester Institute of Population Health, Division of Informatics, Imaging and Data Sciences<br>Buchan, Iain; University of Manchester Institute of Population Health, Division of Informatics, Imaging and Data Sciences<br>Cross, Amanda; Imperial College London School of Public Health, Department of Epidemiology and Biostatistics<br>Leitzmann, Michael; Universitat Regensburg Fakultat fur Medizin, Department for Epidemiology and Preventive Medicine<br>Cook , Michael ; National Institutes of Health, Division of Cancer Epidemiology and Genetics, National Cancer Institute<br>Renehan, Andrew; University of Manchester, Division of Cancer Sciences; University of Manchester Institute of Population Health, Manchester Cancer Research Centre, NIHR Manchester Biochemical Research Centre
**Primary Subject Heading**:	Research methods
Secondary Subject Heading:	Health informatics, Epidemiology, Medical education and training, Communication
Keywords:	Latent class models, Growth curves, Growth mixture models, Lifetime obesity, Trajectories

SCHOLARONE™
Manuscripts

Original article (formatted for BMJ Open)

A framework to construct and interpret latent class trajectory modelling

Hannah Lennon, [1,3] Scott Kelly,[2] Matthew Sperrin,[3] Iain Buchan,[3] Amanda J Cross,[4] Michael Leitzmann,[5] Michael B Cook,[2] Andrew G Renehan[1,3,6]

[1] Division of Cancer Sciences, School of Medical Sciences, Faculty of Biology, Medicine and Health, University of Manchester, Manchester, UK

[2] Division of Cancer Epidemiology and Genetics, National Cancer Institute, National Institutes of Health, Maryland, US

[3] MRC Health eResearch Centre (HeRC), Division of Informatics, Imaging and Data Sciences, University of Manchester, Manchester, UK

[4] Department of Epidemiology and Biostatistics, Imperial College, London, UK

[5] Department of Epidemiology and Preventive Medicine, University of Regensburg, Regensburg, Germany

[6] Manchester Cancer Research Centre, NIHR Manchester Biochemical Research Centre, University of Manchester, Manchester, UK

Running title: *latent class modelling*

Keywords*: Latent class models, growth curves, growth mixture models, body mass index, trajectories, lifetime obesity*

Correspondence to:

Hannah Lennon
Manchester Cancer Research Centre
NIHR Manchester Biomedical Research Centre
Division of Cancer Sciences, School of Medical Sciences,
Faculty of Biology, Medicine and Health,
University of Manchester,
Manchester,
M13 9PL UK
Tel: +44 161 306 7925
E-mail: hannah.lennon@manchester.ac.uk

Abstract: 274 words; main text: 3365 words; 5 tables; 2 figures; References 30 (max: 30); Supplemental material (18 pages); language: UK English.

For peer review only - http://bmjopen.bmj.com/site/about/guidelines.xhtml

ABSTRACT

Objectives: Latent class trajectory modelling (LCTM) is a relatively new methodology in epidemiology to describe life-course exposures, which simplifies heterogeneous populations into homogeneous patterns or classes. However, for a given dataset, it is possible to derive scores of different models based on number of classes, model structure, and trajectory property. Here, we rationalise a systematic framework to derive a 'core' favoured model.

Methods: We developed an eight step framework: step 1, a scoping model; step 2, refining the number of classes; step 3, refining model structure (from fixed-effects through to a flexible random-effect specification); step 4, model adequacy assessment; step 5, graphical presentations; step 6, use of additional discrimination tools ('degree of separation'; Elsensohn's envelope of residual plots); step 7, clinical characterisation and plausibility; and step 8, sensitivity analysis. We illustrated these steps using data from the NIH-AARP cohort of repeated determinations of body mass index (BMI) at baseline (mean age: 62.5 years), and BMI derived by weight recall at ages 18, 35 and 50 years.

Results: From 288,993 participants, we derived a 5-class model for each gender (men: 177,455; women: 111,538). From seven model structures, the favoured model was a proportional random quadratic structure (model F). Favourable properties were also noted for the unrestricted random quadratic structure (model G). However, class proportions varied considerably by model structure – concordance between Models F and G were moderate (Cohen κ: men, 0.57; women, 0.65) but poor with other models. Model adequacy assessments, evaluations using discrimination tools, clinical plausibility, and sensitivity analyses supported our model selection.

Conclusion: We propose a framework to construct and select a 'core' LCTM, which will facilitate generalisability of results in future studies.

Funding: Cancer Research UK National Awareness and Early Detection Initiative (NAEDI), Award Number A17962.

For peer review only - http://bmjopen.bmj.com/site/about/guidelines.xhtml

For peer review only

Strengths and limitations of this study

- We developed a systematic approach, with rationale for each of eight steps, to derive a latent class trajectory model of favoured number of classes and 'core' model structure specification
- The results presented here are based on modelling data from a large well-characterised US cohort; allowing the derivation of numerically meaningful subpopulations (i.e., classes) with distinct phenotypes
- Compared with 'one-off' BMI categorisation, latent class trajectory modelling offers additional phenotypic information and opportunities to identify and intervene early in sub-populations with adverse trajectories
- Whilst we described multiple diagnostic tests, ultimately model selection was based on case-study appropriate model interpretation (for example, model adequacy; discrimination; clinical plausibility; sensitivity analyses) by a multi-disciplinary research team

For peer review only

Introduction

In many epidemiological studies, a risk factor is measured at a single point in time and related to the subsequent development of disease, under the assumption that a single 'one-off' measure is an approximation for that exposure over a long time. Thus, baseline measurement of body mass index (BMI) is associated with subsequent development of common disease like cardiovascular disease (1), diabetes (2), several cancers (3), and all-cause mortality (4). This approach is crude and many investigators seek to use alternative methods that might better capture long-term risk factor exposure termed *life-course analysis*. There are widely-used examples that capture cumulative exposure, such as pack-years for smoking and lung cancer, but the assumption that incidence rate is proportional to total lifetime dose is questionable (5). Many other life-course models simply extract features for use in standard regression approaches; for example, a weight change over time. A more sophisticated approach, which takes account of within-individual correlations, is *mixed-effect modelling*, but this is difficult to interpret for public health implementation. An extension of this approach is the use of *latent classes*, also termed growth mixture models.

Latent class trajectory modelling (LCTM) simplifies heterogeneous populations into more homogeneous clusters or classes. From these, one can potentially include random effects to allow for individual variation within these classes. These models have a long history in the criminology (6) and psychology (7) literatures, and now, are increasingly reported in the human epidemiology literature (for example, disentangling the heterogeneity of childhood asthma (8)). Of relevance to this paper, LCTM has been used in association studies of repeated BMI measures with the following endpoints: all-cause mortality (9); cancer incidence (multiple cancer types (10)); gastro-oesophageal (11); prostate (12)); and cancer mortality (12). The LCTM has three general advantages compared with using 'one-off' exposure determinations: first, it better informs aetiological associations by deeply phenotyping certain 'at risk' subpopulations; and second, LCTM offers a public health strategy to identify early divergent adverse trajectories as potential intervention targets. Some researchers additionally argue that LCTM is well-equipped for future forecasting and

For peer review only - http://bmjopen.bmj.com/site/about/guidelines.xhtml

new-patient generalisations in prediction models, as it handles data following a different predictable pattern from that learnt by the model (13). Thirdly, the trajectory approach allows a better understanding of the causes of between-individual variation in certain features (e.g., weight variation over age), by analysing the trajectory as an outcome rather than exposure.

However, LCTM is a complex form of modelling and requires several different structure assumptions (14). Although firmly acknowledged in the GRoLTS-Checklist: Guidelines for Reporting on Latent Trajectory Studies (15), structure-related assumptions have not been systematically evaluated. For many exposures of interest, typically two to seven classes might be described and, as detailed latter, at least seven model structures might be fitted, with and without linear curve properties, such that it is possible to derive greater than eighty different models. Thus, reported differences between studies using latent class modelling might reflect different modelling assumptions rather than true differences between populations. To facilitate the generalizability of results in future studies, here, we propose a framework to construct and select a 'core' LCTM, using an example of repeatedly determined body mass index (BMI) across adulthood in the National Institutes of Health (NIH)-AARP Diet and Health Study cohort. For exposure-disease outcome association analyses, current approaches generally use two stages: first, latent class trajectory modelling, followed by standard association modelling. The framework described here is limited to the first stage.

METHODS

Cohort

The NIH-AARP Diet and Health Study is a US cohort recruited from 1995 (16). A baseline medical and lifestyle questionnaire, including self-reported weight and height, was returned by 566,398 participants (aged 50 to 71 years; mean age: 62.5 years). An additional risk factor questionnaire was mailed in 1996 and completed by a sub-cohort of 327,860; of whom 288,993 (177,455 men and 111,538 women) provided recall weight for all four time-points -

For peer review only - http://bmjopen.bmj.com/site/about/guidelines.xhtml

ages 18, 35 and 50 years. Derived BMIs at baseline and these ages (assuming constant height) form the data in the present analysis. We excluded participants with extreme BMI values (<15 or >70 kg/m^2) recorded at any time point. Means and standard deviations for derived recalled BMI distributions are representative of BMI distributions for historical period-equivalent US populations (17).

Latent class trajectory modelling

We developed an eight step framework **(Table 1)** modelling BMI as a function of age. Latent classes were used to identify subgroups of participants with distinct trajectories (detailed mathematical equations in supplemental material p2) (18). We used maximum likelihood approaches to fit the model with the '*hlme'* function from 'lcmm' library (19) in the R software environment (version 3.2.1) and cross-checked results using the '*PROC TRAJ'* function in 'SAS traj' (SAS Institute, Inc., Cary, NC) (20) (Table S1).

Step 1: We initially constructed a scoping model provisionally selecting the plausible number of classes based on available literature – in the context of BMI trajectories, we used *K* = 5 classes as reported elsewhere (10, 12). We built models for both genders, as BMI patterns of lifetime changes differ for men and women (21). To determine the initial working model structure, we followed the rationale of Verbeke and Molenburgh (22) and examined the shape of standardised residual plots for each of the five classes in a model with no random-effects. If the residual profile could be approximated by a flat, straight line or a curve, then a random intercept, slope or quadratic term, respectively, were considered. Preliminary plots suggested preference for a quadratic random-effects model (Figure S1).

Step 2: We refined the preliminary working model from step 1 to determine the optimal number of classes, testing *K* = 1 to 7. The number of classes chosen was based on the lowest Bayesian Information Criteria (BIC).

For peer review only - http://bmjopen.bmj.com/site/about/guidelines.xhtml

Step 3: We further refined the model, using the favoured *K* derived in step 2, testing for the optimal model structure. We tested seven models (detailed in Table S2): ranging from a simple fixed-effects model (model A) through a rudimentary method that allows the residual variances to vary between classes (model B) to a suite of five random-effects models with different variance structures (models C-G).

Step 4: We then performed a number of model adequacy assessments. First, for each participant, we calculated the posterior probability of being assigned to each trajectory class, and assigned the individual to the class with the highest probability. An average of these maximum Posterior Probability of Assignments (APPA) above 70%, in all classes, is regarded as acceptable (6). We further assessed model adequacy using Odds of Correct Classification (OCC), mismatch scores and Entropy, E_k (detailed in Table S3). These diagnostic tools assist in model selection (6, 23). In some examples, BIC values may decrease as more groups and parameters are added reflecting model overfit. Therefore, the BIC value might not always provide the optimum selection criteria, and model selection must balance between meaningful trajectories, model parsimony and model adequacy. For example, if the model adequacy measures are strongly violated, one might go back to steps 2 and 3 and consider a different model with a higher BIC value. We selected an optimal model structure using the lowest BIC value and satisfactory values from the model adequacy assessments and referred to the outcome of steps 1-4 as the *favoured model*. To assess the interpretability of the resulting classes, we investigated characteristics of lifestyle behaviours of the favoured model such as smoking, alcohol consumption and physical activity.

Step 5: We used three graphical presentation approaches. The conventional approach is to plot mean trajectories with time encompassing each class. Alternatives include the use of mean trajectory plots with 95% predictive intervals for each class, which displays the predicted random variation within each class; or to plot individual level 'spaghetti plots' with

For peer review only - http://bmjopen.bmj.com/site/about/guidelines.xhtml

time (for example, a random sample of participants), which allows the reader to observe the patterns of changes within classes.

Step 6: We assessed model discrimination, including Degrees of Separation, DoS_K (24, 25) and Elsensohn's envelope of residuals (26). To describe the separation of latent trajectory curves, a multivariate Mahalanobis distance was used. Peugh and Fan (25) argue that it is reasonable to speculate that identification of heterogeneous latent trajectories is facilitated by large statistical separation distance among the subpopulations. Thus, larger values of DoS_K indicate the mean trajectories are well separated while DoS_K equal to zero is the special case when all mean trajectories are identical. If the DoS_K value is small, then one might consider a model with fewer classes.

To check structure assumptions in fixed-effects latent class models, Elsenhohn et al. (26) plotted the local standard deviations of the residuals against time. We extended this method to random-effects models; first, computing the observed residuals for each participant; and second, computing the class- and time-specific weighted local variance of the residuals, with weights being the posterior probabilities of individual belonging to a class. We plotted the upper and lower boundary values of the local standard deviations of the residuals around the mean values for each class. The resulting shape indicates the appropriateness of the model assumptions; where non-parallel boundaries indicate heteroscedasticity of residuals suggesting poor model fit, and differing interval widths suggest that across class variability may not be fully accounted for.

Step 7: We assessed for clinical characterisation and plausibility using four approaches: i) assessing the clinical meaningfulness of the trajectory patterns, aiming to include classes with at least 1% capture of the population; ii) assessing the clinical plausibility of the trajectory classes; iii) tabulation of characteristics by latent classes versus conventional categorisations; and iv) concordance of class membership with conventional BMI category

For peer review only - http://bmjopen.bmj.com/site/about/guidelines.xhtml

membership using the kappa statistic (as LCTM is an unsupervised learning approach, we computed κ for all possible combinations and selected the optimal κ).

Step 8: We conducted sensitivity analyses, in this example, with individuals with at least two and three BMI values, as LCTMs are flexible enough to deal with different observation times between participants.

Patient and Public Involvement

No patients and or public were involved with this manuscript.

Statistical algorithms

All R and SAS codes used to implement these tools are available via the authors and can be downloaded from *www.github.com/hlennon/LCTMtools*.

RESULTS

Number of classes

From the preliminary working model of a quadratic random-effects model, model F (proportional covariance structure), we derived BICs for up to seven classes: three of the class models failed to converge in men and women. **Table 2** reports that the lowest BIC was obtained with five classes in men and women – confirming our initial working model. The proportions by class in men were 68.1%, 25.0%, 3.8%, 2.7%, and 0.4%; and in women, were 32.6%, 41.1%, 21.1%, 3.5% and 1.7%. For model G (our second favoured model), the lowest BICs were noted for five classes in men and women (Table S2).

Assessment for model structures

With the number of classes now selected as five, we tested the seven model structures – A to G. **Table 3** reports that the lowest BIC was for model F in men and women, justifying the selection of model F in the preliminary working phase. The class sizes varied between

For peer review only - http://bmjopen.bmj.com/site/about/guidelines.xhtml

models, with Class I ranging from 41% to 68% in men and from 32% to 95% in women. The APPA for each class in model F was 0.81, 0.74, 0.87, 0.83, and 0.74 in men and 0.74, 0.79, 0.80, 0.83 and 0.84 in women, indicating a good discrimination of trajectory. The classes were well differentiated with the relative Entropy, E_K values ranging from 0.59 to 0.81 in men and 0.66 to 0.83 in women.

There was moderately good concordance (unweighted and weighted) between the unstructured variance models G with model F in men (κ: 0.57) and women (κ: 0.65) (Tables S4 and S5), but poorer concordance between the favoured models and fixed-effects models in men.

Graphical presentation

We plotted the mean trajectories for model A, B, C, D, F and G in men and women **(Figure 1)** illustrating the increased complexity from model A to G. As alternatives, we plotted separately mean trajectories with 95% predictive intervals for each class, in model F (Figure S2), which displays the predicted random variation within each of the classes with time, noting that variation was greater with the more 'complex' classes (classes IV and V compared with classes I, II and III). Spaghetti plots of individual level data illustrated that the timing and size of BMI changes characterise the classes – for example, sharp increases in BMI in early adulthood in Class III but later in adulthood for Class IV (Figure S3).

Additional tools of suitability of fit

The DoS_k values ranged from 0.10 to 0.36 and 0 to 0.34, in men and women, respectively **(Table 3)**. The covariances were high and in the positive direction and therefore models with non-parallel mean trajectories lead to higher separation.

We plotted the local standard deviation of the residuals with time and found that these were broadly homogeneous, i.e., there were few parallel boundaries **(Figure 2)**. The local residuals for the rapidly obese groups in both genders are the exceptions to parallel lines, which might reflect comorbidities in this group and smaller numbers.

For peer review only - http://bmjopen.bmj.com/site/about/guidelines.xhtml

For peer review only

Clinical assessment

Having established the favoured model, model F with five classes in both genders, we assigned descriptive labels to each respective class as follows **(Table 4)**: stable normal weight; normal weight to overweight; normal weight to obese; overweight to obese; and rapid early obesity. We noted that the proportion in the rapid early obesity (Class V) was less than 1% in men. However, overall, the proportion for Class V for men and women combined was nearly 1%. Thus, we retained this class as we judged it to be clinically meaningful as follows. In both genders, there were rapid increases in obesity from early to middle adulthood, then apparent severe weight reductions. We rationalised that this was clinically plausible, as it could be explained either by intentional (e.g. bariatric surgery) or non-intentional weight loss (e.g. reverse causality from development of disease).

We then tabulated the baseline characteristics according to the five classes for model F in men and women, and noted patterns across the classes **(Table 4** – and fully expanded in Tables S5 and S6). Thus, for example, for current smoking status, there were little differences in patterns by class in men: 10%, 8%, 9%, 10%, and 15%; and in women, 16%, 12%, 12%, 13% and 11%. This contrasts with BMI categories—men (12%, 8%, 8%, 7% and 7%) and women (16%, 12%, 10%, 8%, and 7%) (Tables S7 and S8).

Finally, we noted very poor concordance between the favoured model and conventional BMI categorisation in men (κ: 0.18) and women (κ: 0.52) **(Table 5)**.

Sensitivity analyses

We tested the favoured model using a larger sample of individuals with at least three measures, and found no material differences between these models, in men and women, and the main model (Figure S4).

Discussion

Main findings

For peer review only - http://bmjopen.bmj.com/site/about/guidelines.xhtml

We propose an eight-step framework for the construction and selection of models derived from latent class trajectory modelling. We evaluated a range of model structures from fixed-effect models to a set of random-effects models, favouring the latter models in this case study, as they include different variance structures, and more likely to reflect the natural history of changes with time in BMI distributions in different sub-populations. We showed that different model structures resulted in different classes with contrasting clinical phenotypes. We propose pre-specified criteria for model selection and that the reporting of a 'core' model will facilitate generalizability of results in future studies.

Context of other literature

To the best of our knowledge, this is the first study to systematically address structure-related assumptions in LCTMs, and their potential impact on clinically-relevant endpoints – in this example, BMI trajectories. Anecdotally, there is a justifiable criticism regarding the use of LCTM models and an uncertainty of how class memberships are derived – a 'black box' effect. The proposed framework, here, encourages the opposite – a transparent stepwise approach to class and model structure selection. To enhance this process, for example, we have 'borrowed' tools developed to address to quantify uncertainty, such as entropy measures, E and E_k, and applied them to assist assessment of model adequacy. A further modification of discrimination measurement with variance estimation has been described by Shah and colleagues (27), and might have importance for class assignment where 'yes/no' treatment decisions are required.

Variations of model A (fixed-effects) have been reported in the clinical literature (9-12), which assume no within-class variability when deriving latent classes. Interpretation in this setting is that variation from the mean trajectory is random, i.e., the correlation between measurements for the same individual is explained by latent class membership. In the context of any repeated measures in the general population, this assumption might not be valid (14). Saunders (28) argued in support of full random-effects models (i.e. models F and G), calling upon Moffitt's theory from criminology, which recognises that "*there are distinct*

For peer review only - http://bmjopen.bmj.com/site/about/guidelines.xhtml

developmental clusters of trajectories of anti-social behaviour that are the result of divergent aetiologies" – in other words, it is unlikely that latent classes start from a similar baseline.

The publication of the 16-item GRoLTS Guidelines in 2017 (15) heralded an important advance for the application of LCTM. Here, we add a framework for construction and interpretation.

Strengths and weaknesses

The study has strengths. First, the considered and strategic workflow to optimise identification and application of latent classes provides for a more robust and transparent application of these models in epidemiology. Second, the results presented are based on modelling data from a large well-characterised US cohort, therefore allowing the derivation of numerically meaningful subpopulations (i.e. classes) with distinct phenotypes. We uniquely used averaged kappa values to demonstrate that the LCTM-derived subpopulations are markedly different to those derived from a 'one-off' BMI determinations. In turn, BMI trajectories are more likely to reflect normal clinical practice of considering a 'weight history'. Third, we extensively explored different model selections and adequacy tools, and described extensions to other tools, to supplement model interpretation. Fifth, we further supplement model interpretation, we embedded this project within a multi-disciplinary research team including data scientists, statisticians, clinicians and epidemiologists – an approach echoed elsewhere (29). Finally, we have made the statistical algorithms freely available.

There are several study weaknesses. First, LCTMs currently only considers trajectories of one risk factor at a time. Second, there were only four time points in the AARP such that it was not possible to assess weight cycling. Third, whilst we described multiple diagnostic tests, ultimately model selection was based on case-study appropriate model interpretation (for example, model adequacy; discrimination; clinical plausibility; sensitivity analyses) as well as likelihood-based model-fit criteria (18). Some discussion on statistical power and efficiency is warranted. The objective of model selection is a trade-off between efficiency and validation with the aim of summarising distinct features of the data as

For peer review only - http://bmjopen.bmj.com/site/about/guidelines.xhtml

parsimonious as possible, and not just the maximisation of model fits (6). For example, in a hypothetical scenario, putting too much emphasis on the validity of a model in which ten classes provide the best model fit is questionable if three of the ten classes each include less than 0.5% of the population and do not show markedly different characteristics.

Clinical implications and future research

We showed that different model structures resulted in different classes with contrasting clinical phenotypes. Thus, for example, it is well-recognised that the proportions of current smokers decreases with increasing BMI categories. However, this ‘trend’ is not observed across the latent classes derived in our favoured model F, suggesting that the clinical characteristics derived from the LCTM differed from those derived from conventional categorisation approaches. Thus, compared with ‘one-off’ BMI categorisation, latent class trajectory modelling offers additional phenotypic information.

For future research, improving the construction, interpretation and reporting of LCTM (advocated here) is hugely important as the LCTM approach has opportunities to identify and intervene early in sub-populations with adverse trajectories. This approach is analogous to the well-held public strategy of using childhood growth charts to identify and intervening in young children failing to thrive. Thus, in the example of BMI, remembering that 80% of obese adults were not obese in childhood (30), future LCTM studies might identify (new) individuals in their 20s or early 30s on adverse trajectories towards later adulthood obesity. This strategy is a new methodological paradigm, as the repeated measurement of a risk factor (here, BMI) becomes a clinically-relevant endpoint rather than just an exposure.

For peer review only - http://bmjopen.bmj.com/site/about/guidelines.xhtml

Role of the funding source

The funders of the study had no role in study design, data collection, data analysis, data interpretation, or writing of the report.

Conflict of Interest

AGR has received lecture honoraria from Merck Serona and Janssen-Cilag, and independent research funding from Novo Nordisk. All other authors have no conflicts of interest to declare.

Contributions

HL, MS, MC and AGR conceptualised the paper. HL, MS and SK designed the statistical approaches; HL performed the modelling. AC and ML facilitated data access and interpretation of the AARP data. All authors contributed to data interpretation; IB and AGR put modelling into clinical context.

Acknowledgements

We acknowledge the generous funding from Cancer Research UK National Awareness and Early Detection Initiative (NAEDI).

Data sharing statement

No additional data available

For peer review only - http://bmjopen.bmj.com/site/about/guidelines.xhtml

References

1. Wormser D, Kaptoge S, Di Angelantonio E, Wood AM, Pennells L, Thompson A, et al. Separate and combined associations of body-mass index and abdominal adiposity with cardiovascular disease: collaborative analysis of 58 prospective studies. Lancet 2011;377(9771):1085-95.

2. Vazquez G, Duval S, Jacobs DR, Jr., Silventoinen K. Comparison of body mass index, waist circumference, and waist/hip ratio in predicting incident diabetes: a meta-analysis. Epidemiol Rev 2007;29:115-28.

3. Lauby-Secretan B, Scoccianti C, Loomis D, Grosse Y, Bianchini F, Straif K. Body Fatness and Cancer--Viewpoint of the IARC Working Group. Colditz G, Anderson AS, Herbert RA, Kaaks R, Thompson HJ, Baker JL, Breda J, Byers T, Cleary MP, Di Cesare M, Gapstur SM, Gunter M, Hursting SD, Leitzmann M, Ligibel J, Renehan A, Romieu I, Shimokawa I, Ulrich CM, Wade K, Weiderpass E. N Engl J Med 2016;375(8):794-8.

4. Global BMI Mortality Collaboration. Body-mass index and all-cause mortality: individual-participant-data meta-analysis of 239 prospective studies in four continents. Lancet 2016.

5. Peto J. That the effects of smoking should be measured in pack-years: misconceptions 4. Br J Cancer 2012;107(3):406-7.

6. Nagin D. Group-based modeling of development: Harvard University Press; 2005.

7. Stevenson J, Thompson MJ, Sonuga-Barke E. Mental health of preschool children and their mothers in a mixed urban/rural population. III. Latent variable models. Br J Psychiatry 1996;168(1):26-32.

8. Belgrave DC, Buchan I, Bishop C, Lowe L, Simpson A, Custovic A. Trajectories of lung function during childhood. Am J Respir Crit Care Med 2014;189(9):1101-9.

9. Song M, Hu FB, Wu K, Must A, Chan AT, Willett WC, et al. Trajectory of body shape in early and middle life and all cause and cause specific mortality: results from two prospective US cohort studies. BMJ 2016;353:i2195.

10. Song M, Willett WC, Hu FB, Spiegelman D, Must A, Wu K, et al. Trajectory of body shape across the lifespan and cancer risk. Int J Cancer 2016;138(10):2383-95.

11. Petrick JL, Kelly SP, Liao LM, Freedman ND, Graubard BI, Cook MB. Body weight trajectories and risk of oesophageal and gastric cardia adenocarcinomas: a pooled analysis of NIH-AARP and PLCO Studies. Br J Cancer 2017;116(7):951-959.

12. Kelly SP, Graubard BI, Andreotti G, Younes N, Cleary SD, Cook MB. Prediagnostic Body Mass Index Trajectories in Relation to Prostate Cancer Incidence and Mortality in the PLCO Cancer Screening Trial. J Natl Cancer Inst 2017;109(3).

13. Qian T, Masino AJ. Latent Patient Cluster Discovery for Robust Future Forecasting and New-Patient Generalization. PLoS One 2016;11(9):e0162812.

14. Gilthorpe MS, Dahly DL, Tu YK, Kubzansky LD, Goodman E. Challenges in modelling the random structure correctly in growth mixture models and the impact this has on model mixtures. J Dev Orig Health Dis 2014;5(3):197-205.

For peer review only - http://bmjopen.bmj.com/site/about/guidelines.xhtml

15. van de Schoot R, Sijbrandij M, Winter SD, Depaoli S, Vermunt JK. The GRoLTS-Checklist: Guidelines for Reporting on Latent Trajectory Studies. . Structural Equation Modeling: A Multidisciplinary Journal;24:451-467.

16. Schatzkin A, Subar AF, Thompson FE, Harlan LC, Tangrea J, Hollenbeck AR, et al. Design and serendipity in establishing a large cohort with wide dietary intake distributions : the National Institutes of Health-American Association of Retired Persons Diet and Health Study. Am J Epidemiol 2001;154(12):1119-25.

17. Renehan AG, Flood A, Adams KF, Olden M, Hollenbeck AR, Cross AJ, et al. Body mass index at different adult ages, weight change, and colorectal cancer risk in the National Institutes of Health-AARP Cohort. American journal of epidemiology 2012;176(12):1130-1140.

18. Tu Y-K, Gilthorpe MS. Statistical thinking in epidemiology: CRC Press; 2011.

19. Proust-Lima C, Philipps V, Diakite A, Liquet B. Package 'lcmm' Title Extended Mixed Models Using Latent Classes and Latent Processes. In; 2016.

20. Jones BL, Nagin DS. Advances in group-based trajectory modeling and an SAS procedure for estimating them. Sociological Methods & Research 2007;35(542-571).

21. Sperrin M, Marshall AD, Higgins V, Buchan IE, Renehan AG. Slowing down of adult body mass index trend increases in England: a latent class analysis of cross-sectional surveys (1992-2010). Int J Obes (Lond) 2014;38(6):818-24.

22. Verbeke G, Molenberghs G. Linear Mixed Models for Longitudinal Data. 2000.

23. Klijn SL, Weijenberg MP, Lemmens P, van den Brandt PA, Lima Passos V. Introducing the fit-criteria assessment plot - A visualisation tool to assist class enumeration in group-based trajectory modelling. Stat Methods Med Res 2015.

24. Lubke G, Neale MC. Distinguishing between latent classes and continuous factors: Resolution by maximum likelihood? Multivariate Behavioral Research 2006;41(4):499-532.

25. Peugh J, Fan X. How Well Does Growth Mixture Modeling Identify Heterogeneous Growth Trajectories? A Simulation Study Examining GMM's Performance Characteristics. Structural Equation Modeling: A Multidisciplinary Journal 2012;19(2):204-226.

26. Elsensohn M-H, Klich A, Ecochard R, Bastard M, Genolini C, Etard J-F, et al. A graphical method to assess distribution assumption in group-based trajectory models. Statistical methods in medical research 2016;25(2):968-982.

27. Shah NH, Hipwell AE, Stepp SD, Chang CC. Measures of discrimination for latent group-based trajectory models. J Appl Stat 2015;42(1):1-11.

28. Saunders JM. Understanding Random Effects in Group-Based Trajectory Modeling: An Application of Moffitt's Developmental Taxonomy. J Drug Issues 2010;40(1):195-220.

29. Belgrave D, Henderson J, Simpson A, Buchan I, Bishop C, Custovic A. Disaggregating asthma: Big investigation versus big data. J Allergy Clin Immunol 2017;139(2):400-407.

30. Simmonds M, Burch J, Llewellyn A, Griffiths C, Yang H, Owen C, et al. The use of measures of obesity in childhood for predicting obesity and the development of obesity-

For peer review only - http://bmjopen.bmj.com/site/about/guidelines.xhtml

related diseases in adulthood: a systematic review and meta-analysis. Health Technol Assess 2015;19(43):1-336.

For peer review only

For peer review only - http://bmjopen.bmj.com/site/about/guidelines.xhtml

Table 1 Framework of eight steps to construct a latent class trajectory model

Step	Step description	Criteria for selection
1	Scope model by provisionally selecting a plausible number of classes based on available literature and the structure based on plausible clinical patterns	Examine linearity of the shape of standardised residual plots for each of the classes in a model with no random-effects
2	Refine the model from step 1 to confirm the optimal number of classes, typically testing K = 1 to 7 classes	Lowest Bayesian Information Criteria (BIC) value
3	Refine optimal model structure from fixed- through to unrestricted random-effects of the model using the favoured K derived in step 2	
4	Run model adequacy assessments as described in Table S3 including Posterior Probability of Assignments (APPA); Odds of Correct Classification (OCC); and relative entropy	i) APPA: average of maximum probabilities should be greater than 70% for all classes<br>ii) OCC values greater than 5.0<br>iii) Relative entropy values greater than 0.5
5	Investigate graphical presentation	i) Plot mean trajectories across time for each class in a single graph<br>ii) Plot mean trajectories with 95% predictive intervals for each class (one class per graph)<br>iii) Plot individual class 'spaghetti plots' across time for a random sample
6	Run additional tools to assess discrimination including Degrees of Separation and Elsensohn's envelope of residuals	i) DoS greater than zero<br>ii) Envelope of residuals is assessed in plots by observing clear separations between classes
7	Assess for clinical characterisation and plausibility	i) Tabulation of characteristics by latent classes. Are the trajectory patterns clinically meaningful? Perhaps, consider classes with a minimum percentage of the population.<br>ii) Are the trajectory patterns clinically plausible?<br>iii) Concordance of class characteristics with those for other well-established variables
8	Conduct sensitivity analyses – for example, testing models without complete data at all time points	General assessment of patterns of trajectories compared with main model

For peer review only

For peer review only - http://bmjopen.bmj.com/site/about/guidelines.xhtml

Table 2 Number of classes (*K* = 1 to 7) using random-effects quadratic structure model F (proportional covariance structure) by gender

Model	*K*	Number of parameters	BIC	Proportions per class %						
				Class I	Class II	Class III	Class IV	Class V	Class VI	Class VII
**MEN**										
**Model F**	1	10	*	100						
	2	15	3324009	83	17					
	3	20	3310908	62	32	3				
	4	25	3324128	100	0	0	0			
	5	30	**3301301**	68	25	4	3	0.4		
	6	35	*							
	7	40	*							
**WOMEN**										
**Model F**	1	10	*	100						
	2	15	2195386	86	14					
	3	20	2179080	58	34	8				
	4	25	2179137	100	0	0	0			
	5	30	**2169791**	41	32	21	4	2		
	6	35	*							
	7	40	*							

* Models failed to converge
BIC: Bayesian Information Criteria
Results from random-effects quadratic structure Model G (unrestricted) by gender are shown in supplemental material TS2.

For peer review only

For peer review only - http://bmjopen.bmj.com/site/about/guidelines.xhtml

Table 3 Model adequacy assessments of latent trajectory class models based on different assumptions for K = 5 classes, by gender in the NIH-AARP cohort

Model	Description	BIC	Proportion per class %	Average Posterior Probability Assignment (APPA)	Relative Entropy E_k	Degree of Separation DoS_k
**Men**						
A	Homoscedastic	3476322	51: 22: 21: 6: 1	83: 84: 85: 91: 95	0.78	0.10
B	Heteroscedastic	3511646	41: 26: 20: 7: 6	85: 82: 84: 89: 86	-	0.15
C	Random intercept	3364856	68: 24: 4: 3: 2	90: 82: 89: 85: 82	0.81	0.34
D	Random slope	3325463	63: 19: 13: 3: 4	72: 70: 74: 83: 83	0.59	0.26
E	Random quadratic, Equal	*				
F	Random quadratic, Proportional	**3301301**	68: 25: 4: 3: 0.4	81: 74: 87: 83: 74	0.68	0.36
G	Random quadratic, Unrestricted	3320005	56: 30: 8: 5: 0.4	74: 70: 80: 79: 88	0.63	0.33
**Women**						
A	Homoscedastic	2289509	45: 36: 13: 4: 1	90: 84: 88: 93: 96	0.83	0.14
B	Heteroscedastic	2238444	32: 30: 15: 15: 8	87: 83: 85: 89: 91	-	0.05
C	Random intercept	2240681	52: 35: 9: 2: 2	89: 82: 86: 89: 91	0.79	0.21
D	Random slope	2193155	79: 12: 5: 2: 2	91: 80: 79: 86: 91	0.82	0.34
E	Random quadratic, Equal	2188224	95: 5: 0: 0: 0	49: 88: 0: 0: 0	0.26	0.05
F	Random quadratic, Proportional	**2169793**	41: 33: 21: 3: 2	74: 79: 80: 83:84	0.66	0.09
G	Random quadratic, Unrestricted	2187707	67: 23: 6: 3: 1	84: 77: 84:82: 87	0.73	0.34

*Failed to converge

For peer review only

Table 4 Latent class characteristics of 177,453† men and 111,503† women in the NIH-AARP cohort

	Model F (favoured model)				
	**Class I** Stable normal weight	**Class II** Normal weight to overweight	**Class III** Normal weight to obese	**Class IV** Overweight to obese	**Class V** Rapid increase to obese
**MEN**					
Number of participants, N	120 867 (68.1%)	44 383 (25.0%)	6 723 (3.8%)	4 764 (2.7%)	718 (0.4%)
Mean (sd) BMI at 18 years	20.75 (0.01)	23.92 (0.02)	21.69 (0.04)	27.16 (0.09)	24.76 (0.2)
Mean (sd) BMI at 35 years	23.35 (0.01)	26.72 (0.01)	30.24 (0.04)	26.61 (0.09)	37.69 (0.26)
Mean (sd) BMI at 50 years	24.66 (0.01)	29.12 (0.02)	31.34 (0.05)	30.95 (0.12)	35.01 (0.35)
Mean (sd) entry age, years	62.88 (0.01)	61.59 (0.03)	62.03 (0.06)	59.71 (0.07)	57.73 (0.18)
Mean (sd) current (at baseline) BMI, kg/m^2	25.39 (0.01)	30.84 (0.02)	29.07 (0.05)	35.14 (0.12)	30.51 (0.28)
Mean (sd) waist circumference, cm	94.38 (0.02)	106.23 (0.05)	102.73 (0.13)	115.24 (0.28)	105.91 (0.64)
Smoking, %					
Current	11 823 (10%)	3 761 (8%)	638 (9%)	460 (10%)	106 (15%)
Former	67 948 (56%)	26 898 (61%)	3 809 (57%)	3 002 (63%)	370 (52%)
Never	37 149 (31%)	12 130 (27%)	2 012 (30%)	1 110 (23%)	218 (30%)
Missing	3 946 (3%)	1 594 (4%)	264 (4%)	191 (4%)	24 (3%)
Mean (sd) alcohol consumption per day, g	18.69 (0.13)	17.45 (0.21)	15.48 (0.56)	15.57 (0.69)	10.22 (1.14)
**WOMEN**					
Number of participants, N	36 311 (32.6%)	45 832 (41.1%)	23 544 (21.1%)	3 898 (3.5%)	1 918 (1.7%)
Mean (sd) entry age, years	62.48 (0.03)	62.23 (0.02)	61.07 (0.03)	59.61 (0.09)	59.69 (0.12)
Mean (sd) BMI at 18 years	19.3 (0.01)	20.44 (0.01)	22.61 (0.02)	29.01 (0.09)	23.97 (0.1)
Mean (sd) BMI at 35 years	20.26 (0.01)	22.53 (0.01)	25.51 (0.02)	28.38 (0.1)	36.92 (0.16)
Mean (sd) BMI at 50 years	21.16 (0.01)	24.77 (0.01)	29.52 (0.03)	33.51 (0.13)	37.69 (0.2)
Mean (sd) current (at baseline) BMI, kg/m^2	21.97 (0.01)	26.66 (0.01)	32.4 (0.03)	37.47 (0.15)	34.4 (0.19)
Mean (sd) waist circumference, cm	76.19 (0.04)	86.66 (0.05)	97.82 (0.09)	106.29 (0.32)	101.96 (0.41)
Smoking, %					
Current	5803 (16%)	5660 (12%)	2733 (12%)	495 (13%)	214 (11%)
Former	14173 (39%)	18557 (40%)	9748 (41%)	1764 (45%)	731 (38%)
Never	15313 (42%)	20290 (44%)	10417 (44%)	1507 (39%)	909 (47%)
Missing	1022 (3%)	1325 (3%)	646 (3%)	132 (3%)	64 (3%)
Mean (sd) alcohol consumption per day, g	8.07 (0.1)	6.26 (0.08)	4.47 (0.11)	3.92 (0.29)	3.4 (0.48)

For peer review only - http://bmjopen.bmj.com/site/about/guidelines.xhtml

Hormone Therapy Use, %					
Ever	13989 (39%)	20206 (44%)	12215 (52%)	2241 (57%)	1092 (57%)
Never	22322 (61%)	25626 (56%)	11329 (48%)	1657 (43%)	826 (43%)

sd: standard deviation; †Exclusions include 35 women and 2 men with biologically implausible cancers from classes in proportions (2,16,12,0,5) and (1,0,0,1,0) respectively.

For peer review only

Table 5 Concurrence between BMI categories and classes in model F from the NIH-AARP cohort

Latent class	N	BMI categories (kg/m^2) < 18.5	18.5 to 24.9	25.0 to 29.9	30.0 to 34.9	>35.0
			**MEN**			
	177453	875	53466	86967	28234	7911
I	120866	312	50171	68646	1736	1
II	44383	319	1860	14097	23472	4635
III	6723	47	859	3335	1943	539
IV	4763	172	437	679	917	2558
V	718	25	139	210	166	178
			**Cohen κ_ω = 0.182 (0.181,0.183)**			
			**WOMEN**			
	111503	1327	48273	36025	16245	9633
I	36311	1215	33371	1725	0	0
II	45832	3	12584	28636	4607	2
III	23544	83	1683	4777	10587	6414
IV	3898	14	382	519	614	2369
V	1918	12	253	368	437	848
			**Cohen κ_ω= 0.52 (0.51,0.53)**			

BMI: body mass index; κ_ω weighted

For peer review only

FIGURE LEGENDS

Figure 1 BMI mean trajectories by men (left) and women (right) for models A-G. Colours are used to discriminate classes within each plot, but should not be used for direct comparisons across plots.

Figure 2 Illustration of the local (Elsensohn) residual envelope plots (shown here for model B)

For peer review only

For peer review only - http://bmjopen.bmj.com/site/about/guidelines.xhtml

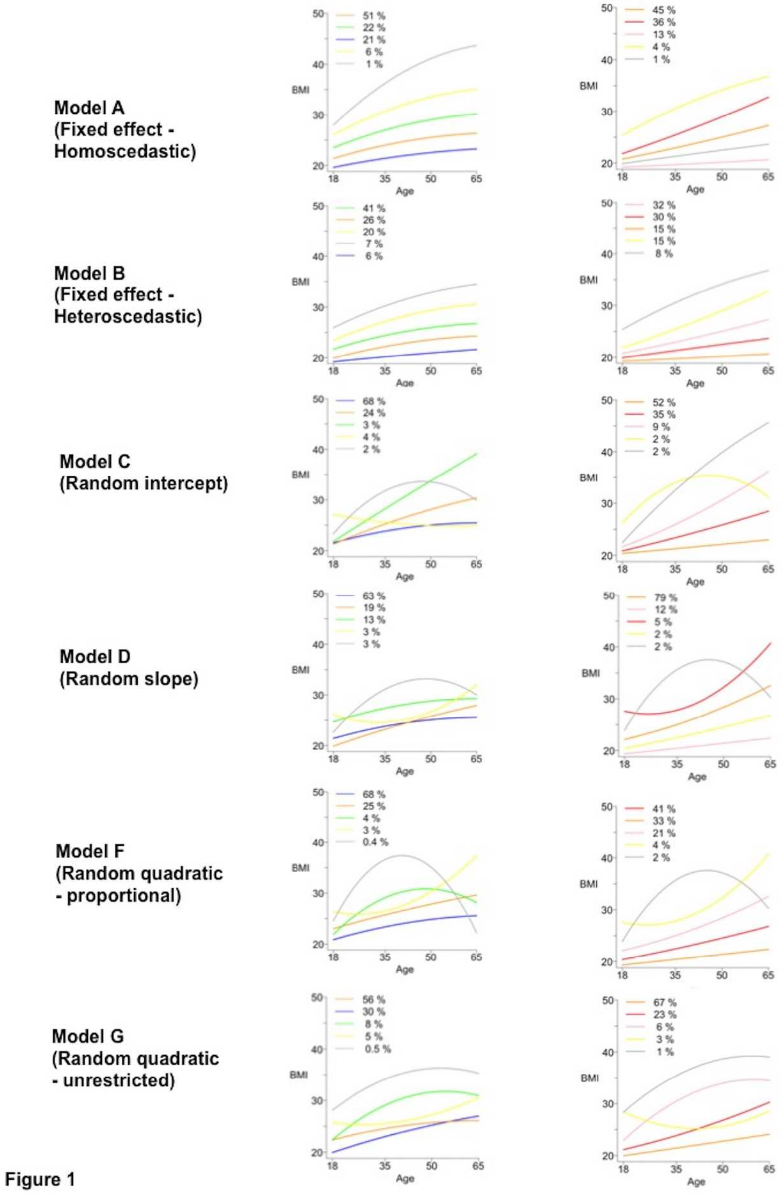

BMI mean trajectories by men (left) and women (right) for models A-G. Colours are used to discriminate classes within each plot, but should not be used for direct comparisons across plots.

254x366mm (300 x 300 DPI)

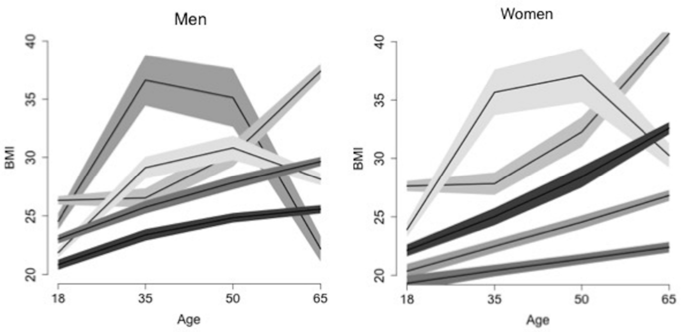

Figure 2

Illustration of the local (Elsensohn) residual envelope plots

254x366mm (300 x 300 DPI)

SUPPLEMENTAL MATERIAL

A framework to construct and interpret latent class trajectory modelling

Hannah Lennon[1,3] Scott Kelly,[2] Matthew Sperrin,[3] Iain Buchan,[3] Amanda J Cross,[4] Michael Leitzmann,[5] Michael B Cook,[2] Andrew G Renehan[1,3, 6]

For peer review only

Table S1 Different models with increasing complexity, associated software and referenced use in literature

**Model**	**Description**	**Interpretation**	**Software** *Command*	**References**
A	Fixed effects homoscedastic (common residual variance across classes)	No random effects – with the interpretation that any deviation of an individuals trajectory from its mean class trajectory is due to random error only	SAS traj *PROC TRAJ*	(1-5)
B	Fixed effects heteroscedastic (class-specific residual variances)	The same interpretation as Model A with random errors that can be larger and smaller in different classes.	R mmlcr *mmlcr*	(6)
C	Random intercept	The interpretation is allowing individuals to vary in initial weight but each class member is assumed to follow the same shape and magnitude of the mean trajectory	SAS traj *PROC TRAJ*	(7)
D	Random slope	Allowing individuals to vary in initial weight and slope of the mean trajectory but same curvature as trajectory	SAS traj *PROC TRAJ*	(7)
E	Random quadratic – Common variance structure across classes	Additional freedom of allowing individuals to vary within classes by initial weight, shape and magnitude, however each class is assumed to have the same amount of variability	R lcmm *hlme/lcmm*	
F	Random quadratic – Proportionality constraint to allow variance structures to vary across classes	Increasing flexibility of model E as variance structures are allowed to differ up to a multiplicative factor to allow some classes to have larger or smaller within-class variances. This model is can be thought of more parsimonious version of model G from (reducing the number of variance-covariance parameters to be estimated from 6xK parameters to 6+(K-1) parameters.	R lcmm *hlme/lcmm*	
G	Random quadratic – Class-specific variance structure (unstructured)	The most flexible model in which each class has its own separate random quadratic variance structure to describe its own within-class variability. Statistically this permits the variance and covariance of the intercept, slope and quadratic term to vary freely across all classes.	SAS traj *PROC TRAJ*	

[1]The SAS traj package has been converted for Stata users as the traj command in Stata (Collage Station, TX, USA).

For peer review only - http://bmjopen.bmj.com/site/about/guidelines.xhtml

More detailed description of model structure

For N individuals, the latent class trajectory model we consider is given by:

$$BMI_{itk} = \beta_0^k + \beta_1^k AGE_t + \beta_2^k AGE_t^2 + b_0^k + b_1^k AGE_t + b_2^k AGE_t^2 + \epsilon_t, \qquad \text{(Equation 1)}$$

where BMI_{itk} is the BMI of individual $i = 1, \ldots, N$, at time $t = 1, \ldots, T$ in class $k = 1, \ldots, K$. The random effect b^k is class-specific and follows a multivariate Normal distribution with zero mean and a 3×3 variance-covariance matrix B. For the residual error term ϵ_t, the usual assumption hold, ϵ_t is normally distributed with zero mean and variance σ^2. The probability of an individual belonging to class k is described by a multinomial distribution, i.e.,

$$Pr(individual\ i\ belonging\ to\ class\ k) = \frac{e^{\pi_k}}{\sum_{k=1}^{K} e^{\pi_k}} = \frac{e^{\pi_k}}{e^{\pi_1} + \cdots + e^{\pi_K}}, \qquad \text{(Equation 2)}$$

such that π_k are parameters to be estimated in the model. To select the number of latent classes, we assume a working model (Equation 1) for the random effect structure and the criterion used to select the number of classes was the lowest Bayesian Information Criteria (BIC).

More detailed description of model adequacy assessment

Common tools for model adequacy assessment checking are described below in Table S2. Here we give more details of the two extensions of these tools; degree of separation and Elsensohn's residuals (to random effects).

Degrees of separation

A model's ability to detect classes accurately is affected by the degree of separation between latent trajectory curves (8, 9). To describe the separation of latent growth curves, we used the multivariate Mahalanobis distance with the multivariate Mahalanobis distance (D) units defined as:

$$D_{ij} = (\mu_i - \mu_j)^T \Sigma^{-1} (\mu_i - \mu_j), \qquad i, j = 1, \ldots K,$$

For peer review only - http://bmjopen.bmj.com/site/about/guidelines.xhtml

where μ_i is a $T\times1$ vector of mean values for class i, and Σ^{-1} is the inverse of a $T\times T$ matrix of sample covariances of at times $t = 1,\dots,T$. The larger the difference, the larger the separation between curves. Peugh and Fan (9) argue that it is reasonable to expect that it is easier to identify heterogeneous latent growth trajectories when the statistical separation distance among the subpopulations is larger than when the separation distance among the latent subpopulations is much smaller.

To give an overall measure of separation for each model, we propose a weighted sum of multivariate Mahalanobis distance matrix with weights being the estimated class proportions, $\hat{\pi}_\iota$.. Then the degree of separation DoS_K is defined as

$$DoS_K = \sum_{i=1}^{K}\sum_{j=1}^{i}\hat{\pi}_\iota\hat{\pi}_J D_{ij}\,, \quad i,j = 1,2,\dots,K.$$

Larger values of DoS_K indicate the mean trajectories are well separated while DoS_K is zero in the special case when all mean trajectories are identical. If the DoS_K value is small, then you may wish to consider a model with fewer classes.

Elsensohn's envelope of residuals

To check the model assumption in fixed effect latent class models, Elsenhohn et al. (6) suggest plotting the local standard deviations of the residuals to check the appropriateness of the model. With the assumption that the residuals are homogeneous over time. To check the appropriateness of each of our model assumptions, we extended their method to include random effects in the models. We compute the local standard deviations of the residuals using the following steps:

1) Compute the observed residuals r_{itk} for each subject i, at time t given the individual is in class k

Equation 1: $r_{itk} = y_{it} - \hat{y}_{itk} = y_{it} - x_i^T\beta^k - z_i^T b^k,$

For peer review only - http://bmjopen.bmj.com/site/about/guidelines.xhtml

where y_{itk} is the observed value for individual i at time t in class k and $\hat{y}_{itk}$ is the fitted value of BMI from our fitted model, here the random effect model. 2) Compute the class- and time-specific weighted local variance of the residuals, $Var_w[r_{itk}]$, with weights being p_{ik}, the posterior probabilities of individual i belonging to group k. #) Plot the upper and lower boundaries for the local standard deviations of the residuals

Equation 2: $b_{itk} = \mu_{tk} \pm \sqrt{Var_w[r_{itk}]}$,

$where\ \mu_{tk}$ is the mean value of class k at time t. 4) Plot the boundaries b_{itk} onto the mean trajectory plots.

The shape of the local standard deviation of the residuals indicates the appropriateness of the model assumptions; where non-parallel boundaries indicate heteroscedasticity of residuals suggesting poor model fit, and differing interval widths suggest across group variability may not be fully accounted for. We believe this complements the others metrics well. If the boundaries suggest a poor fit, you may consider a more complex random effect structure to be more suitable, for example, a higher order polynomial.

For peer review only

Figure S1 Standardised residual plots of fixed-effect model (model A) for each class justifying the selection of a random-effect structure in the scoping model

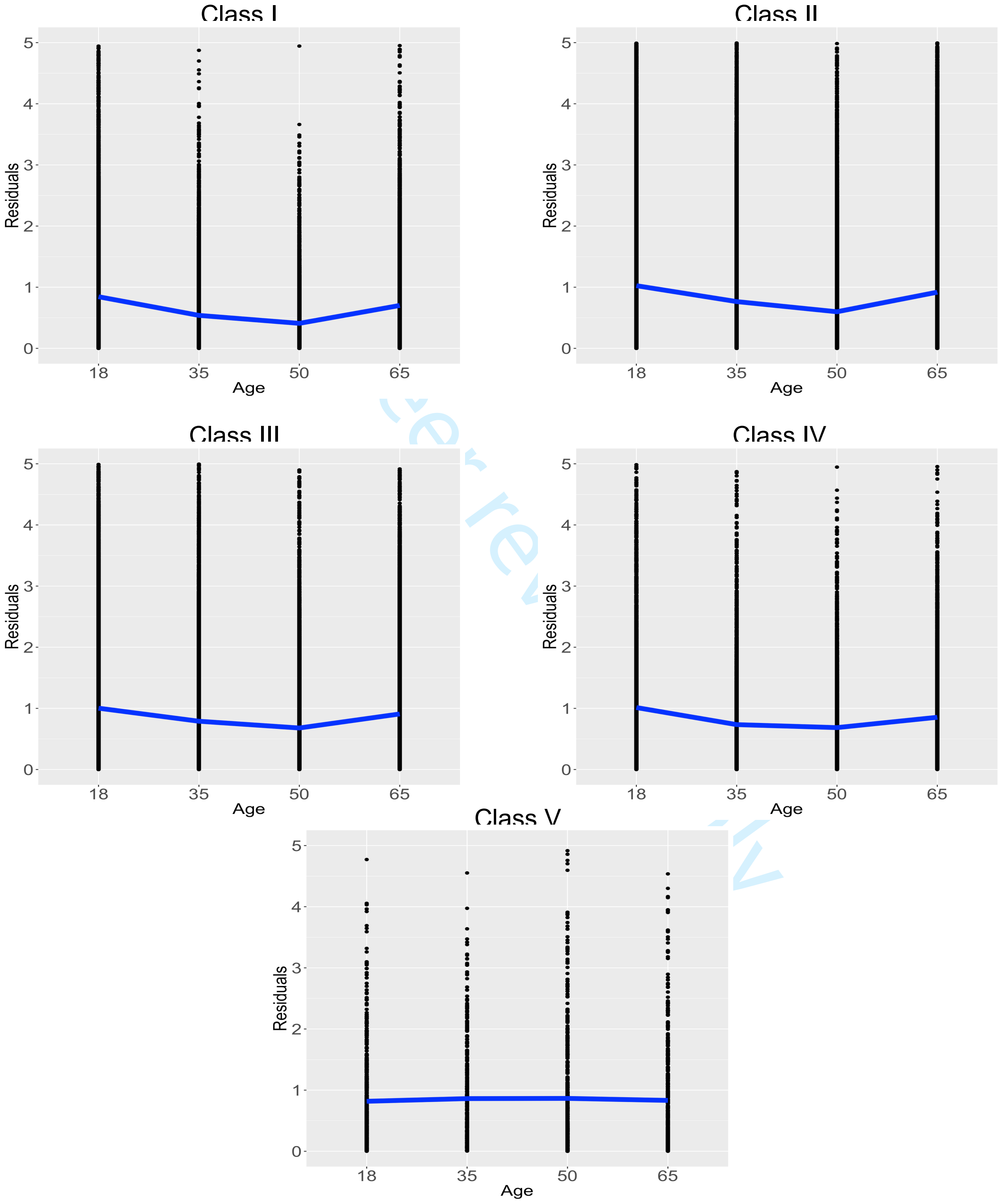

For peer review only - http://bmjopen.bmj.com/site/about/guidelines.xhtml

Table S2 Number of classes (K = 1† to 7) using random quadratic effect models: model G (unrestricted) by gender, NIH-AARP cohort

Model	K	Num of parameters	BIC	Proportions per class (%)						
				Class I	Class II	Class III	Class IV	Class V	Class VI	Class VII
**MEN**										
**Model G**	2	14	3361853	94	6					
	3	21	3341066	87	7	7				
	4	28	3329426	52	37	7	5			
	5	35	**3320005**	51	33	9	6	1		
	6	42	3315240	45	31	12	9	2	1	
	7	48	3311326	51	19	13	7	5	3	0.4
**WOMEN**										
**Model G**	2	14	2219825	89	11					
	3	21	2203726	61	34	0.5				
	4	28	2194293	61	32	5	2			
	5	35	**2187707**	60	29	7	3	1		
	6	42	2184412	63	19	10	4	1	0.5	
	7	48	*							

* Models failed to converge, † Models with K=1 failed to converge

For peer review only

Table S3 Common model selection tools for latent class trajectory models

Tool	Criteria for a good model	Description
Likelihood based measures	Lowest BIC, AIC,	These likelihood based model selection tools include: Bayesian Information Criteria (BIC), Akaike Information Criteria (AIC), Likelihood Ratio Tests (LRT), Lo-Mendel-Rubin (LMT-LRT), Bootstrap-LRT. (See Peugh and Fan, 2015 for discussion) (10)
APPA	APPA > 70% for each class	The average Posterior Probability Assignment, $\bar{p}_k$, looks at whether individuals are assigned with high probability and the overall average probability of assignment to each class.
Odds of correct classification	OCC > 5.0 for each class	The odds of correct classification (OCC) is the ratio of the odds of a correct classification into each group on the basis of the maximum probability classification rule and the estimated class membership proportions, $\widehat{\pi_k}$, $$OCC_k = \frac{\bar{p}_k/(1-\bar{p}_k)}{\widehat{\pi_k}/(1-\widehat{\pi_k})}.$$
Mismatch	Close to 0 for each class	The difference between the estimated class proportions and the class membership proportions once individuals have been assigned to a class, i.e. $$Mismatch_k = \widehat{\pi_k} - \frac{N_k}{N},$$ where N_k is the number of individuals in a class and N is the total number.
Entropy	Close to 0	Entropy is a global measure of classification uncertainty, which takes into account all $N \times K$ posterior probabilities. The entropy of a model is defined as $$E = -\sum_{i=1}^{N}\sum_{k=1}^{K} \hat{p}_{ik} \log \hat{p}_{ik},$$ which takes values from $[0, \infty)$, with higher values indicating a larger amount of uncertainty. Entropy values closest to 0 correspond to models with least classification uncertainty.
Relative Entropy	Close to 1	Relative entropy is a standardised version of entropy in the interval $[0,1]$ defined by $$E_K = 1 - \frac{E}{N \log K},$$ where values close to 1 indicate lowest classification uncertainty. In the special case when there is most uncertainty and each individual has equal probability of belonging to each class, $E_K = 0$. Jedidi et al., describes relative entropy as a relative measure of 'fuzziness', and suggested cause concern when close to zero, as this implies that the latent class centroids are not sufficiently separated (11).

For peer review only

Table S4 Concurrence between fixed effect (Model A) and random quadratic effect LCTM models (Model F and G) in men and women in the NIH-AARP cohort

Models	Cohen's κ_w (95% CI)	Concurrence						
**MEN**						Class		
**Model A and F**	0.31		Model F	I	II	II	IV	V
	(0.27,0.34)	Model A	177453	91106	38437	36430	10118	1362
		Class I	120866	81148	4961	34757	0	0
		Class II	44383	7826	28617	1035	6785	120
		Class III	6723	1405	3611	78	1491	138
		Class IV	4763	619	1076	553	1607	908
		Class V	718	108	172	7	235	196
			Model F	I	II	II	IV	V
		Model G	177453	99897	53372	14978	8320	886
**Model F and G**	0.57	Class I	120866	77962	41688	1117	99	0
	(0.56,0.59)	Class II	44383	21684	11464	6433	4796	6
		Class III	6723	219	10	6389	1	104
		Class IV	4763	17	210	746	3402	388
		Class V	718	15	0	293	22	388
**WOMEN**								
**Model A and F**	0.80		Model F	I	II	II	IV	V
	(0.80,0.80)	Model A	111503	50712	40080	14784	4840	1087
		Class I	36311	36311	0	0	0	0
		Class II	45832	13060	32383	389	0	0
		Class III	23544	1066	6881	13008	2589	0
		Class IV	3898	245	588	969	1501	595
		Class V	1918	30	228	418	750	492
**Model F and G**	0.65		Model F	I	II	II	IV	V
	(0.65,0.65)	Model G	111503	74481	25824	6929	3133	1136
		Class I	36311	36299	9	3	0	0
		Class II	45832	37334	8298	200	0	0
		Class III	23544	840	16896	4320	1463	25
		Class IV	3898	5	617	1098	1658	520
		Class V	1918	3	4	1308	12	591

For peer review only

Table S5 Kappa Matrices† between Models A to G in men and women in the NIH-AARP cohort

**Models**		**Cohen's Kappa Values (95% CI)**				
**MEN**		Model				
**Unweighted, κ**						
		Model B	Model C	Model D	Model F	Model G
	Model A	0.64 (0.64, 0.64)	0.20 (0.20, 0.21)	0.20 (0.19, 0.20)	0.38 (0.38, 0.38)	0.13 (0.12, 0.13)
	Model B		0.20 (0.20, 0.20)	0.19 (0.19, 0.19)	0.32 (0.31, 0.32)	0.16 (0.16, 0.17)
	Model C			0.59 (0.58, 0.59)	0.48 (0.47, 0.48)	0.43 (0.42, 0.43)
	Model D				0.50 (0.50, 0.50)	0.49 (0.48, 0.49)
	Model F					0.18 (0.18, 0.19)
**Weighted, κ_w**						
		Model B	Model C	Model D	Model F	Model G
	Model A	0.36 (0.34, 0.38)	0.43 (0.41, 0.44)	0.37 (0.34, 0.41)	0.31 (0.27, 0.34)	0.38 (0.36, 0.39)
	Model B		0.43 (0.42, 0.44)	0.39 (0.37, 0.41)	0.43 (0.42, 0.44)	0.39 (0.38, 0.4)
	Model C			0.49 (0.45, 0.52)	0.65 (0.65, 0.65)	0.62 (0.62, 0.62)
	Model D				0.71 (0.71, 0.71)	0.67 (0.67, 0.67)
	Model F					0.57 (0.56, 0.59)
**WOMEN**						
**Unweighted, κ**						
		Model B	Model C	Model D	Model F	Model G
	Model A	0.64 (0.64, 0.64)	0.65 (0.65, 0.66)	0.25 (0.24, 0.25)	0.63 (0.63, 0.63)	0.37 (0.37, 0.38)
	Model B		0.45 (0.44, 0.45)	0.17 (0.17, 0.17)	0.48 (0.47, 0.48)	0.30 (0.29, 0.30)
	Model C			0.26 (0.25, 0.26)	0.49 (0.49, 0.5)	0.41 (0.41, 0.41)
	Model D				0.28 (0.27, 0.28)	0.52 (0.52, 0.53)
	Model F					0.32 (0.31, 0.32)
**Weighted, κ_w**						
		Model B	Model C	Model D	Model F	Model G
	Model A	0.78 (0.78, 0.78)	0.69 (0.69, 0.69)	0.57 (0.57, 0.57)	0.80 (0.80, 0.80)	0.65 (0.65, 0.65)
	Model B		0.48 (0.48, 0.49)	0.42 (0.41, 0.42)	0.68 (0.68, 0.68)	0.45 (0.45, 0.46)
	Model C			0.64 (0.64, 0.64)	0.71 (0.71, 0.71)	0.63 (0.63, 0.63)
	Model D				0.64 (0.63, 0.66)	0.81 (0.81, 0.81)
	Model F					0.65 (0.65, 0.65)

† Kappa values presented here are optimised over the (5!=120) possible combinations of matching the classes labels across the models

For peer review only

Figure S2 Mean trajectories and 95% prediction intervals for each class in model F – men (left) and women (right)

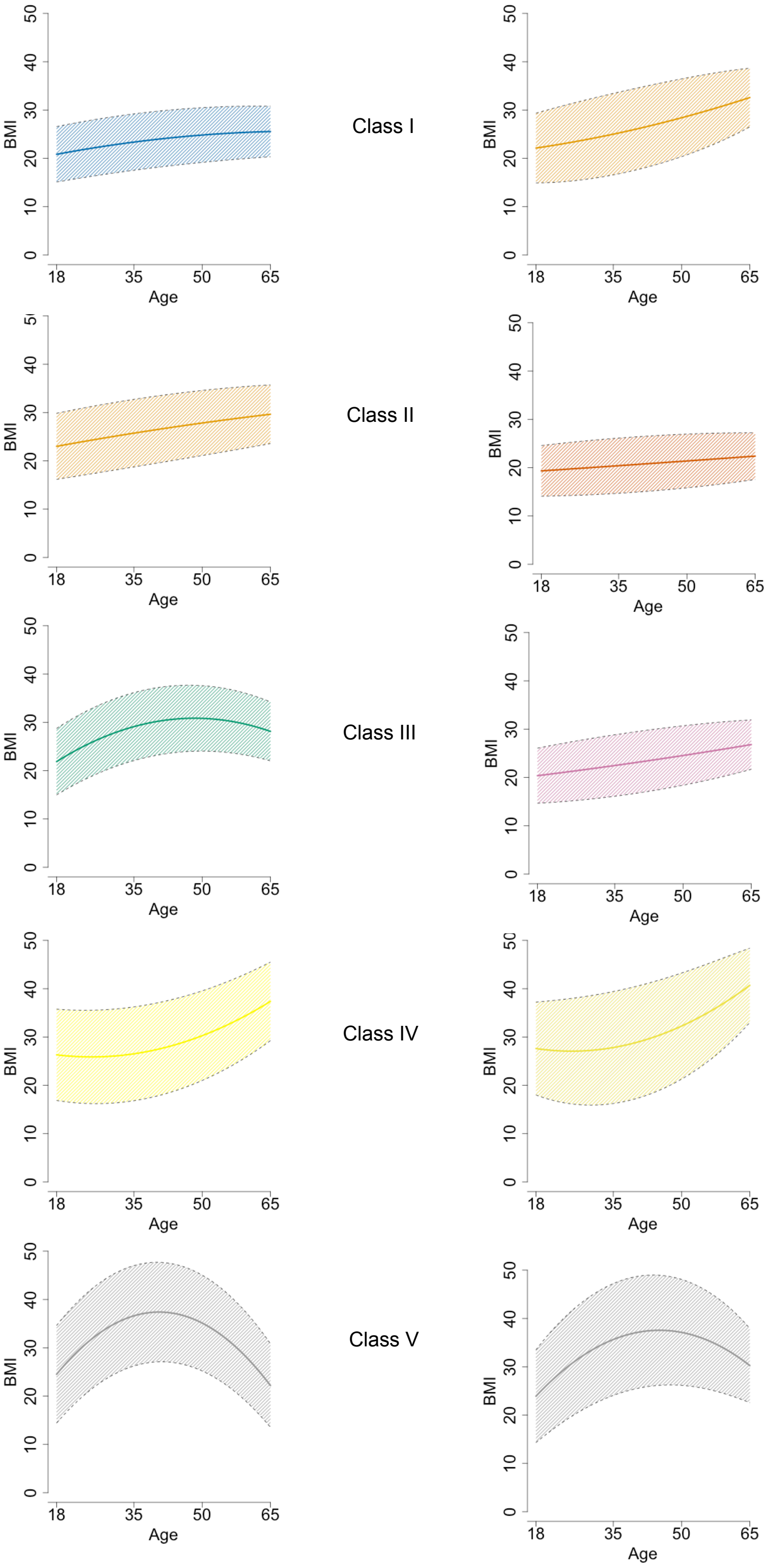

Figure S3 A randomly selected sample of 250 men from each class in model F

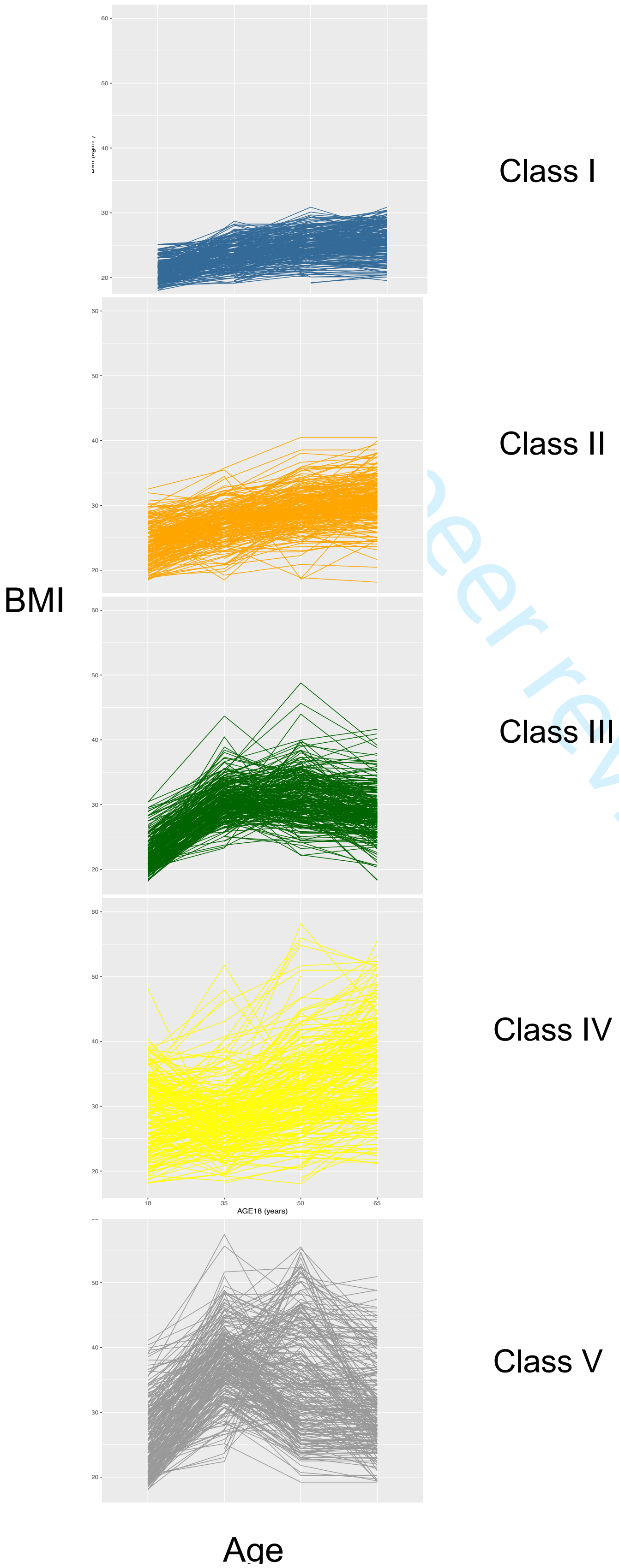

For peer review only

Table S5 Characteristics by five latent classes (Model F) of 177,453 men in the NIH-AARP cohort

	Model F (preferred model)				
	**Class I**	**Class II**	**Class III**	**Class IV**	**Class V**
	Stable normal weight	Normal weight to overweight	Normal to stable obese	Overweight to obese	Rapid increase to obese
Number of subjects, N	120 867 (68%)	44 383 (32%)	6 723 (5%)	4 764 (3%)	718 (0.5%)
Mean (sd) entry age, years	62.88 (0.01)	61.59 (0.03)	62.03 (0.06)	59.71 (0.07)	57.73 (0.18)
Mean (sd) current (at baseline) BMI, kg/m^2	25.39 (0.01)	30.84 (0.02)	29.07 (0.05)	35.14 (0.12)	30.51 (0.28)
Mean (sd) BMI at 50 years, kg/m^2	24.66 (0.01)	29.12 (0.02)	31.34 (0.05)	30.95 (0.12)	35.01 (0.35)
Mean (sd) BMI at 35 years, kg/m^2	23.35 (0.01)	26.72 (0.01)	30.24 (0.04)	26.61 (0.09)	37.69 (0.26)
Mean (sd) BMI at 18 years, kg/m^2	20.75 (0.01)	23.92 (0.02)	21.69 (0.04)	27.16 (0.09)	24.76 (0.2)
Mean (sd) maximum BMI, kg/m^2	26.94 (0.01)	32.76 (0.02)	34.09 (0.05)	37.04 (0.13)	40.17 (0.33)
Mean (sd) minimum BMI, kg/m^2	20.56 (0.01)	23.16 (0.02)	22.46 (0.05)	23.15 (0.07)	24.42 (0.20)
Mean (sd) waist circumference, cm	94.38 (0.02)	106.23 (0.05)	102.73 (0.13)	115.24 (0.28)	105.91 (0.64)
Mean (sd) hip circumference, cm	100.28 (0.02)	109.58 (0.05)	107.02 (0.13)	117.73 (0.25)	110.54 (0.62)
Mean (sd) hip-to-waist ratio,	0.94 (<0.01)	0.97 (<0.01)	0.96 (<0.01)	0.98 (<0.01)	0.96 (<0.01)
Mean (sd) height, m	1.78 (<0.01)	1.79 (<0.01)	1.79 (<0.01)	1.81 (<0.01)	1.78 (<0.01)
Comorbidity, %					
Diabetes	8036 (7%)	6155 (14%)	1999 (30%)	861 (18%)	281 (39%)
Heart disease	19927 (16%)	8709 (20%)	1890 (28%)	908 (19%)	165 (23%)
Smoking, %					
Current	11 823 (10%)	3 761 (8%)	638 (9%)	460 (10%)	106 (15%)
Former	67 948 (56%)	26 898 (61%)	3 809 (57%)	3 002 (63%)	370 (52%)
Never	37 149 (31%)	12 130 (27%)	2 012 (30%)	1 110 (23%)	218 (30%)
Missing	3 946 (3%)	1 594 (4%)	264 (4%)	191 (4%)	24 (3%)
Mean (sd) alcohol consumption, g/day	18.69 (0.13)	17.45 (0.21)	15.48 (0.56)	15.57 (0.69)	10.22 (1.14)
Race, %					
Non-Hispanic White	113 685 (94%)	41 819 (94%)	6 303 (94%)	4 447 (93%)	668 (93%)
Hispanic	2 194 (2%)	1 132 (3%)	157 (2%)	145 (3%)	19 (3%)
Black	1 922 (2%)	650 (1%)	129 (2%)	60 (1%)	14 (2%)
Asian, Pacific islander, American Indian, or Alaskan	2 023 (2%)	343 (1%)	48 (1%)	50 (1%)	4 (1%)

For peer review only - http://bmjopen.bmj.com/site/about/guidelines.xhtml

For peer review only

Missing	1 042 (1%)	439 (1%)	86 (1%)	61 (1%)	13 (2%)
Highest Education level achieved, %	5 508 (5%)	2 439 (5%)	415 (6%)	301 (6%)	37 (5%)
Less than 8 years old	16 768 (14%)	6 993 (16%)	1 281 (19%)	813 (17%)	139 (19%)
Completed primary	11 284 (9%)	4 324 (10%)	737 (11%)	474 (10%)	82 (11%)
Completed high school	25 929 (21%)	10 708 (24%)	1 674 (25%)	1 239 (26%)	197 (27%)
Post high school or come college	61 377 (51%)	19 919 (45%)	2 616 (39%)	1 936 (41%)	263 (37%)
College graduate or postgraduate	5 508 (5%)	2 439 (5%)	415 (6%)	301 (6%)	37 (5%)
Vigorous physical activity three or more times per week in the last year, %	91 250 (75%)	29 941 (67%)	4 670 (69%)	2 600 (55%)	438 (61%)
**Nutrition**					
At least 3 fruit servings per day, %	88 699 (73%)	31 603 (71%)	4 855 (72%)	3 335 (70%)	519 (72%)
At least 3 vegetable servings per day, %	56 255 (47%)	19 376 (44%)	3 245 (48%)	2 050 (43%)	364 (51%)
Mean (sd) red meat consumption, g/day	72.68 (0.18)	88.78 (0.37)	86.4 (0.99)	104.96 (1.44)	91.76 (3.6)
Mean (sd) calories per day, kcal	2010 (3)	2095 (5)	2089 (13)	2264 (20)	2146 (42)
Mean (sd) calories per day 10 years ago, kcal					
Mean (sd) calories per day age 12, kcal	1355 (2)	1404 (3)	1473 (8)	1491 (10)	1695 (30)
Drug use					
Ibuprofen use currently					
Aspirin use currently	38720 (32%)	15719 (35%)	2621 (39%)	1697 (36%)	266 (37%)
Fibre					
Mean (sd) fibre from diet age 12, mg/day	9.61 (0.01)	9.85 (0.02)	10.31 (0.07)	10.22 (0.08)	11.2 (0.23)
Mean (sd) fibre from diet 10 years ago, mg/day	6.95 (0.01)	6.95 (0.02)	7.43 (0.05)	7.26 (0.06)	7.71 (0.17)
Mean (sd) fibre CSFII, g/day	20.31 (0.03)	20.45 (0.05)	21.58 (0.14)	21.54 (0.19)	22.6 (0.48)
Mean (sd) fibre total NDSR, g/day	20.71 (0.03)	20.81 (0.05)	21.96 (0.14)	21.93 (0.19)	22.81 (0.5)
Calcium					
Mean (sd) calcium supplement, mg	152.09 (0.75)	136.56 (1.16)	143 (3.1)	138.13 (3.59)	159.2 (10.81)
Mean (sd) calcium from diet current, mg	808.96 (1.34)	845.53 (2.46)	874.55 (6.59)	906.76 (9.07)	910.57 (20.65)
Mean (sd) calcium from diet 10 years ago, mg					
Mean (sd) calcium from diet age 12, mg	653.16 (1.08)	653.03 (1.8)	681.39 (4.77)	672.61 (5.63)	734.68 (15.26)
Folate					
Mean (sd) folate supplement, µg/day	209.35 (0.67)	196.89 (1.13)	207.14 (2.92)	197.72 (3.49)	222.93 (9.74)
Mean (sd) folate pre 1998, µg/day	335.71 (0.48)	336.53 (0.83)	349.14 (2.29)	346.99 (2.85)	355.86 (7.87)

For peer review only - http://bmjopen.bmj.com/site/about/guidelines.xhtml

Mean (sd) folate NDSR, µg/day	447.13 (0.58)	451.13 (1.03)	463.45 (2.85)	470.15 (3.73)	482.46 (10.16)

sd: standard deviation

For peer review only

For peer review only - http://bmjopen.bmj.com/site/about/guidelines.xhtml

Table S6 Characteristics by five latent classes (Model F) of 111,503 women in the NIH-AARP cohort

	Model F (preferred model)				
	**Class I**	**Class II**	**Class III**	**Class IV**	**Class V**
	Stable normal weight	Normal weight to overweight	Normal weight to obese	Overweight to obese	Rapid increase to obese
Number of subjects, N	36 311 (32.7%)	45 832 (41.1%)	23 544 (21.1%)	3 898 (3.5%)	1 918 (1.7%)
Mean (sd) entry age, years	62.48 (0.03)	62.23 (0.02)	61.07 (0.03)	59.61 (0.09)	59.69 (0.12)
Mean (sd) current (at baseline) BMI, kg/m^2	21.97 (0.01)	26.66 (0.01)	32.4 (0.03)	37.47 (0.15)	34.4 (0.19)
Mean (sd) BMI at 50 years, kg/m^2	21.16 (0.01)	24.77 (0.01)	29.52 (0.03)	33.51 (0.13)	37.69 (0.2)
Mean (sd) BMI at 35 years, kg/m^2	20.26 (0.01)	22.53 (0.01)	25.51 (0.02)	28.38 (0.1)	36.92 (0.16)
Mean (sd) BMI at 18 years, kg/m^2	19.3 (0.01)	20.44 (0.01)	22.61 (0.02)	29.01 (0.09)	23.97 (0.1)
Mean (sd) maximum BMI, kg/m^2	23.29 (0.01)	28.13 (0.02)	34.72 (0.04)	40.73 (0.14)	43.18 (0.2)
Mean (sd) minimum BMI, kg/m^2	18.57 (0.01)	20.08 (0.01)	21.85 (0.02)	24.05 (0.07)	24.59 (0.11)
Mean (sd) waist circumference, cm	76.19 (0.04)	86.66 (0.05)	97.82 (0.09)	106.29 (0.32)	101.96 (0.41)
Mean (sd) hip circumference, cm	96.78 (0.03)	105.23 (0.04)	114.7 (0.08)	122.14 (0.31)	118.59 (0.42)
Mean (sd) hip-to-waist ratio,	0.78 (<0.01)	0.82 (<0.01)	0.84 (<0.01)	0.84 (<0.01)	0.85 (<0.01)
Mean (sd) height, m	1.65 (<0.01)	1.64 (<0.01)	1.63 (<0.01)	1.63 (<0.01)	1.62 (<0.01)
Comorbidity, %					
Diabetes	680 (2%)	2344 (5%)	3237 (14%)	725 (19%)	604 (31%)
Heart disease	2269 (6%)	3677 (8%)	2518 (11%)	447 (11%)	296 (15%)
Smoking, %					
Current	5803 (16%)	5660 (12%)	2733 (12%)	495 (13%)	214 (11%)
Former	14173 (39%)	18557 (40%)	9748 (41%)	1764 (45%)	731 (38%)
Never	15313 (42%)	20290 (44%)	10417 (44%)	1507 (39%)	909 (47%)
Missing	1022 (3%)	1325 (3%)	646 (3%)	132 (3%)	64 (3%)
Mean (sd) alcohol consumption, g/day	8.07 (0.1)	6.26 (0.08)	4.47 (0.11)	3.92 (0.29)	3.4 (0.48)
Race, %					
Non-Hispanic White	34468 (95%)	42261 (92%)	21178 (90%)	3446 (88%)	1745 (91%)

For peer review only

For peer review only - http://bmjopen.bmj.com/site/about/guidelines.xhtml

Hispanic	699 (2%)	1882 (4%)	1563 (7%)	293 (8%)	105 (5%)
Black	369 (1%)	739 (2%)	324 (1%)	58 (1%)	26 (1%)
Asian, Pacific islander, American Indian, or Alaskan	467 (1%)	443 (1%)	174 (1%)	35 (1%)	15 (1%)
Missing	308 (1%)	507 (1%)	305 (1%)	66 (2%)	27 (1%)
Highest Education level achieved, %					
Less than 8 years old	1155 (3%)	2151 (5%)	1457 (6%)	225 (6%)	124 (6%)
Completed primary	7578 (21%)	11545 (25%)	5984 (25%)	922 (24%)	524 (27%)
Completed high school	3700 (10%)	5021 (11%)	2771 (12%)	489 (13%)	258 (13%)
Post high school or come college	9801 (27%)	11877 (26%)	6088 (26%)	1038 (27%)	521 (27%)
College graduate or postgraduate	14077 (39%)	15238 (33%)	7244 (31%)	1224 (31%)	491 (26%)
Vigorous physical activity three or more times per week in the last year, %	25959 (71%)	30035 (66%)	12692 (54%)	1809 (46%)	1011 (53%)
**Nutrition**					
At least 3 fruit servings per day, %	28441 (78%)	35607 (78%)	17801 (76%)	2889 (74%)	1473 (77%)
At least 3 vegetable servings per day, %	22030 (61%)	27235 (59%)	13282 (56%)	2184 (56%)	1163 (61%)
Mean (sd) red meat consumption, g/day	40.46 (0.19)	46.46 (0.19)	54.41 (0.33)	60.98 (0.98)	56.86 (1.22)
Mean (sd) calories per day, kcal	1546 (4)	1557 (3)	1634 (5)	1727 (15)	1688 (20)
Mean (sd) calories per day 10years ago, kcal	817 (2)	853 (2)	889 (3)	901 (7)	961 (10)
Mean (sd) calories per day age 12, kcal	1250 (3)	1261 (3)	1290 (4)	1336 (10)	1356 (14)
Drug use					
Ibuprofen use currently	4250 (12%)	7065 (15%)	4953 (21%)	1025 (26%)	474 (25%)
Aspirin use currently	6627 (18%)	9559 (21%)	5221 (22%)	913 (23%)	492 (26%)
Hormone Therapy Use, %					
Ever	13989 (39%)	20206 (44%)	12215 (52%)	2241 (57%)	1092 (57%)
Never	22322 (61%)	25626 (56%)	11329 (48%)	1657 (43%)	826 (43%)
Fibre					
Mean (sd) fibre from diet 10years ago, mg/day	7.14 (0.02)	7.22 (0.02)	7.31 (0.03)	7.45 (0.06)	7.57 (0.09)
Mean (sd) fibre from diet age 12, mg/day	8.82 (0.02)	8.96 (0.02)	9.17 (0.03)	9.45 (0.08)	9.39 (0.11)
Mean (sd) fibre CSFII, g/day	18.01 (0.05)	17.75 (0.04)	18.04 (0.06)	18.78 (0.17)	19.14 (0.26)
Mean (sd) fibre total NDSR, g/day	18.05 (0.05)	17.78 (0.04)	18.08 (0.07)	18.85 (0.17)	19.17 (0.26)
Calcium					

For peer review only

Mean (sd) calcium supplement, mg/day	466.85 (2.53)	393.26 (2.1)	337.9 (2.79)	318.84 (6.84)	341.76 (10.22)
Mean (sd) calcium from diet current, mg/day	712.35 (2.23)	716.94 (1.96)	733.95 (2.97)	779.53 (8.16)	765.72 (10.81)
Mean (sd) calcium from diet 10 years ago, mg/day	384.29 (1.18)	387.85 (1.04)	392 (1.5)	390.64 (3.67)	411.4 (5.56)
Mean (sd) calcium from diet age 12, mg/day	677 (2.04)	659.92 (1.81)	650.42 (2.55)	652.48 (6.35)	651.64 (9.03)
Folate					
Mean (sd) folate supplement, µg/day	252.3 (1.23)	239.96 (1.1)	227.06 (1.54)	217.19 (3.78)	228.85 (5.44)
Mean (sd) folate pre 1998, µg/day	291.37 (0.79)	286.07 (0.67)	288.08 (1.02)	295.47 (2.65)	297.1 (3.77)
Mean (sd) folate NDSR, µg/day	375.26 (0.96)	371.15 (0.82)	377.73 (1.26)	389.45 (3.31)	391.35 (4.83)

sd: standard deviation

For peer review only

For peer review only - http://bmjopen.bmj.com/site/about/guidelines.xhtml

Table S7 Characteristics by BMI categories of 177,453 men in the AARP cohort

	Normal weight	Overweight	Obese I	Obese II	Obese III
Number of subjects, N (%)	53466 (30.1)	86967 (49.0)	28234 (15.9)	6033 (3.4)	1878 (1.1)
Mean (sd) entry age, years	62.83 (0.02)	62.47 (0.02)	61.87 (0.03)	61.16 (0.07)	60.48 (0.12)
Mean (sd) current (at baseline) BMI, kg/m^2					
Mean (sd) BMI at 50 years, kg/m^2	23.18 (0.01)	27.16 (0)	31.9 (0.01)	36.86 (0.02)	43.93 (0.1)
Mean (sd) BMI at 35 years, kg/m^2	23.28 (0.01)	26.25 (0.01)	29.74 (0.02)	33.33 (0.05)	38.15 (0.15)
Mean (sd) BMI at 18 years, kg/m^2	22.66 (0.01)	24.64 (0.01)	26.89 (0.02)	29.07 (0.05)	31.7 (0.12)
	20.65 (0.01)	21.75 (0.01)	23.14 (0.02)	24.46 (0.05)	25.92 (0.11)
Mean (sd) maximum BMI, kg/m^2					
Mean (sd) minimum BMI, kg/m^2	25.19 (0.01)	28.89 (0.01)	33.58 (0.02)	38.61 (0.05)	45.63 (0.14)
	20.05 (0.01)	21.47 (0.01)	22.85 (0.02)	24.17 (0.05)	25.57 (0.11)
Mean (sd) waist circumference, cm					
Mean (sd) hip circumference, cm	89.56 (0.03)	98.19 (0.03)	108.65 (0.05)	119.1 (0.12)	132.59 (0.29)
Mean (sd) hip-to-waist ratio,	96.92 (0.03)	103.26 (0.02)	111.41 (0.05)	120.44 (0.14)	132.79 (0.36)
Mean (sd) height, m	0.93 (0)	0.96 (0)	0.98 (0)	1 (0)	1 (0)
	70.23 (0.01)	70.07 (0.01)	70.18 (0.02)	69.93 (0.04)	69.7 (0.08)
Comorbidity, %					
Diabetes	3194 (6%)	7752 (9%)	4375 (15%)	1404 (23%)	555 (30%)
Heart disease	8624 (16%)	15314 (18%)	5788 (21%)	1312 (22%)	410 (22%)
Smoking, %					
Current	6513 (12%)	7345 (8%)	2219 (8%)	427 (7%)	129 (7%)
Former	27129 (51%)	51742 (59%)	17719 (63%)	3818 (63%)	1203 (64%)
Never	18089 (34%)	27129 (51%)	7278 (26%)	1557 (26%)	484 (26%)
Missing	1735 (3%)	2948 (3%)	1018 (4%)	231 (4%)	62 (3%)
Mean (sd) alcohol consumption per day, g	18.24 (0.19)	18.36 (0.15)	18.01 (0.28)	16.41 (0.62)	11.72 (0.88)
Race, %					
Non-Hispanic White	50172 (94%)	81972 (94%)	26548 (94%)	5675 (94%)	1738 (93%)
Hispanic	843 (2%)	1775 (2%)	777 (3%)	171 (3%)	68 (4%)
Black	750 (1%)	1448 (2%)	454 (2%)	86 (1%)	28 (1%)
Asian, Pacific islander, American Indian, or Alaskan	1222 (2%)	1001 (1%)	176 (1%)	34 (1%)	15 (1%)
Missing	479 (1%)	771 (1%)	279 (1%)	67 (1%)	29 (2%)

For peer review only

Highest Education level achieved, %					
Less than 8 years old	2136 (4%)	4259 (5%)	1695 (6%)	422 (7%)	148 (8%)
Completed primary	6591 (12%)	13066 (15%)	4805 (17%)	1075 (18%)	356 (19%)
Completed high school	4643 (9%)	8406 (10%)	2930 (10%)	639 (11%)	209 (11%)
Post high school or come college	10728 (20%)	19668 (23%)	7120 (25%)	1540 (26%)	498 (27%)
College graduate or postgraduate	29368 (55%)	41568 (48%)	11684 (41%)	2357 (39%)	667 (36%)
Vigorous physical activity three or more times per week in the last year, %	41480 (78%)	64458 (74%)	18280 (65%)	3284 (54%)	786 (42%)
**Nutrition**					
At least 3 fruit servings per day, %	40079 (75%)	62921 (72%)	19870 (70%)	4203 (70%)	1303 (69%)
At least 3 vegetable servings per day, %	32887 (62%)	49213 (57%)	15003 (53%)	3220 (53%)	1037 (55%)
Mean (sd) red meat consumption, g/day	66.09 (0.28)	77.79 (0.21)	93.13 (0.44)	107.21 (1.09)	124.23 (2.72)
Mean (sd) calories per day, kcal	1997.3 (3.96)	2019.26 (3.08)	2123.92 (6.21)	2244.87 (14.7)	2411.64 (34.3)
Mean (sd) calories per day 10 years ago, kcal	861.51 (1.57)	865.75 (1.28)	896.59 (2.4)	936.47 (5.47)	974.51 (10.19)
Mean (sd) calories per day age 12, kcal	667.55 (1.63)	650.14 (1.28)	645.21 (2.27)	657.3 (4.93)	667.25 (9.06)
Drug use					
Ibuprofen use currently,	3969 (15%)	8036 (17%)	3688 (23%)	973 (27%)	408 (36%)
Missing	27029 (51%)	39942 (46%)	11894 (42%)	2460 (41%)	743 (40%)
Aspirin use currently,	16456 (40%)	29091 (43%)	10360 (47%)	2174 (47%)	699 (50%)
Missing	12646 (24%)	18622 (21%)	6146 (22%)	1444 (24%)	469 (25%)
Fibre					
Mean (sd) fibre from diet age 12, mg	9.65 (0.02)	9.69 (0.02)	9.84 (0.03)	10.04 (0.07)	10.3 (0.13)
Mean (sd) fibre from diet 10 years ago, mg	7.12 (0.01)	6.89 (0.01)	6.95 (0.02)	7.1 (0.05)	7.35 (0.09)
Mean (sd) fibre CSFII, g	20.95 (0.05)	20.11 (0.03)	20.26 (0.06)	20.85 (0.14)	22.3 (0.28)
Mean (sd) fibre total NDSR, g	21.35 (0.05)	20.49 (0.03)	20.66 (0.07)	21.22 (0.15)	22.65 (0.29)
Calcium					
Mean (sd) calcium supplement, mg	811.3 (2)	812.92 (1.62)	852.6 (3.12)	906.94 (7.45)	973.28 (16.08)
Mean (sd) calcium from diet current, mg	163.44 (1.18)	144.71 (0.85)	131.79 (1.43)	127.03 (3.03)	122.25 (5.34)
Mean (sd) calcium from diet 10 years ago, mg	406 (1)	398.45 (0.79)	404.71 (1.45)	422.22 (3.28)	426.44 (5.87)
Mean (sd) calcium from diet age 12, mg	667.55 (1.63)	650.14 (1.28)	645.21 (2.27)	657.3 (4.93)	667.25 (9.06)
Folate					

For peer review only

Mean (sd) folate supplement, g	342.65 (0.74)	332.76 (0.56)	335.07 (1.04)	343.98 (2.3)	360.39 (4.58)
Mean (sd) folate pre 1998, g	217.7 (1.01)	205.22 (0.8)	191.35 (1.41)	186.39 (3.01)	180.32 (5.43)
Mean (sd) folate NDSR, g	455.75 (0.92)	443.69 (0.69)	449.63 (1.3)	464.98 (2.95)	490.92 (5.98)

sd: standard deviation
N=875 men classified as <18.0 kg/m^2 at study entry not included

For peer review only

Table S8 Characteristics by BMI categories of 111,503 women in the AARP cohort

	Normal weight	Overweight	Obese I	Obese II	Obese III
Number of subjects, N (%)	48273 (43.3)	36025 (32.2)	16245 (14.6)	6126 (5.5.)	3507 (3.1)
Mean (sd) entry age, years	61.96 (0.02)	62.15 (0.03)	61.89 (0.04)	61.21 (0.07)	60.45 (0.09)
Mean (sd) current (at baseline) BMI, kg/m^2	22.45 (0.01)	27.23 (0.01)	32.12 (0.01)	37.14 (0.02)	44.66 (0.08)
Mean (sd) BMI at 50 years, kg/m^2	22.04 (0.01)	25.31 (0.01)	28.75 (0.03)	32.46 (0.06)	38.33 (0.12)
Mean (sd) BMI at 35 years, kg/m^2	21.12 (0.01)	22.93 (0.01)	24.93 (0.03)	27.22 (0.06)	30.72 (0.11)
Mean (sd) BMI at 18 years, kg/m^2	20.11 (0.01)	20.83 (0.01)	21.88 (0.03)	23.03 (0.05)	24.46 (0.08)
Mean (sd) maximum BMI, kg/m^2	24.21 (0.01)	28.88 (0.02)	34.05 (0.03)	39.21 (0.06)	46.57 (0.11)
Mean (sd) minimum BMI, kg/m^2	19.16 (0.01)	20.3 (0.01)	21.44 (0.02)	22.57 (0.05)	24.24 (0.08)
Mean (sd) waist circumference, cm	76.99 (0.04)	88.19 (0.05)	97.92 (0.08)	106.84 (0.13)	120.12 (0.23)
Mean (sd) hip circumference, cm	97.47 (0.03)	106.73 (0.04)	115.24 (0.07)	124.36 (0.14)	137.66 (0.24)
Mean (sd) hip-to-waist ratio,	0.79 (0)	0.83 (0)	0.85 (0)	0.86 (0)	0.85 (0)
Mean (sd) height, m	1218 (3%)	2199 (6%)	2050 (13%)	1211 (20%)	873 (25%)
Comorbidity, %					
Diabetes	1218 (3%)	2199 (6%)	2050 (13%)	1211 (20%)	873 (25%)
Heart disease	3050 (6%)	3043 (8%)	1749 (11%)	779 (13%)	482 (14%)
Smoking, %					
Current	7699 (16%)	4467 (12%)	1584 (10%)	502 (8%)	256 (7%)
Former	18632 (39%)	14883 (41%)	6843 (42%)	2633 (43%)	1586 (45%)
Never	20558 (43%)	15636 (43%)	7380 (45%)	2823 (46%)	1552 (44%)
Missing	1384 (3%)	1039 (3%)	438 (3%)	168 (3%)	113 (3%)
Mean (sd) alcohol consumption per day, g	7.94 (0.09)	5.92 (0.09)	4.3 (0.13)	3.25 (0.21)	2.67 (0.26)
Race, %					
Non-Hispanic White	45651 (95%)	33005 (92%)	14627 (90%)	5471 (89%)	3090 (88%)
Hispanic	988 (2%)	1688 (5%)	1067 (7%)	472 (8%)	299 (9%)
Black	582 (1%)	581 (2%)	225 (1%)	74 (1%)	45 (1%)
Asian, Pacific islander, American Indian, or Alaskan	620 (1%)	333 (1%)	114 (1%)	32 (1%)	16 (0%)
Missing	432 (1%)	418 (1%)	212 (1%)	77 (1%)	57 (2%)

For peer review only - http://bmjopen.bmj.com/site/about/guidelines.xhtml

Highest Education level achieved, %					
Less than 8 years old	1585 (3%)	1797 (5%)	1017 (6%)	417 (7%)	261 (7%)
Completed primary	10367 (21%)	9077 (25%)	4288 (26%)	1604 (26%)	954 (27%)
Completed high school	4914 (10%)	4138 (11%)	1869 (12%)	740 (12%)	459 (13%)
Post high school or come college	12696 (26%)	9375 (26%)	4327 (27%)	1644 (27%)	938 (27%)
College graduate or postgraduate	18711 (39%)	11638 (32%)	4744 (29%)	1721 (28%)	895 (26%)
Vigorous physical activity three or more times per week in the last year, %	34629 (72%)	23170 (64%)	8917 (55%)	2694 (44%)	1241 (35%)
**Nutrition**	37782 (78%)	27905 (77%)	12355 (76%)	4596 (75%)	2559 (73%)
At least 3 fruit servings per day, %	34422 (71%)	24747 (69%)	10907 (67%)	3921 (64%)	2257 (64%)
At least 3 vegetable servings per day, %					
Mean (sd) red meat consumption, g/day	40.35 (0.17)	47.73 (0.22)	54.09 (0.39)	61.25 (0.67)	71.18 (1.07)
Mean (sd) calories per day, kcal	1531.86 (2.99)	1568.13 (3.81)	1626.68 (6.33)	1696.9 (10.28)	1864.32 (16.6)
Mean (sd) calories per day 10years ago, kcal	818.42 (1.6)	859.29 (2.01)	894.97 (3.17)	910.64 (5.15)	962.24 (7.27)
Mean (sd) calories per day age 12, kcal	673.14 (1.77)	658.24 (2.05)	650.22 (3.07)	646.33 (5.04)	657.19 (6.67)
Hormone Therapy Use, %					
Never	18932 (39%)	16154 (45%)	8334 (51%)	3459 (56%)	2261 (64%)
Ever	29341 (61%)	19871 (55%)	7911 (49%)	2667 (44%)	1246 (36%)
Ibuprofen use currently,	5842 (21%)	5795 (26%)	3352 (33%)	1566 (39%)	1098 (47%)
Ibuprofen use missing	20318 (42%)	14129 (39%)	6018 (37%)	2075 (34%)	1191 (34%)
Aspirin use currently,	8875 (28%)	7709 (32%)	3714 (36%)	1445 (38%)	833 (41%)
Asprin use missing	16220 (34%)	12185 (34%)	5777 (36%)	2364 (39%)	1483 (42%)
,					
Fibre					
Mean (sd) fibre from diet 10years ago, mg	8.82 (0.02)	9.01 (0.02)	9.14 (0.04)	9.32 (0.06)	9.5 (0.08)
Mean (sd) fibre from diet age 12, mg	7.14 (0.02)	7.22 (0.02)	7.36 (0.03)	7.34 (0.05)	7.65 (0.07)
Mean (sd) fibre CSFII, g	18 (0.04)	17.74 (0.05)	17.94 (0.08)	18 (0.12)	19.35 (0.18)
Mean (sd) fibre total NDSR, g	18.05 (0.04)	17.77 (0.05)	17.98 (0.08)	18.05 (0.12)	19.43 (0.18)
Calcium					
Mean (sd) calcium supplement, mg	710.52 (1.9)	719.2 (2.26)	733.66 (3.61)	751.64 (5.75)	808.42 (8.73)
Mean (sd) calcium from diet current, mg	458.8 (2.18)	382.25 (2.35)	333.6 (3.31)	307.85 (5.3)	270.5 (6.54)
Mean (sd) calcium from diet 10 years ago, mg	382.09 (1.01)	388.3 (1.19)	397.37 (1.82)	397.86 (2.95)	414.1 (4.09)

For peer review only - http://bmjopen.bmj.com/site/about/guidelines.xhtml

Mean (sd) calcium from diet age 12, mg	673.14 (1.77)	658.24 (2.05)	650.22 (3.07)	646.33 (5.04)	657.19 (6.67)
Folate					
Mean (sd) folate supplement, g	250.24 (1.07)	239.5 (1.25)	225.57 (1.84)	217.96 (3.02)	209.48 (4.03)
Mean (sd) folate pre 1998, g	290.16 (0.68)	285.89 (0.78)	287.43 (1.2)	288.09 (1.98)	304.36 (2.79)
Mean (sd) folate NDSR, g	373.99 (0.82)	371.32 (0.96)	376.02 (1.49)	380.5 (2.43)	407.36 (3.5)

sd: standard deviation
N=1327 Women classified as <18.0 kg/m^2 at study entry not included

For peer review only

Figure S4 Latent Class trajectories derived on individuals with at least three time points, with random quadratic effect proportional specification (model F) (left: Men; right: Women)

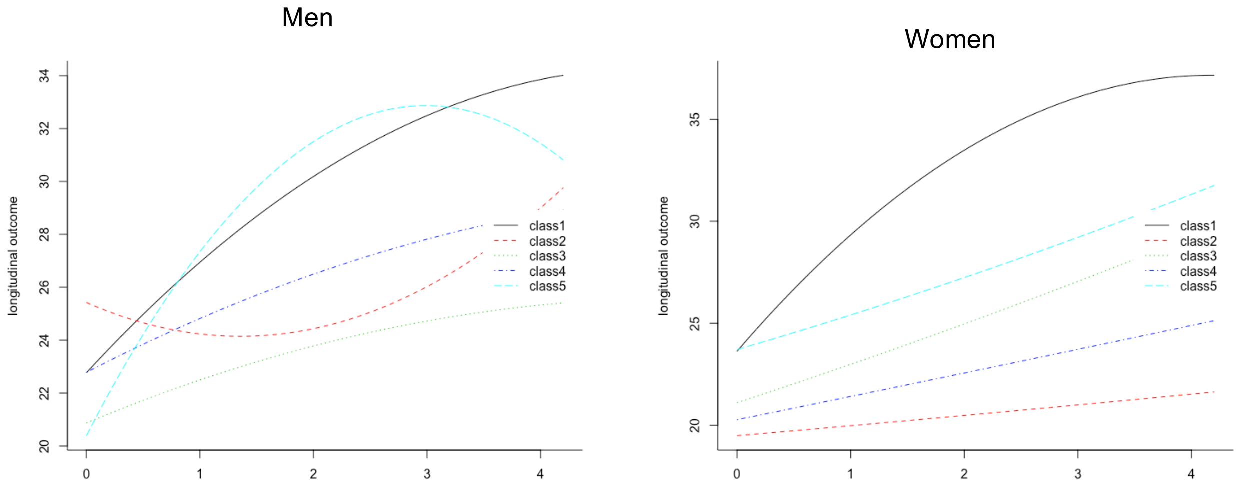

er review only

REFERENCES

1. Kubzansky LD, Gilthorpe MS, Goodman E. A prospective study of psychological distress and weight status in adolescents/young adults. Annals of Behavioral Medicine 2012;43(2):219-228.

2. Kelly SP, Graubard BI, Andreotti G, Younes N, Cleary SD, Cook MB. Prediagnostic Body Mass Index Trajectories in Relation to Prostate Cancer Incidence and Mortality in the PLCO Cancer Screening Trial. J Natl Cancer Inst 2017;109(3).

3. Song M, Willett WC, Hu FB, Spiegelman D, Must A, Wu K, et al. Trajectory of body shape across the lifespan and cancer risk. Int J Cancer 2016;138(10):2383-95.

4. Song M, Hu FB, Wu K, Must A, Chan AT, Willett WC, et al. Trajectory of body shape in early and middle life and all cause and cause specific mortality: results from two prospective US cohort studies. BMJ 2016;353:i2195.

5. Zheng H, Tumin D, Qian Z. Obesity and Mortality Risk: New Findings From Body Mass Index Trajectories. American Journal of Epidemiology 2013;178(11):1591-1599.

6. Elsensohn M-H, Klich A, Ecochard R, Bastard M, Genolini C, Etard J-F, et al. A graphical method to assess distribution assumption in group-based trajectory models. Statistical methods in medical research 2016;25(2):968-982.

7. Vistisen D, Witte DR, Tabak AG, Herder C, Brunner EJ, Kivimaki M, et al. Patterns of obesity development before the diagnosis of type 2 diabetes: the Whitehall II cohort study. PLoS Med 2014;11(2):e1001602.

8. Lubke G, Neale MC. Distinguishing between latent classes and continuous factors: Resolution by maximum likelihood? Multivariate Behavioral Research 2006;41(4):499-532.

9. Peugh J, Fan X. How Well Does Growth Mixture Modeling Identify Heterogeneous Growth Trajectories? A Simulation Study Examining GMM's Performance Characteristics. Structural Equation Modeling: A Multidisciplinary Journal 2012;19(2):204-226.

10. Peugh J, Fan X. Enumeration Index Performance in Generalized Growth Mixture Models: A Monte Carlo Test of Muthén's (2003) Hypothesis. Structural Equation Modeling: A Multidisciplinary Journal 2015;22(1):115-131.

11. Jedidi K, Ramaswamy V, Desarbo WS. A maximum likelihood method for latent class regression involving a censored dependent variable. Psychometrika 1993;58(3):375-394.

For peer review only

For peer review only - http://bmjopen.bmj.com/site/about/guidelines.xhtml
